# Neurotransmitter classification from electron microscopy images at synaptic sites in *Drosophila melanogaster*

**DOI:** 10.1016/j.cell.2024.03.016

**Published:** 2024-05-09

**Authors:** Nils Eckstein, Alexander Shakeel Bates, Andrew Champion, Michelle Du, Yijie Yin, Philipp Schlegel, Alicia Kun-Yang Lu, Thomson Rymer, Samantha Finley-May, Tyler Paterson, Ruchi Parekh, Sven Dorkenwald, Arie Matsliah, Szi-Chieh Yu, Claire McKellar, Amy Sterling, Katharina Eichler, Marta Costa, Sebastian Seung, Mala Murthy, Volker Hartenstein, Gregory S.X.E. Jefferis, Jan Funke

**Affiliations:** 1 HHMI Janelia Research Campus, Ashburn, VA, USA; 2 Institute of Neuroinformatics UZH/ETHZ, Zurich, Switzerland; 3 Neurobiology Division, MRC Laboratory of Molecular Biology, Cambridge, UK; 4 Centre for Neural Circuits and Behaviour, The University of Oxford, Tinsley Building, Mansfield Road, Oxford OX1 3SR, UK; 5 Department of Neurobiology and Howard Hughes Medical Institute, Harvard Medical School, Boston, MA, USA; 6 Drosophila Connectomics Group, Department of Zoology, University of Cambridge, Cambridge, UK; 7 Princeton Neuroscience Institute, Princeton University, Princeton, NJ, USA; 8 Department of Molecular Cell and Developmental Biology, University of California, Los Angeles, Los Angeles, CA, USA

**Keywords:** neuroscience, machine learning, electron microscopy, *Drosophila melanogaster*, neurotransmitter, explainable AI

## Abstract

High-resolution electron microscopy of nervous systems has enabled the reconstruction of synaptic connectomes. However, we do not know the synaptic sign for each connection (i.e., whether a connection is excitatory or inhibitory), which is implied by the released transmitter. We demonstrate that artificial neural networks can predict transmitter types for presynapses from electron micrographs: a network trained to predict six transmitters (acetylcholine, glutamate, GABA, serotonin, dopamine, octopamine) achieves an accuracy of 87% for individual synapses, 94% for neurons, and 91% for known cell types across a *D. melanogaster* whole brain. We visualize the ultrastructural features used for prediction, discovering subtle but significant differences between transmitter phenotypes. We also analyze transmitter distributions across the brain and find that neurons that develop together largely express only one fast-acting transmitter (acetylcholine, glutamate, or GABA). We hope that our publicly available predictions act as an accelerant for neuroscientific hypothesis generation for the fly.

## Highlights

• Machine learning identifies synaptic transmitters from electron micrographs
• Six transmitters predicted across the whole fly brain connectome
• Explainable AI reveals ultrastructural differences between transmitter identities
• Fly brain hemilineages predominantly express one fast-acting transmitter

## Graphical abstract

**Figure:**
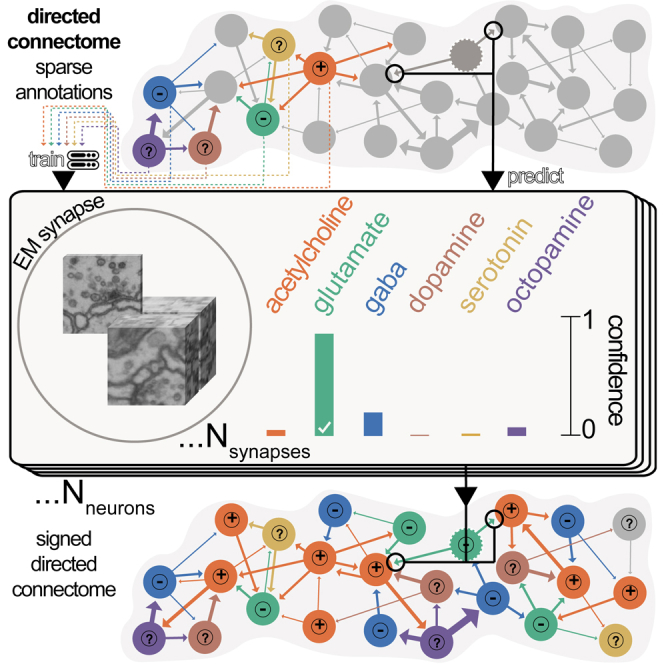

## Teaser

Neurotransmitter identities are predicted directly from electron micrographs with high fidelity across the whole fly brain for acetylcholine, glutamate, GABA, dopamine, serotonin, and octopamine.

## Introduction

Generating a synaptic connectome entails identifying all neurons and synapses in a sample. Recent advances in volume electron microscopy (EM) have enabled connectome generation for entire nervous systems.^1^^,^^2^^,^^3^^,^^4^^,^^5^^,^^6^ Automated methods for segmenting neurons,^7^^,^^8^^,^^9^^,^^10^^,^^11^ detecting synapses,^12^^,^^13^^,^^14^^,^^15^^,^^16^ and proofreading^17^ have significantly reduced the human effort required. These methods have been applied to create connectomes for the *Drosophila melanogaster* brain^6^^,^^18^^,^^19^ and its ventral nerve cord.^5^^,^^20^ However, EM does not directly tell us about gene expression, most crucially the transmitter pathways active in each neuron. This gap hinders our understanding of key neurobiological processes relevant to circuit function.

The action that a neuron has on its downstream targets depends on the transmitter it releases and the postsynaptic receptors that receive it. The so-called *classical* fast-acting transmitters (i.e., acetylcholine, glutamate, and GABA) are most common.^21^^,^^22^ They are contained in small, clear vesicles. Monoamines such as dopamine, serotonin, and octopamine are packaged into clear core or small dense core vesicles.^23^ About 53 neuropeptides and peptide hormones^24^^,^^25^^,^^26^ are contained in larger dense core vesicles.^27^^,^^28^ While co-transmission of a small molecule transmitter and a neuropeptide is common,^21^^,^^22^^,^^29^ the usage of acetylcholine, glutamate, and GABA is largely mutually exclusive.^30^^,^^31^ Therefore, Dale’s law, an expectation that each neuron expresses a single small molecule transmitter, often guides our hypotheses about neuronal function.^32^^,^^33^ We expect that this transmitter expression will be stereotyped for cell types across individuals, given their shared gene expression profiles.^34^ We also expect transmitter expression to be organized at higher levels, for example by hemilineage. Hemilineages are basic developmental units of the insect brain and typically consist of dozens of cell types. In the ventral nerve cord Lacin et al.^31^ comprehensively showed that only one of acetylcholine, glutamate, or GABA is expressed per hemilineage. By analogy with Dale’s law, we call this observation Lacin’s law. It was unclear whether Lacin’s law also holds in the brain.

In larger organisms, experts can distinguish excitatory and inhibitory transmitters based on synaptic vesicle ellipticity^35^^,^^36^^,^^37^ or synapse symmetry.^38^ However, in invertebrates transmitter identity cannot be consistently identified by human annotators from electron micrographs. Instead, investigators use molecular biology and light microscopy pipelines to link RNA expression data or immunoreactivity to proteins involved in transmitter biosynthesis with specific neuronal morphologies.^22^^,^^39^^,^^40^^,^^41^^,^^42^^,^^43^^,^^44^ Standard high-throughput methods such as single-cell RNA-seq cannot be used since they do not retain morphology information. Therefore, researchers profile sparse genetic driver lines,^45^^,^^46^ which target only a few neurons, followed by accurate morphological matching to EM reconstructions.^47^^,^^48^ Since these experimental pipelines can take weeks per cell type and are limited by the availability of sparse genetic driver lines, they do not scale to whole nervous system discovery. Consequently, the transmitter identity is known for only ∼700 of the ∼7,000 cell types of the adult *D. melanogaster* central brain.^49^^,^^50^ However, this subset provided ground truth that we can use to learn the features of specific transmission types, by mapping known transmitter identities to previously localized presynaptic sites in two brain datasets (FAFB [the Full Adult Fly Brain]^6^ and the HemiBrain^51^).

## Results

### Assembling ground-truth neurotransmisson data

**Figure 1:**
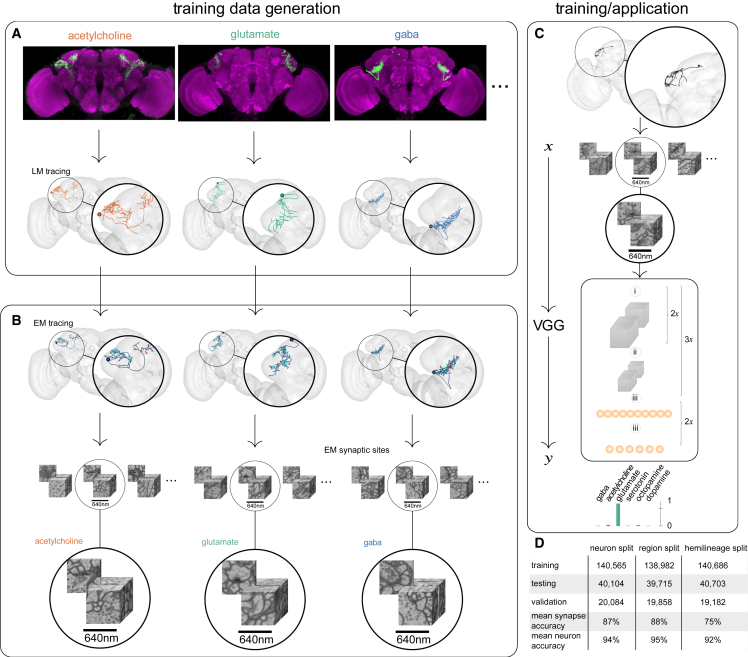
Method overview We assembled a dataset of neurons with known transmitter expression (see STAR Methods) in two *D. melanogaster* brain EM datasets (FAFB and HemiBrain) and retrieved corresponding synaptic locations. (A) Typically, neurons had been genetically tagged to identify their transmitter identity and reconstruct their coarse morphology using light microscopy (Data S1). (B) Light microscopy tracings of neurons are then matched to corresponding EM reconstructions with annotated synaptic locations, yielding a dataset of EM volumes of synaptic sites with known transmitter identity. (C) We used the resulting pair (*x*, *y*) where *x* is a 3D EM volume of a synaptic site and *y* is the transmitter of that synaptic site (one of GABA, acetylcholine, glutamate, serotonin, octopamine, or dopamine) to train a 3D VGG-style deep neural network to assign a given synaptic site *x* to one of the six considered transmitters. We used the trained network to predict the transmitter identity of synapses from neurons with so far unknown transmitter identity. Panels i, ii, and iii denote convolution, down-sampling, and fully connected layers, respectively. (D) Overview of our results on the FAFB dataset. Shown are the number of presynapses for training, testing, and validation as well as average synapse and neuron classification accuracy on the testing set for each data split. See also Data S2.

We compiled a list of 356 *D. melanogaster* neuronal cell types from 21 studies (Data S1), selecting those with robust transmitter data. We identified these same cell types within the FAFB-Catmaid and HemiBrain datasets (Figure 1A). We selected cell types with clear transmitter data from RNA expression or immunohistochemistry, complemented by specific cell type targeting through the GAL4/split-GAL4 system.^46^ We did not generally pursue types reported to exhibit co-transmission (see STAR Methods). Given that *D. melanogaster* neurons are highly stereotyped,^50^ these cell types were identifiable in our EM datasets. We chose to proceed with transmitters supported by at least 10 EM reconstructions per dataset, i.e., acetylcholine, glutamate, dopamine, serotonin, and octopamine (Data S1, see STAR Methods). All presynapses for each neuronal reconstruction were assumed to use the single small-molecule transmitter reported in the literature (Figure 1B). In total, we matched 3,025 FAFB-Catmaid neuronal reconstructions (211,564 synapses) and 5,902 HemiBrain reconstructions (840,535) to cell types with a known transmitter (Data S2).

We took a slightly different approach in selecting presynapses from our two datasets. For HemiBrain, we used automatically predicted presynaptic sites across many brain cell types, of which a proportion are false detections.^13^ In FAFB, however, we used manually placed synaptic markers laid by human researchers from a smaller pool of manually reconstructed neurons (see STAR Methods). Therefore, compared with HemiBrain, our FAFB-Catmaid dataset contained higher-quality synapses from a smaller number of neurons. Note that in later results, to work with the full connectome, we explored transmitter predictions across automatically detected presynapses^12^ in the newer FAFB-FlyWire reconstruction project.^17^^,^^18^^,^^50^ After building our ground truth, we expanded our literature review to find 268 more cell types with the reported transmission of one of our six transmitters (Data S7), which we could use to further validate our results.

### Network architecture, training, testing, and validation datasets

For each transmitter y∈ {GABA, acetylcholine, glutamate, serotonin, octopamine, dopamine}, we partitioned the data into training, testing, and validation sets by randomly assigning entire neurons (neuron split), such that approximately 70% of presynapses were used for training, 10% for validation, and the remaining 20% for testing. This approach mirrors real-world scenarios where we typically know the transmitter of an entire neuron and are interested in predicting the transmitter of a different neuron. We employed a 3D deep convolutional network based on the Visual Geometry Group (VGG) architecture^52^ to predict transmitter identity from cubes of EM image data (edge length 640 nm), each centered on a presynaptic site (Figure 1C). The cube size was chosen to be large enough to provide surrounding context, including synaptic features like vesicles, T-bars, clefts, and postsynaptic densities, while at the same time being small enough to fit into the limited memory of a GPU. The network consisted of four functional blocks, each with two 3D convolution operations, batch normalization, ReLU non-linearities, and subsequent max pooling with a downsampling factor of 2, except for FAFB where we limited downsampling to the x and y dimensions for the first three blocks to account for image voxel anisotropy. The last block was followed by three fully connected layers with dropout (*p* = 0.5) applied to the last one. We trained the network to minimize cross-entropy loss over the six classes (GABA, acetylcholine, glutamate, serotonin, octopamine, and dopamine) using the Adam optimizer.^53^ We trained for a total of 500,000 iterations in batches containing eight samples and selected the iteration with the highest validation accuracy for testing.

**Figure 2:**
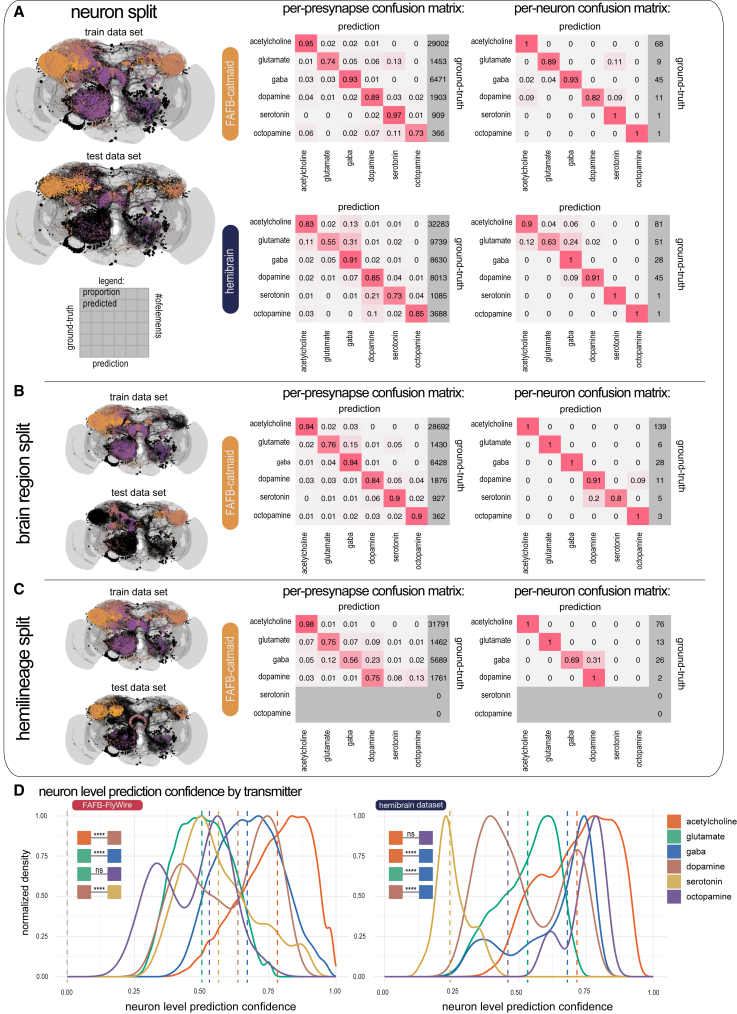
The accuracy of the trained classifier on a per-presynapse and per-neuron basis (A) Left: visualization of the training (upper) and testing (lower) data (split by entire neurons) that we used for the results in this manuscript. Presynapse locations are color coded according to their z-depth; anterior-posterior shown as purple-orange, neuron skeletons in black. Right: confusion matrices for the trained classifier on the testing data, shown per presynapse and as a majority vote per neuron, on datasets FAFB and HemiBrain. We considered only those neurons with more than 30 presynapses. (B) Classification results on alternative training and testing data (split by brain regions) from FAFB. (C) Same as (B) but split by hemilineage. It was not possible to generate a fully balanced split and as a result there are no serotonin and octopamine neurons in the testing set, as indicated by the grayed-out rows. (D) The distribution of neuron-level confidence scores by transmitter, across our pool of central brain neurons in the FlyWire and HemiBrain datasets (FAFB-FlyWire, 136,927; HemiBrain, 24,666). Vertical dashed line, median value. Colored boxes with stars indicate statistical comparisons, Wilcoxon two-sample tests (n.s., not significant; ^∗^*p* ≤ 0.05; ^∗∗∗∗^*p* ≤ 0.00001). See also Figure S1 and Data S3 and S4.

For the FAFB neuron split, the testing set consisted of a total of 40,104 presynapses from 185 neurons that the network was not trained on. The network achieved an average per-transmitter accuracy of 87% on FAFB and 78% on HemiBrain. We assigned each neuron with over 30 presynapses in the testing set a transmitter through a majority vote of its presynapses, yielding an average accuracy of 94% for transmitter prediction per neuron on FAFB-Catmaid and 91% on HemiBrain (Figure 2A). Our goal was to train a network with high prediction accuracy based on ultrastructural features of synapses. Cytological correlates of synapse location or neuron development might have driven transmitter identification by our network. We, therefore, split the data by the neuropil (i.e., brain region) location of synpases^54^ (Figure 2B) and their developmental origin (hemilineage) (Figure 2C). Accuracy in these split datasets remained high (Figure 1D), indicating that the network is unlikely to use related features.

Applying Dale’s law to the entirety of both datasets, we adopted the most frequent synapse-level transmitter prediction as the neuron-level transmitter prediction and developed a confidence score based on the proportion of presynapses that “voted” for the neuron-level transmitter prediction and our confusion matrices (see STAR Methods). The distribution of confidence scores across the two datasets suggested the network was most confident in acetylcholine predictions (Figure 2D). In general, we found excellent agreement with the literature (Data S7). We predicted most known cholinergic cell types correctly (FAFB-FlyWire, 91%; HemiBrain, 91%), as well as most glutamatergic (FAFB-FlyWire, 91%; HemiBrain, 95%), GABAergic (FAFB-FlyWire, 96%; HemiBrain, 97%), dopaminergic (FAFB-FlyWire, 90%; HemiBrain, 85%), and octopaminergic (FAFB-FlyWire, 85%; HemiBrain, 100%) cell types (Figures S2D and S2F). Notably, the optic lobe, which has the most known transmitter assignment,^39^ was largely not used in our ground truth due to a relative paucity of FAFB-Catmaid reconstructions and its absence from the HemiBrain dataset. Nevertheless, 96% of ∼29,000 cholinergic optic lobe neurons were predicted correctly, as well as 87% of ∼3,600 GABAergic neurons and 91% of ∼1,600 glutamatergic neurons.

**Figure S1:**
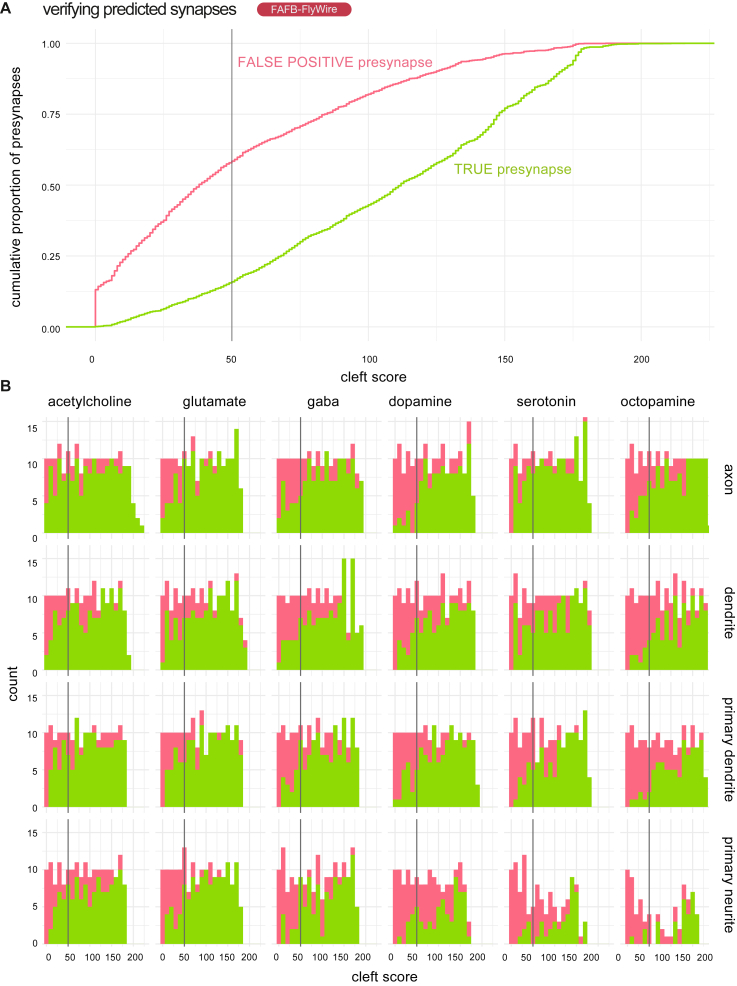
Human review of auto-detected presynapses in the FAFB-FlyWire dataset, related to Figures 3 and 2 (A) Empirical cumulative density distribution curve for review of 4,306 automatically detected preynapses from the FAFB-FlyWire dataset. A presynapse comprises the synaptic machinery and vesicles on the source neuron’s side of the synaptic cleft. Detected preynapses have a “cleft score” that ranges between 0 to over 200, which indicates how discriminable the synaptic cleft at the presynaptic site is for the detection network.^12^ Our threshold of 50 is indicated by a vertical grey line. Green, determined to be a true presynapse by a human annotator; pink, determined to not be a true presynapse. (B) Rates of false presynapse detection across cleft scores, transmitter types, and compartments. We sampled ∼180 for each set of conditions. We sampled ∼10 presynapses per cleft score bin (width 10), presynaptic transmitter prediction type (columns) and neuronal compartment (rows). Histograms show the number of presynapses determined to be real (green) or not (red) by a human annotator (A.S.B). Presynapses were reviewed using the flywire interface.^17^

**Figure S2:**
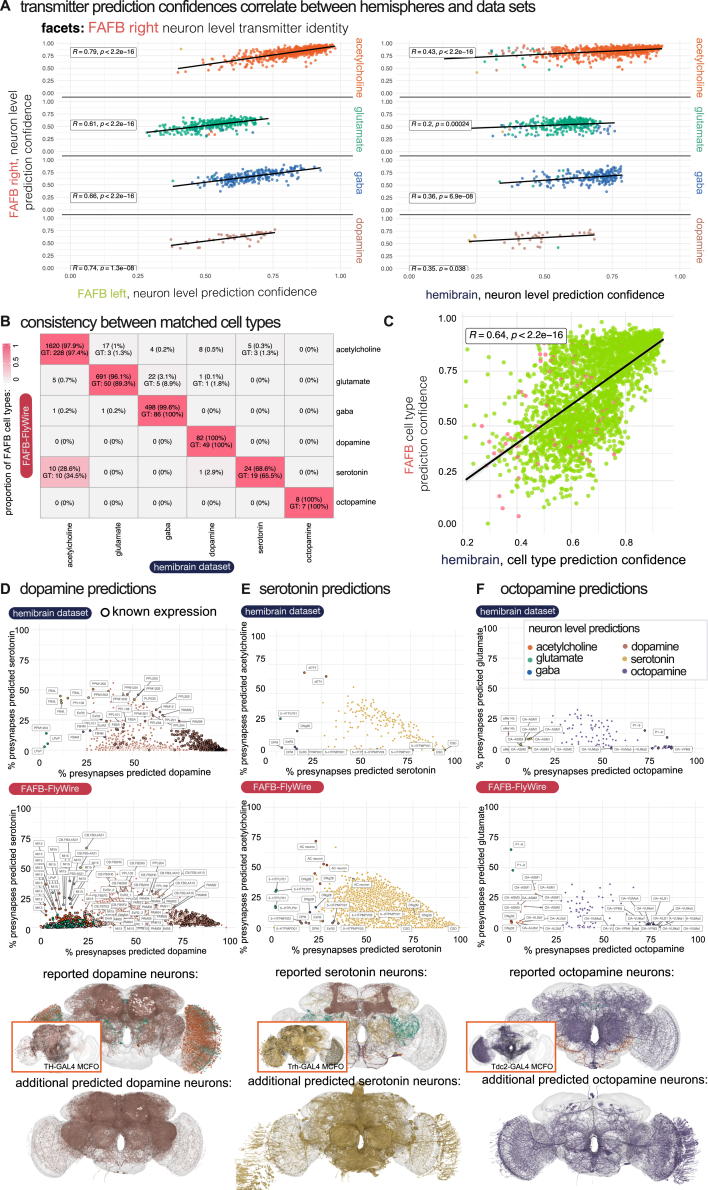
Comparing neurons’ transmitter predictions between connectome datasets from separate animals (FAFB-FlyWire and HemiBrain) and between two hemispheres in the same dataset (FAFB-FlyWire), related to Figures 4 and 5 (A) Left. Scatterplots comparing neuron-level confidence scores in the transmitter prediction of FAFB-FlyWire-right central brain neurons, faceted by the neuron-level transmitter prediction for the FAFB-FlyWire right side homolog. Individual points colored by their FAFB-FlyWire-left or HemiBrain neuron-level transmitter prediction. Only 40 (2.5%) disagree with the FAFB-FlyWire left side prediction and 94 (7.7%) disagree HemiBrain. Right, scatterplots comparing neuron-level confidence scores in the transmitter prediction of HemiBrain dataset central brain neurons. Individual points colored by their HemiBrain side neuron-level transmitter prediction score. Only 94 (7.7%) disagree with the HemiBrain prediction. (B) A confusion matrix showing the neuronal cell type level prediction (mode of the neuron-level transmitter predictions per neuronal cell type) for neuronal cell types in the FAFB-FlyWire and HemiBrain datasets. Cells give the number of cross-matched neuronal cell types we examined, and the number of those present in the ground truth data for at least one of the two datasets. (C) A scatterplot showing the correlation between our mean prediction confidence scores for FAFB-FlyWire and HemiBrain neuronal cell types. Each point is a neuronal cell type identified in both datasets (2626). Green points mean that the transmitter prediction agrees between the two datasets and pink points indicate disagreement. Scatterplots display Pearson’s product-moment correlation, giving R, the coefficient and the associated *p*-value. (D) A look at dopamine predicted neurons. We show two scatterplots using data and predictions from the HemiBrain (upper) and FAFB-FlyWire (lower) datasets. The proportion of presynapses in each neuron (each point) that are predicted as dopamine (X axis) and serotonin (Y-axis). Neurons that have been predicted as dopaminergic, or known as dopaminergic from the literature (dark circles), are shown. Those neurons from the ground truth data are circled with a black ring. Upper brain plot shows neurons known to be dopaminergic (colored by their neuron-level transmitter prediction). The visual system Mi15 neurons are thought to express dopamine and acetylcholine.^39^ Lower, brain plot shows neurons strongly predicted to be dopaminergic (>50% of presynapses ‘voting’ for dopamine), excluding those in the upper plot. Many weakly predicted dopaminergic neurons belong to the central complex and mushroom body, where the density of presynapses from other neurons may have contributed to possible mis-predictions (see STAR Methods). Image inset with orange border shows light-level single neuron skeletons from MultiColor FlpOut experiments from the FlyCircuit project,^212^ from the TH-GAL4 line which labels most putative dopaminergic neurons in the fly brain. All neurons have been transformed onto the right hemisphere of the standard FlyCircuit template brain, FCWB. We have found the FlyCircuit MultiColor FlpOut data (23513 morphologies) to be unfaithful to the expected expression patterns for Cha-GAL4 (cholinergic neurons), vGlut-GAL4 (glutamatergic neurons) and Gad1-GAL4 (GABAergic neurons) and therefore of limited use in assigning transmitters, but provide the data for monoamines here to give the reader some impression of what whole brain expression patterns may look like. (E) Same as *a*, but for serotonin predictions. Some PPL101-6 neurons are may co-express dopamine and serotonin but are predicted as dopaminergic. Some known serotonergic neurons have low proportions of presynapses predicted as serotonergic. Flycircuit neurons from the Trh-GAL4 driver shown in inset. Trh is involved in serotonin biosynthesis. (F) Same as *a*, but for octopamine predictions. Flycircuit neurons from the Tdc2-GAL4 driver shown in inset, which labels putative octopaminergic and tyraminergic neurons. Most octopamine neurons have been identified in prior work. Many of our octopamine predictions (no dark circle) indicate neurons that express some other dense core vesicle transmitter in abundance, for example, PI neurons which express an insulin-like peptide. Interestingly, the putative octopaminergic aMe14b neurons^138^ (also known as OA-AL2b2 neurons) are predicted for acetylcholine. Busch et al. noted that they might not be octopaminergic, as not all neurons in cluster AL2 of NP7088 are OA-immunoreactive, and because OA-AL2b2 (HemiBrain type: aMe14b) was identified in NP7088, but not in tdc2-GAL4. OA-ASM (HemiBrain type: aMe14b) neurons also are not predicted octopaminergic, but serotonergic. On OA-ASM, Busch et al. note: “There are 8 OA-immunoreactive somata localized to the anterior superior medial protocerebrum uniquely labeled by tdc2-GAL4 (the ASM cluster). Yet they are not necessarily octopaminergic, as there are GAL4-positive neurons without OA-immunoreactivity in this cluster."

However, we noticed a few clear mispredictions and discrepancies, most commonly in cases of suspected co-transmission (see STAR Methods). For example, Kenyon cells had been mispredicted for dopamine in both HemiBrain and FAFB-FlyWire rather than acetylcholine,^55^ some known serotonergic neurons (Figure S2E) were not predicted for serotonin in either dataset,^56^^,^^57^^,^^58^^,^^59^^,^^60^ many first-order sensory neurons and antennal lobe local neurons were mispredicted for serotonin rather than acetylcholine in both datasets^61^^,^^62^^,^^63^^,^^64^ and some intrinsic neurons of the fan-shaped body were mispredicted in HemiBrain but not in FAFB-FlyWire and vice versa. Overall, serotonin was our least reliable prediction (FAFB-FlyWire, 33%; HemiBrain, 38%) likely due to its relative paucity in our ground-truth data. Our results for a full ventral nerve cord (MaleVNC, 82% accuracy, limited to acetylcholine, glutamate, and GABA) are reported elsewhere.^5^^,^^65^

### Classifier synapse feature analysis

**Figure 3:**
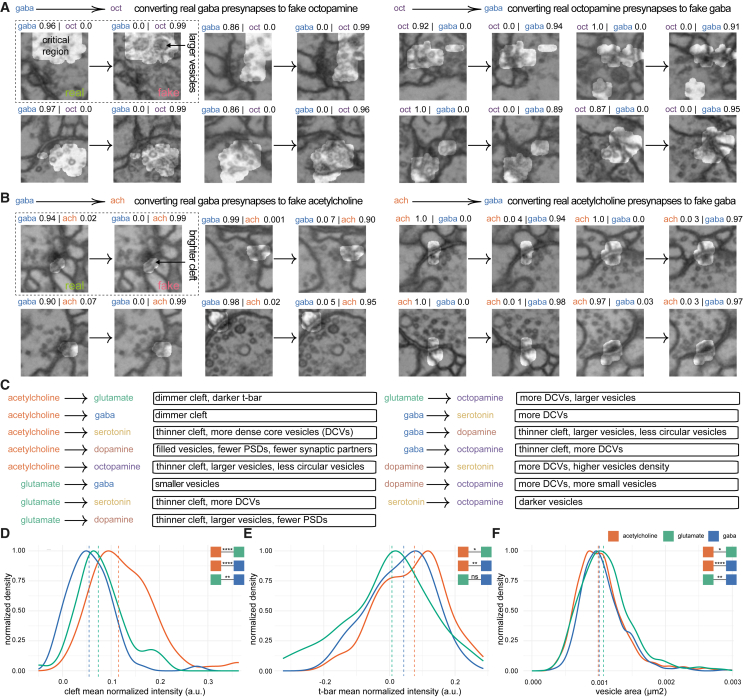
Classifier feature analysis using a discriminative attribution method (A) Example translations of real synapse images to fake counterfactual images. Highlights show attribution masks indicating the most important changes between the two classes. Classifier scores are shown above each image. Left, two columns show the translation of real GABA synapses into counterfactual octopamine synapses. Right, same as left but for octopamine to GABA. (B) Same as (A) but for GABA-acetylcholine. (C) Pairwise differences between transmitters, found through manual inspection of real and counterfactual images. Dense core vesicles, DCVs; postsynaptic densities, PSDs. (D) Normalized density plot showing the distribution of cleft intensity among original synapse images. Number of annotated synapses: acetylcholine 84, glutamate 61, GABA 74. (E) Same as (C) for T-bar intensities. Number of annotated synapses: acetylcholine 85, glutamate 62, GABA 75. (F) Same as (C) for vesicle sizes. Number of annotated vesicles: acetylcholine 1,729, glutamate 1,153, GABA 1,382. Vertical dashed line, median value. Colored boxes with stars indicate statistical comparisons, Wilcoxon tests. Note that the vesicle size comparison assumes that vesicle sizes from the same synapses are conditionally independent given the transmitter (n.s., not significant; ^∗^*p* ≤ 0.05; ^∗∗^*p* ≤ 0.001; ^∗∗∗∗^*p* ≤ 0.00001). See also Figure S1 and Data S8.

The classifier’s high accuracy on test datasets raised questions about how transmitter classes are discriminated, as human annotators cannot reliably determine transmitter identity from EM alone. We reasoned that identifying the visual features that our network used to label transmitters could verify that decisions were not based on class-irrelevant confounders and discover unknown ultrastructural differences between synapse classes. To visualize these features, we explored *post hoc* single-input attribution methods that derive an attribution map for a single input image, highlighting image areas crucial for classification. Existing methods^66^^,^^67^^,^^68^^,^^69^ did not provide images we could interpret since the highlighted areas were too large and variable. This may be because single-input attribution methods are subject to highlighting non-class-specific “distractors,”^70^ e.g., features stemming from different orientations of the synapse, section thickness, or intensity variations. We introduced an attribution method to focus on class-relevant features between pairs of classes while disregarding distractors^71^ and used the FAFB dataset for its superior x,y resolution. Given a real image ***x***_R_of a class *y*_R_, we first created a counterfactual image ***x***_C_ by translating ***x***_R_ into an image of another class *y*_C_ using a CycleGAN,^72^ resulting in paired images of different transmitter types. Crucially, this domain translation of an image from class *y*_R_ to class *y*_C_ keeps class-irrelevant distractors (e.g., the orientation of the synapse) intact. Class-relevant features, however, are changed due to the adversarially trained discriminator of the CycleGAN. We used our previously trained classifier to confirm that the domain translation was successful: we filtered all images such that ***x***_R_ was classified as *y*_R_ and the counterfactual ***x***_C_ as *y*_C_. We then identified a small region in ***x***_C_, that when swapped with ***x***_R_ changed the prediction of the trained classifier (Figures 3A and 3B). Specifically, we identified a minimal binary mask ***m***, such that the hybrid image $x_{H}=m\cdot x_{R}+\left(1-m\right)\cdot x_{C}$ was classified as *y*_R_. To find ***m***, we used a modified version of the DeepLift method, where we used the counterfactual ***x***_C_ as the neutral ref*.*^71^.

This method allowed us to manually identify at least one distinguishing feature (Figure 3C) between each pair of transmitters (see Data S8). We only included features consistently observed in both directions, e.g., translating a real GABA image to acetylcholine results in a brighter cleft and translating a real acetylcholine image to GABA produces a darker cleft. Comparing transmitter identities in paired images allowed us to observe features as subtle as sub-pixel changes in vesicle diameters (e.g., between GABA and glutamate), which would be near impossible to pick up in unpaired images. We confirmed the identified features between the classical transmitters GABA, acetylcholine and glutamate on the original synapse images by manually segmenting the synaptic cleft, vesicles, and T-bars of 222 synapse images (75 GABA, 85 acetylcholine, 62 glutamate; annotators were blind to the predicted transmitter class). We found strong support for each of the identified features between those transmitters (Figures 3D–3F): acetylcholine has a brighter cleft than GABA and glutamate (*p* ≤ 0.0001), glutamate has larger vesicles than GABA (*p* ≤ 0.001) and glutamate has a darker T-bar than acetylcholine (*p* ≤ 0.001).

### Comparing neurotransmitter predictions for neuron homologs across datasets

**Figure 4:**
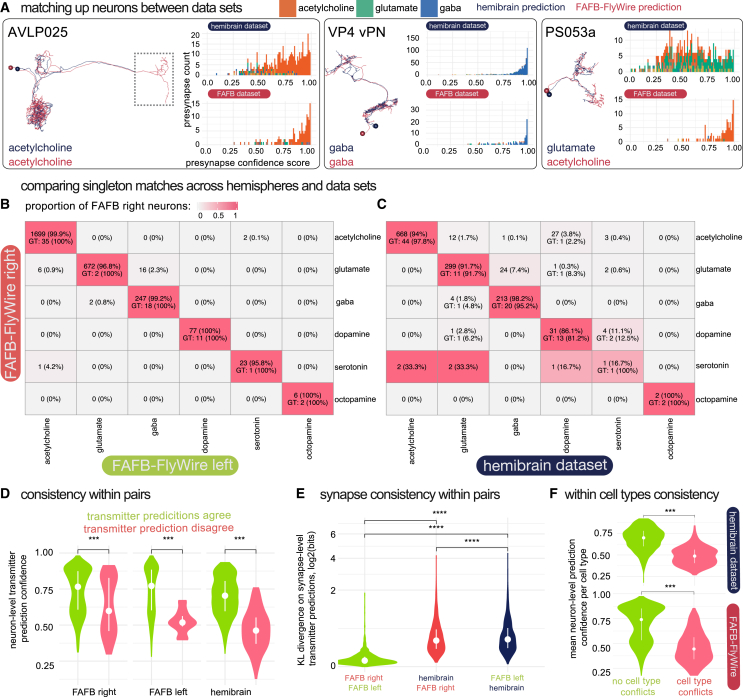
Comparing neuron-level transmitter predictions between connectome datasets from separate animals and between hemispheres (A) Images of co-registered, matched neurons between the HemiBrain (navy) and the FAFB-FlyWire (red) datasets. Histograms show synapse-level transmitter prediction scores for exemplar pairs. Neurons can be matched despite missing data (left, grey dashed box). PS053a has conflicting neuron-level transmitter predictions. (B) Confusion matrix compares matched singleton FAFB-FlyWire-right and FAFB-FlyWire-left pairs’ neuron-level transmitter predictions (1,586 pairs). (C) Confusion matrix comparing matched FAFB-FlyWire-right and HemiBrain-right neuron-level transmitter predictions (1,318 pairs). Cells colored by the proportion of FAFB-FlyWire right neurons of each transmitter type (row normalized) that are matched to its homolog-columns give homolog prediction. (D) Neuron-level transmitter prediction scores between matched singletons that have (red, right) or do not have (green, left) a conflict between their neuron-level transmitter predictions, across all three hemispheres. Matches:mismatches across all comparisons for FAFB-FlyWire right: 2,650:170 FAFB-FlyWire left, 1,562:40, and HemiBrain neurons, 1,088:130. (E) Comparison of similarity scores for matches (Kullback-Leibler divergence on synapse-level transmitter prediction scores). (F) The neuron-level transmitter prediction consistency among cell types that have multiple repeats, i.e., not singletons. Green, the mean neuron-level transmitter prediction confidence for cell types where all members of the type are predicted to use the same transmitter. Red, the mean neuron-level transmitter prediction confidence for cell types where not all members of the type are predicted to use the same transmitter. Violin plots show the median value (dot) and the inter-quartile range (line, 25^th^ to 75^th^ percentiles). Data were compared using Wilcoxon two-sample tests (n.s., not significant; ^∗∗∗^*p* ≤ 0.0001; ^∗∗∗∗^*p* ≤ 0.00001. See also Figure S2 and Data S7.

The insect brain consists of thousands of isomorphic cell types. Every cell type has a copy on each hemisphere. These cell types contain only a few neurons (median, 2, IQR, 2, neurons per cell type per hemisphere for the HemiBrain dataset, excluding the largest outlier classes of Kenyon cells and sensory receptor neurons), often only a single neuron per hemisphere (41% of cell types in HemiBrain). We refer to these neurons as singletons. They provide a natural mechanism by which to test whether our transmitter predictions are consistent as, being unique on each hemisphere, they can be unambiguous matching to their homologs between hemispheres and brains (Figure 4A). We matched 1,586 right hemisphere singletons to their left hemisphere homologs in FAFB-FlyWire and 1,318 to their HemiBrain homologs.^50^

We found good agreement between left-right matched FAFB-FlyWire singletons (Figure 4B) and FAFB-HemiBrain matched singletons (Figure 4C) for cholinergic, glutamatergic, and GABAergic pairs. Inconsistent results between matched neurons were more common with singletons predicted to express dopamine, serotonin, or octopamine. The average confidence score for FAFB-FlyWire right (mean, 0.79, SD, 0.16), FAFB-FlyWire left (mean, 0.79, SD, 0.17), and HemiBrain (mean, 0.67, SD, 0.14) neurons were significantly higher when there was no mismatch between our paired neurons than when there was a conflict (FAFB-FlyWire right: mean, 0.62, SD, 0.22; FAFB-FlyWire left: mean, 0.47, SD, 0.14; hemibrain: mean, 0.43, SD, 0.13) (Figure 4D).

We found that matched singleton pairs and cell types correlated in their neuron-level transmitter prediction scores (Figures 4D–4F and S2A). When the network was less confident it was consistently less confident for both members of a pair, both across hemispheres and datasets, although within-dataset comparisons were more similar (Figure 4E). In cases where changes in the confidence score were correlated, the reason is more likely to be biological than dataset specific. For example, low, correlated scores could indicate that the network has encountered a biological situation that is rare in, or outside of, our training data.

We also examined isomorphic cell types in the HemiBrain dataset for which there was more than one member of the cell type per hemisphere (Figures 4F and S2B). Only 14% of cell types with more than one member per hemisphere did not have the same neuron-level transmitter prediction for each member. Moreover, we matched 2,626 neuronal cell types between FAFB-FlyWire and the HemiBrain datasets and found that 95% agree in their neuron-level transmitter prediction between the two datasets (Figures S2B and S2C). Again, among these conflicted types, the mean neuron-level transmitter prediction score was significantly lower (Figure S2C). Together, this suggests that there may be a biological factor, e.g., the expression of transmitters not in our training data or co-transmission, that leads to lower confidence scores and incorrect or inconsistent predictions with certain cell types, rather than a confound related to the EM data quality.

### An overview of neurotransmitter usage in the nervous system

**Figure 5:**
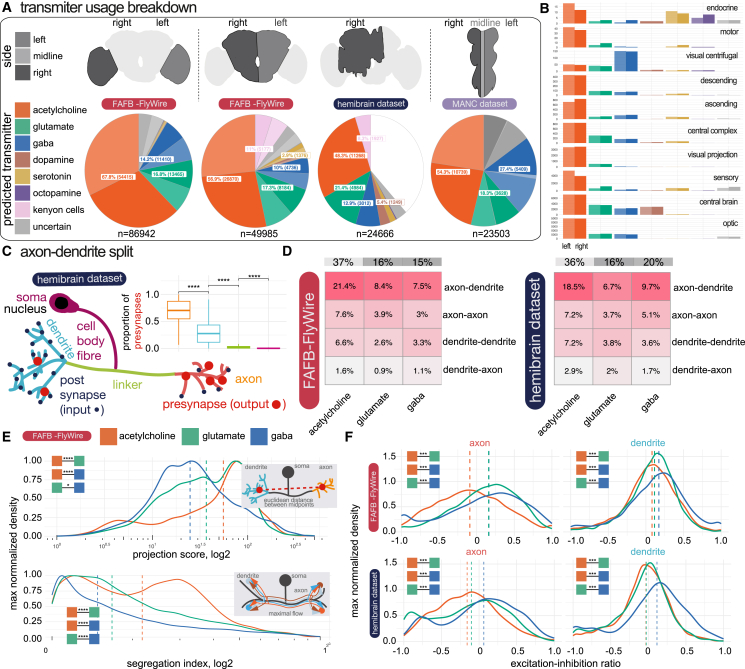
Breakdown of transmitter use across the *D. melanogaster* nervous system (A) Our neuron-level transmitter predictions across the female optic lobes and central brain and a male ventral nerve cord (see STAR Methods). (B) Bar plots for the numbers of neurons predicted for different transmitter usages in each super class in the FAFB-FlyWire dataset.^50^ (C) Schematic of a neuron broken into its neuronal compartments. Inset, the proportion of presynapses in each of the four compartment types. (D) Synaptic budget across different connection types in FAFB-FlyWire (left) and HemiBrain (right). Heatmaps show the proportion of synaptic contacts from neurons of different predicted transmitter types (columns) used in different inter-compartmental connection types (rows). FAFB-FlyWire, 9,123; hemibrain, 10,122 neurons. (E) Scaled density plots showing neuronal polarity by neuron-level transmitter prediction. Upper, distribution of projection scores, which is the distance in Euclidean space between the dendritic an axonic midpoint. Lower, segregation index: the higher the score, the more polarized the neuron.^73^ (F) Scaled density plots showing the distribution of excitation-inhibition balance (proportion of excitatory, acetylcholine, input minus the proportion of inhibitory input; GABA, glutamate) across neuron-level transmitter predictions and compartments. Vertical dashed line, median value. Colored boxes with stars indicate statistical comparisons, Wilcoxon two-sample tests (n.s., not significant; ^∗^*p* ≤ 0.05; ^∗∗∗^*p* ≤ 0.0001; ^∗∗∗∗^*p* ≤ 0.00001). See also Figures S2 and S3 and Data S3 and S4.

**Figure 6:**
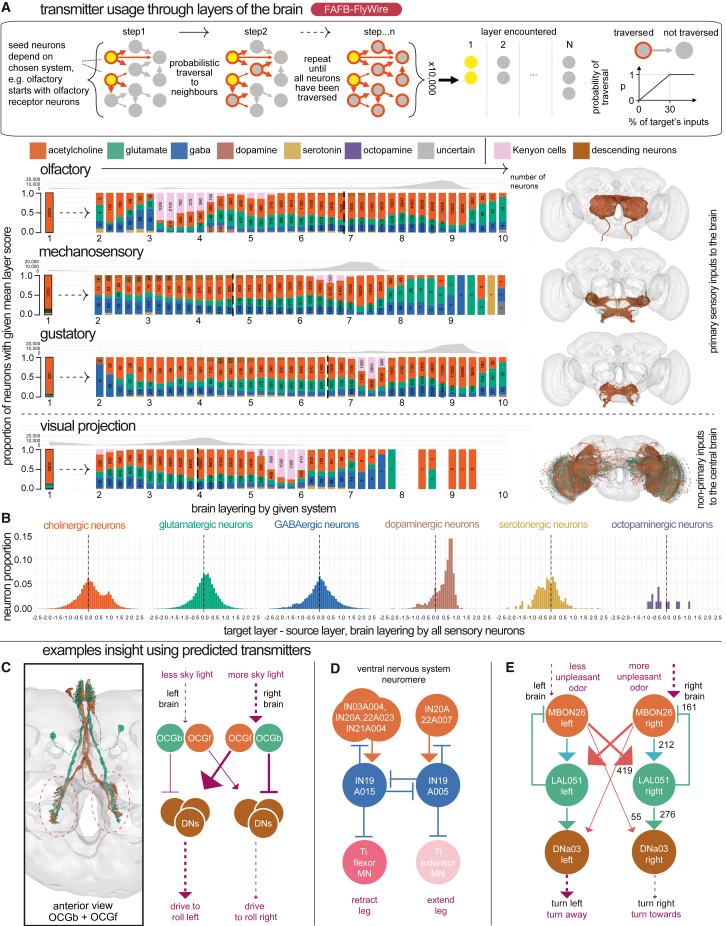
Transmitter usage through sensory layers and specific circuits (A) Schematic depicts the probabilistic graph traversal model used to “layer” different sensory systems, adapted from Schlegel et al.,^74^ underlying data from Dorkenwald et al.^18^ Starting from first-order central brain input neurons, we recorded the mean step (“layer”) at which each subsequent FAFB-FlyWire neuron is encountered by the simulation.^18^ Bar charts show transmitter input across distinguishable sensory systems.^18^ Bars normalized and binned by target neurons’ layer score (width 0.2); text reports neuron count. Kenyon cells are shown in pink and descending neurons, i.e., the last captured point of the sensory-motor transform in the brain, in brown so that the reader can compare layer progression between systems. Vertical line shows the mean descending neuron layer. Olfactory sensory neurons mispredicted for serotonin are corrected to acetylcholine.^64^ “Uncertain” neurons (see STAR Methods) were removed from this analysis. (B) Feedforward and feedback connectivity across sensory systems by neuron-level transmitter prediction. For each unitary neuron-neuron connection (greater than 100) between a source and target neuron, we calculated a target-source layer difference: the layer value^74^ for the target neuron minus the layer value of the source neuron. Y axis gives the proportion of unitary connections in each bin (width 0.1). (C) A potential circuit for righting the fly’s body axis relative to celestial cues. Purple arrow weight indicates activity level. (D) A potential circuit for differential leg extension/retraction control. (E) A potential circuit for steering away from unpleasant odors. Numbers give synaptic counts from HemiBrain. See also Figure S4.

**Figure S3:**
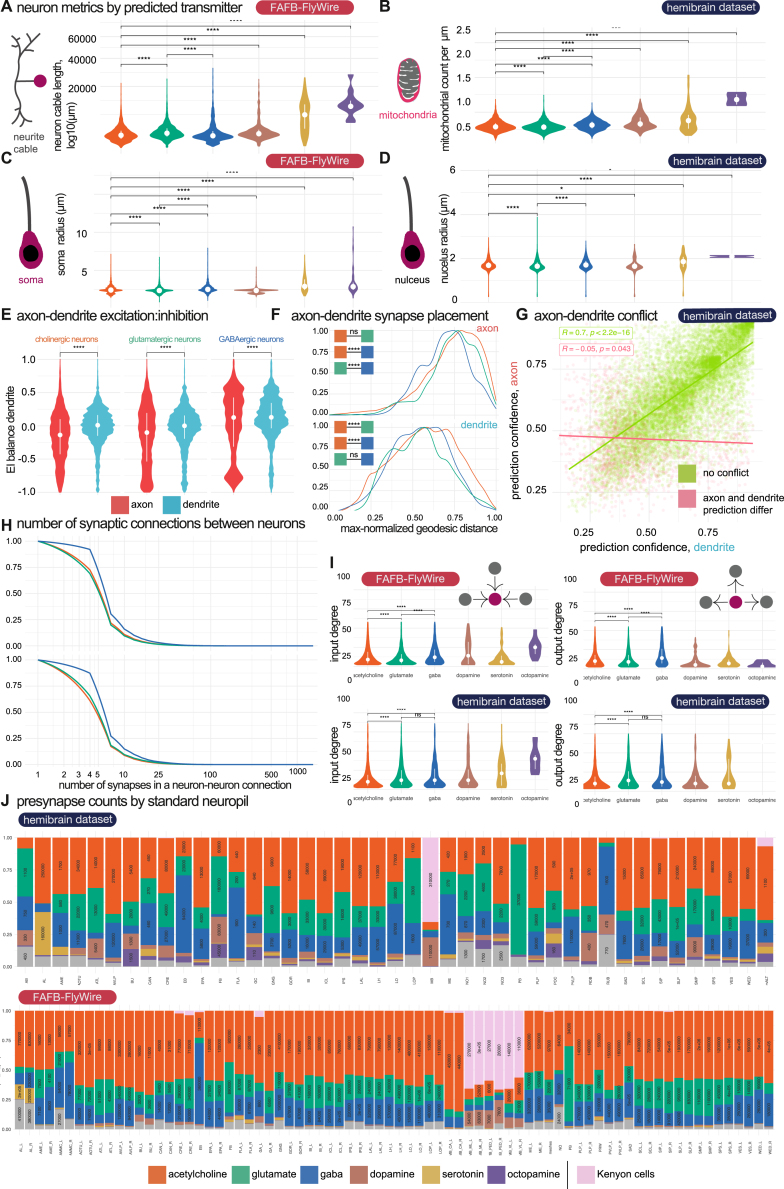
Comparing neuron features across transmitter classes, related to Figure 5 (A) Cable length by neuron-level transmitter prediction. (B) Mitochondria density by neuron-level transmitter predictions. Violin plots show the number of automatically detected mitochondia^135^ per micron cubed. Volume measures per neuron originate from the HemiBrain’s automatically reconstructed 3D neuron volumes.^51^ A mitochondria detection is currently only available in the HemiBrain dataset. The mean number of mitochondria per neuron is 245, s.d. 275. (C) Soma, i.e., neuronal cell body, and (D) nucleus size by neuron-level transmitter predictions. The HemiBrain dataset provides a soma segmentation (left), and the FAFB-FlyWire dataset provides a nucleus segmentation (right)^17^^,^^135^. (E) Violin plots of excitation:inhibition balance by neuron-level transmitter prediction and compartment. (F) Correlation between compartment-level transmitter prediction score for axons and dendrites. Each point is a separate neuron in the HemiBrain dataset, *n* = 10,122. 11.0% disagree on the compartment-level transmitter prediction (red). The scatterplot displays Pearson’s product-moment correlation, giving R, the coefficient and the associated *p*-value. (G) Scaled density plot showing the density of input connections onto all FAFB-FlyWire and HemiBrain neurons (facets) at different synaptic weights (X axis, log2). (H) Scaled density plots showing the max-normalised geodesic distance (the distance along a neuron’s arbour) from input synapses (colored by the source neuron’s neuron-level transmitter prediction) to the target neurons’ cell body. (I) Differences in the number of outgoing and incoming connections by neuron-level transmitter prediction. The input and output degree for a neuron is the number of unitary connections it has incoming and outgoing, respectively (the number of synaptic pairs, regardless of synaptic weight). All source-target connections with a synaptic count ≥ 10 included. Left, boxplots show the distribution of input degrees by the target neurons’ neuron-level transmitter prediction. Right, output degrees by the source neurons’ neuron-level transmitter prediction. A subset of total central brain neurons that were skeletonized (see STAR Methods) were used for this analysis (FAFB-FlyWire: 88,115, HemiBrain: 11,277). (J) Breakdown of neuron-level transmitter predictions by brain region in HemiBrain. Plot shows the proportion of synapses in each HemiBrain neuropil that belong to a neuron of a given neuron-level transmitter prediction (colors). A total of ∼ 4,000,000 were assigned a neuropil and neuron-level transmitter prediction, which helps buffer erroneous synapse-level transmitter predictions. Number labels give the total number of synapses in each group. Not all the standard neuropils^54^ are shown because the HemiBrain only comprises ∼ 1/3 of the central brain. Total number of neuronal reconstructions (see STAR Methods) by dataset: FAFB-FlyWire: 136,927, HemiBrain: 24,666. (J) Breakdown of neuron-level transmitter predictions by brain region in FAFB-FlyWire. Neuropils^54^: AB, asymmetric body, AL, antennal lobe, AME, accessory medulla, AOTU, anterior optic tubercle, ATL, antler, AVLP, anterior ventrolaterla protocerebrum (incomplete in in HemiBrain), BU, bulb, CAN, cantle, CRE, crepine, EB, ellipsoid body, EPA, epaulette, FB, fan-shaped body, FLA, flange, GC, great commissure (incomplete in HemiBrain), GNG, gnathal ganglion (incomplete in HemiBrain), GOR, gorget, IB, inframedial bridge, ICL, inferior clamp, IPS, inferior posterior slope, LAL, lateral accessory lobe, LH, lateral horn, LO, lobula (incomplete in HemiBrain), LOP, lobula plate (incomplete in HemiBrain), ME, medulla (incomplete in HemiBrain), NO1, nodulus compartment 1, NO2, nodulus compartment 2, NO3, nodulus compartment 3, PB, protocerebral bridge, PLP, posterior lateral protocerebrum, POC, posterior optic commissure, PVLP, posterior ventrolateral protocerebrum (incomplete), ROB, round body, RUB, rubus, SAD, saddle, SCL, superior clamp, SIP, superior intermediate protocerebrum, SLP, superior lateral protocerebrum, SMP, superior medial protocerebrum, SPS, superior posterior slope, VES, vest, WED, wedge. Violin plots show the median value (dot) and the inter-quartile range (line, 25^th^ to 75^th^ percentiles). Significance values: ns: *p* > 0.05; ^∗^: p ≤0.05; ^∗∗^: p ≤ 0.01; ^∗∗∗^: p ≤0.001; ^∗∗∗∗^: p ≤ 0.0001.

We next wanted to get an overview of transmitter usage in the *D. melanogaster* nervous system (Figures 5A and 5B), including a breakdown by axon and dendrite connections across the brain (Figures 5C, 5D, and 6B). We also explored potential correlations between neuron-level morphological features and transmitter predictions (Figures 5E, 5F, and S3), alongside variations in transmitter usage across sensory systems within the fly brain (Figure 6A).

We calculated neuron-level transmitter predictions for all 24,666 well-reconstructed neurons in the HemiBrain dataset (Data S3), all 49,985 central brain and 86,942 optic lobe FAFB-FlyWire neurons (Data S4), and 23,503 ventral nerve cord neurons from the MaleVNC dataset. In the central brain, the largest fraction of neurons were predicted to be cholinergic with smaller fractions for glutamatergic and GABAergic neurons (Figure 6A). Single-cell RNA sequencing has suggested a breakdown of 44%–45% cholinergic, 14%–15% glutamatergic, and 10%–15% GABAergic neurons^21^^,^^22^ in the central brain. Dopaminergic neurons had distinctive features, such as higher mitochondrial density (Figure S3C), a diverse set of input neurons but fewer downstream targets (Figure S3I), primarily in deeper sensory layers (Figure 6C). They are encountered after Kenyon cells in the olfactory system (often thought of as a conditioned stimulus) but before the mushroom body in the gustatory system (typically an unconditioned stimulus). These observations suggest that dopaminergic neurons are highly active and sample widely from more superficial brain layers to provide teaching signals to select target neurons of a deeper sensory layer.

One interesting new insight from large-scale neuron-level transmitter predictions concerns the fan-shaped body. This central brain structure computes navigational variables^75^^,^^76^ and is built as a matrix with ∼9 rows and ∼10 columns.^77^ Our predictions show that row-wise tangential input is overwhelmingly glutamatergic (∼84%), with no inhibitory local neurons in the structure. There are some dopaminergic^78^ but very few GABAergic or cholinergic row-wise inputs. In contrast, acetylcholine was predicted for almost all column-wise input types (∼87%), intrinsic neurons (∼88%), and output types (∼96%); the small number of non-cholinergic neurons are likely mispredictions (see STAR Methods). These findings can be compared to the elegant layout of transmitter expression in the mushroom body,^79^ a discovery that has accelerated research in this associative memory structure in recent years.

**Figure S4:**
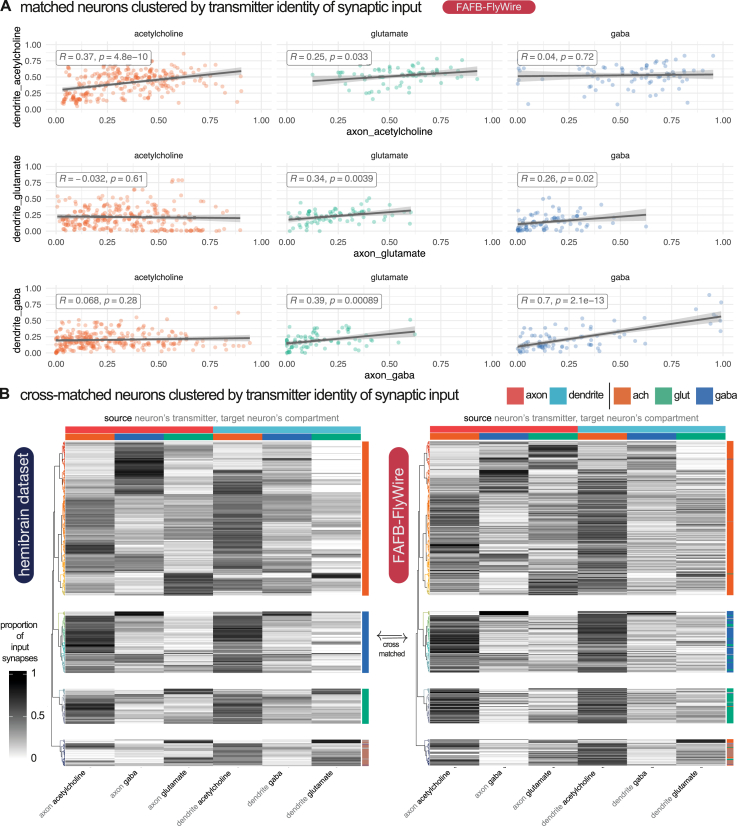
Diversity in targeting by transmitter class and compartment, related to Figure 6 (A) Correlations between opposing input transmitter types by compartment. Plots faceted by the source (upstream) neurons’ neuron-level transmitter prediction (axis values). Colored by the target (downstream) neurons’ neuron-level transmitter prediction. The X axis shows the proportion of a neuron’s input accounted for by the input type on the axis label. Each dot is one neuron. For calculating the R^2^ and *p*-values, neurons for which a proportion on either the X or Y axis fell below 0.1 or above 0.9 were excluded, to remove outlier cases with a very strong input preference. (B) Equivalent 451 neuronal cell types from the FAFB-FlyWire and HemiBrain datasets clustered by input type. Only neurons for which at least 50% of inputs came from well-reconstructed and predicted neurons in our 88,115 FAFB-FlyWire neurons or 11,277 HemiBrain neurons were used. For each source neuron to target neuron connection, we used the identity (neuron-level transmitter prediction), location (neuronal compartment) and normalized connection weight (number of synaptic contacts made on that compartment / total number of synaptic inputs to the target neuron). We calculated cell type averages, and separated target cell types by their transmitter prediction and then clustered within each grouping. Heatmaps show the proportion of synaptic input onto the axon (upper horizontal color bar, red) and dendrite (blue), separated by the neuron-level transmitter prediction for each input (lower horizontal color bar). Each row is a separate neuronal cell type (see Figure S4C for names). Cell types are grouped by a hierarchical clustering within their neuron-level transmitter prediction class (vertical color bar: acetylcholine, glutamate or GABA) employing Ward’s clustering criterion. This clustering was performed in the HemiBrain dataset and applied to the FAFB-FlyWire dataset. Dendrogram (left) colors show a split into 30 groups. The same dendrogam is used in both heatmaps. Cosine similarity, z = 0.892, *p*-value < 0.0001, 100,000 row shuffles. A subset of total central brain neurons that had been skeletonized (see STAR Methods) were used for these analysis (FAFB-FlyWire: 88,115, HemiBrain: 11,277).

The identity of the presynaptic and postsynaptic neuronal compartments is another factor, in addition to transmitter usage, that determines the effect of a synaptic connection.^80^ Insect neurons frequently possess arbors with a mix of input and output synapses, but morphological features can resolve axons and dendrites in most cases^19^^,^^73^^,^^74^^,^^81^^,^^82^^,^^83^ (Figure 5C). Axons and dendrites can synaptically connect with either being the source or the target (Figures 5C and 5B). We “split”^73^ thousands of neurons into separate axonal and dendritic compartments (see STAR Methods). Although most presynapses are on the axon (FAFB-FlyWire: median 76%, SD, 21%; hemibrain: median 70%, SD, 21%), neurons had a large proportion of their output sites on their dendrites (FAFB-FlyWire: median 23%, SD, 21%; hemibrain: median 30%, SD, 21%). We found that while the majority of the synaptic budget is spent on axo-dendritic connections (FAFB-FlyWire: 55%; hemibrain: 48%), a large fraction is spent on axo-axonic connections (FAFB-FlyWire: 22%; HemiBrain: 20%) and a similar amount on dendro-dendritic connections (FAFB-FlyWire: 19%; hemibrain: 21%). These figures are comparable to those recently reported for the *D. melanogaster* larva^19^ (axo-dendritic, 54%; axo-axonic, 36%; dendro-dendritic, 8%; dendro-axonic, 3%). Neurons tended to receive strong input on their axons from just one of acetylcholine, glutamate, or GABA (Figure S4A), in patterns that were consistent between the two datasets (Figure S4B, cosine similarity > 0.9). Axons may therefore be particularly selective in their inputs, particularly inhibitory inputs (Figure S3E).

### Comparing putative inhibitory and excitatory neurons

Putative excitatory neurons predominate in the brain (Figure 5A). The principal excitatory transmitter is acetylcholine,^84^^,^^85^ and the main inhibitory transmitter is GABA.^86^^,^^87^^,^^88^ Glutamate can act in either an inhibitory^89^^,^^90^^,^^91^^,^^92^ or excitatory capacity.^93^^,^^94^^,^^95^ Analyzing the FAFB-FlyWire dataset using a probabilistic layer assignment model,^18^^,^^74^ we observed shifts in the proportions of transmitter use across sensory systems. For example, there is a greater proportion of GABAergic neurons in the early olfactory system, with glutamatergic neurons rising later. This switch also suggests that neurons in deeper layers are likely to be inhibited by glutamate because GABA is scarce.

Cholinergic (mean 0.21, SD, 0.75) and glutamatergic (mean 0.07, SD, 0.57) neurons tended to target higher-layer neurons (Figure 6B) i.e., predominantly feedforward connectivity; GABAergic neurons had no such bias (mean −0.02, SD, 0.61). On average, GABAergic neurons were more “local” in Euclidean space than cholinergic neurons (Figure 5E upper); they were also smaller by cable length (Figure S3A) and less polarized (Figure 5E lower). However, on average GABAergic neurons had more input neurons and downstream targets (Figure S3I). These connections may have a stronger effect than their cholinergic counterparts for two reasons; first, they show greater synaptic count (Figure S3F), and second, because they are located slightly closer to the target neuron’s primary branch point (Figure S3H), which could enable more powerful inhibition.^96^ Additionally, GABAergic neurons had higher mitochondrial density than cholinergic or glutamatergic neurons (Figure S3C), which could indicate a higher level of energy use and neuronal activity. Lastly, GABAergic neurons received more excitatory than inhibitory drive onto their dendrites compared with cholinergic neurons (Figure S3E). Instead, cholinergic neurons often have large inhibitory inputs onto their axons (median, −0.16). Together, this could mean that GABAergic neurons are more active and integrate a wider array of inputs to inhibit a wider array of downstream neurons than cholinergic neurons; both their axons and dendrites make outputs but these are mainly local, perhaps inhibiting many competing elements in a local circuit.^73^^,^^87^^,^^97^^,^^98^ In particular, they may gate the output of cholinergic axons. On all these metrics, glutamatergic neurons lie between GABA and acetylcholine.

### Example transmitter-dependent circuit hypotheses

Our brain-wide transmitter predictions now enable many testable circuit hypotheses. We present three examples that also illustrate how we think about potential confounds.

#### Ocellar righting circuit

In a vignette on ocellar circuitry by Dorkenwald et al.,^18^ our FAFB-FlyWire predictions strongly predicted that 12 OCG01 neurons (with extremely similar axonal morphologies) fall into three glutamatergic and three cholinergic cell types (Figure 6C). A hypothesis emerged: these neurons form pairs, each comprising one inhibitory and one excitatory neuron. For instance, OCG01b (glutamate) and OCG01f (acetylcholine) may collaborate to induce a righting reflex in response to sky-light cues during a roll. The hypothesis was strengthened by the internal control that having two FAFB hemispheres provides; the HemiBrain dataset, in which these neurons are heavily truncated, yielded misleading and likely false predictions (all glutamate).

#### Leg extension circuit

Cheong et al.^99^ uncovered an inhibitory majority in local circuits controlling leg movement. The GABAergic interneurons IN19A were identified as key regulators, reciprocally inhibiting each other to prevent inappropriate co-contraction of opponent leg muscles. Upstream neurons were predicted cholinergic,^5^ creating a circuit architecture facilitating leg extension; a neuron such as IN21A004 could promote leg extension by inhibiting a flexor and disinhibiting a downstream extensor. In this case, it was initially unclear whether the 6 IN20A projection neurons (one per leg) were cholinergic or glutamatergic since 3/6 were predicted glutamatergic. However, all derive from the same hemilineage, which was majority predicted cholinergic. Since Lacin’s law is strongly followed in the ventral nerve cord,^31^^,^^65^ all six IN20A neurons are likely cholinergic.

#### Simultaneous excitatory and inhibitory control

The cholinergic, bilateral mushroom body output neuron, MBON26, is known to cause turning upon optogenetic activation^100^ and innervates mushroom body compartments involved in innate olfactory aversion and appetitive learning.^79^ It directly connects to the downstream turn-control descending neuron, DNa03, and indirectly connects via a predicted glutamatergic local neuron, LAL051 (Figure 6E). If glutamate were purely inhibitory, we might expect unilateral MBON26 activation to result in both ipsi- and contralateral DNa03 inhibition, with slightly more ipsi-DNa03 activity and therefore an ipsilateral turn. If glutamate were purely excitatory, we might expect unilateral MBON26 activation to result in both ipsi- and contralateral DNa03 activation, with slightly more contra-DNa03 activity and therefore a contralateral turn. If glutamate excited DNa03 (via AMPA, kainate, or NMDA receptors, e.g., Li et al.^101^) and inhibited MBON26 (e.g., via *GluClAlpha*), we might expect unilateral MBON26 activation to result in ipsilateral DNa03 inhibition and contralateral DNa03 activation, and so a stronger contralateral turn command. Therefore, combining both circuit structure and transmitter predictions, we suspect that glutamate both excites and inhibits in this circuit to steer the fly away from an aversive olfactory stimulus.

### The distribution of neurotransmitter predictions within developmental units

The nervous system may already naturally group neurons by their transmitter expression because of their development as hemilineages:^31^ these are groups of ∼100 neurons that have developed together in a discrete bundle, the hemilineage tract (see STAR Methods for assignment detail). To assess Lacin’s law in the brain, we examined the neuron-level transmitter predictions of all neurons in the 183 secondary (larval-born) hemilineages per brain hemisphere in the FlyWire dataset^50^ (Data S5 and Data S6).

**Figure 7:**
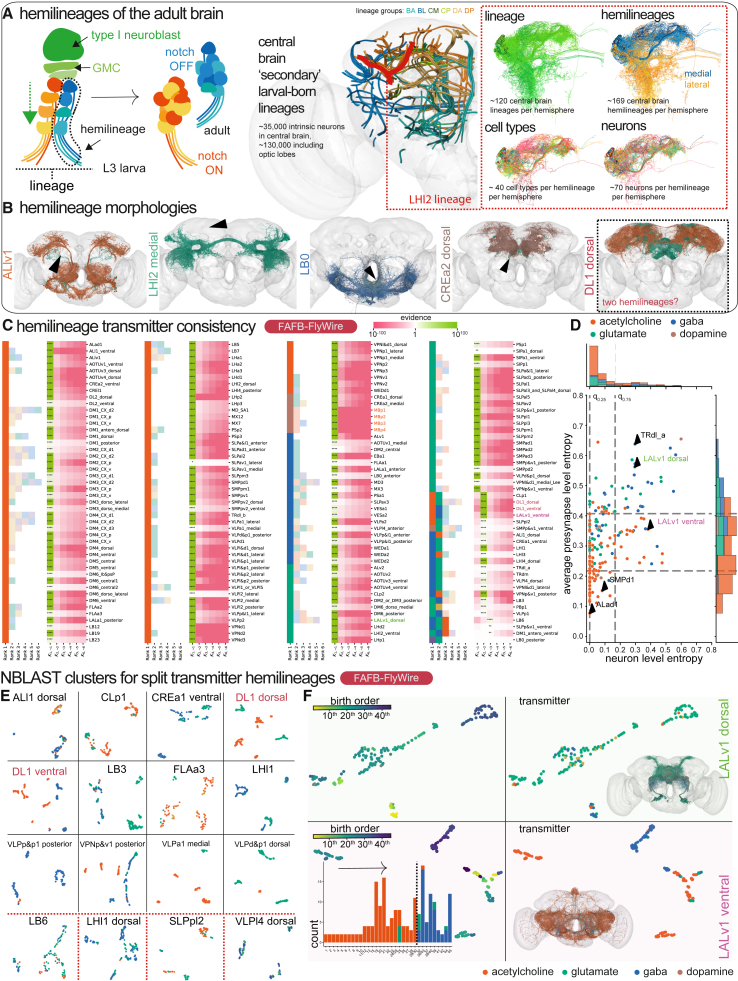
Transmitter usage across all hemilineages in the central fly brain (A) Left, the progression of a type I neuroblast from third-instar larva (L3) into the adult ganglion mother cell (GMC). Right, a breakdown of a single secondary lineage, “LHl2” into its constituent hemilineages (see STAR Methods). (B) Example of homologous FAFB-FlyWire hemilineages on both sides of the brain, colored by neuron-level transmitter prediction. Black arrows point to one stray member of each hemilineage with a different neuron-level transmitter prediction, which is likely a first-born neuron with distinct morphology^31^^,^^102^ (Data S5). The dashed box indicates a hemilineage with potential split transmitter expression. (C) Bayes factor analysis of hemilineage consistency. For each hemilineage (row), the right set of columns corresponds to the likelihood ratio of the hemilineage expressing that number of transmitters versus the likelihood of any other number ($\lambda =16;{\tilde{c}}_{\exp}=0.67$). Evidence strength: substantial ^∗^*K* ⩾ 10^1/2^; good ^∗∗^*K* ⩾ 10^1^; strong ^∗∗∗^*K* ⩾ 10^3/2^; decisive ^∗∗∗∗^*K* ⩾ 10^2^.^103^ The left set of columns indicates the frequency ranked transmitter predictions within the hemilineage, with ranks greater than the maximum likely number of transmitters shaded lighter. “LB0 posterior” did not have substantial evidence for any particular number of transmitters. (D) Neuron level entropy (*H*(*N*_*h*_)) versus average synapse level entropy (*H*(*S*_*h*_)) for all predicted hemilineages with more than 10 neurons and more than 30 presynapses per neuron. Dashed lines indicate 25% and 75% percentiles: *q*_25_(*H*(*N*_*h*_)) = 0.00, *q*_25_(*H*(*S*_*h*_)) = 0.22, *q*_75_(*H*(*N*_*h*_)) = 0.17, and *q*_75_(*H*(*S*_*h*_)) = 0.41.Typeequationhere. (E) NBLAST UMAP plots of selected hemilineages that exhibit some degree of predicted split transmitter usage. UMAPs are based on NBLAST morphological similarity scores between all possible pairs of neurons in each hemilineage. Points represent neurons, colored by neuron-level transmitter prediction. Black solid lines bound examples with a morphology-transmitter split, red dashed lines bound examples with no such clear divide. Red text label examples for which data annotation issues may explain split usage. (F) “LALv1” has two hemilineages: dorsal (developmentally defined by Notch-ON) and ventral (Notch-OFF). NBLAST UMAPs for the two “LALv1” hemilineages (rows): dorsal (developmentally defined by Notch-ON) and ventral (Notch-OFF), colored by birth order (left) and neuron-level transmitter prediction (right). Bottom left, histogram of neuron-level transmitter prediction by birth order.^102^ See also Figure S5 and Data S6.

**Figure S5:**
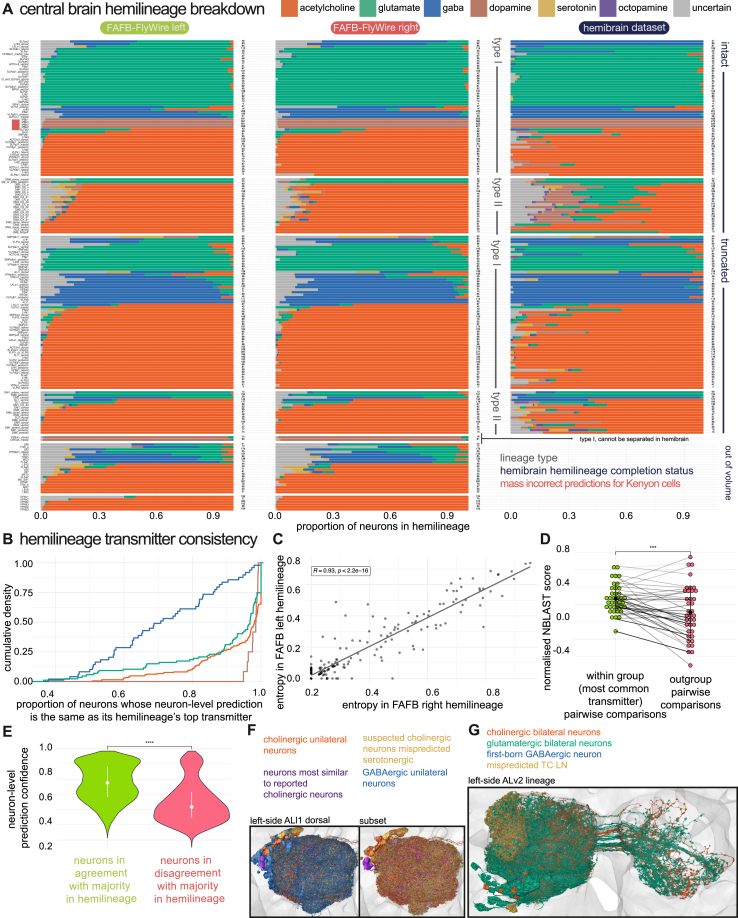
The distribution of neuron-level transmitter predictions within secondary hemilineages, related to Figure 7 (A) Consistency of neuron-level transmitter predictions within selected hemilineages in the central adult *D. melanogaster* brain. Bar plots show the proportion of neurons in each hemilineage predicted to express each of our six transmitters. Data is shown for neurons of the left (left) and right (middle) hemispheres of the FAFB-FlyWire dataset, as well as both hemispheres of the HemiBrain dataset (right). Note that the HemiBrain dataset is only a partial brain, many brain neurons have large missing portions or do not exist in this dataset. Hemilineage names are given on the left of the bar plots, and the numbers of neurons per hemilineage are on the right. The red bar highlights lineages of cholinergic Kenyon cells, MBp1-4, which are mispredicted dopaminergic. The plot is faceted first by presence in the HemiBrain dataset (intact, truncated, missing), then by lineage type (Type I and Type II). (B) Empirical cumulative density plot shows how consistent a transmitter within each hemilineage is predicted to be. The Y axis gives the proportion of hemilineages, and the X axis gives the proportion of neurons in those hemilineages that “voted" for the top transmitter (color groups). (C) How the Shannon entropy (base 6) in the neuron-level transmitter predictions for each hemilineage correlate, between the hemilineage copy on the right (X axis) and left (Y-axis) hemispheres of the FAFB-FlyWire dataset. (D) Dot plot shows the mean normalized pairwise NBLAST scores^47^ between neurons expressing the majority transmitter within a hemilineage (for each green dot, each member of pair expresses the main transmitter) and between these neurons and those expressing other transmitters (pink, at least 10 neurons expresses the other transmitter). Dots represent means taken per hemilineage (183). (E) Violin plot shows the distribution of neuron-level transmitter prediction confidences for neurons that are in agreement with their hemilineage’s transmitter use (green, strictly obey Lacin’s law) and those that do not (pink, strictly obey Lacin’s law). (F) Majority unilateral, left-side antennal lobe local neurons of ‘ALlv1 dorsal’. Neurons colored by their neuron-level transmitter prediction except for a minority of predicted serotonergic neurons with the most ventral cel bodies, given in purple. These are the most similar to described Krasavietz positive cholinergic local neurons.^63^^,^^189^ The upper plot shows neurons predicted to transmit acetylcholine with neurons likely mispredicted to transmit serotonin; they have similar primary neurite and soma positions. We suspect they all should be predicted for acetylcholine. Lower, GABAergic predicted neurons have been added in. (G) Majority bilateral, left-side antennal lobe local neurons of ‘ALlv2’. Neuron meshes colored by neuron-level transmitter prediction. Data were compared using Wilcoxon two-sample tests. Significance values: ns: *p* > 0.05; ^∗^: p ≤0.05; ^∗∗^: p ≤ 0.01; ^∗∗∗^: p ≤0.001; ^∗∗∗∗^: p ≤ 0.0001.

We asked how likely it is to observe a given prediction of transmitters in a hemilineage under some error rate given by the confusion matrix on the test set, if Lacin’s law is obeyed. We then compared this likelihood to the alternative hypothesis that a hemilineage consists of neurons with more than one transmitter. We calculated the Bayes factor $K_{2,1}=\frac{p\left(\hat{y}|m=2\right)}{p\left(\hat{y}|m=1\right)}$ and $K_{3,2}=\frac{p\left(\hat{y}|m=3\right)}{p\left(\hat{y}|m=2\right)}$ for our selected hemilineages from synapse-level transmitter predictions: i.e., the likelihood ratio of the observed model predictions given that a hemilineage expresses two rather than one transmitter or three rather than two transmitters, respectively (see STAR Methods). Maximal one-versus-rest Bayes factors ($K_{m,\neg m}$) summarized our predictions of the number and set of transmitters for each hemilineage (Figure 7C). We found only 19 of 183 hemilineages with evidence of expressing two transmitters (*n* = 19 decisive) and 3 hemilineages with evidence of expressing three fast-acting transmitters (*n* = 1 decisive, *n* = 2 good). These hemilineages are flagged in Figure 7C. However, some of these hemilineages (∼12) such as “TRdla” and “LALv1 dorsal” showed high synaptic entropy *H*(*S*_*h*_) (see STAR Methods, Figure 7D), indicating that individual neurons within the hemilineage contain substantial multimodal transmitter predictions (Figure 7C). As such, multimodality at the neuron level is at least partially explained by uncertain or inhomogeneous predictions between individual synapses within a neuron. This is in contrast to hemilineage “LALv1 ventral,” which has a synaptic entropy within the 75% percentile, and for which a large Bayes factor *K*_2,1_ value directly stems from neuron-level segregation of the predicted transmitters within the hemilineage. We predicted the remaining 163 hemilineages to express a single transmitter (*n* = 161 decisive, *n* = 1 good, *n* = 1 substantial). Results for 154 HemiBrain hemilineages were very similar (Figure S5A, Data S5).

Therefore, 88% of our hemilineages’ predictions were strongly biased toward a singular transmitter identity (Figures 7B, 7C, and S5A). The entropy of neuron-level transmitter predictions for each hemilineage was correlated strongly between left and right hemispheres (Figure S5C), suggesting that the observed variation is biological in origin rather than data quality related. In some cases, only a few neurons deviated from the majority prediction (Figure 7B, black arrows). We suspect that these neurons are the “first-born” neurons of the hemilineage, which often have a divergent morphology from the rest of the hemilineage^31^^,^^102^; they may also be divergent in their transmitter usage.

Other prominently split hemilineages also demonstrated morphology-correlated shifts in transmitter expression (Figures 7C, S5A, and S5D). This suggests discrete switches in expression during development, presumably at stereotyped developmental time points. For instance, LALv1 ventral (Figure 7F) revealed a discernible switch in transmitter expression accompanied by morphological differences. Because a recent analysis delineated the birth order of LALv1 neurons,^102^ we were able to map this transmission-morphotype switch into its developmental sequence (Figure 7F, lower). In a few cases (Figure 7E, lower), split expression occurred without overt morphological differences, raising questions about sporadic switches and potential confounds. Often, glutamatergic and GABAergic neurons are mixed—our network’s most common confusion (Figure 2A). Notably, potentially as much as 3% of the brain expresses mRNA for machinery related to both,^21^ and at least a few may transmit both.^104^ Comparing ∼6 GABA-glutamate mixed hemilineages in FlyWire to HemiBrain indicates that perhaps they more uniformly express GABA (Figure S5A). In two cases (the “DL1” hemilineages), we think that the striking morphology-transmitter correlated splits reveal a hemilineage-based division in a lineage-associated tract that was otherwise hard for human annotators to make, i.e., an issue of data annotation.

Challenges surfaced with the “ALl1 dorsal” and “ALv2” hemilineages that produce antennal lobe local neurons, a morphologically variable and diverse class of neuron.^105^ They seem to break Dale’s law^104^^,^^106^ and Lacin’s law (Figure 7C), with similar morphology types predicted to express different transmitters (Figures S5F and S5G). Despite these challenges, we could align our results with existing literature (see STAR Methods). Surprisingly, 18%–27% of antennal lobe local neurons may be cholinergic, suggesting that lateral excitation is a more prominent feature of antennal lobe processing than previously thought.

## Discussion

### Using high-level annotations to learn low-level features

In evaluating our predictions as broadly as we could, we have found them to be correct for 91% of 624 FAFB-FlyWire cell types and 91% of 524 HemiBrain cell types (Data S7). This result likely depended on three properties of the data that we selected. (1) For both training and inference we used a specific sub-cellular domain (the synapse), likely to contain image features related to our molecules of interest. (2) We aggregated these features on a per-cell basis; this was crucial for linking ground-truth labels and input data and also for improved prediction accuracy. (3) By using cross-modal matching of cell types between EM and light-level neuronal morphology data, we were able to build an expansive ground-truth dataset from molecular information external to the EM data. We anticipate broad applications for this general approach in the biological sciences. Our methodology could be repurposed to identify differences in sub-cellular structures^107^ associated with discrete cell types in a range of neuronal and non-neuronal tissues.

### Machine learning reveals key molecular descriptors of neuronal function

*D. melanogaster* represented a hard case for transmitter prediction because humans cannot tell fast-acting transmitters apart in insects. Our classifier accurately predicts transmitter identity from local 3D EM volumes (Figure 2). We find that the overall accuracy of our synapse-level transmitter predictions were slightly more performant in FAFB than in HemiBrain; in particular, HemiBrain is more likely to confuse glutamate for GABA (Figure 2A). This might be because of different sample preparation and staining protocols used for those datasets, our use of only manually annotated presynapses for FAFB ground truth, and/or the higher lateral resolution of FAFB (4 nm vs. 8 nm).

Given that the relationship between synaptic phenotype and transmitter identity is not understood in *D. melanogaster*, we built an explainable AI method that created counterfactual synthetic images to investigate class differences^71^ (Figures 3A and 3B). This enabled us to manually identify at least one feature difference between each pair of fast-acting transmitters (Figure 3C). However, a simple logistic regression classifier trained on three of those features was not able to discriminate between fast-acting transmitters at the same level as the classifier (*n* = 219, 80%/20% randomized training/testing, balanced accuracy 0.52). This indicates that the features we identified are not exhaustive.

We expect our pipeline to be transferable to other connectomic datasets from diverse species. Analogous work detecting excitatory versus inhibitory synapses in the *Ciona intestinalis* larva has added signs for 49 neurons.^108^ In vertebrate EM datasets, human annotators can see differences in the vesicles for excitatory and inhibitory synapses.^16^^,^^35^^,^^36^^,^^37^^,^^109^^,^^110^ Symmetric synapses (usually inhibitory) have already been disambiguated from asymmetric ones (usually excitatory) automatically and at scale.^38^ Using our methodology, performant and more specific transmitter predictions are likely to be achievable in both vertebrate and invertebrate datasets.

### Sign labeling across a whole-insect central nervous system

Dale’s law, despite its exceptions, serves as a valuable framework for understanding neural action in the face of incomplete data. We needed to use it to accumulate synapse-level transmitter predictions into singular neuron-level transmitter predictions. Extending our conceptual framework, we found that Lacin’s law^31^ is a useful principle that holds in 88% of hemilineages (Figure 7C). We posit that the major exceptions emerge as correlated, discrete switches in both transmitter use and gross morphotype (Figure 7F). Lacin’s law serves the network anatomist in three main ways: supporting prediction validity (Figures S5A and 2D), flagging potential errors when a neuron-level transmitter prediction deviates from the hemilineage majority, and allowing for preliminary transmitter labels to be applied to whole hemilineages in new insect datasets.^111^^,^^112^

The functional role of a synaptic connection depends on upstream transmitters and downstream receptors. Simulating connectomes directly^113^ at a whole nervous system scale may be possible with our method (e.g., Shiu et al.^114^), but incorporating information on postsynaptic receptor expression can make brain models more accurate.^113^ Most importantly, while acetylcholine excites and GABA inhibits, glutamate can perform either function in the fly. In the *D. melanogaster* central brain, most reported examples for glutamate are of inhibition via *GluClAlpha* channels.^89^^,^^90^^,^^91^^,^^92^^,^^115^ The nature of this inhibition could be different, e.g., possibly subtractive^91^ rather than divisive.^86^^,^^87^^,^^88^^,^^115^ Indeed, glutamate may be used more for specific long-range feedforward inhibition and GABA for local divisive inhibition (Figure 6A). However, central excitatory glutamatergic transmission has also been reported.^93^^,^^95^^,^^116^^,^^117^ At the neuromuscular junction, glutamate primarily excites.^94^ We calculated from single-cell RNA sequencing^118^ that ∼80% of neurons in the central brain express RNA transcripts for *GluClAlpha* as well as at least one excitatory ionotropic glutamate receptor. The sign of a glutamatergic connection may depend on the sub-localization and ratio of receptors at recipient sites.^119^

Beyond fast-acting transmitters, other factors—such as cellular compartments^80^—influence the strengths and signs of neuron-neuron connections. Notably, a large fraction of the synaptic budget is spent on axo-axonic connections (FAFB-FlyWire, 22%; HemiBrain, 20%). Putative inhibitory connections accounted for two-thirds of the axo-axonic sub-budget (Figure 5D) and axons often receive a skewed excitatory:inhibitory connection ratio (Figure 5F). This suggests that the nervous system may employ different circuit logic around axons compared with dendrites.

### Limitations of the study

We can think of this study’s limitations in two major ways: misprediction (perhaps largely because of co- or alternative transmission, see STAR Methods) by our classifier and other limitations on sign labeling from transmitter identity alone. Outstanding problems that must be solved before we have highly accurate connection signs minimally include: (1) the correction of transmitter mispredictions, (2) the prediction of other transmitters, notably histamine, tyramine, glycine, nitric oxide, and ∼53 different neuropeptides, (3) the annotation of monoaminergic co-transmission, (4) the annotation of peptidergic (co-)transmission, (5) the annotation of postsynaptic receptor expression, and (6) the annotation of gap junctions. For (1), community open science annotation projects projects such as FlyWire via Codex^120^ and Virtual Fly Brain^121^ will be valuable. In particular, our least performant predictions were for serotonin. Notably, ∼20 known, large, neuropeptidergic neurons are predicted for serotonin. This may be because we included peptidergic, serotonergic neurons in our ground truth.^122^^,^^123^ Answers to (2), (3), and (4) could be achieved by linking morphological and transcriptomic cell types^49^ to build more ground truth. One could extend our method to further transmitters and co-transmission combinations as additional training data becomes available (we have shared our literature review of cell type level co-transmission, Data S7). In addition, the detection of individual vesicles, especially dense core vesicles at synapses, or somata could tell us which neurons express neuropeptides and peptide hormones. For problems (5) and (6), linked transcriptomes alone are unlikely either to solve the issue or to provide suitable ground truth for their detection in EM. One path forward would be to assemble ground-truth data with super-resolution light-level microscopy to observe endogenous protein sub-localization^119^^,^^124^^,^^125^ or with higher-resolution EM to identify key molecules at identified synapses in dense biological samples.^126^ As we showed here with transmitter usage, the identification of some hundreds of pairs of connected neurons may provide sufficient ground truth for a machine-learning solution to predict the remainder across whole nervous systems.

## STAR★Methods

### Key resources table

**Table:** 

REAGENT or RESOURCE	SOURCE	IDENTIFIER
**Deposited data**		

FAFB ssTEM image data	Zheng et al.^6^	https://temca2data.org/
FAFB-FlyWire	Dorkenwald et al.^120^	https://ngl.flywire.ai/
FAFB-FlyWire connectome	Dorkenwald et al.,^17^ Matsliah et al.^127^	https://codex.flywire.ai/
FAFB-FlyWire annotations	Schlegel et al.^128^	https://doi.org/10.5281/zenodo.8077334
HemiBrain FIBSEM image data	Scheffer et al.^129^	https://neuprint.janelia.org/?dataset=hemibrain:v1.2.1
HemiBrain connectome	Scheffer et al.^129^	https://neuprint.janelia.org/?dataset=hemibrain:v1.2.1
FAFB-FlyWire synapse-level predictions	This paper	synister fw mat571 t11 synapses.feather
HemiBrain synapse-level predictions	This paper	hemibrain-v1.2-tbar-neurotransmitters.feather.bz2
FAFB-FlyWire neuron-level predictions	This paper	https://codex.flywire.ai
HemiBrain neuron-level predictions	This paper	https://neuprint.janelia.org/?dataset=hemibrain:v1.2.1
All HemiBrain and FlyWire synapse and neuron-level predictions	This paper	https://doi.org/10.5281/zenodo.10593546

**Software and algorithms**		

R	R Core Team^130^	RRID SCR 001905
*knitr* (R)	Xie^131^	RRID SCR 018533
*ggplot2* (R)	Wickham^132^	RRID SCR 014601
*tidyverse* (R)	Wickham et al.^98^	RRID SCR 019186
*rgl* (R)	Wickham et al.^98^	https://cran.r-project.org/web/packages/rgl/index.html
*natverse* (R)	Bates et al.^133^	https://natverse.org/natverse/
*neuprintr* (R)	Bates et al.^133^	https://natverse.org/neuprintr/
*hemibrainr* (R)	Schlegel et al.^123^	https://github.com/flyconnectome/hemibrainr
*catmaid* (R)	Bates et al.^133^	https://natverse.org/rcatmaid/
*fafbseg* (R)	Schlegel et al.^128^	https://natverse.org/fafbseg
*nat.jrcbrains* (R)	Bates et al.^133^	https://natverse.org/nat.jrcbrains/
*nat.nblast* (R)	Costa et al.^121^	https://natverse.org/nat.nblast/
python	Van Rossum and Drake^134^	RRID SCR 008394
*navis* (python)	Schlegel et al.^51^	https://github.com/navis-org/navis
*navis-flybrains* (python)	Schlegel et al.^51^	https://github.com/navis-org/navis-flybrains
*pymaid* (python)	Schlegel et al.^51^	https://github.com/navis-org/pymaid
*fafbseg-py* (python)	Schlegel et al.^51^	https://github.com/navis-org/fafbseg-py
*skeletor* (python)	Schlegel et al.^74^	https://github.com/navis-org/skeletor
*neuprint-python* (python)	Plaza et al.^135^	https://github.com/connectome-neuprint/neuprint-python
*synister, transmitter prediction* (python)	This paper	https://doi.org/10.5281/zenodo.10593546

### Resource availability

#### Lead contact

Further information and requests for resources and reagents should be directed to and will be fulfilled by the lead contact, Jan Funke (funkej@janelia.hhmi.org).

#### Materials availability

This study did not generate new unique reagents.

#### Data and code availability

• All of our data is also available through Zenodo, https://doi.org/10.5281/zenodo.10593546, and will be available through the Virtual Fly Brain project.^121^ Our synapse-level transmitter prediction and neuron-level transmitter predictions are also hosted by extant connectome annotation and browsing services for the HemiBrain () and FAFB-FlyWire (https://codex.flywire.ai/) datasets.^120^^,^^135^ As supplemental data for this paper, we have provided: (1) the studies we have used to generate our ground-truth data (Data S1), (2) identifiers for the neurons we used for our ground truth data (Data S2), (3) our neuron-level transmitter predictions for each complete neuron in the HemiBrain dataset, hemibrain:v1.2.1 (Data S3), and the FAFB-FlyWire dataset, 630 materialization (used in this paper but with links to the newer 783 materialization)(Data S4), (5) images of all discerned brain hemilineages for FlyWire^50^ and HemiBrain, with neurons colored by their predicted transmitter (Data S5), as well as (6) summary data on hemilineage transmitter usage (Data S6), and (7) an transmitter expression summary by brain cell type (Data S7). We have provided a section in these Methods of advice to users on how to use our data.
• Our transmitter classification network and associated training and prediction code are available in our Synister repository (https://github.com/funkelab/synister) which also contains instructions on how to access the FAFB-FlyWire and HemiBrain predictions, and through Zenodo, https://doi.org/10.5281/zenodo.10593546.
• Any additional information required to reanalyze the data reported in this paper is available from the lead contact upon request.

### Experimental model and study participant details

The HemiBrain connectome^51^ is a partial female fly brain that has been semi-automatically reconstructed using flood-filling networks^8^ from data acquired by focused ion-beam milling scanning EM (FIBSEM).^136^ Presynapses (i.e., sites with T-bars) and postsynapses were identified completely automatically. The data can be accessed via the NeuPrint connectome analysis service.^135^ Automatically detected mitochondria counts in the HemiBrain dataset were pulled from neuPrint^135^ (https://connectome-neuprint.github.io/neuprint-python/docs/mitocriteria.html).

The FAFB ssTEM image data comprises an entire female fly brain. Two auto-segmentations of the data exist,^17^^,^^137^ we used the FlyWire segmentation and automatically detected synapses^12^ for our biological analyses. However, to build our ground truth data we used high-fidelity manually reconstructed neurons and synapses that were annotated using CATMAID.^73^ The manually placed FAFB synapses in this study were identified in Catmaid.^6^^,^^73^ Catmaid is a collaborative environment in which 27 labs have participated to build connectomes for specific circuits. For these annotations, we thank Ruairi Roberts, Fiona Love, Lisa Marin, Amelia Edmondson-Stait, Xincheng Zhao, Jawaid Ali, Johann Schor, Imaan Tamimi, Arian Jamasb, Marisa Dreher, Markus Pleijzier, Robert Turnbull, Nadiya Sharifi, Steven Calle, Andrew Dacks, Konrad Heinz, Kimberly Meechan, Aidan Smith, Najla Masoodpanah, Serene Dhawan, Peter Gibb, Corey Fisher, Claire Peterson, Jason Polsky, Tansy Yang, Katharina Eichler, Joseph Hsu, Irene Varela, Lucia Kmecova, Istvan Taisz, Jacob Ratliff, Kaylynn Coates, Anna Li, Marta Costa, Tyler Paterson, Claire Managan, Adam Heath, Katie Stevens, Jack Mccarty, Nora Forknall, Laurin Bueld, Neha Rampally, Zane Mitrevica, Kelli Fairbanks, Stanley Tran, Shada Alghailani, Quinn Vanderbeck, Lauren Warner, Henrique Ludwig, Jeremy Johnson and Levi Helmick, each of whom has contributed over 1,000 synapses. We in particular thank the Wellcome Trust UK and US Drosophila Connectomics, Jefferis, Janelia Connectome Annotation Team, Bock, Preat, Wilson, Dacks, Hampel and Seeds groups for sharing their published and unpublished work in the Catmaid dataset. Development and administration of the Catmaid tracing environment and analysis tools were funded in part by National Institutes of Health BRAIN Initiative grant 1RF1MH120679-01 to Davi Bock and Gregory Jefferis, with software development effort and administrative support provided by Tom Kazimiers (Kazmos GmbH) and Eric Perlman (Yikes LLC). We thank Marissa Sorek for assistance with community management and Ran Lu, Thomas Macrina, Kisuk Lee, J. Alexander Bae, Shang Mu, Barak Nehoran, Eric Mitchell, Sergiy Popovych, Jongpeng Wu, Zhen Jia, Manuel Castro, Nico Kemnitz, Dodam Ih for alignment and segmentation of the FAFB EM volume and registration to the original FAFB EM dataset. Both datasets are from female flies.

### Method details

#### Detail on assembling ground truth data for *D. melanogaster*

We created a list of 356 neuronal cell types from 21 studies (see Data S1) and identified as many of them as we could in the FAFB-Catmaid and HemiBrain datasets (Figure 1A).^39^^,^^40^^,^^51^^,^^56^^,^^57^^,^^77^^,^^78^^,^^79^^,^^88^^,^^90^^,^^138^^,^^139^^,^^140^^,^^141^^,^^142^^,^^143^^,^^144^^,^^145^^,^^146^^,^^147^^,^^148^^,^^149^^,^^150^^,^^151^^,^^152^^,^^153^^,^^154^^,^^155^^,^^156^^,^^157^^,^^158^ FAFB neurons were found in extant FAFB-Catmaid data (https://neuropil.janelia.org/tracing/fafb) based on the FAFB-Catmaid literature,^6^^,^^82^^,^^96^^,^^97^^,^^129^^,^^142^^,^^157^^,^^159^^,^^160^^,^^161^ by using NBLAST^47^ or by manually reconstructing them in Catmaid from scratch using previously reported methodologies to approximate candidate locations for known cell types based on their hemilineage identity.^6^^,^^73^^,^^97^^,^^162^ The transmitter expression evidence for these cell types in the literature came from detecting RNA expression related to transmitter usage (minority) or immunohistochemistry (majority). For these studies, neurons were picked (transcriptomics) or stained (immunohistochemistry) with guidance from green fluorescent protein expression in a GAL4/split-GAL4 lines.^46^ These genetic driver lines target transgene expression to a small constellation of discriminable cell types, or even individual cell types or neurons, which could be linked to specific homologs in the EM datasets. There are only a small number of our identified connectomic cell types for which we found we knew co-transmission of small-molecule transmitters occurred. Mi15 (acetylcholine, serotonin), Mi9 (glutamate, GABA), Mi4 (GABA, nitric oxide), C3 (GABA, nitric oxide), Mi15 (acetylcholine, dopamine, nitric oxide)^39^ most octopaminergic neurons (octopamine, glutamate)^21^^,^^163^ and a subset of PPL1 neurons (dopamine, serotonin).^78^^,^^150^ We excluded these cell types from our training data, except for the known octopaminergic neurons because 70% of them are thought to co-express glutamate. Because each neuron in our ground truth data must have a single transmitter label, these were given “octopamine” alone. Co-transmission of a fast-acting transmitter and a neuropeptide was more common, e.g., Kenyon cells (at least acetylcholine, sparkly and short neuropeptide F),^21^^,^^25^^,^^55^^,^^164^ the dorsal paired medial (DPM) neuron (GABA, serotonin, sparkly, nplp1, proctolin, orcokinin, eclosion hormone, CG34136)^25^^,^^56^^,^^60^ and most central complex, mushroom body output and mushroom body dopaminergic neurons.^25^^,^^157^ Of these, we excluded Kenyon cells due to their unusual synapse morphology.

For each neuron reported in the literature, we assumed that all associated presynapses obeyed Dale’s law (Figure 1B). The intention was that our FAFB ground truth data would consist entirely of manually placed presynaptic sites, which has the advantage of later training our network with only high-fidelity presynapses. Synapses were annotated at presynaptic sites, defined by T-bars, vesicles, and a thick dark active zone by a synaptic cleft.^165^ We scored each continuous synaptic cleft as a single presynapse regardless of its size or the number of associated T-bars. Note that where we say we used FlyWire data, we looked at transmitter predictions across automatically detected presynapses in FAFB^12^ using the newer FlyWire (https://ngl.flywire.ai/) reconstruction.^17^^,^^18^^,^^50^ This enabled us to work with the full fly brain connectome.

Many neuronal cell types with transmitter expression data had already been linked to reconstructions in the HemiBrain dataset by the reporting authors or.^51^ We matched the remaining unlinked cell types using the morphological match algorithms NBLAST and/or a color MIP search.^47^^,^^48^^,^^50^ We used automatically detected presynapses from identified neurons,^13^ which were more numerous than the manually placed ones we had from Catmaid, but potentially contained more low-quality identifications and multiple detections across the same continuous cleft. Manually placed presynapses were not available in this dataset at scale.

In total, we matched 3,025 FAFB-Catmaid neuronal reconstructions to cell types with a known transmitter. The assembled Catmaid ground truth dataset contained 153,593 acetylcholine presynapses (587 neurons), 7,953 glutamate presynapses (50 neurons), 32,836 GABA presynapses (175 neurons), 9,526 dopamine presynapses (83 neurons), 4,732 serotonin presynapses (5 neurons), and 2,924 octopamine presynapses (6 neurons) (see Data S2). We also matched 5,902 HemiBrain reconstructions to cell types with a known transmitter. It contained 451,033 acetylcholine presynapses (3,094 neurons), 75,239 glutamate presynapses (218 neurons), 80,732 GABA presynapses (242 neurons), 117,054 dopamine presynapses (310 neurons), 70,460 serotonin presynapses (38 neurons), and 46,017 octopamine presynapses (21 neurons).

Due to a relative paucity of linked presynapses when we began this study we did not use our annotations for: allatostatin A (2 neurons in the HemiBrain), corazonin (6), drosulfakinin (6), glycine (9), insulin (23), IPNa (2), *Drosophila* NPF (3), and SIFamide (4). Histamine is also used in the *D. melanogaster* brain, primarily by photoreceptor neurons not captured in our original datasets, and a few ascending neurons. Nitric oxide is used as a co-transmitter,^25^ we did not include it in our dataset. We therefore only carried forward: acetylcholine, glutamate, GABA, dopamine, serotonin and octopamine. At least one of these six transmitters is probably expressed by most neurons in the brain.^21^

We thank Michael Reiser, Vivek Jayaraman, Arthur Zhao, Tatsuo Okubo, Jenny Lu and Kathi Eichler for identifying neuron matches in Catmaid which helped us build our ground truth dataset, Mareike Selcho for helping to confirm our FAFB-FlyWire identifications of octopaminegic neurons and Clare Pilgrim for further cell type to known transmission annotations.

#### Chosen neuronal reconstructions

We calculated neuron-level transmitter predictions for all 24,666 well reconstructed neurons in the HemiBrain dataset (see Data S3), all 49,985 central brain neurons and 86,942 neurons from FAFB-FlyWire (see Data S4) and 23,503 ventral nerve cord neurons from the MANC dataset. Data was pulled from: HemiBrain: https://neuprint.janelia.org/?dataset=hemibrain:v1.2.1, FAFB-FlyWire: https://codex.flywire.ai, MANC: https://neuprint.janelia.org/?dataset=manc:v1.0. All FAFB-FlyWire data is from the 630 materialization, a specific public version of the FlyWire dataset first released with the flagship FAFB-FlyWire dataset preprint.^18^ Our synapse-level transmitter predictions can be associated with these segments and therefore neurons built by the community even if they are further edited (further FAFB-FlyWire dataset releases are planned, i.e., the 783 materialization destined for the publication). Note that the male ventral nerve cord results were generated using our methodology in the MANC dataset (manc:v1.0) but reported on elsewhere.^5^ We included all neurons from the project with the status “Traced” and gave 3638 neurons that had fewer than 100 presynapses the label “uncertain”. Due to the availability of single cell type ground truth, only acetylcholine, glutamate and GABA were predicted in this dataset. Together, these results are part of our data releases and shown in Figure 5A and used in Figure 6.

For other biological analyses (Figures 5D–5F, S3E–S3H, and S4), we analyzed a subset of central brain neurons from HemiBrain and FAFB-FlyWire. We excluded Kenyon cells and neurons because we had very high confidence that these predictions were incorrect. We kept other possible mispredictions. We excluded truncated neurons and neurons with fewer than 100 presynapses. Truncated neurons typically included bilateral neurons in HemiBrain and ascending, descending and first-order sensory neurons in both datasets.

For this purpose, 246,66 neurons in HemiBrain were filtered down to 11,277 by excluding neurons without the HemiBrain project status label “Traced", neurons with less than 100 presynapses and removing truncated neurons with a large part of their arbor cut from the dataset (label “cropped"), including neurons with the terms: LP, LC, LT, DN and LLP in their cell type label. These neurons were semi-automatically reconstructed by,^51^ and come from neuPrint release hemibrain:v1.2.1.

For the FAFB-FlyWire dataset we used 88,115 intrinsic brain neurons. Neurons were selected as well traced if a human annotator could confirm that they appeared to have a full dendrite, axons, cell body fibre tract and cell body (soma). This pool is small compared with the total 136,927 FAFB-FlyWire neurons because 1) they were selected as well reconstructed in FAFB-FlyWire by the start of 2023 and 2) we only selected neurons that we could ‘skeletonize’ and ‘split’ into separable axons and dendrites^73^^,^^133^ for our biological analyses and 3) we only chose neurons whose arbors were contained within the central brain.

Neurons are unmatched between datasets in our analyses e.g., (Figures 5 and S3) unless noted otherwise (e.g., Figure 4), i.e., the corpus of neuronal cell types from each dataset is partially overlapping but distinct, cell type content may account for some of the FAFB–HemiBrain differences. The full pool of HemiBrain neurons was not cross-matched to FAFB-FlyWire neurons at the time of writing, only 2626 neuronal cell types.

We thank the flywire.ai community for allowing us to use their semi-automatic FAFB-FlyWire neuronal reconstructions, which took over 1,366,543 edits from human annotators to build from automatically reconstructed segments.^17^ We also want to specifically thank those human annotators that contributed to a smaller pool of 27,706 neurons, built between 2019 and 2022, on which our results were initially piloted and preprinted. These neurons were built by 1,366,543 edits of automatically reconstructed segments,^17^ from 100 human annotators Those persons contributing more than 1,000 edits were: Doug Bland, Austin T Burke, Yijie Yin, Laia Serratosa Capdevila, Kyle Patrick Willie, Arti Yadav, Ryan Willie, Nash Hadjerol, Zairene Lenizo, Griffin Badalemente, J. Anthony Ocho, Shirleyjoy Serona, Dharini Sapkal, Anjali Pandey, Ben Silverman, Varun Sane, Zeba Vohra, regine salem, Mendell Lopez, J. Dolorosa, Imaan Tamimi, Chitra Nair, Dhwani Patel, Joshua Bañez, Márcia Santos, Katharina Eichler, Shaina Mae Monungolh, Dustin Garner, Jay Gager, Joseph Hsu, Mark Larson, Bhargavi Parmar, Rey Adrian Candilada, Dhara Kakadiya, Alexandre Javier, Itisha Joshi, Michelle Pantujan, Irene Salgarella, James Hebditch, Kaushik Parmar, Darrel Jay Akiatan, Kendrick Joules Vinson, Marina Gkantia, Ariel Dagohoy, remer tancontian, Chan Hyuk Kang, Hane Two, Markus Pleijzier, Emil Kind, Olivia Sato, Yashvi Patel, Miguel Albero, Eva Munnelly, Katie Molloy, Christopher Dunne, Quinn Vanderbeck, Rashmita Rana, Merlin Moore, Lucia Kmecova, Alexis E Santana Cruz, Nadia Seraf, Usb, Claire McKellar, Monika Patel, Mareike Selcho, Greg Jefferis, Steven Calle, Siqi Fang, Arzoo Diwan, Sarah Morejohn, Christa Baker, Brian Reicher, Sangeeta Sisodiya, Tansy Yang, Paul Brooks, Selden, Marlon Blanquart, Hyungjun Choi, Celia D, Sanna Koskela, Joanna Eckhardt, Krzysztof Kruk, Wolf Huetteroth, Alisa Poh, Stefanie Hampel, Wes Murfin, Li Guo, Zhihao Zheng, Szi-chieh Yu, Jones, Farzaan Salman, Amalia Braun, Mark Lloyd Pielago, Nidhi Patel, Ben Gorko, Akanksha Jadia, Fernando J Figueroa Santiago and Urja Verma. We thank Forrest Collman, Casey Schneider-Mizell, Chris Jordan, Derrick Brittain, Akilesh Haligeri for CAVE development and maintenance, and Kai Kuehner, Oluwaseun Ogedengbe, Jay Gager, Will Silversmith, Ryan Morey for Neuroglancer development, tools, and Codex development - these tools made efficient reconstruction possible.

#### Neuron skeletonization and axon-dendrite splits

Some morphology analyses required “skeletonized“ neuronal reconstructions and splitting them into axon and dendrite.^73^ For HemiBrain we retrieved neuronal skeletons from the HemiBrain neuPrint project,^135^ which were generated by an edge collapse method. For FAFB-FlyWire we calculated skeletons using the “wavefront" (rays = 2) algorithm as implemented in the python library “skeletor".^128^ We “skeletonized" or retrieved skeletons for a subset of neurons (HemiBrain: 11,277, FAFB-FlyWire: 88,115)

Separate axons and dendrites^73^^,^^81^^,^^83^ were discerned using a graph theoretic algorithm,^73^ implemented in the R based natverse toolbox.^133^ To improve the results of this algorithm, we removed likely erroneous presynaptic detections on the soma, primary neurite or otherwise outside of the synaptic neuropil, a mean of ∼ 5% per neuron. In general, the soma, cell body fibre tract and linker cable (primary dendrite) do not contain a large number of synaptic contacts. The raw numbers of synapses after filtering can be quite different between the FAFB-FlyWire and HemiBrain datasets (FAFB-FlyWire: median 202, hemibrain: median 386). This method benefited from the O2 High-Performance Compute Cluster, supported by the Research Computing Group, at Harvard Medical School (https://it.hms.harvard.edu/our-services/research-computing).

#### Neuron level transmitter prediction confidence

Assuming Dale’s law holds for all neurons, we labeled each neuron with a single transmitter and provided a confidence score for this assignment. To do this, we first retrieved all presynaptic sites associated with each neuronal reconstruction. We removed potential erroneous presynapses by employing standard thresholds for synapse detection for each dataset (a cleft score above 50 for FAFB-FlyWire auto-detected synapses,^18^ and a confidence score above 0.5 for HemiBrain auto-detected synapses^51^). We only considered neurons with at least 100 presynapses. We further validated our cut-off for automatically-detected FAFB presynapses^12^ by evenly sampling 4,306 presynapses across all six synapse-level transmitter predictions and neuronal compartments (see Figure 5C) and determining whether the auto-detected preynapses were valid by cross-checking with a human annotator. 32% of auto-detected presynapses were erroneous. Our chosen threshold eliminates ∼ 13% of valid presynapses and ∼ 60% of erroneous detections (Figure S1A). Because a higher proportion of auto-detected presynapses on the primary dendrite, cell body fiber tract and soma were erroneous, we retained only axonic and dendritic presynapses. (Figure S1B). Presynapses had also to be no less than 15 μm from a neuron’s cell body, and 0.1 μm from its primary dendrite, to ensure that erroneous detections on non-synaptic cable were not used, this accounted for ∼ 5% of presynaptic connections per neuron. Filtering presynapses produced a different neuron-level transmitter prediction in 4% of neurons (columns ‘top_nt’ (unfiltered after the 50 cleft score threshold) and ‘conf_nt’ (filtered)). Notably, a larger proportion of octopamine predicted presynapses proved erroneous (43%), indicating that the network may be more likely to guess octopamine for dark features of non-synaptic origin (Figure S2F).

To calculate neuron-level transmitter predictions, we determined which synapse-level transmitter prediction was most common for each neuron. If the difference between the top and second highest transmitter was <10%, a neuron’s neuron-level transmitter prediction was designated *uncertain*. To determine a neuron-level transmitter prediction confidence score for each neuron (or neuronal compartment), we computed the average confusion matrix value of each presynapse prediction as given in the row of the winning transmitter. More formally, we assigned a confidence value c(n) to a neuron *n* as:

(Equation 1) $$c\left(n\right)=\frac{1}{\left|S_{n}\right|}\sum_{s\in S_{n}}C_{{\hat{y}}_{n},{\hat{y}}_{s}}\text{,}$$

where $S_{n}$ is the set of presynapses of neuron *n*, ${\hat{y}}_{n}$ the winning transmitter of the entire neuron, ${\hat{y}}_{s}$ the predicted transmitter of synapse *s*, and *C* the confusion matrix as computed on the test dataset. For example, if we determined a FAFB-FlyWire neuron was cholinergic because a majority of its presynapses were predicted to transmit acetylcholine, each presynapse predicted as acetylcholine would contribute a value of 0.95—the proportion of cholinergic ground-truth presynapses correctly determined as cholinergic (Figure 2A)—and any presynapse presumably mispredicted as GABA would contribute a value of 0.02 (the proportion of ground-truth cholinergic presynapses mispredicted as GABAergic). A neuron’s confidence score is the mean of these values over all presynapses. The distribution in these scores across the FAFB-FlyWire and HemiBrain datasets shows that we have most confident predictions in acetylcholine and least confident in serotonin (Figure 2D). Indeed, our serotonin predictions are less reliable, with several suspected mispredictions including missing known serotonergic neurons (Figure S2E).

It should also be noted that each neuron was given a predicted transmitter identity even if in reality it would express none of the six transmitters with which we trained. When the network is applied to data outside of the training distribution, it is often less confident in its erroneous prediction, but not always.

#### Correlating misprediction with reported unlearned transmsison types

In examining our neuron-level transmitter predictions together with reported expression in the literature, we noticed a few issues and errors, which we have investigated to try to establish the major reasons for misprediction.

For HemiBrain neurons we know to use transmitters outside of the six we learned,^51^ we found that glycinergic neurons^143^ were mostly predicted to use acetylcholine (mean confidence 0.66), Allatostatin A^143^ were mostly predicted to use GABA (0.7) (even though it most commonly co-transmits with glutamate^21^), Corazonin^166^ use was predicted as acetylcholine (0.3), Drosulfakinin^167^^,^^168^ use was mostly predicted as octopamine (0.47), insulin-like peptides^169^^,^^170^ use was mostly predicted as acetylcholine or serotonin (0.44), IPNamide^154^ use was mostly predicted as glutamate (0.32), *Drosophila* neuropeptide F^145^ use was mostly predicted as octopamine (0.46) and SIFamide^171^ use was mostly predicted as octopamine (0.43).

Mispredictions in which one of our six learned transmitters is predicted when another should have been, can occur in one dataset but not the other (Figures S2D–S2F). For example, our mispredictions include neurons thought to be dopaminergic being assigned serotonin (FB4L), octopamine (FB4M) or glutamate (PPM1024) in HemiBrain but correctly in FlyWire, and other dopaminergic neurons being mispredicted as glutamatergic (PAM01) in FlyWire but not in HemiBrain, which may happen due to unclear dataset-specific confounds. However, some of the most notable mispredictions occur in both. For example, the largest scale misprediction we caught was our network’s incorrect mass prediction of Kenyon cells as dopaminergic. Immunohistochemistry and RNA sequencing data have shown that Kenyon cells express the machinery necessary for cholinergic transmission.^21^^,^^55^^,^^164^ Despite this, our neuron-level transmitter predictions guessed dopamine across all Kenyon cell types (FlyWire: 99.9%, HemiBrain: 99.9%) with high confidence (FlyWire: mean, 0.627, s.d., 0.99, HemiBrain: mean, 0.54, s.d., 0.046). A possible source of error might be contamination by nearby presynapses belonging to other neurons. For example, dopaminergic PAM neurons were used in our ground truth data, and are synaptic partners of Kenyon cells in the highly synapse-dense region of the mushroom body (Figure S3I). The cube of image data (edge length 640 nm) centered on each predicted presynaptic location was not masked with neuronal identity, and features from proximal presynapses may have skewed the result. However, if we consider presynapses on Kenyon cell axons (in the mushroom body lobes, dense dopaminergic innervation) and dendrites (mushroom body calyx, only sparse dopaminergic innervation^78^) separately, we see that dopamine is still the most common prediction for both (77% of axons and 95% of dendrites in FlyWire). This suggests that the confusion may arise either due to the uncommon biology of the Kenyon cell presynapse or because the network saw Kenyon cell presynaptic features near labelled dopamine presynapses during training - rather than observing dopamine presynaptic features near Kenyon cell presynapses when testing. More generally, across our dataset we observed a small percentage of axon-dendrite prediction mismatches in their compartment-level transmitter predictions (FAFB-FlyWire: 6.5%, hemibrain: 11.0%) (Figure S3G). In neurons where there was no mismatch, there was a strong correlation between the compartment-level transmitter prediction for the axon and the dendrite (Figure S3A), suggesting that the image features impacting our predictions are the same for both. Because axons are far from dendrites in Euclidean space, these features are most likely features of the neuron and/or its presynapses themselves.

Co-transmission with transmitters outside of our training data may be a major reason why our results can sometimes differ from what we expect. In mammals, cases of co-packaging of fast-acting clear core vesicular transmitters are known, including transmitters of opposing downstream effect.^172^ While transmitter-related gene expression and immunoreactivity are largely exclusive between the fast-acting transmitters in flies,^21^^,^^22^^,^^30^^,^^31^ most neuropeptides and monoamines can co-transmit with fast-acting transmitters.^21^^,^^163^ Co-transmission of especially fast-acting transmitters alongside neuropeptides and other neuromodulators is expected to be common in the brain, although examples at the resolution of individual cell types are sparse. The αβ and *γ* Kenyon cells are known to express the short neuropeptide F precursor, and immunostain for short neuropeptide F,^173^ which is highly indicative of neuropeptide transmission or co-transmission. Kenyon cells may also express the neuropeptide sparkly.^25^^,^^174^ In addition, *Ddc*, which encodes a protein responsible for converting L-DOPA to dopamine, is expressed in $\alpha^{\prime }\beta^{\prime }$ and *γ* Kenyon cells, where it could play a role in the biosynthesis of dopamine or another aromatic L-amino acid.^21^ Indeed, we did not include Kenyon cells in our ground truth data, because of this evidence for co-transmission.

The other major mispredictions we noticed include the singleton cell type DPM in the mushroom body, some olfactory sensory neurons of the antennal lobe and some central complex neurons such LPsP, vDelta, hDelta, and a few fan-shaped body tangential neurons, particularly in HemiBrain. The mushroom body, antennal lobe and central complex are especially synapse-dense regions of the brain as revealed by presynaptic antibody staining (see Figure S3I for regional biases in transmitter prediction). They are also known to exhibit diverse neuropeptide expression.^106^^,^^175^ DPM has been found to express the fast-acting transmitter GABA the monoamine serotonin and several neuropeptides (sparkly, nplp1, proctolin, orcokinin, eclosion hormone, CG34136).^25^^,^^56^^,^^60^ DPM were not used in our ground truth data and in both datasets are predicted to be dopaminergic. 20% of olfactory sensory neurons in HemiBrain and 25% in FlyWire are likely mispredicted as serotonergic instead of cholinergic.^64^ There is a glomerular pattern to the olfactory sensory receptor neuron cell types that are predicted serotonergic, and though this does not match up with known glomerular patterns for short neuropeptide F expression in sensory neurons^106^ it may match an as-yet unstudied neuropeptide distribution in the antennal lobe. Notably, these presynapses are in a region of the brain (antennal lobe) with a high density of true serotonergic presynapses from the CSD neurons, though the CSD is pan-glomerular. The CSD neurons were represented in our ground truth, but olfactory sensory neurons were not.

Central complex LPsP neurons are potentially mispredicted as glutamatergic instead of dopaminergic^78^^,^^176^ in FlyWire and HemiBrain and are in a region of the brain (protocerebral bridge) with a high density of true glutamatergic presynapses from the delta7 neurons. It is a rare case of a known dopaminerigc neuron to be mispredicted as something else in both datasets. Interestingly though, known DopR receptors are only weakly expressed in the region of LPsP output, the protocerebral bridge, and so LPsP may have in some way unusual dopaminergic presynapses.^177^ In addition, preliminary data suggests LPsP may co-transmit dopamine and glutamate (personal communication, Pablo Reimers). Both delta7 neurons and LPsP were represented in our ground truth data. Other protocerebral bridge neurons, IbSpsP neurons, were predicted for acetylcholine in FlyWire but for glutamate in HemiBrain.

The neurons of the optic lobe, despite being poorly represented in our ground-truth data, are remarkably well predicted. 96% of ∼ 29,000 optic lobe neurons (T4, T5, Tm2-4, Tm9, Tm20, L2-5, Lawf1-2) were correctly predicted acetylcholine. 87% of ∼ 3,600 optic lobe neurons (C2, C2, Pm4, Mi4, Dm10) were correctly predicted GABA. 91% of ∼ 1,600 optic lobe neurons (TmY5a, Dm8) were correctly predicted glutamate. However, some cell types exhibited a glutamate GABA confusion. ∼ 5,700 neurons from the known glutamatergic cell types (L1, Dm1, Dm3, Dm4, Dm11, Dm12) are correctly predicted a putative inhibitory class, but only 61% were predicted glutamate and 30% GABA. Our only unusual result was that just 3% of Dm9 neurons were predicted glutamate as expected. Dm9 neurons have ∼ 20-fold more autapses (self-connections) than usual,^178^ which may present a confound. Mi1 neurons have a similar rate of autapses and only 67% correct acetylcholine assignment. We note that one wet-lab study reports a different expression from what we have predicted in some optic lobe cell types, in a study based on mosaic analysis with repressible cell marker (MARCM) clones generated from drivers such as Cha-GAL4.^179^ These are TmY5a (acetylcholine from MARCM, glutamate from prediction), Mi2 (acetylcholine from MARCM, glutamate-GABA from prediction) and Pm1a (acetylcholine from MARCM, GABA from prediction). We believe that^179^ were misled by MARCM, our experience has been that clones generated from a Cha-GAL4 driver are not faithful to acetylcholine transmission.^142^^,^^159^

Our predictions have also provided a starting place for looking for dopamine, serotonin and octopamine neurons unknown to the literature, but particularly for serotonin we encourage data users to validate predictions in the wet lab. Genetic and histological studies have estimated there to be ∼ 130 dopaminergic neurons, ∼ 80 serotonergic neurons and ∼ 44 octopaminergic neurons^138^ in the brain. Discounting Kenyon cells and sensory receptor neurons, we have predicted 6052 dopaminergic, 2000 neurons and 289 octopaminergic neurons in FlyWire. Many of these cases likely indicate co-transmitting neurons, although especially for dopamine they may also reveal as-yet unknown dopaminergic cell types because the TH-GAL4 line most often used to find dopaminergic neurons is not completely faithful to dopamine transmission.^177^ Some known monoaminergic neurons were mispredicted. Two monoaminergic DNs, DNg28 (serotonin^180^) and DNg32 (octopamine^181^ - possibly because most of their presynapses are outside of the brain, in the ventral nerve cord. 114 photoreceptor neurons, R1-8, which may express histamine and possibly acetylcholine,^39^ are incorrectly predicted for octopamine in FAFB-FlyWire. We advise users to treat these predictions with caution.

In conclusion, by examining known cases of misprediction we think the main two reasons for misprediction are co-transmission and expression of transmitters outside of our training set. Both issues be remedied by expanding the training data with co-transmission information and by including neurons with other transmitters. The method presented here can be extended to support more than the six transmitters considered so far. The prediction of co-transmission will require an architecture change, as the current architecture can not confidently pick more than one transmitter at the time. One way this could be achieved is to replace the softmax layer in the current architecture with element-wise sigmoid activation functions. In addition to mispredictions stemming from co-transmission or other transmitters, rarer biology (such as autapses, unusual T-bars and unusual postsynapses) may also be a factor some of the time. Generally, we found that when there is conflict, the FlyWire predictions are often superior.

#### Working through fan-shaped body neuron expression

The fan-shaped body is a central brain neuropil that computes navigational variables, including internal goals for the fly. We focused on it because along with the antennal lobe we considered it one of the worst predicted parts of the brain in the HemiBrain dataset. In brief, it is built as a matrix with 9 rows and 10 columns.^77^ Tangential neurons input projects to specific, whole layers. Other input types (e.g., PFN) target specific columns within a single row. Intrinsic neurons can tile rows (e.g., hDelta types) or columns (e.g., vDelta types). Output neurons sample across several columns (e.g., FC and FS types) or more specific segments (e.g., PFL and FR types). We further found that almost all intrinsic cell types were predicted to transmit acetylcholine (∼ 88%), as well as almost all output cell types (∼ 96%) and column-specific input types (∼ 87%) with some misprediction due to a paucity of automatically detected presynapses in or near an unusual brain region^51^ known as the gall (Figure 5G, dashed circles). Synapse detection often fails in the gall,^77^ as presynapses here have elongated T-bars and dense core vesicles, indicative of neuropeptide co-transmission (Figure 5G, circles). We found that almost all tangential neurons are predicted to transmit glutamate (∼ 84%). Of the remainder, we are confident that 4 neurons (cell types: FB5AB, FB1G) are correctly predicted to transmit acetylcholine because they come from a cholinergic hemilineage (‘DM4 dorsal’) (Figure 7B). Indeed, an intersection with a ChA driver line has suggested that FB5AB and so-called ‘vFB’ neurons are cholinergic,^127^ though note that vFB morphology is not consistent with FB1G. Interestingly, the only neurons predicted to use GABA in both FlyWire and HemiBrain are FB5A (primary neurons from lineage ‘EBa1’) and FB3B, FB3C and FB3E (secondary neurons from lineage ‘EBa1’).^50^^,^^182^ Although the FB3 neurons are predicted for glutamate in FlyWire we suspect the HemiBrain prediction for GABA could be correct because ‘EBa1’ is majority GABAergic (Figure 7B). Indeed, metabotropic GABA receptors have only been found specifically in layers 3 and 5.^177^ A few dopaminergic tangential cell types have been reported (FB5H, FB6H, FB7B).^77^ In FlyWire only FB6H is predicted dopaminergic, the others are predicted to transmit glutamate. In HemiBrain, they are all correctly predicted dopamine. In both datasets, predicted dopaminergic neurons include: FB1C, FB1H, FB2A, FB4M and FB4Y. They all come from the known dopaminergic PPM3 cluster.^77^^,^^78^^,^^175^ FB4L also belongs to the PPM3 cluster but are predicted serotonin in both HemiBrain and FlyWire. Interestingly, TH, DopEcR and DopR1 immunoreactivity can be detected weakly across the fan-shaped body,^177^ likely by ExR2 and neurons receiving ExR2 input. However DopR1 is particularly strong in layers 1-2, DopR2 is expressed specifically and strongly in layer 3 (due to discrepancies between HemiBrain annotation and literature, “layer 3″ in other literature seems to be related to the projection pattern of many FB4 neurons), and D2R is expressed specifically in dorsal layers 7-8.^124^ This is in agreement with our predicted sites of dopamine transmission in the fan-shaped body.

We have also examined neuropeptides in the fan-shaped body as reported in the literature carefully, and here give our thoughts on how layer-specific expression may have caused some misprediction in this structure. Central complex hDelta cell types^77^^,^^90^^,^^127^^,^^183^ (not in our ground truth) are a set of 190 neurons whose morphologies are very similar, and which segment the fan-shaped body of the fly. However, our HemiBrain prediction results estimate 30% to be cholinergic and 45% to be dopaminergic. While the field lacks authoritative data, we expected them all to express the same transmitter or set of transmitters, most likely acetylcholine. Indeed, in FlyWire they come out as 83% cholinergic and 12% serotonergic, suggesting that all of the class should be cholinergic. The case is even worse with vDelta neurons, another similar class in the same neuropil born from the same set of hemilineages, for which HemiBrain predictions give us neuron-level transmitter predictions that are 40% glutamatergic and 35% dopaminergic, but are predicted 85% cholinergic in FlyWire. Fortunately, this issue is not common, only 14% of cell types in the HemiBrain have a transmitter prediction conflict. The fan-shaped body is known to express leucokinin, allatostatin A, short neuropeptide F, *Drosophila* neuropeptide F, tachykinin, proctolin, dFMRFa, SIFamide and myoinhibitory peptide in a highly specific and layer-wise manner.^106^^,^^175^^,^^177^^,^^184^ Because we expect all intrinsic and output types of the fan-shaped body to be cholinergic based on FlyWire results, we think the glutamate predictions for intrinsic cell types from HemiBrain are erroneous. The most notable example is the cholinergic hDeltaK neurons, which have been likely mispredicted to transmit serotonin in FlyWire and dopamine in HemiBrain because their presynapses lie in layer 6 of the fan-shaped body (Figure 5G, arrows), an unusual layer that is known to express allatostatin A, short neuropeptide F and innexin 6.^175^^,^^184^^,^^185^ hDeltaK is the only major case of intrinsic neuron misprediction in FlyWire. hDeltaC and hDeltaL are also likely mispredicted to transmit dopamine in HemiBrain and have their axons in layer 6. In addition, short neuropeptide F has been shown to co-localise in 5-6 neurons with cholinergic transmission specifically in layers 5-6,^175^ indicative that another hDelta class co-transmits it because vDelta neurons per layer are more numerous. In particular, hDeltaF and hDeltaG are likely mispredicted to transmit dopamine in this region. We suspect one of those types co-transmits short neuropeptide F. hDeltaJ (layer 4) and hDeltaM (layer 5) are predicted to transmit dopamine in HemiBrain but acetylcholine in FlyWire. It is less clear what neuropeptide expression, or not, may have caused these confounds, but we note that dFMRFa is expressed in layer 4, and SIFamide and proctolin are expressed in layer 5.^175^^,^^186^ suspect tachykinin expression in vDeltaA neurons, which are majority mispredicted to transmit dopamine in HemiBrain and minority mispredicted to transmit serotonin in FlyWire. However, only 16-18 neurons are thought to express tachykinin and there are over 80 vDeltaA neurons. It is also possible that the 16 hDeltaD/E neurons express tachykinin, half of which are mispredicted dopamine in HemiBrain, but likely correctly acetylcholine in FlyWire.

There are also a few mispredictions of glutamatergic tangential neurons, particularly in HemiBrain. Mispredictions are probably lower in these tangential neurons because glutamate has been shown mainly not to co-transmit with the most abundant neuropeptides in the fan-shaped body – short neuropeptide F, tachykinin and myoinhibitory peptide.^175^ However, leucokinin neuropeptide is expressed in layer 6 tangential neurons,^184^ e.g., FB6A, which are erroneously predicted octopaminergic in HemiBrain but perhaps correctly for glutamate in FlyWire. We think it is likely that FB6A co-transmits glutamate and leucokinin. Tachykinin is also expressed in layer 2 of the fan-shaped body. Because it does not co-localize with ChAT stains in this layer and because of cell body placement, it might be expressed by specific glutamatergic tangential neurons^177^^,^^187^ and may account for why FB2B is mispredicted octopaminergic in HemiBrain, and FB2A and FB2I predicted dopaminergic in FlyWire and HemiBrain. Allatostatin A is expressed in layer 6 in neurons other than “dFB" FB6A neurons.^188^ Because in HemiBrain FB6H/L/K/J are potentially mispredicted to transmit dopamine (likely correctly glutamate in FlyWire) we wonder if these could be the allatostatin A neurons. Proctolin is expressed in layer 5 and because FB5S has some dark vesicles and 1/7 in HemiBrain was predicted for octopamine, we wonder whether this is a proctolin co-transmitting type. Curiously, while most 5-HT receptors are not expressed in the fan-shaped body,^177^ serotonin immunolabeling revealed that serotonin is used in layers 3, 6 and more ventral layers.^150^^,^^175^ One serotonergic source is ExR3, which projects to layer 6, but there may be others innervating more ventral layers. ExR3 is mispredicted to transmit octopamine in HemiBrain and dopamine in FlyWire, and has dense core vesicles indicative of unknown neuropeptide co-transmission. A final notable misprediction is the numerous, expected cholinergic class PFNp, which receives various mispredicted labels in HemiBrain and FlyWire. However, the class appears to have very few detected output presynapses, only a mean of 56 in HemiBrain, indicative of little or unusual neurotransmission, or else automatic synapse detection was problematic in the dorsal nodulus and layer 1 of the fan-shaped body.

#### Working through antennal lobe local neuron expression

We tackled in detail a difficult case concerning a morphologically variable and diverse class of neurons^105^ from two split transmitter hemilineages: local neurons of the antennal lobe. We found we could still make sense of our neuron-level transmitter predictions in relation to the extant literature. We wanted to draw attention to this case because transmitter usage in these local neurons seems to break Dale’s law,^104^^,^^106^ Lacin’s law (Figure 7C) and the expectation of uniform transmitter expression within a type (Figures S5F and S5G), and so presented perhaps the biggest challenge for our resource. They are known to express acetylcholine,^63^^,^^189^^,^^190^ GABA,^62^^,^^89^^,^^151^ glutamate^61^^,^^89^^,^^104^ and various neuropeptides transmitters.^106^

‘ALl1 dorsal’ is a local neuron hemilineage that our Bayesian analysis identified as having a decisive split in its transmitter usage. From the literature, we expected ∼ 54 local neurons per hemisphere of which ∼ 90% would be GABAergic.^151^ We also expected perhaps as many as ∼ 15 cholinergic local neurons.^63^^,^^189^ In our connectomic datasets, we find ∼ 126 local neurons per hemisphere for ‘ALl1 dorsal’.^74^ We found that 143 (left: 74, right: 69) are predicted GABAergic, 48 (left: 20, right: 28) cholinergic, 13 (left: 9, right: 4) dopaminergic, 14 (left: 7, right: 7) glutamatergic and 37 (left: 18, right: 19) serotonergic. Serotonin and dopamine in the antennal lobe have been well-studied but no reports of either in local neurons exist in the literature. We, therefore, think that these neurons should mostly have been predicted GABAergic or cholinergic, perhaps mostly the latter based on morphological similarity (Figure 7E). However, the other predictions could be valid, albeit surprising. The predicted cholinergic neurons are unilateral and ‘broad’, i.e., pan-glomerular, in accordance with previous descriptions^63^^,^^189^ but more numerous. Positioned closely among them are other local lLN1_bc neurons that are predicted to be dopaminergic. We suspect that some cholinergic local neurons have been mispredicted dopaminergic because at least ∼ 3 cholinergic neurons co-express allatostatin A and ∼ 10-15 co-express myoinhibitory peptide.^106^ The predicted GABAergic local neurons in the hemilineage have cell body fiber tracts slightly offset from the cholinergic/dopaminergic subgroup. Half the local neurons that had previously been categorized as LN2 ‘patchy’ local neurons,^74^^,^^105^ i.e., those that innervate discontinuous glomeruli in space, were predicted glutamatergic (cell type: lLN2P_a) and half GABAergic (cell types: lLN2P_b, lLN2P_c). Analogously ‘picky’ local neurons in the larval brain are also known to be glutamatergic, while the grossly morphologically similar ‘choosy’ neurons are GABAergic.^191^ A subset of lLN1 neurons is known to express both GABA and glutamate to effect synaptic plasticity, but it is not known how large this subset is or whether this phenomenon extends to other local neuron classes.^104^ Some of these neurons have been predicted GABAergic and some mispredicted serotonergic. Tachykinin is also known to co-express in ∼ 40 GABAergic ‘ALl1 dorsal’ neurons per hemisphere^106^ and may have been a confound that generated serotonin predictions. Specifically, among them are ∼ 13 lLN2T_abc neurons that have a cell body placement and general morphology most similar to reported cholinergic local neurons.^63^^,^^189^ The remainder is most likely GABAergic, e.g., lLN2_d gross morphology looks more like related GABAergic neurons.^63^^,^^189^

The remaining, largely bilateral, local neurons come from a second hemilineage ‘ALv2’. This too, appears to be a split hemilineage containing 133 (left: 67, right: 66) glutamatergic neurons alongside a newly identified sub-population of 28 (left: 14, right: 16) potential cholinergic neurons. Previous immunohistochemistry work^61^ found no sign of GABA usage at ‘ALv2’ presynapses, and we predict only one ‘ALv2’ GABAergic neuron on each side (v2LN41), perhaps a deviant first-born neuron of the hemilineage. One ‘ALv2’ local neuron was predicted serotonergic (v2LN36), this unusual neuron had previously been determined to be glutamatergic using immunohistochemistry and able to undergo stark morphological changes dependent on sex and mating state.^192^ ‘ALv2’ axons extend to specific glomeruli in the contralateral antennal lobe. Significantly, this means that specific lateral excitation between glomeruli on different sides of the brain should be possible. As with their glutamatergic counterparts, these sparse bilateral local neurons focus on connections mainly between thermosensory glomeruli.^74^ Excluding serotonin and dopamine mispredictions our HemiBrain predictions were similar, but with some misprediction of ‘ALv2’ neurons as using GABA rather than glutamate. We have a little further detail on local neuron misprediction in our Methods section on misprediction correlates. In conclusion, we think there could be far more cholinergic local neurons in the antennal lobe than previously thought which has implications for second-order olfactory information processing. See Figures S5F and S5G. We thank Asa Barth-Maron for discussions and insights into antennal lobe local neurons.

### Hemilineage definition in *D. melanogaster*

The neurons of the central nervous system are generated by a set of stem cells known as neuroblasts. During division, neuroblasts generate two cells, one additional stem cell and one cell that further divides into two sibling neurons. In only one of these siblings, the so-called Notch pathway is activated, leading to two different *hemilineages* of neurons within each lineage, one Notch positive the other Nothc negative^31^^,^^193^^,^^194^^,^^195^ (or more than two in the case of Type II neuroblasts, which mainly contribute to the central complex, Figure S5A). In some cases, one of a lineage’s hemilineage may apoptose,^196^ leaving only a single hemilineage. Neurons can be born in this way during embryogenesis and are known as primary neurons (∼ 10% of the adult brain^134^^,^^197^). Many neuromodulatory neurons appear to be primary.^138^^,^^141^ Those made during larval development are known as secondary neurons. We found 120 lineages that broke down into 183 hemilineage-associated tracts, per hemisphere. Of these, we think 144 are secondary Type I hemilineages. The remainder are secondary Type II. These eight lineages (Type II; ‘DM1-6’; ‘DL1-2’) produce intermediate progenitors, each in turn delivering two hemilineages. Their relationship with their lineage-associated tracts is often less clear.

Neuronal fibers co-fasciculate with neurons from the same hemilineage and form bundles as they enter the neuropil from the insect brain’s outer layer of cell bodies. Only secondary hemilineages (each ∼ 100 neurons) can easily be demarcated in adult *D. melanogaster*. In EM data annotators can make our “lineage-associated tracts" (sometimes called “secondary axon tracts"), many of which contain a single hemilineage. It should be noted that cases have been identified^102^ where a single lineage-associated tract is composed of two hemilineages. Type II hemilineages may be less uniform than Type I in their tract organization. Each intermediate progenitor of a Type II lineage behaves like a “small neuroblast" and produces 10-20 neurons, divided into two hemilineages.^198^ According to this idea, there should be around 50-100 hemilineages for each type 2 neuroblast. That does not match the morphologically visible tracts in BP106 strings in the larva or adult central brain.

Our lineage-associated tracts have been mapped by light microscopy and assigned to named lineage clones.^139^^,^^199^^,^^200^^,^^201^^,^^202^ We have discovered them in the adult brain FAFB dataset.^50^ Lacin et al.^31^ have shown that Each hemilineage in the adult ventral nerve cord uses just one of the fast-acting transmitters, acetylcholine, glutamate or GABA, even though mRNA transcripts for combinations of these can appear in the nucleus. We have found the same with our predictions in the ventral nerve cord.^65^ We refer to this principle as *Lacin’s law*. Using the FlyWire dataset we show in this work that it largely holds in the adult central brain with some interesting exceptions (Figure 7).

Because HemiBrain dataset is only a partial brain, many neurons have large missing portions or do not exist in this dataset, causing a discrepancy between neuron count and hemilineage content compared with FlyWire. However, we could still identify most central brain hemilineages and see that their predicted transmitter expression and projection envelope are similar to those of FlyWire neurons (Data S6). Comparing GABA-glutamate mixed hemilineages (‘LHl1’, ‘VLPl4 dorsal’, ‘LHl4 dorsal’, ‘SLPav3’, ‘WEDd2’, ‘VLPl4 anterior’, ‘VPNp&v1 posterior’, ‘CREa1 ventral’, ‘VESa1’, ‘EBa1’, ‘DM2 central’) in FlyWire to HemiBrain indicates that perhaps they more uniformly express GABA (Figure S5A). On the other hand, HemiBrain predictions were more likely than FAFB-Catmaid predictions to confuse glutamate for GABA, but not *vice verse*(Figure 2A). In addition, dopamine misprediction seems be higher in HemiBrain, ‘CLp1’, ‘SMPpv2 dorsal’, ‘SMPpm1’, ‘VLPd&p1 posterior’, ‘SMPpv1’, ‘SMPpd1’, ‘VLPl&p1 posterior’, ‘CREl1’, ‘LHl2 dorsal’, ‘SLPpm3’, ‘VPNp1 medial’, ‘DM6 dorsolateral’, ‘DM3 dorsolateral’ have predicted dopaminergic neurons only in HemiBrain. The largest discrepancy was for ‘DM6 IbSpsP’. IbSpsP neurons were predicted for acetylcholine in FlyWire but for glutamate in HemiBrain.

#### Hemilineage assignments in *D. melanogaster*

Our work assigning neurons in the FAFB-FlyWire dataset to hemilineages has been reported in Schlegel et al..^50^ We briefly detail the process here. Cell body fiber tracts for identified hemilineages had previously been identified using TrakEM2^203^ in a light-level atlas for a *D. melanogaster* brain, stained with an antibody against neurotactin (BP104).^199^ We extracted these expertly identified tracts and registered them into a common template brain, JFRC2, using CMTK,^204^ and then into FAFB space.^133^ These lineage-associated tracts could then be be readily identified in EM stacks based on their point of entry and subsequent trajectory. We generally consider lineage-associated tracts as fiber units belonging to one hemilineage. However, we suspect that the putative hemilineages ‘DL1 dorsal’, ‘DL1 ventral’, ‘DL2 dorsal’ and ‘DL2 ventral’ (derived from the Type II lineages ‘DL1’ and ‘DL2’) may be examples where this has happened, and each of these groupings is a composite of not one but two hemilineages (giving four hemilineages per lineage here, which is possible because these are Type II lineages,^205^^,^^206^^,^^207^ a Type II would have two). We identified cell body fiber tracts in the ssTEM FAFB dataset in the vicinity of the transformed lineage-associated tracts using the flywire.ai Web interface.^17^ We compared our candidate neurons to registered images of lineage clones, produced using genetic tools and light microscopy.^139^^,^^201^ Hemilineages can be told apart in an image of a lineage clone, by the different placement of their cell body clusters, and the tract their cell body fibers take into the neuropil. From light-level data that labels a whole lineage and all of its hemilineages together,^139^^,^^199^^,^^200^^,^^201^ it is occasionally difficult to assess whether there is a single hemilineage or multiple, if the cell body clusters happen to not separate sufficiently. While they can be told apart using developmental genetic tools, e.g., in the case of ‘LALv1’,^102^ this has not been done at scale.

In Figure 7E we focused on birth order in ‘LALv1’ because ‘LALv1 ventral’ exhibits split transmitter use. ‘LALv1’ is, fortunately, one of few hemilineages whose birth order has been delineated.^102^ ‘LALv1’ neurons were matched 1:1 to FAFB-FlyWire reconstructions with a combination of visual inspection and morphological clustering^47^ to the morphological progression by birth order^102^).

We have used 183 well-annotated hemilineages of the central brain, which were human-reviewed on both the right and left FAFB-FlyWire hemispheres, in this work. However, assigning neurons to hemilineages is difficult. For example, neurons from the lineage ‘LALv1’ co-bundle between its two hemilineages, making their disambiguation dependent on examining light-level data and single neuron clones.^102^ Without prior work^102^ on this lineage’s detailed composition, we might have incorrectly assumed that its glutamatergic and cholinergic neurons from its two separate hemilineages intermingled in a single hemilineage. High-quality light-level data is not available for every hemilineage, meaning that we had to make some judgment calls in our hemilineage discrimination.

Indeed, the ‘DL1’ hemilineages at first seemed a striking example of split transmitter use within hemilineages, i.e., disobeying Lacin’s law. However, it is possible that the ‘DL1’ and ‘DL2’ hemilineages we have delineated are actually each not a single hemilineage but two, i.e., there are four hemilineages in total for ‘DL1/2’.^205^^,^^206^^,^^207^ The split transmitter use may reveal this hemilineage-based division in a lineage-associated tract that was otherwise hard for human annotators to make. Therefore we suspect that our split-use label in this case reveals an issue of data annotation.

#### Hemilineage prediction entropy

##### Neuron level entropy

To quantify multimodality of transmitter predictions on neuron level within a hemilineage, we calculated the entropy *H* of the transmitter distribution over neurons in the following way: Let $n\in N_{h}$ be a neuron in hemilineage *h* and ${\hat{y}}_{n}\in Y=$ {GABA, acetylcholine, glutamate, serotonin, octopamine, dopamine } the predicted transmitter of neuron *n*. Then

(Equation 2) $$H\left(N_{h}\right)=-\sum_{y\in Y}p_{h}\left(y\right)\log_{6}p_{h}\left(y\right),\text{with}$$

(Equation 3) $$p_{h}\left(y\right)=\frac{1}{\left|N_{h}\right|}\sum_{n\in N_{h}}\delta \left({\hat{y}}_{n}=y\right)\text{.}$$

A value of $H\left(N_{h}\right)=0$ then means that all neurons within hemilineage *h* have the same predicted transmitter, while a value of $H\left(N_{h}\right)=1$ means that within hemilineage *h* all predicted transmitters are equally common.

##### Synapse level entropy

Similarly, we quantified the average multimodality over synapses within neurons of a given hemilineage: Let $s\in S_{n}$ be the synapses in neuron $n\in N_{h}$ of hemilineage *h* and ${\hat{y}}_{s}$ the predicted transmitter. The entropy of predicted synaptic transmitters $H\left(s_{n}\right)$ in neuron n is then given by:

(Equation 4) $$H\left(S_{n}\right)=-\sum_{y\in Y}p_{n}\left(y\right)\log_{6}p_{n}\left(y\right),\text{with}$$

(Equation 5) $$p_{n}\left(y\right)=\frac{1}{\left|S_{n}\right|}\sum_{s\in S_{n}}\delta \left({\hat{y}}_{s}=y\right)$$

With this the average synaptic entropy over all neurons within hemilineage *h* is given by:

(Equation 6) $$H\left(S_{h}\right)=\frac{1}{\left|N_{h}\right|}\sum_{n\in N_{h}}H\left(S_{n}\right)$$

A value of $H\left(S_{h}\right)=0$ means that all synapses of all neurons in hemilineage *h* have the same predicted transmitter, while a value of $H\left(S_{h}\right)=1$ means that in all neurons within hemilineage *h* all synaptic transmitter predictions are equally common. Figure 7D shows the distribution of $H\left(N_{h}\right)$ and $H\left(S_{h}\right)$ of all predicted hemilineages’ neurons that have more than 30 synapses each.

On the population level, we found relatively higher values of $H\left(S_{h}\right)$ (Synapse level entropy) than $H\left(N_{h}\right)$ (Neuron level entropy). 75% of hemilineages show a synapse level entropy below $q_{75}\left(H\left(S_{h}\right)\right)=0.41$ as compared to $q_{75}\left(H\left(N_{h}\right)\right)=0.20$. This is reassuring as it suggests less variation of transmitter identity of neurons within a hemilineage compared to variations of transmitter identity predictions within individual neurons, meaning there is improved consensus of predictions when aggregating across populations. However, in cases with a high level of synaptic entropy, such as hemilineage ‘TRdl_a’, it is less clear whether neuron-level multimodality is an artifact of uncertain, multimodal predictions on the synapse level of individual neurons. In contrast, hemilineages such as ‘SMPpd1’ show high neuron level entropy $H\left(N_{h}\right)\geq q_{75}$ but low synapse level entropy $H\left(S_{h}\right)\leq q_{25}$, suggesting clear neuron level segregation of predicted transmitters within those hemilineages. Hemilineages such as ‘ALad1’ with $H\left(S_{h}\right)\geq q_{25}$ and $H\left(S_{n}\right)<q_{25}$ appear homogeneous within each neuron and within the entire hemilineage.

#### Probability to observe transmitter predictions $\hat{y}$

Given a neuron has true transmitter y∈Y, the probability that we predict transmitter $\hat{y}\in Y$ (assuming that each prediction is independent and identically distributed) is given by the categorical distribution

(Equation 7) $$p\left(\hat{y}|y\right)=C_{y,\hat{y}}\text{,}$$

where *C* is the neuron confusion matrix obtained on the test dataset (see Figure 2A).

Let *m* be the number of different transmitters in hemilineage *h*. We model the probability $p\left(\hat{y}|m\right)$ of observing transmitter predictions $\hat{y}=\left\{{\hat{y}}_{0},{\hat{y}}_{1},\ldots ,{\hat{y}}_{n}\right\}$ under the assumption that hemilineage *h* contains *m* different transmitters. Here, ${\hat{y}}_{j}$ is the predicted transmitter of neuron *j* in hemilineage *h* with *n* neurons total. Let $P_{c}\left(Y\right)$ be the set of subsets of true transmitters *Y* with cardinality *c*, then:

(Equation 8) $$p\left(\hat{y}|m\right)=\sum_{S\in P_{m}\left(Y\right)}p\left(\hat{y}|S\right)\cdot p\left(S|m\right)\text{,}$$

where $p\left(\hat{y}|S\right)$ is the probability to observe predictions $\hat{y}$ if the hemilineage has true underlying transmitters y∈S and p(S|m) is the probability for the set of true transmitters *S* given the hemilineage contains *m* different transmitters. Since we assume i.i.d. predictions $\hat{y}$, $p\left(\hat{y}|S\right)$ factorizes as follows:

(Equation 9) $$p\left(\hat{y}|S\right)=\prod_{j}p\left({\hat{y}}_{j}|S\right)$$

and marginalizing over y∈S yields:

(Equation 10) $$p\left(\hat{y}|S\right)=\prod_{j}\sum_{y\in S}p\left({\hat{y}}_{j}|y\right)\cdot p\left(y|S\right)$$

(Equation 11) $$=\prod_{j}\sum_{y\in S}C_{y,{\hat{y}}_{j}}\cdot p\left(y|S\right)\text{.}$$

Regarding p(S|m) and p(y|S) we assume a flat prior, i.e.,:

(Equation 12) $$p\left(S|m\right)={\left(\begin{array}{c} \left|Y\right|\\ m \end{array}\right)}^{-1}$$

(Equation 13) $$p\left(y|S\right)=\frac{1}{\left|S\right|}=\frac{1}{m}\text{.}$$

With this, the probability of observing predictions $\hat{y}$ given *m* different transmitters becomes:

(Equation 14) $$p\left(\hat{y}|m\right)={\left(\begin{array}{c} \left|Y\right|\\ m \end{array}\right)}^{-1}\sum_{S\in P_{m}\left(Y\right)}\left(\prod_{j}\sum_{y\in S}C_{y,{\hat{y}}_{j}}\cdot \frac{1}{\left|S\right|}\right)$$

#### Bayes Factor

With this formalism in place, we can compare hypotheses about the number of true transmitters *m* in a given hemilineage by using the Bayes Factor $K=\frac{p\left(D|M_{1}\right)}{p\left(D|M_{2}\right)}$, where D is our observed data (predicted transmitters) and $M_{1}$, $M_{2}$ are two models about the underlying true transmitters that we wish to compare. The Bayes factor for a model $M_{1}$ with $m_{1}$ true transmitters per hemilineage and model $M_{2}$ with $m_{2}$ different transmitters is given by:

(Equation 15) $$K_{m_{1},m_{2}}=\frac{p\left(\hat{y}|m_{1}\right)}{p\left(\hat{y}|m_{2}\right)}$$

(Equation 16) $$=\frac{{\left(\begin{array}{c} \left|Y\right|\\ m_{1} \end{array}\right)}^{-1}\sum_{S\in P_{m_{1}}\left(Y\right)}\left(\prod_{j}\sum_{y\in S}C_{y,{\hat{y}}_{j}}\cdot \frac{1}{m_{1}}\right)}{{\left(\begin{array}{c} \left|Y\right|\\ m_{2} \end{array}\right)}^{-1}\sum_{S\in P_{m_{2}}\left(Y\right)}\left(\prod_{j}\sum_{y\in S}C_{y,{\hat{y}}_{j}}\cdot \frac{1}{m_{2}}\right)}\text{.}$$

So far, we assumed that $p\left({\hat{y}}_{j}|y\right)=C_{y,{\hat{y}}_{j}}$, i.e., we estimated this distribution on the test dataset. However, because our test set is finite we can not expect that the estimated error rates perfectly transfer to other datasets. To relax our assumptions about this distribution we simulated additional errors, by incorporating additive smoothing on the counts of neurons $N_{y,\hat{y}}$ that have true transmitter *y* and were predicted as transmitter $\hat{y}$, i.e.,:

(Equation 17) $${\tilde{C}}_{y,\hat{y}}=\frac{N_{y,\hat{y}}+\beta }{\sum_{\hat{y}}N_{y,\hat{y}}+6\beta }\text{,}$$

where $\beta \in N_{0}$ is the smoothing parameter. With $C_{y,\hat{y}}=\frac{N_{y,\hat{y}}}{\sum_{\hat{y}}N_{y,\hat{y}}}$ we then have

(Equation 18) $${\tilde{C}}_{y,\hat{y}}=\frac{C_{y,\hat{y}}+\frac{\beta }{\sum_{\hat{y}}N_{y,\hat{y}}}}{1+6\frac{\beta }{\sum_{\hat{y}}N_{y,\hat{y}}}}=\frac{C_{y,\hat{y}}+\alpha }{1+6\alpha }$$

and $\alpha \in R_{\geq 0}$ the count normalized smoothing parameter. In the limit of α→∞, ${\tilde{C}}_{y,\hat{y}}$ approaches the uniform distribution with probability 1/6 for each transmitter, whereas a value of α=0 means we recover the observed confusion matrix *C*. With this our distributions are now parametrized by *α* and the Bayes factor becomes:

(Equation 19) $$K_{m_{1},m_{2}}=\frac{\int_{\alpha }p\left(\hat{y},\alpha |m_{1}\right)p\left(\alpha \right)d\alpha }{\int_{\alpha }p\left(\hat{y},\alpha |m_{2}\right)p\left(\alpha \right)d\alpha }$$

, (Equation 20) $$=\frac{\tilde{p}\left(\hat{y}|m_{1}\right)}{\tilde{p}\left(\hat{y}|m_{2}\right)}$$

where $\tilde{p}\left(\hat{y}|m\right)$ is as defined in 14 but with $C_{y,{\hat{y}}_{j}}$ replaced with its expected value $E_{p\left(\alpha \right)}\left[{\tilde{C}}_{y,{\hat{y}}_{j}}\right]$.

The prior distribution on *α*, p(α) allows us to encode our prior knowledge about *α* and use it to weight the likelihood of the corresponding model. Given the data, a value of α=ϵ with epsilon small (0<ϵ≪1), should be most probable, while the probability of values α>ϵ should monotonically decrease as we deviate more from the observed confusion matrix. Values of α<ϵ should have probability zero, because they correspond to the un-smoothed confusion matrix with zero entries, i.e., a probability of zero for mis-classification of certain transmitters. While these probabilities may be small, they are likely greater than zero and an artifact caused by the finite test set. Many distributions fulfill these criteria, in particular the family of exponential distributions with rate parameter *λ*:

(Equation 21) $$p\left(\alpha \right)=\left\{ \begin{array}{cc} \lambda e^{-\lambda \left(\alpha -\epsilon \right)} & \alpha \geq \epsilon \\ 0 & \alpha <\epsilon \end{array}\right.\text{.}$$

Thus *λ* controls the weight for smoothing parameter *α* in the integral $E_{p\left(\alpha |M\right)}\left[\tilde{C}{\left(\alpha \right)}_{y,{\hat{y}}_{j}}\right]=\int_{\alpha }{\tilde{C}}_{y,{\hat{y}}_{j}}p\left(\alpha \right)d\alpha$. For λ→0, the expected confusion matrix approaches the unweighted average of all C(α) in the integration range. For λ→∞, the expected confusion matrix approaches the *ϵ*-smoothed confusion matrix ${\tilde{C}}_{y,\hat{y}}=\frac{C_{y,\hat{y}}+\epsilon }{1+6\epsilon }$.

The rate parameter *λ* can also be understood via its influence on the expected average accuracy ${\tilde{c}}_{\exp}=\frac{1}{6}\sum_{i}E_{p\left(\alpha |M\right)}{\left[\tilde{C}\right]}_{i,i}$. For values of λ→0, the expected accuracy approaches chance level while for values of λ→∞, the expected accuracy approaches the *ϵ*-smoothed, observed accuracy on the test set.

To summarize overall maximum likelihood of number of true transmitters in a given lineage, for a fixed *λ* we consider a one-versus-rest Bayes factor:

(Equation 22) $$K_{m,\neg m}=\frac{\tilde{p}\left(\hat{y}|m\right)}{\sum_{n\neq m}\tilde{p}\left(\hat{y}|n\right)}$$

#### Identification of ultrastructural features

We identified ultrastructural features by visualizing relevant differences between pairs of transmitters. To this end, we trained CycleGANs^72^ to translate original images of presynapses from one transmitter to counterfactual images of another transmitter. Since a single CycleGAN can translate images in both directions (e.g., the same CycleGAN can be used to translate GABA → glutamate and glutamate → GABA), this required training of a total of 15 CycleGANs to translate each of the six transmitters into any other transmitter. Each CycleGAN was trained on the same FAFB training dataset we used to train our synapse classifier, but limited to 2D images for efficiency.

For each pair of transmitters (i,j), we then translated each original synapse image of class *i* into a counterfactual image of class *j*. We confirmed that the translation was successful by classifying both the original and counterfactual image. To this end, we retrained our synapse classifier on 2D images as well and only kept a pair of images if the original image was correctly classified as *i* and the counterfactual as *j*, with a score of at least 0.8 for each. For each image pair, we then found the smallest mask such that swapping the contents of this mask between the original and counterfactual image flips the classification (details of this procedure are given in^71^). We then sorted all image pairs by the size of this mask and visually inspected the 40 pairs with the smallest mask between each pair of transmitters (Data S8).

We took note of visual differences between the original and counterfactual images within the masked region to obtain hypotheses about ultrastructural features. Crucially, we only included differences that were symmetric between translations: for each observed difference, we required that the difference is visible when translating from class *i* to *j*, as well as the inverse when translating from *j* to *i*.

#### Advice for resource users

We have publicly released (Zenodo, https://doi.org/10.5281/zenodo.10593546) our comprehensive results encompassing the entire central nervous system (FAFB-FlyWire, HemiBrain and MaleVNC). We thank Davi Bock, Eric Perlman and Stuart Berg and the flywire.ai project for helping us make our preliminary results available to the community via CAVE. We thank Clare Pilgrim, Alex McLachlan and David Osumi-Sutherland for incorporating our predictions into Virtual Fly Brain. Researchers^18^^,^^50^^,^^65^^,^^74^^,^^76^^,^^90^^,^^114^^,^^202^^,^^208^^,^^209^^,^^210^^,^^211^ have already utilized our preprinted results to formulate neurobiological hypotheses. The most reliable predictions align across hemispheres, exhibit cohesion within a cell type, adhere to Lacin’s law and remain consistent across datasets. We have provided two worked examples of less reliable cases, tackling misprediction among major fan-shaped body classes and antennal lobe local neurons. Considerations for users include: (i) Dale’s law in the insect should be taken as mutually exclusive between acetylcholine, glutamate and GABA usage but we expect co-transmission with both monoamines and neuropeptides to be common. (ii) Confounds will produce an error rate that may differ between neuron classes. A user can note higher-level features to provide greater clarity, e.g., Lacin’s law. Users should scrutinize neuron-level transmitter predictions that do not agree with the majority vote in a hemilineage (Data S6), in particular, GABA versus glutamate confusions (e.g., Figures 5G and S5A). True deviations may occur for distinct morphological subsets or among first-born neurons. (iii) Direct inspection of presynapses in EM data can reveal confounds, such as irregular T-bars or large dense core vesicles for co-transmitted neuropeptides. Unusual-looking presynapses are less likely to yield trustworthy predictions. (iv) Caution is advised for serotonin results, often indicative of uncertainty, while unreported octopamine results may only suggest dense core vesicle use (Figures 2D and S2). (v) One can be more confident in results that are consistent for the same cell type between hemispheres and datasets (Figure 4). Where there is conflict, we often find our FAFB-FlyWire predictions to be more reliable. (vi) Given the neuron-level transmitter prediction confidence scores we see for well-matched neurons, we suggest that if users wish to be conservative they could use a stringent neuron-level transmitter prediction confidence score threshold of ∼ 0.62 and ∼ 0.53 for FAFB-FlyWire and HemiBrain neurons respectively (1 s.d. lower than the mean score among concurring matches). (vii) One needs also to be wary of the opposite roles glutamate could play in a circuit. (viii) Our model is forced to select one of the six classes, even if there is no evidence for any of them.

### Quantification and statistical analysis

All violin plot data is presented as the 25th percentile, the median and the 75th percentile, unless otherwise indicated. Unless otherwise indicated, all statistical tests were Wilcoxon two-sample tests, unless otherwise indicated. The Bayes analyses performed and our confidence score calculation are described in our method detail. Statistical comparisons were analyzed R. The N in our analyses are indicated either in the figure legend or in the figure panels themselves. Our Figure 5A provides the total number of neuronal reconstructions by dataset (FAFB-FlyWire: 136,927, HemiBrain: 24,666). For Figures 5D–5F, S3E–S3H, and S4) a subset of total central brain neurons that were skeletonised were used in analysis (FAFB-FlyWire: 88,115, HemiBrain: 11,277). Counts by class, lineage, hemilineage, cell type and matches between datasets (shared cell_type column) can be found in our supplementary data (FAFB-FlyWire: Data S4, HemiBrain: Data S3). Figures were prepared in R^130^ using Ggplot2^132^ and were arranged using Adobe Illustrator.

## Supplemental information

Document S1. Supplemental references

Data S1. A .csv file that provides each cell type (cell_type) we used to generate our ground-truth data, the transmitter it is has been reported to express from the literature (known_nt), the study that made this report (known_source), and the type of evidence it contained (evidence), related to Figure 1 Note that we used this data to build our original ground truth, but have since made a wider effort to annotate known transmission for validation, reflected in Data S3 and S4, columns known_nt and known_source.

Data S2. A .csv file that indicates each neuronal reconstruction used to generate our ground-truth data, related to Figure 1 Presynapses from each reconstruction were used. Columns provide a unique identifier (id) (which is the root ID for a flywire neuron, a skeleton ID for a FAFB-Catmaid neuron and a bodyid for a HemiBrain neuron), the designated transmitter from the literature (known_nt), its cell type (cell_type), and the dataset from which the neuron comes (dataset). Full set of presynaptic locations available upon request. FAFB-Catmaid reconstructions can be found on virtual fly brain: https://fafb.catmaid.virtualflybrain.org/. Flywire reconstructions can be found at: https://ngl.flywire.ai/. HemiBrain reconstructions can be found at: https://neuprint.janelia.org/?dataset=hemibrain. We used version 630 in this study.

Data S3. A .csv file in which each row is a single identified HemiBrain reconstruction, related to Figures 2 and 5 Includes all such neurons used in our analysis. Columns provide each neuron’s unique identifier (bodyid), morphological cell type (cell_type), number of output synapses (pre), whether or not the neuronal reconstruction is cut off by the HemiBrain volume (cropped), neuron-level transmitter prediction (conf_nt), the confidence score for that prediction, which was calculated using the synapse-level confusion metrics and with synapse filtering (conf_nt_p), see STAR Methods, neuron-level transmitter prediction with no synapse filtering for axon/dendrite (top_nt, top_nt_p), the proportion of synapse-level transmitter predictions that “voted" for each transmitter (transmitter), the segregation index, a measure of neuronal polarity^S1^ (segregation_index), the projection score, a measure of the distance between axon and dendrite (projection_score), the hemilineage to which this neuron belongs (ito_lee_hemilineage, hartenstein_hemilineage), whether or not presynapses from this neuron were included in our ground-truth data (in_ground_truth).

Data S4. A .csv file where each row is a single identified FAFB-FlyWire reconstruction, related to Figure 2 and Figure 5 Includes all such neurons used in our analysis. Columns provide each neuron’s unique identifier from the 630 materialization of the FAFB-FlyWire dataset used in this paper (root_id_630), and the newer 783 materialization of the FAFB-FlyWire dataset (root_id_783), the neuron’s (cell_type), neuron-level transmitter prediction (conf_nt), the confidence score for that prediction, which was calculated using the synapse-level confusion metrics and with synapse filtering (conf_nt_p), see STAR Methods, neuron-level transmitter prediction with no synapse filtering for axon/dendrite (top_nt, top_nt_p), the proportion of synapse-level transmitter predictions that “voted" for each transmitter (transmitter), the segregation index, a measure of neuronal polarity^S2^ (segregation_index), other morphological annotations from Schlegel et al.^S2^ (side, ito_lee_hemilineage, artenstein_hemilineage, morphology_group, flow, cell_class, cell_sub_class), the proportion of synapse-level transmitter predictions that “voted” for each transmitter ({transmitter }), whether or not presynapses from this neuron were included in our ground-truth data (in_ground_truth) and extra notes on the cell type designation, including alternate name (notes). Note that hemilineage designation is given from two nomenclatures^S3–S6^ (ito_lee_hemilineage, hartenstein_hemilineage), and that ‘side’ refers to the hemisphere in which reconstructions’ cell bodies lie. Because the root_id for neurons is changing as neurons are edited in an active connectome project^S7^ we also supply the position of a point in the neuron to help identify it in FAFB-FlyWire voxel space (pos_x, pos_y, pos_z) and an ID for the nucleus segmentation (nucleus_id).

Data S5. A .zip archive containing .png files depicting each of the 183 brain hemilineages we have used from the FAFB-FlyWire dataset, related to Figure 7 Neurons in each hemilineage are colored by their neuron-level transmitter predictions, hemilineage names given in the file name. Hemilineage labels for the FAFB-FlyWire dataset are fully reported in Schlegel et al.^S2^

Data S6. A .csv containing summary results for our 183 central brain secondary hemilineages, related to Figure 7 Columns provide each hemilineage’s name (ito_lee_hemilineage),^S3,S6^ and lineage-associated tract (hartenstein_hemilineage),^S4,S5^ the number of neurons on the left and right hemispheres (left, right, center), the lineage type i.e., Type I or II (lineage_type), the number of neurons predicted for each transmitter ({transmitter }) and the majority vote of the hemilineage (majority_nt). The same information is given for HemiBrain hemilineages, with ‘hemibrain_’ added to the column name.

Data S7. A .csv in which each row is a described cell type of the fly brain, related to Figure 4 Cell type annotations were drawn from Schlegel et al.,^S2^ neurons with no cell type or hemibrain type annotated are not included. Columns provide: each cell type’s name (cell_type), predicted transmitter for the cell type (cell_type_nt), the cell type level transmitter confidence score (cell_type_confidence), the sum of neuron-level transmitter predictions across all FAFB-FlyWire reconstructions for each transmitter ({transmitter }_cell_type_{dataset}_confidence), the number of neurons with that confidence ({transmitter }_{dataset }_n), the transmitters reported to be used by the cell type in the literature (known_nt), the citations for that information (known_nt_source) and morphological annotations from Schlegel et al.^S2^ (ito_lee_hemilineage, hartenstein_hemilineage, morphology_group, flow, cell_class, cell_sub_class). The cell type level transmitter is determined by the highest {transmitter }_cell_type_{dataset}_confidence. The cell type level transmitter confidence score (cell_type_nt) was calculated using our prediction confusion matrices (see STAR Methods). Results for both FAFB-FlyWire (‘_fafb_’) and HemiBrain (‘_hemibrain_’) datasets are given.

Data S8. A .tar.gz archive containing .png files used to identify ultrastructural differences between synapses with different transmitters, related to Figure 3 Contains PNG files of 2D images cropped around synapses, together with their counterfactual translations into different neurotransmitter types and highlights of the most important areas for classification. There is one PNG file for each pair of neurotransmitters, named ‘source_target.png’. E.g., the file ‘ach_dop.png’ contains translations of real cholinergic synapses into counterfactual dopaminergic synapses. Each PNG shows images in six columns, which are: 1: original raw + classifier scores, 2: counterfactual raw + classifier scores, 3: counterfactual raw, with original raw in mask + classifier scores, 4: mask, shown on original + relative classifier change, 5: mask, shown on counterfactual, 6: difference in mask area.
