# Supplementary material for: Neurotransmitter classification from electron microscopy images at synaptic sites in Drosophila melanogaster
Source: Cell. 2024 May 9;187(10):2574–2594.e23. doi: 10.1016/j.cell.2024.03.016 (PMC11106717; doi:10.1016/j.cell.2024.03.016)
Supplement: Document S1. Supplemental references [file mmc1.pdf]

Cell, Volume *187*

Supplemental information

Neurotransmitter classification from electron microscopy images at synaptic sites in *Drosophila melanogaster*

Nils Eckstein, Alexander Shakeel Bates, Andrew Champion, Michelle Du, Yijie Yin, Philipp Schlegel, Alicia Kun-Yang Lu, Thomson Rymer, Samantha Finley-May, Tyler Paterson, Ruchi Parekh, Sven Dorkenwald, Arie Matsliah, Szi-Chieh Yu, Claire McKellar, Amy Sterling, Katharina Eichler, Marta Costa, Sebastian Seung, Mala Murthy, Volker Hartenstein, Gregory S.X.E. Jefferis, and Jan Funke

Supplemental References

S1. Schneider-Mizell, C. M., Gerhard, S., Longair, M., Kazimiers, T., Li, F., Zwart, M. F., Champion, A., Midgley, F. M., Fetter, R. D., Saalfeld, S., Cardona, A. (2016). Quantitative neuroanatomy for connectomics in drosophila. Elife, 5:e12059.

S2. Schlegel, P., Yin, Y., Bates, A. S., Dorkenwald, S., Eichler, K., Brooks, P., Han, D. S., Gkantia, M., dos Santos, M., Munnelly, E. J., et al (2023). A consensus cell type atlas from multiple connectomes reveals principles of circuit stereotypy and variation.Preprint at bioRxiv 10.1101/2023.06.27.546055

S3. Ito, M., Masuda, N., Shinomiya, K., Endo, K., and Ito, K. (2013). Systematic analysis of neural projections reveals clonal composition of the Drosophila brain. Curr. Biol., 23:644–655.

S4. Lovick, J. K., Ngo, K. T., Omoto, J. J., Wong, D. C., Nguyen, J. D., and Hartenstein, V. (2013). Postembryonic lineages of the Drosophila brain: I. development of the lineage-associated fiber tracts. Dev. Biol., 384:228–257.

S5. Wong, D. C., Lovick, J. K., Ngo, K. T., Borisuthirattana, W., Omoto, J. J., and Hartenstein, V. (2013). Postembryonic lineages of the Drosophila brain: II. identification of lineage projection patterns based on MARCM clones. Dev. Biol., 384:258–289.

S6. Yu, H.-H., Awasaki, T., Schroeder, M. D., Long, F., Yang, J. S., He, Y., Ding, P., Kao, J.-C., Wu, G. Y.-Y., Peng, H., et al (2013). Clonal development and organization of the adult Drosophila central brain. Curr. Biol., 23, 633, 643.

S7. Dorkenwald, S., McKellar, C. E., Macrina, T., Kemnitz, N., Lee, K., Lu, R., Wu, J., Popovych, S., Mitchell, E., Nehoran, B., et al (2022). FlyWire: online community for whole-brain connectomics. Nat. Methods, 19:119–128.
