# Supplementary figures and images for: Neurotransmitter classification from electron microscopy images at synaptic sites in Drosophila melanogaster (part 2 of 2)
Source: Cell. 2024 May 9;187(10):2574–2594.e23. doi: 10.1016/j.cell.2024.03.016 (PMC11106717; doi:10.1016/j.cell.2024.03.016)

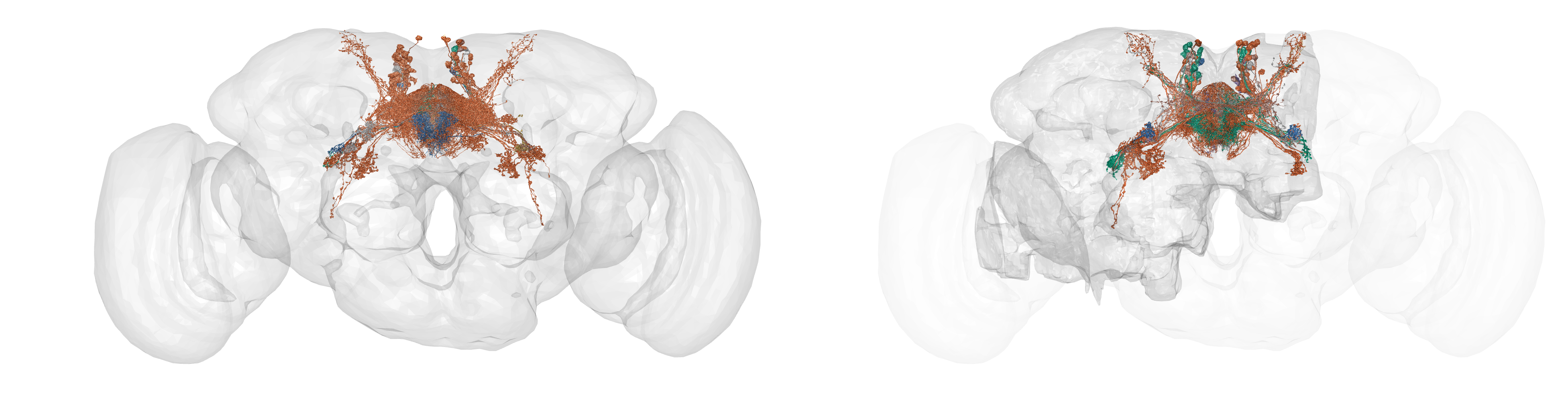

Supplement: Data S5. A .zip archive containing .png files depicting each of the 183 brain hemilineages we have used from the FAFB-FlyWire dataset, related to Figure 7 — Neurons in each hemilineage are colored by their neuron-level transmitter predictions, hemilineage names given in the file name. Hemilineage labels for the FAFB-FlyWire dataset are fully reported in Schlegel et al.S2 [file mmc6.zip › chosen_hemilineages/DM2_CX_d2__fafb_hemibrain.png]

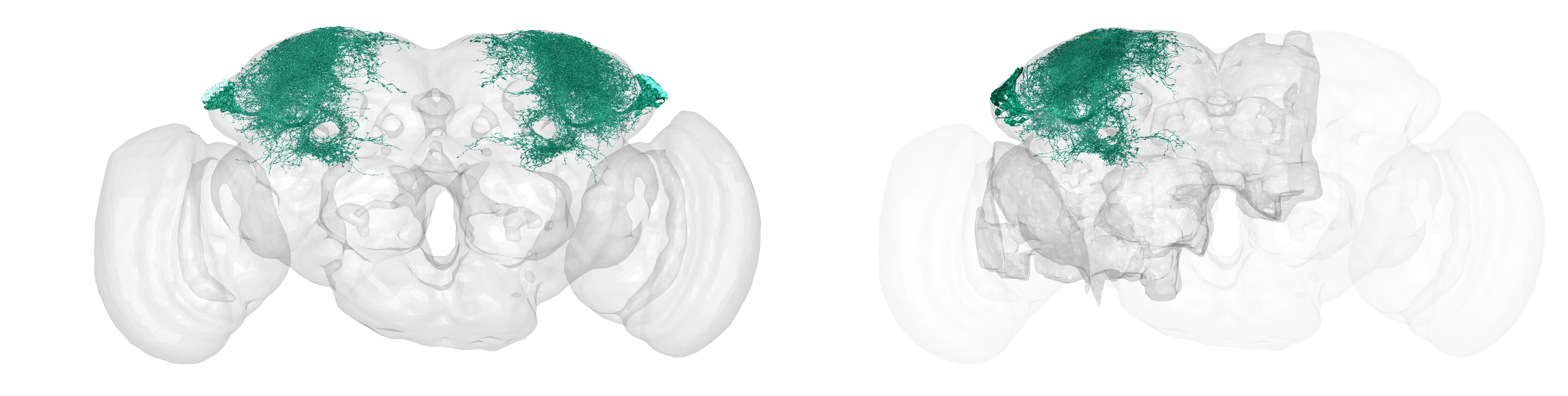

Supplement: Data S5. A .zip archive containing .png files depicting each of the 183 brain hemilineages we have used from the FAFB-FlyWire dataset, related to Figure 7 — Neurons in each hemilineage are colored by their neuron-level transmitter predictions, hemilineage names given in the file name. Hemilineage labels for the FAFB-FlyWire dataset are fully reported in Schlegel et al.S2 [file mmc6.zip › chosen_hemilineages/SLPal1__fafb_hemibrain.png]

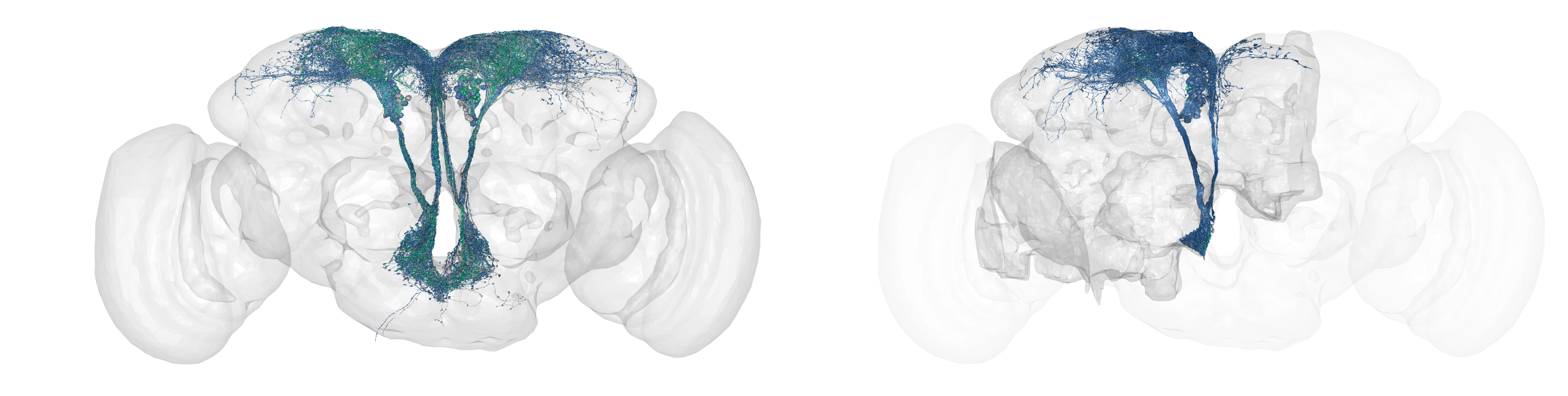

Supplement: Data S5. A .zip archive containing .png files depicting each of the 183 brain hemilineages we have used from the FAFB-FlyWire dataset, related to Figure 7 — Neurons in each hemilineage are colored by their neuron-level transmitter predictions, hemilineage names given in the file name. Hemilineage labels for the FAFB-FlyWire dataset are fully reported in Schlegel et al.S2 [file mmc6.zip › chosen_hemilineages/DM2_central__fafb_hemibrain.png]

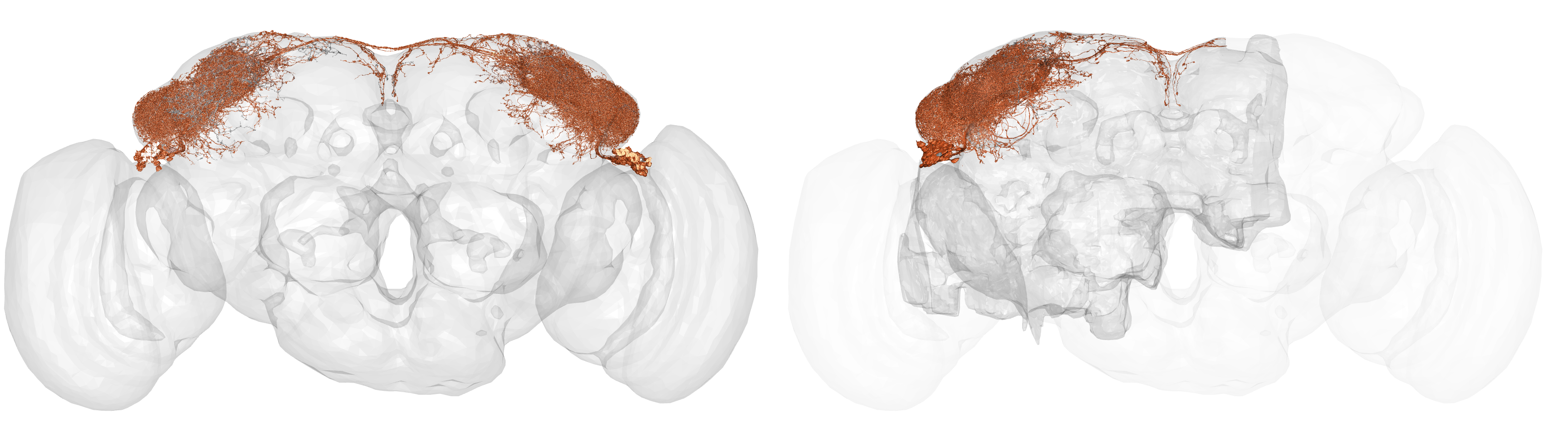

Supplement: Data S5. A .zip archive containing .png files depicting each of the 183 brain hemilineages we have used from the FAFB-FlyWire dataset, related to Figure 7 — Neurons in each hemilineage are colored by their neuron-level transmitter predictions, hemilineage names given in the file name. Hemilineage labels for the FAFB-FlyWire dataset are fully reported in Schlegel et al.S2 [file mmc6.zip › chosen_hemilineages/LHa1__fafb_hemibrain.png]

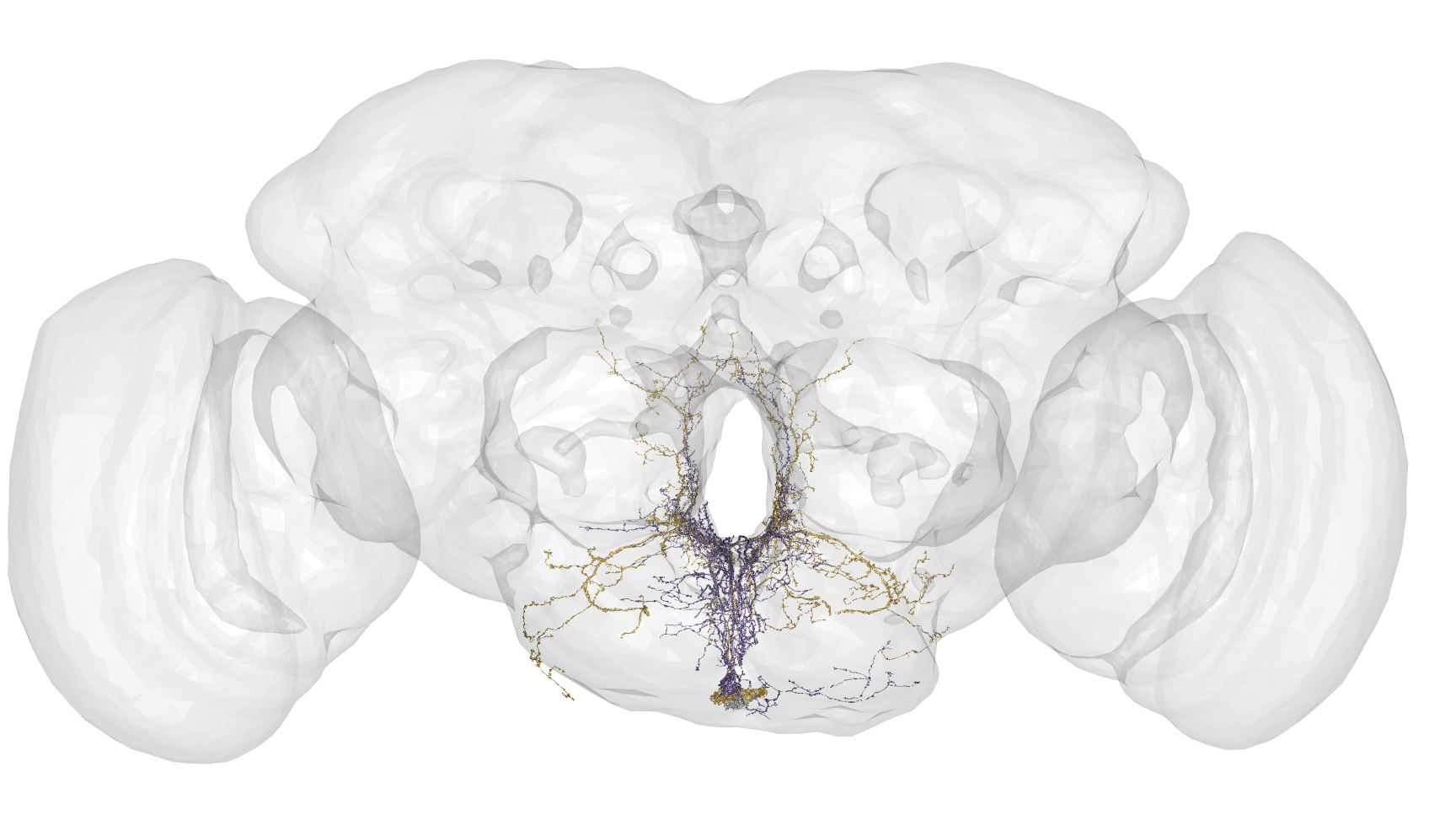

Supplement: Data S5. A .zip archive containing .png files depicting each of the 183 brain hemilineages we have used from the FAFB-FlyWire dataset, related to Figure 7 — Neurons in each hemilineage are colored by their neuron-level transmitter predictions, hemilineage names given in the file name. Hemilineage labels for the FAFB-FlyWire dataset are fully reported in Schlegel et al.S2 [file mmc6.zip › chosen_hemilineages/LB0_posterior__fafb.png]

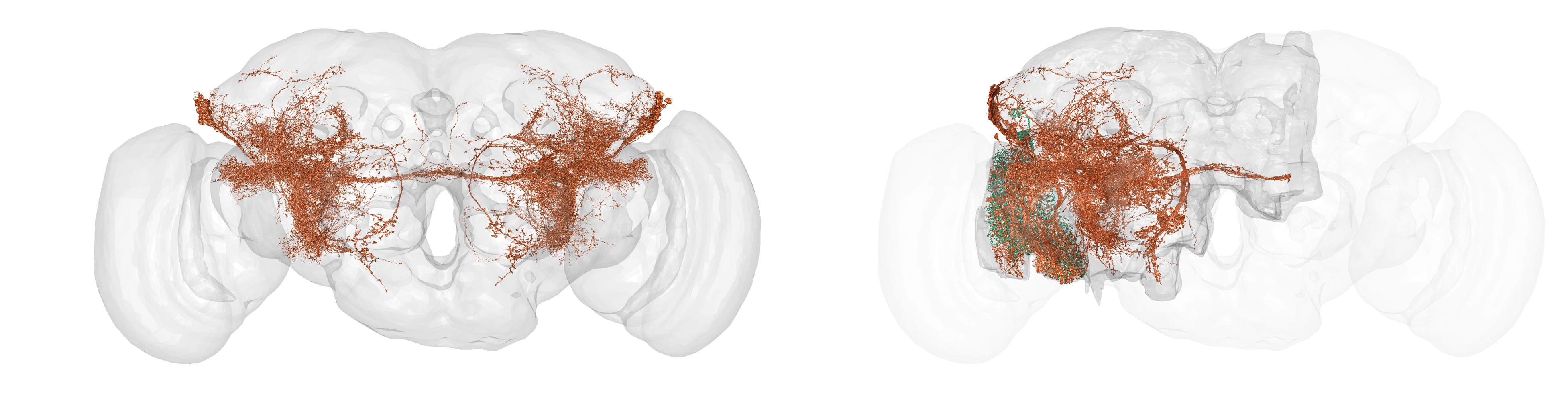

Supplement: Data S5. A .zip archive containing .png files depicting each of the 183 brain hemilineages we have used from the FAFB-FlyWire dataset, related to Figure 7 — Neurons in each hemilineage are colored by their neuron-level transmitter predictions, hemilineage names given in the file name. Hemilineage labels for the FAFB-FlyWire dataset are fully reported in Schlegel et al.S2 [file mmc6.zip › chosen_hemilineages/LHl4_posterior__fafb_hemibrain.png]

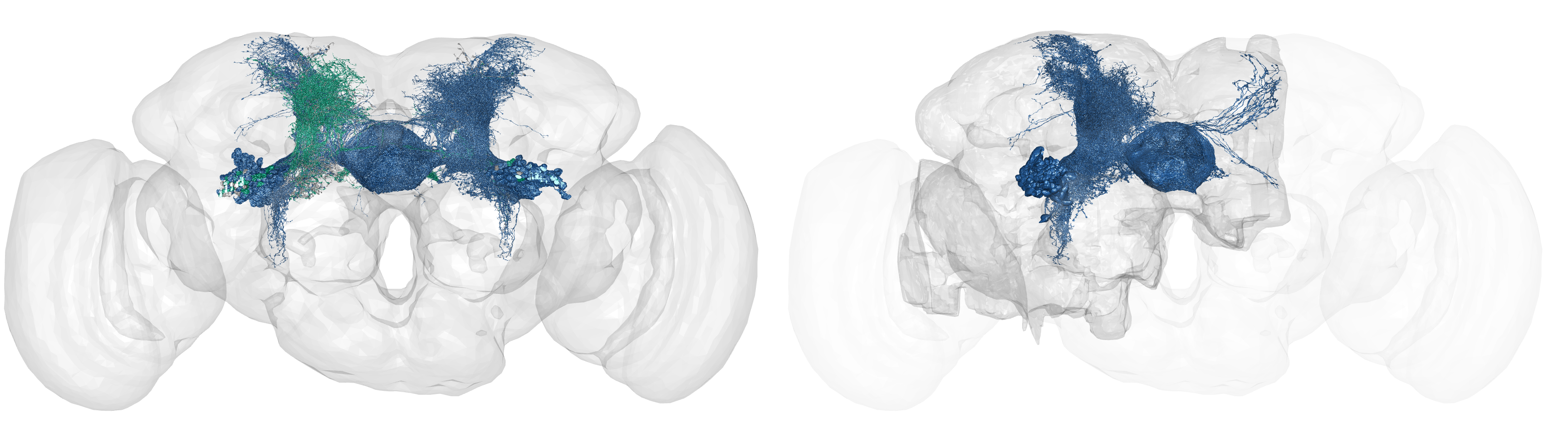

Supplement: Data S5. A .zip archive containing .png files depicting each of the 183 brain hemilineages we have used from the FAFB-FlyWire dataset, related to Figure 7 — Neurons in each hemilineage are colored by their neuron-level transmitter predictions, hemilineage names given in the file name. Hemilineage labels for the FAFB-FlyWire dataset are fully reported in Schlegel et al.S2 [file mmc6.zip › chosen_hemilineages/EBa1__fafb_hemibrain.png]

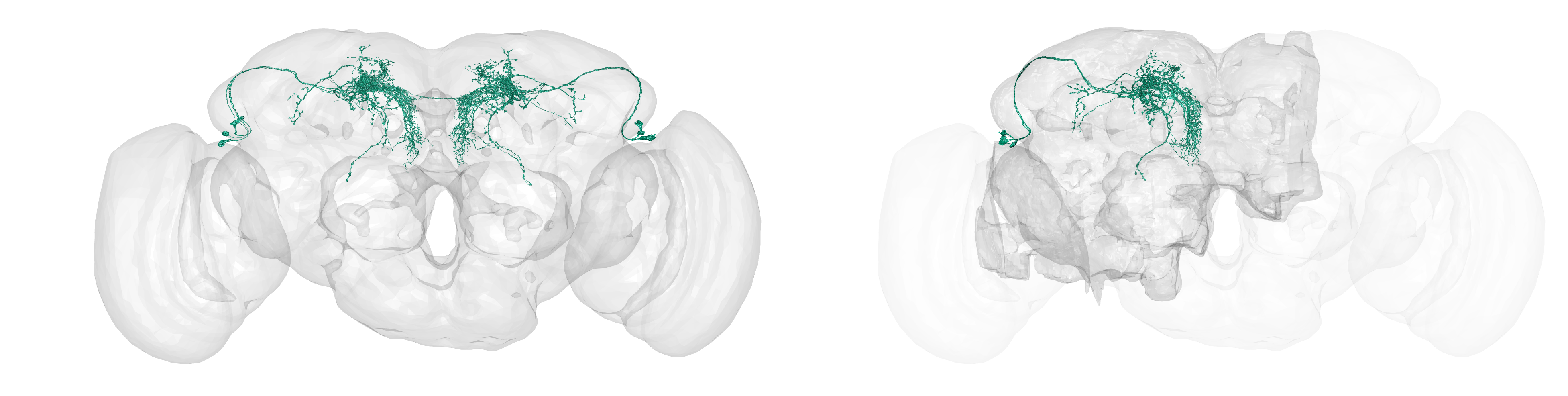

Supplement: Data S5. A .zip archive containing .png files depicting each of the 183 brain hemilineages we have used from the FAFB-FlyWire dataset, related to Figure 7 — Neurons in each hemilineage are colored by their neuron-level transmitter predictions, hemilineage names given in the file name. Hemilineage labels for the FAFB-FlyWire dataset are fully reported in Schlegel et al.S2 [file mmc6.zip › chosen_hemilineages/SIPa1_dorsal__fafb_hemibrain.png]

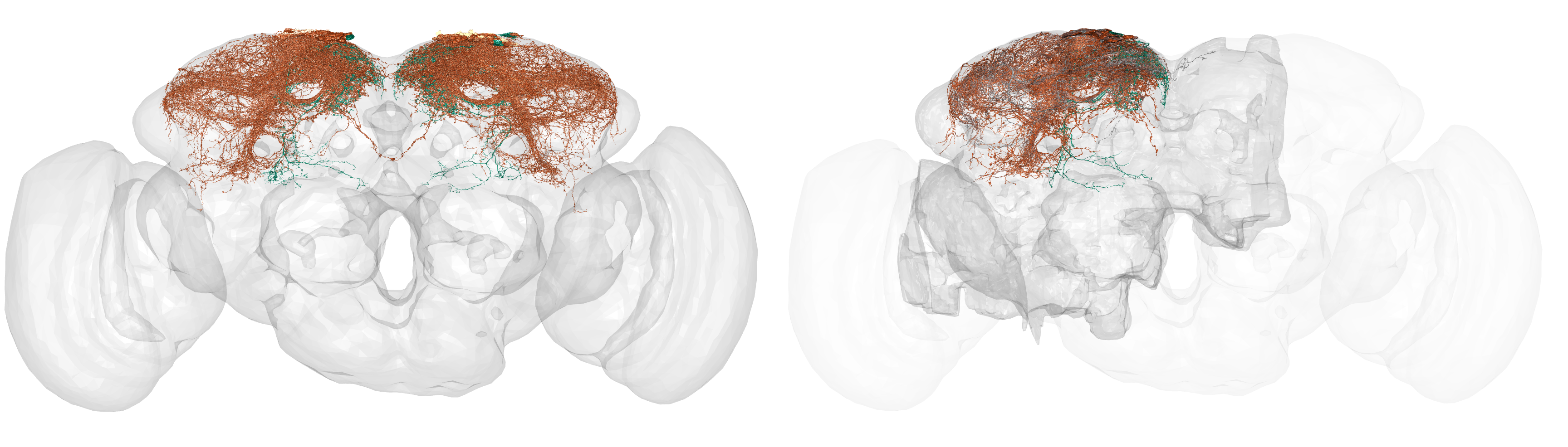

Supplement: Data S5. A .zip archive containing .png files depicting each of the 183 brain hemilineages we have used from the FAFB-FlyWire dataset, related to Figure 7 — Neurons in each hemilineage are colored by their neuron-level transmitter predictions, hemilineage names given in the file name. Hemilineage labels for the FAFB-FlyWire dataset are fully reported in Schlegel et al.S2 [file mmc6.zip › chosen_hemilineages/SMPpd1__fafb_hemibrain.png]

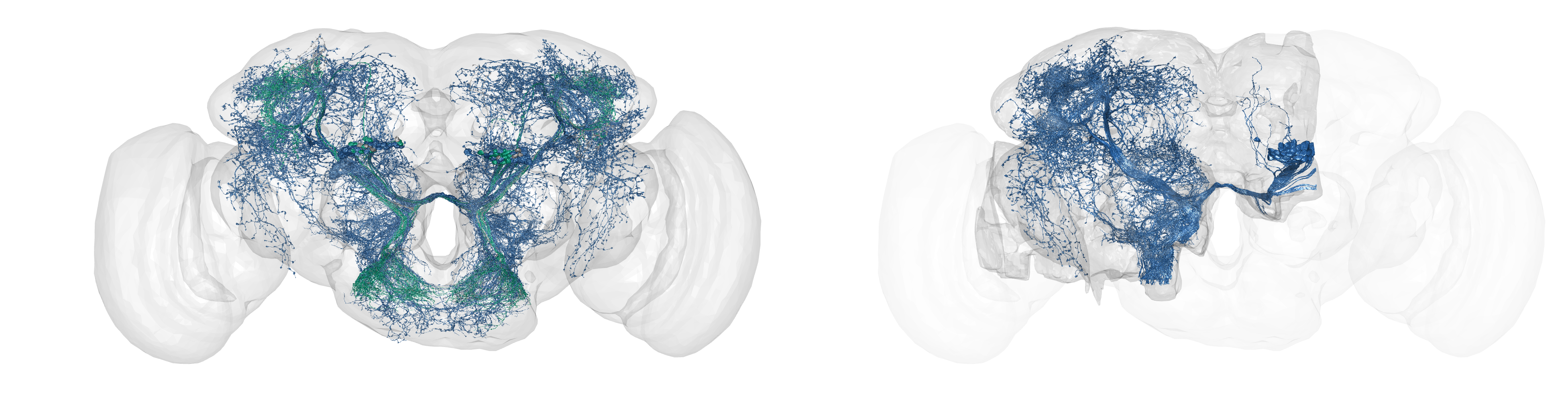

Supplement: Data S5. A .zip archive containing .png files depicting each of the 183 brain hemilineages we have used from the FAFB-FlyWire dataset, related to Figure 7 — Neurons in each hemilineage are colored by their neuron-level transmitter predictions, hemilineage names given in the file name. Hemilineage labels for the FAFB-FlyWire dataset are fully reported in Schlegel et al.S2 [file mmc6.zip › chosen_hemilineages/CREa1_ventral__fafb_hemibrain.png]

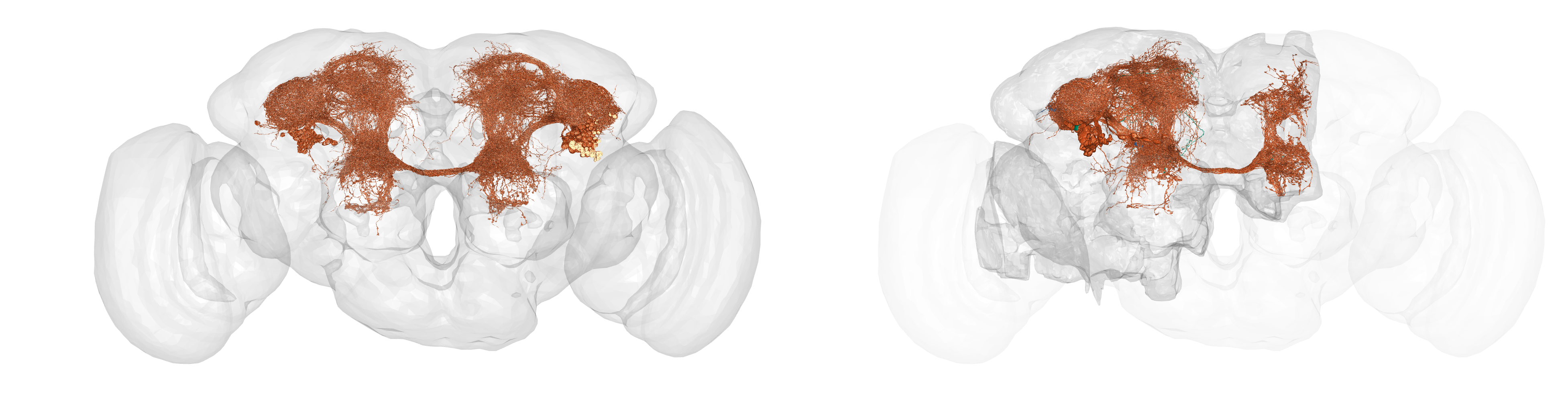

Supplement: Data S5. A .zip archive containing .png files depicting each of the 183 brain hemilineages we have used from the FAFB-FlyWire dataset, related to Figure 7 — Neurons in each hemilineage are colored by their neuron-level transmitter predictions, hemilineage names given in the file name. Hemilineage labels for the FAFB-FlyWire dataset are fully reported in Schlegel et al.S2 [file mmc6.zip › chosen_hemilineages/AOTUv4_dorsal__fafb_hemibrain.png]

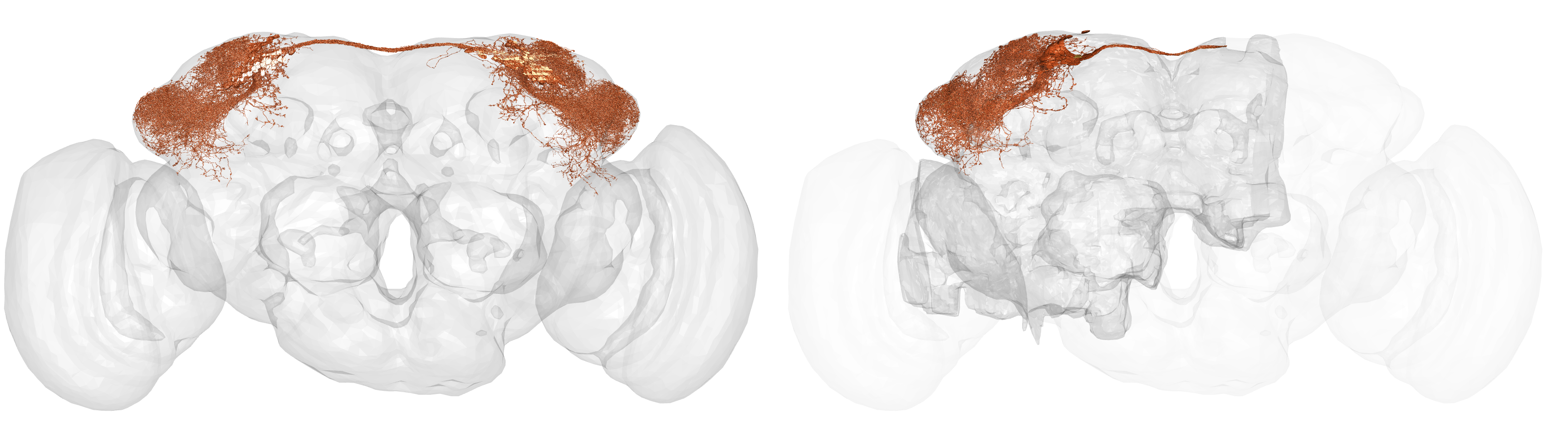

Supplement: Data S5. A .zip archive containing .png files depicting each of the 183 brain hemilineages we have used from the FAFB-FlyWire dataset, related to Figure 7 — Neurons in each hemilineage are colored by their neuron-level transmitter predictions, hemilineage names given in the file name. Hemilineage labels for the FAFB-FlyWire dataset are fully reported in Schlegel et al.S2 [file mmc6.zip › chosen_hemilineages/LHd1__fafb_hemibrain.png]

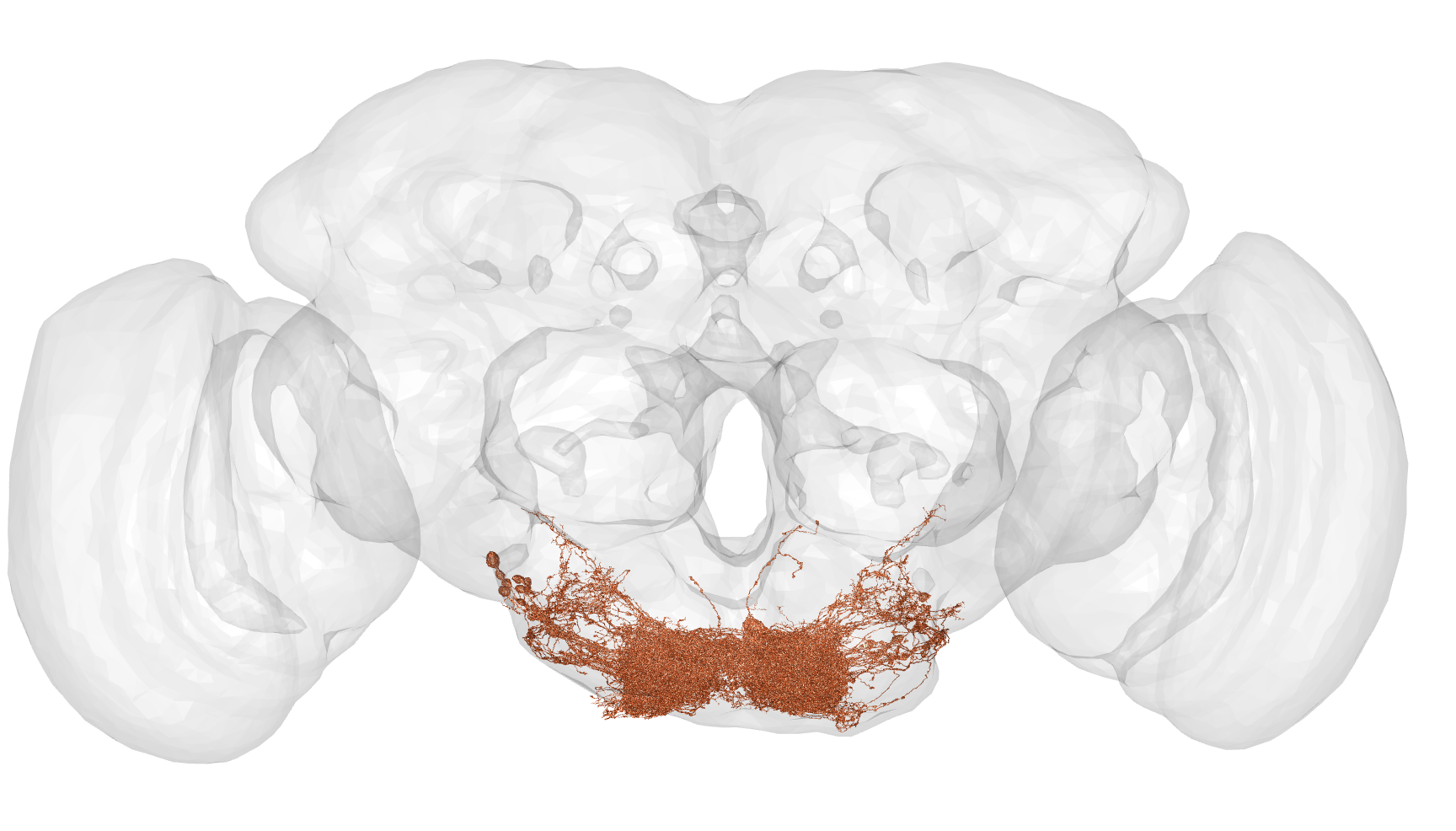

Supplement: Data S5. A .zip archive containing .png files depicting each of the 183 brain hemilineages we have used from the FAFB-FlyWire dataset, related to Figure 7 — Neurons in each hemilineage are colored by their neuron-level transmitter predictions, hemilineage names given in the file name. Hemilineage labels for the FAFB-FlyWire dataset are fully reported in Schlegel et al.S2 [file mmc6.zip › chosen_hemilineages/LB23__fafb.png]

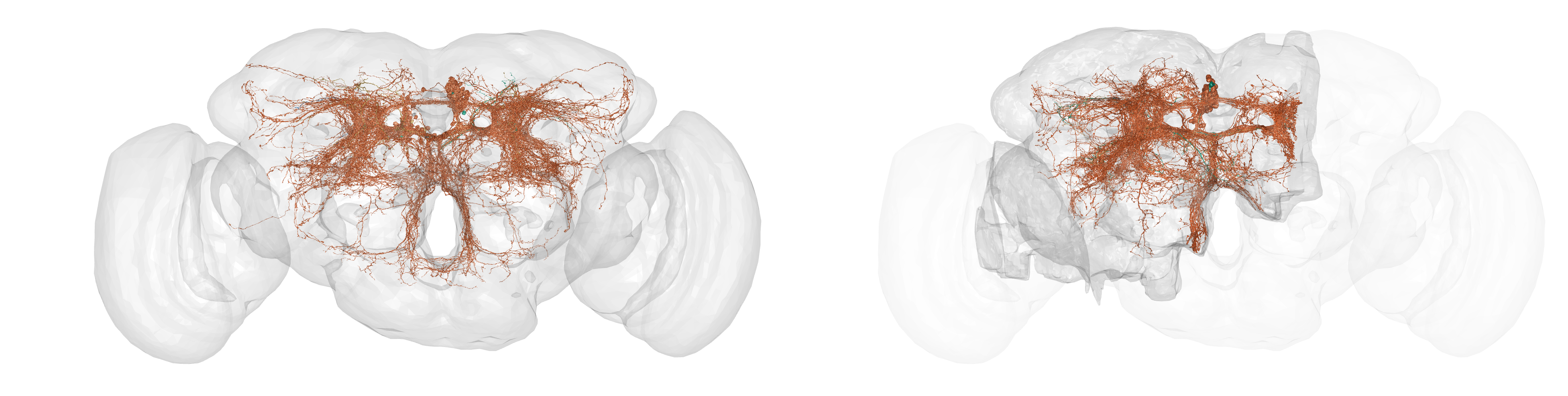

Supplement: Data S5. A .zip archive containing .png files depicting each of the 183 brain hemilineages we have used from the FAFB-FlyWire dataset, related to Figure 7 — Neurons in each hemilineage are colored by their neuron-level transmitter predictions, hemilineage names given in the file name. Hemilineage labels for the FAFB-FlyWire dataset are fully reported in Schlegel et al.S2 [file mmc6.zip › chosen_hemilineages/DM1_dorsal__fafb_hemibrain.png]

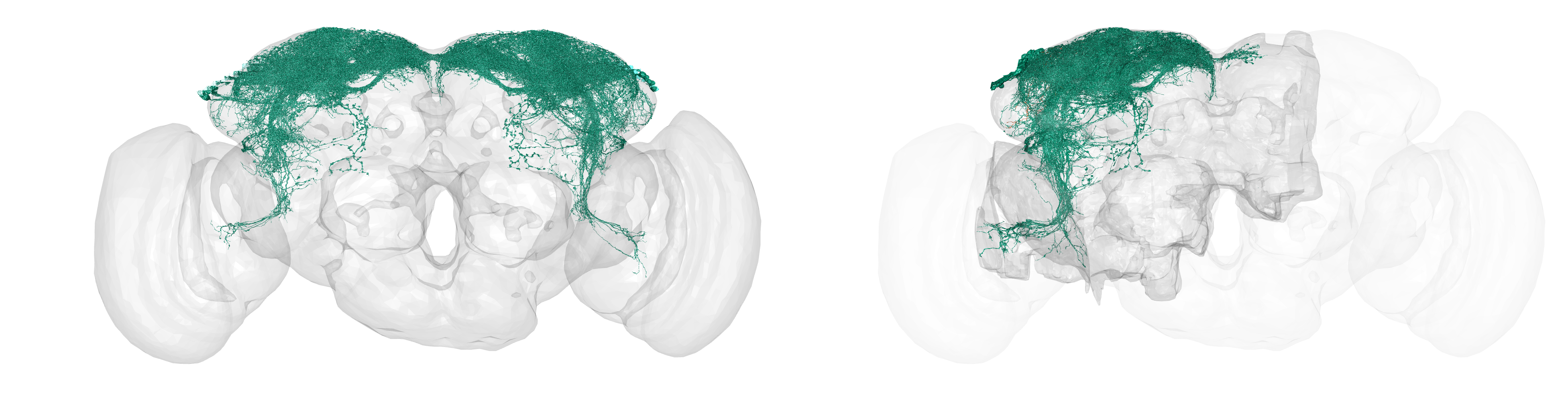

Supplement: Data S5. A .zip archive containing .png files depicting each of the 183 brain hemilineages we have used from the FAFB-FlyWire dataset, related to Figure 7 — Neurons in each hemilineage are colored by their neuron-level transmitter predictions, hemilineage names given in the file name. Hemilineage labels for the FAFB-FlyWire dataset are fully reported in Schlegel et al.S2 [file mmc6.zip › chosen_hemilineages/SLPpl1__fafb_hemibrain.png]

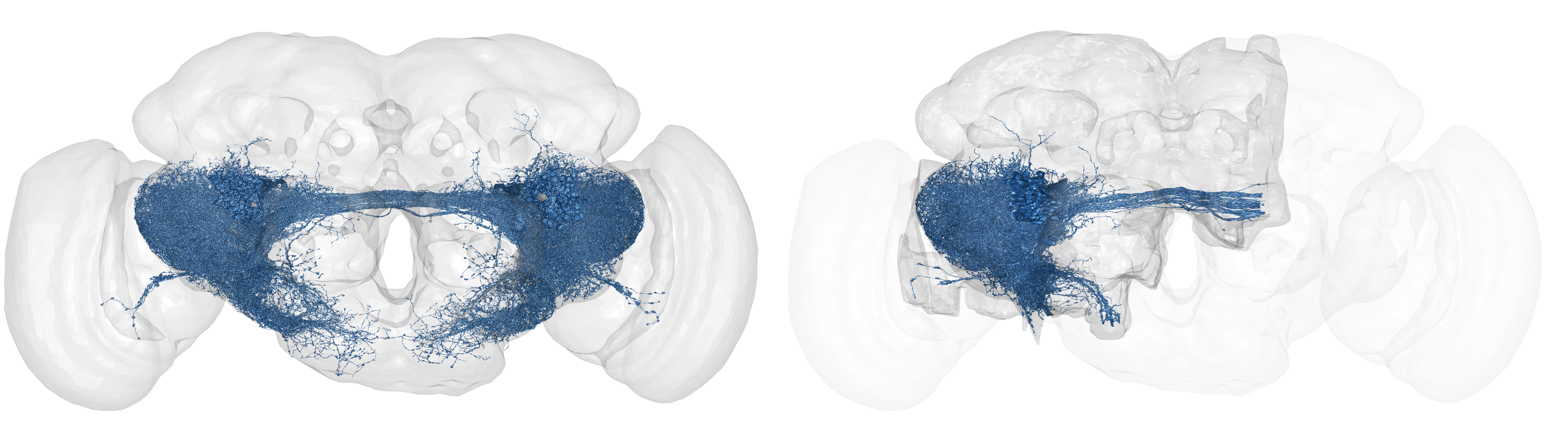

Supplement: Data S5. A .zip archive containing .png files depicting each of the 183 brain hemilineages we have used from the FAFB-FlyWire dataset, related to Figure 7 — Neurons in each hemilineage are colored by their neuron-level transmitter predictions, hemilineage names given in the file name. Hemilineage labels for the FAFB-FlyWire dataset are fully reported in Schlegel et al.S2 [file mmc6.zip › chosen_hemilineages/VLPa2__fafb_hemibrain.png]

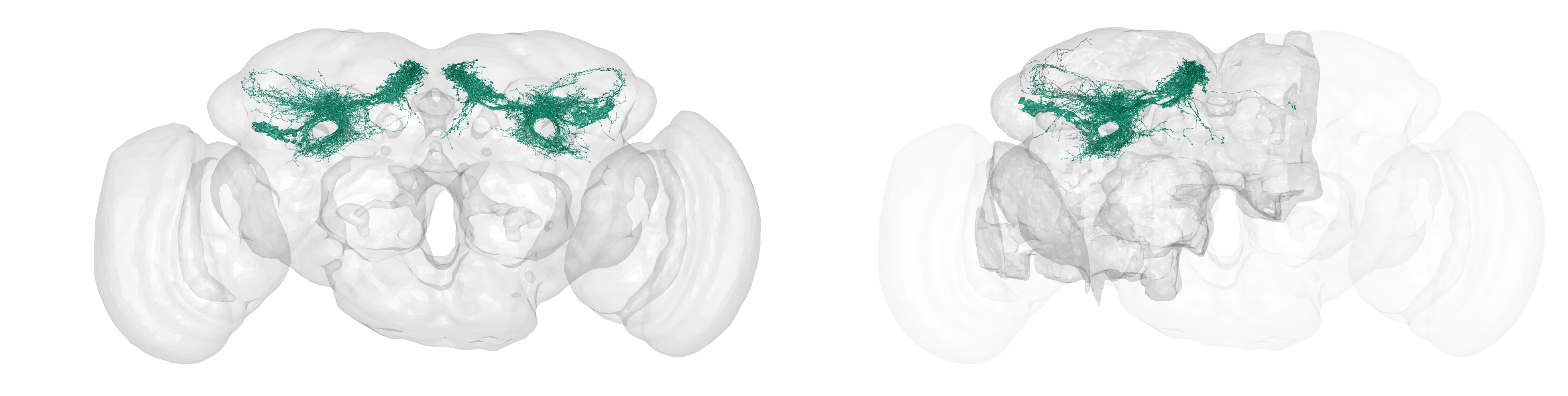

Supplement: Data S5. A .zip archive containing .png files depicting each of the 183 brain hemilineages we have used from the FAFB-FlyWire dataset, related to Figure 7 — Neurons in each hemilineage are colored by their neuron-level transmitter predictions, hemilineage names given in the file name. Hemilineage labels for the FAFB-FlyWire dataset are fully reported in Schlegel et al.S2 [file mmc6.zip › chosen_hemilineages/CLp2__fafb_hemibrain.png]

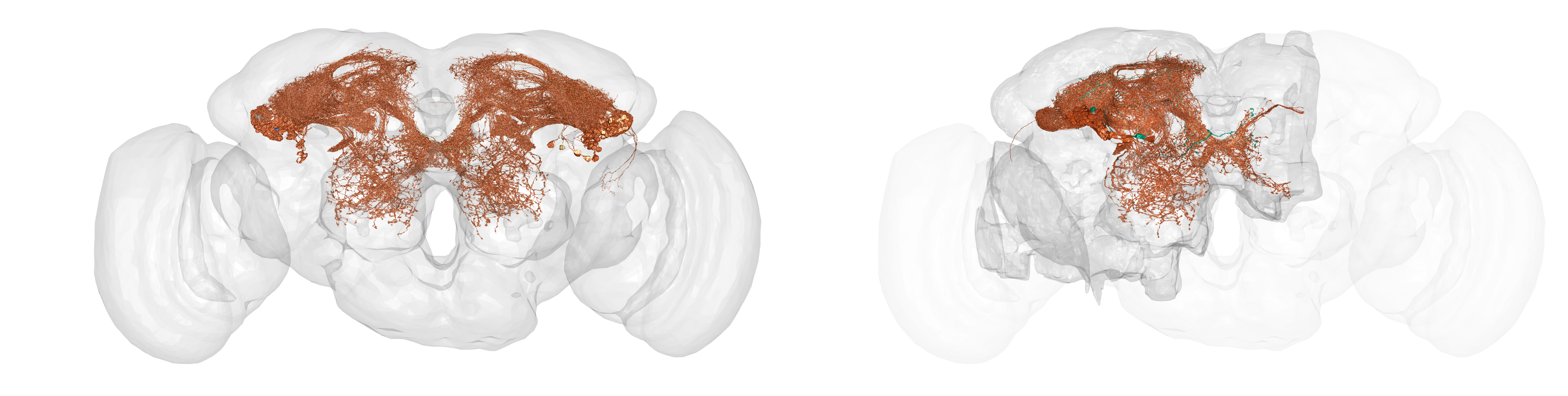

Supplement: Data S5. A .zip archive containing .png files depicting each of the 183 brain hemilineages we have used from the FAFB-FlyWire dataset, related to Figure 7 — Neurons in each hemilineage are colored by their neuron-level transmitter predictions, hemilineage names given in the file name. Hemilineage labels for the FAFB-FlyWire dataset are fully reported in Schlegel et al.S2 [file mmc6.zip › chosen_hemilineages/AOTUv3_dorsal__fafb_hemibrain.png]

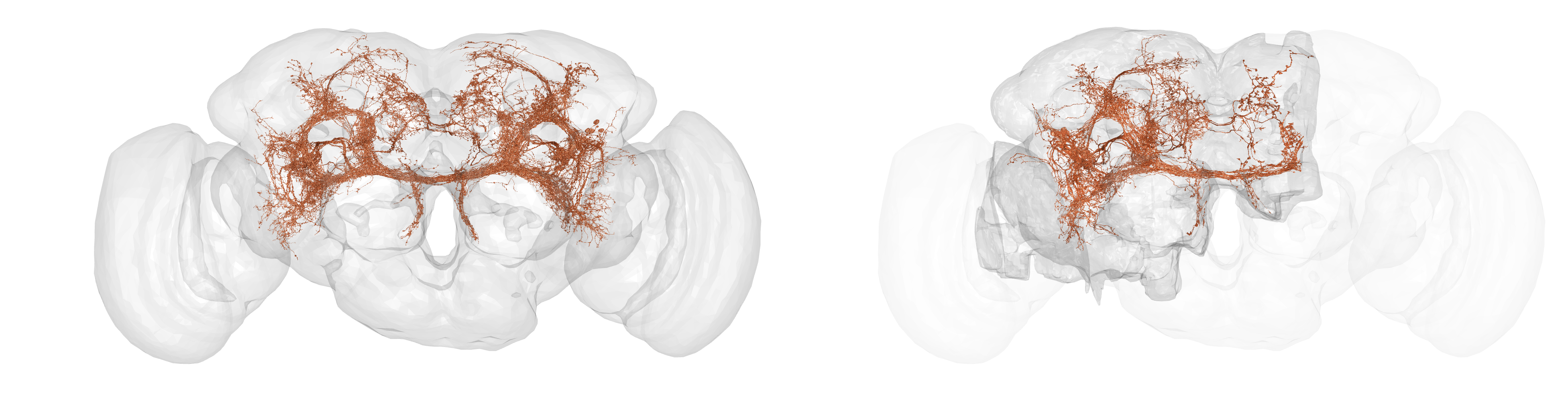

Supplement: Data S5. A .zip archive containing .png files depicting each of the 183 brain hemilineages we have used from the FAFB-FlyWire dataset, related to Figure 7 — Neurons in each hemilineage are colored by their neuron-level transmitter predictions, hemilineage names given in the file name. Hemilineage labels for the FAFB-FlyWire dataset are fully reported in Schlegel et al.S2 [file mmc6.zip › chosen_hemilineages/DL2_ventral__fafb_hemibrain.png]

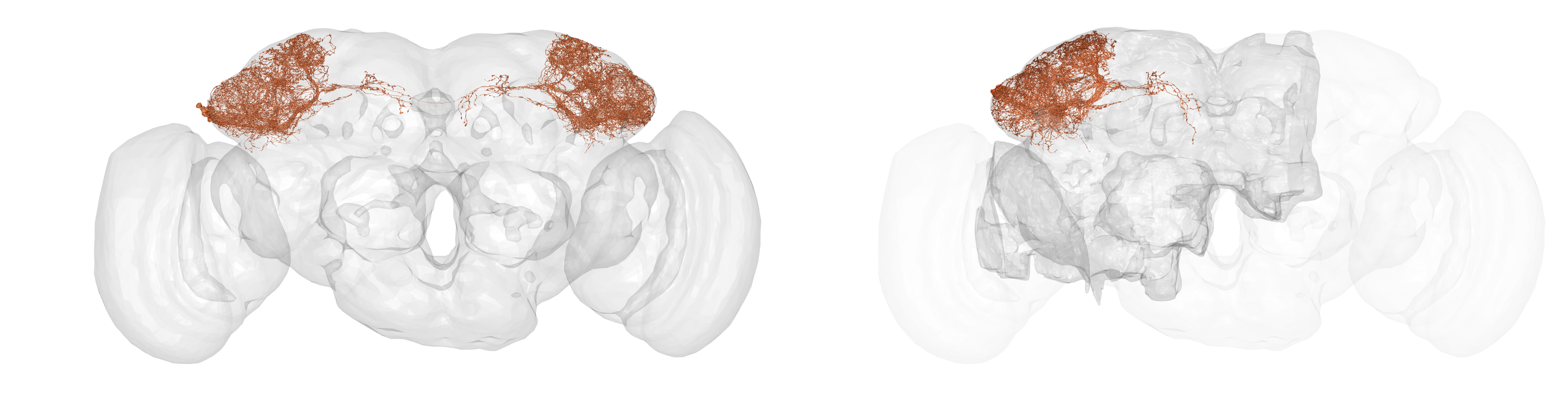

Supplement: Data S5. A .zip archive containing .png files depicting each of the 183 brain hemilineages we have used from the FAFB-FlyWire dataset, related to Figure 7 — Neurons in each hemilineage are colored by their neuron-level transmitter predictions, hemilineage names given in the file name. Hemilineage labels for the FAFB-FlyWire dataset are fully reported in Schlegel et al.S2 [file mmc6.zip › chosen_hemilineages/LHp3__fafb_hemibrain.png]

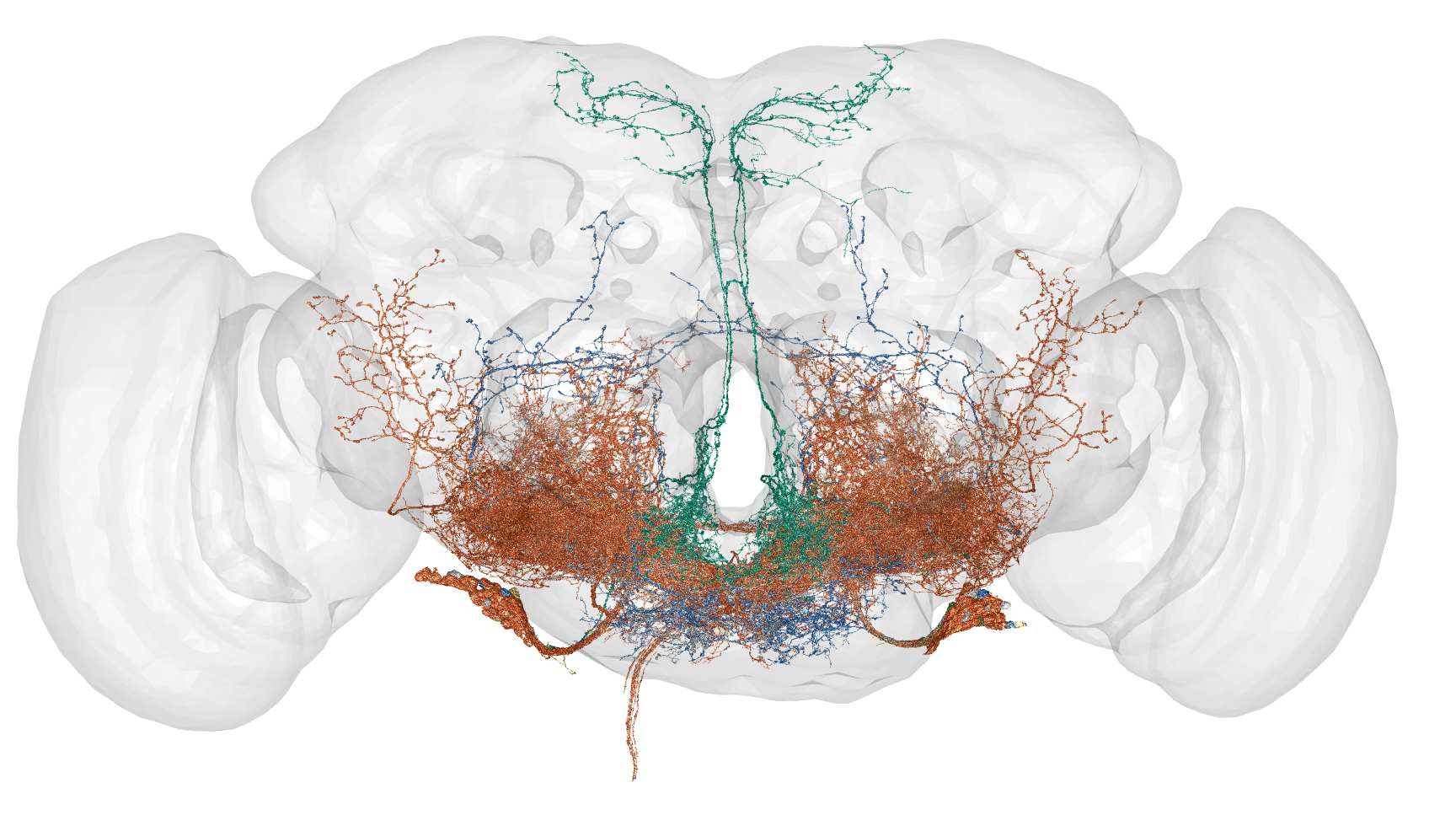

Supplement: Data S5. A .zip archive containing .png files depicting each of the 183 brain hemilineages we have used from the FAFB-FlyWire dataset, related to Figure 7 — Neurons in each hemilineage are colored by their neuron-level transmitter predictions, hemilineage names given in the file name. Hemilineage labels for the FAFB-FlyWire dataset are fully reported in Schlegel et al.S2 [file mmc6.zip › chosen_hemilineages/LB7__fafb.png]

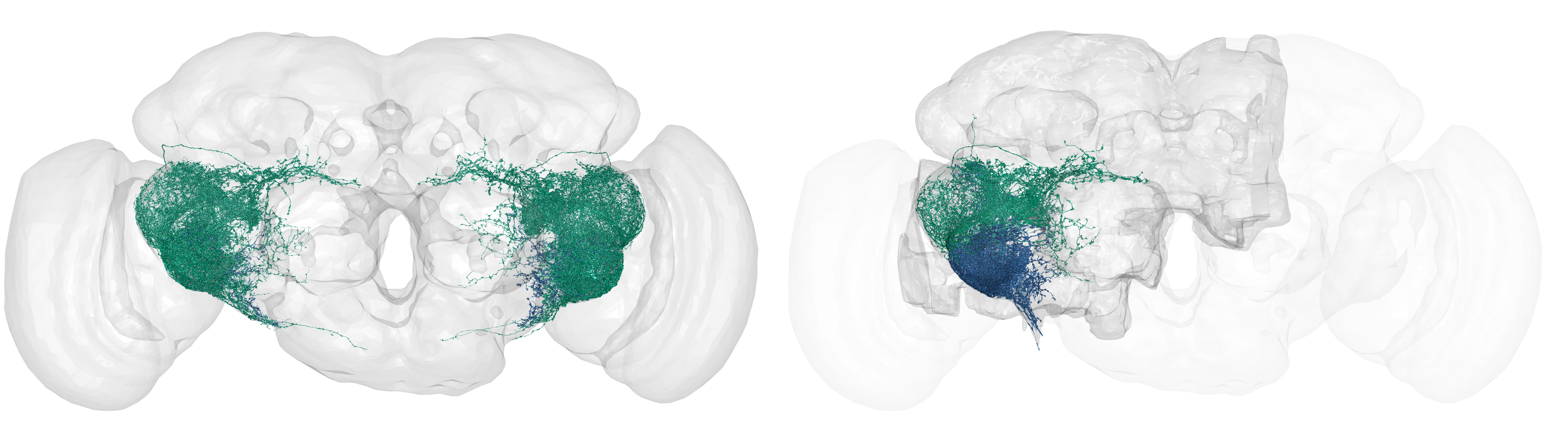

Supplement: Data S5. A .zip archive containing .png files depicting each of the 183 brain hemilineages we have used from the FAFB-FlyWire dataset, related to Figure 7 — Neurons in each hemilineage are colored by their neuron-level transmitter predictions, hemilineage names given in the file name. Hemilineage labels for the FAFB-FlyWire dataset are fully reported in Schlegel et al.S2 [file mmc6.zip › chosen_hemilineages/VLPp1__fafb_hemibrain.png]

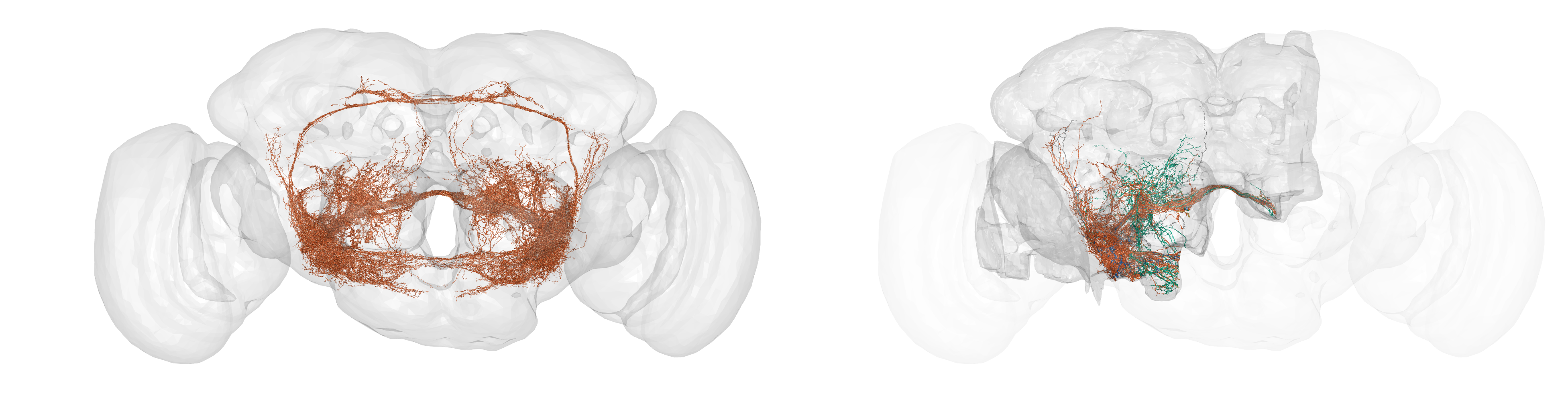

Supplement: Data S5. A .zip archive containing .png files depicting each of the 183 brain hemilineages we have used from the FAFB-FlyWire dataset, related to Figure 7 — Neurons in each hemilineage are colored by their neuron-level transmitter predictions, hemilineage names given in the file name. Hemilineage labels for the FAFB-FlyWire dataset are fully reported in Schlegel et al.S2 [file mmc6.zip › chosen_hemilineages/DM4_ventral__fafb_hemibrain.png]

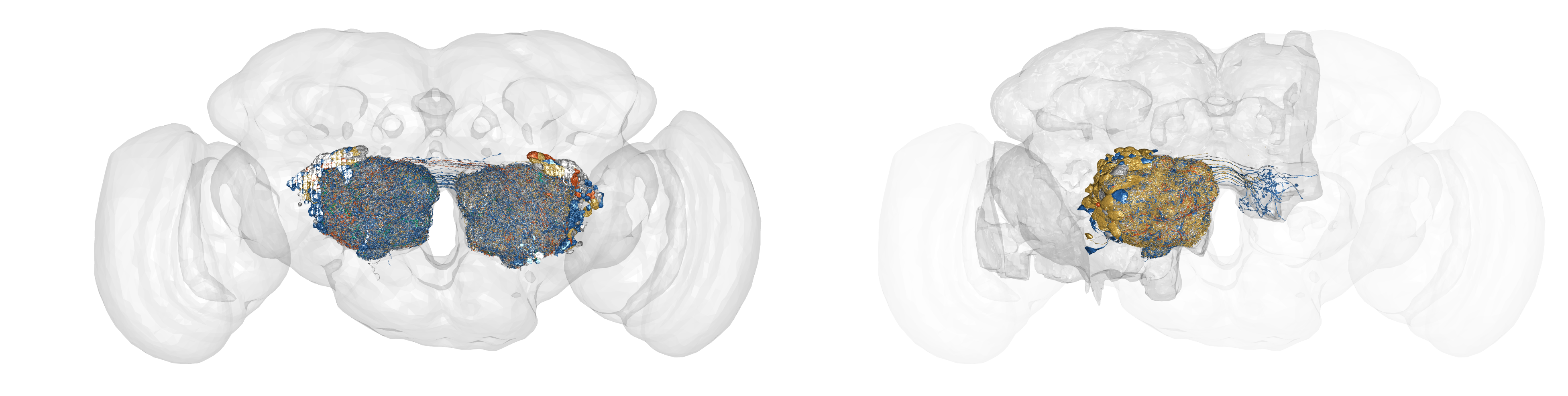

Supplement: Data S5. A .zip archive containing .png files depicting each of the 183 brain hemilineages we have used from the FAFB-FlyWire dataset, related to Figure 7 — Neurons in each hemilineage are colored by their neuron-level transmitter predictions, hemilineage names given in the file name. Hemilineage labels for the FAFB-FlyWire dataset are fully reported in Schlegel et al.S2 [file mmc6.zip › chosen_hemilineages/ALl1_dorsal__fafb_hemibrain.png]

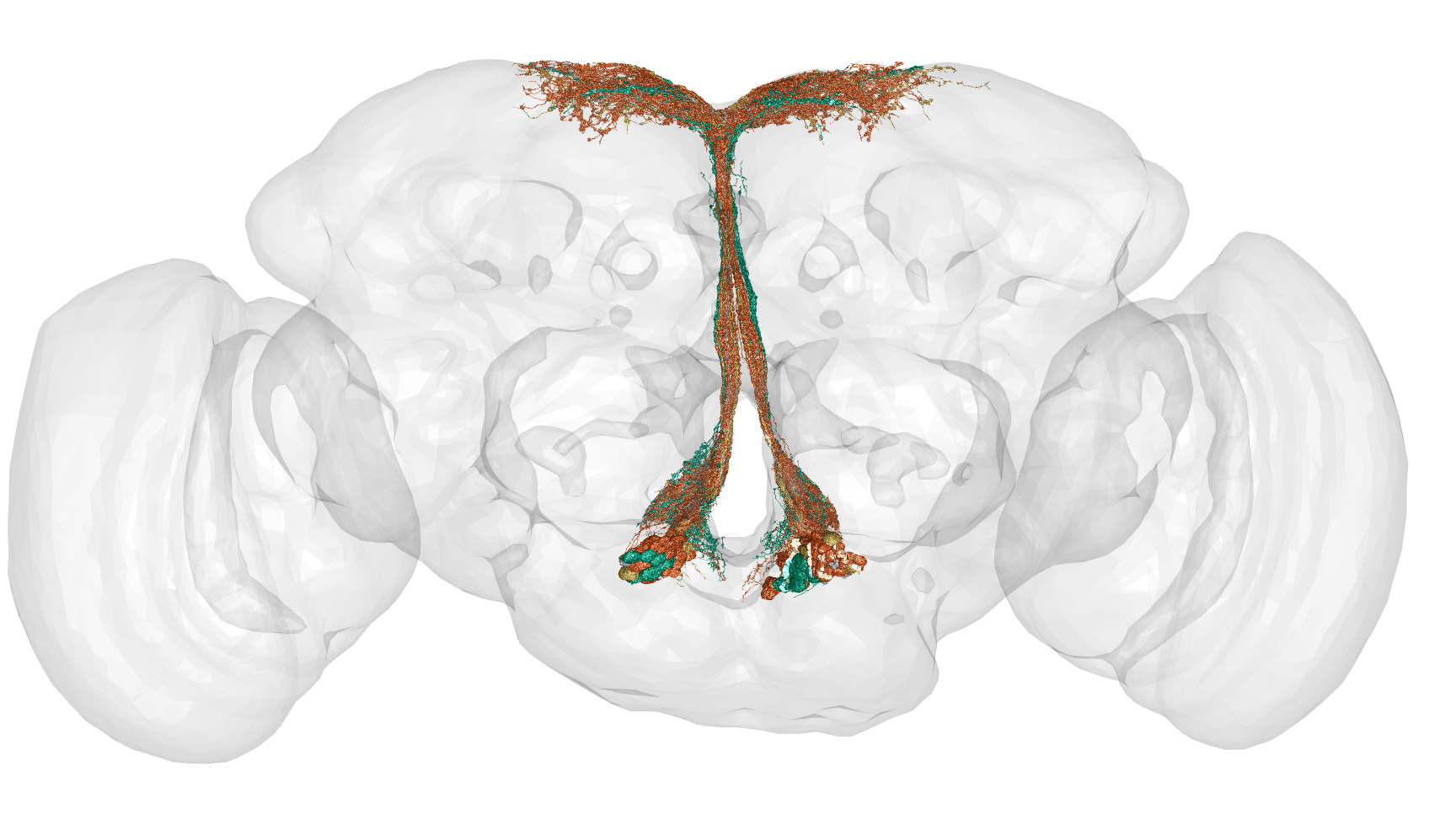

Supplement: Data S5. A .zip archive containing .png files depicting each of the 183 brain hemilineages we have used from the FAFB-FlyWire dataset, related to Figure 7 — Neurons in each hemilineage are colored by their neuron-level transmitter predictions, hemilineage names given in the file name. Hemilineage labels for the FAFB-FlyWire dataset are fully reported in Schlegel et al.S2 [file mmc6.zip › chosen_hemilineages/FLAa3__fafb.png]

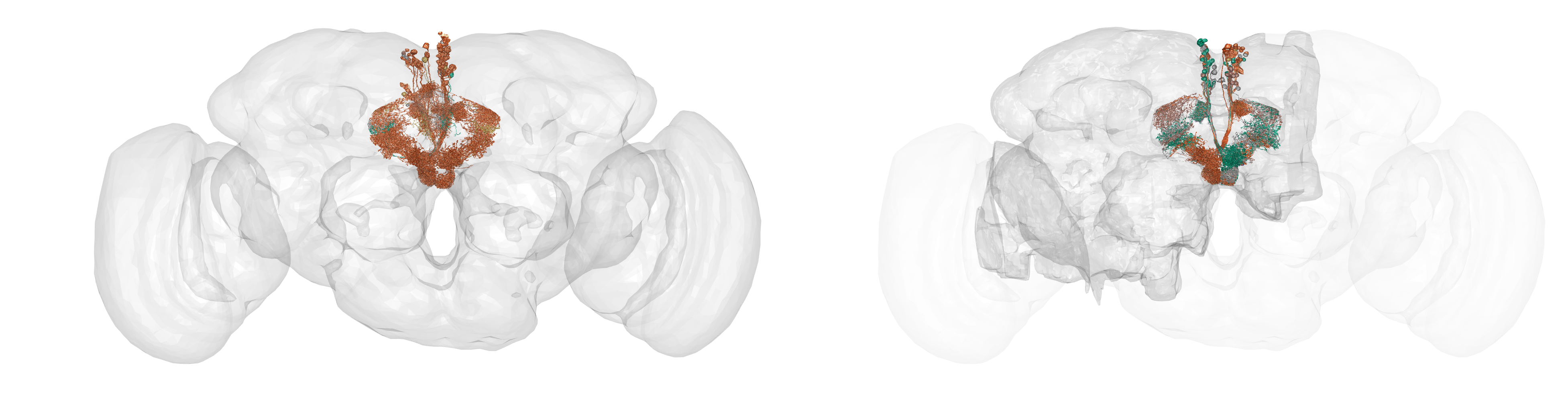

Supplement: Data S5. A .zip archive containing .png files depicting each of the 183 brain hemilineages we have used from the FAFB-FlyWire dataset, related to Figure 7 — Neurons in each hemilineage are colored by their neuron-level transmitter predictions, hemilineage names given in the file name. Hemilineage labels for the FAFB-FlyWire dataset are fully reported in Schlegel et al.S2 [file mmc6.zip › chosen_hemilineages/DM1_CX_v__fafb_hemibrain.png]

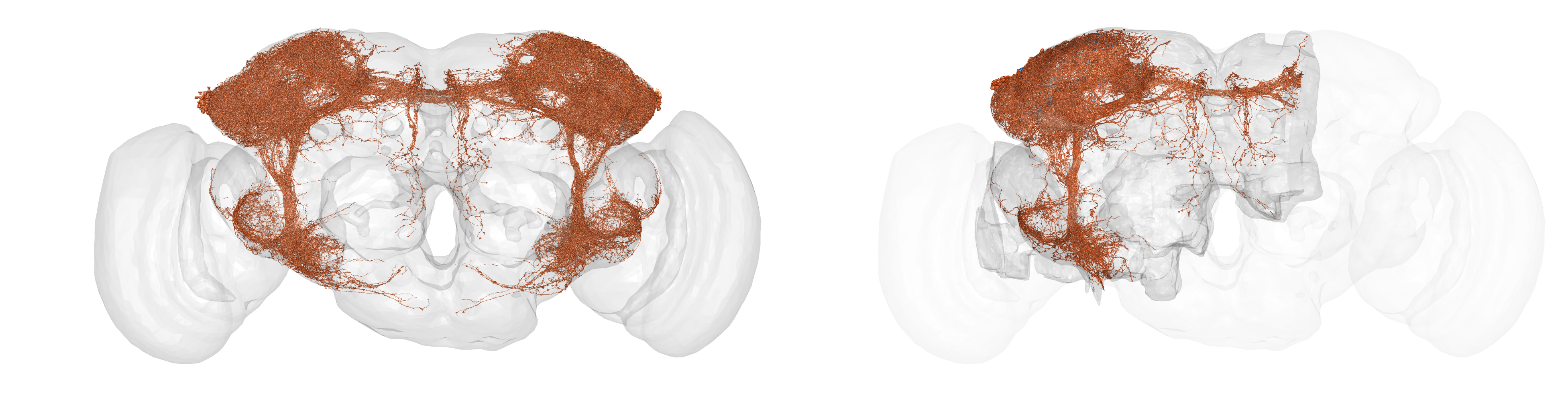

Supplement: Data S5. A .zip archive containing .png files depicting each of the 183 brain hemilineages we have used from the FAFB-FlyWire dataset, related to Figure 7 — Neurons in each hemilineage are colored by their neuron-level transmitter predictions, hemilineage names given in the file name. Hemilineage labels for the FAFB-FlyWire dataset are fully reported in Schlegel et al.S2 [file mmc6.zip › chosen_hemilineages/LHp2__fafb_hemibrain.png]

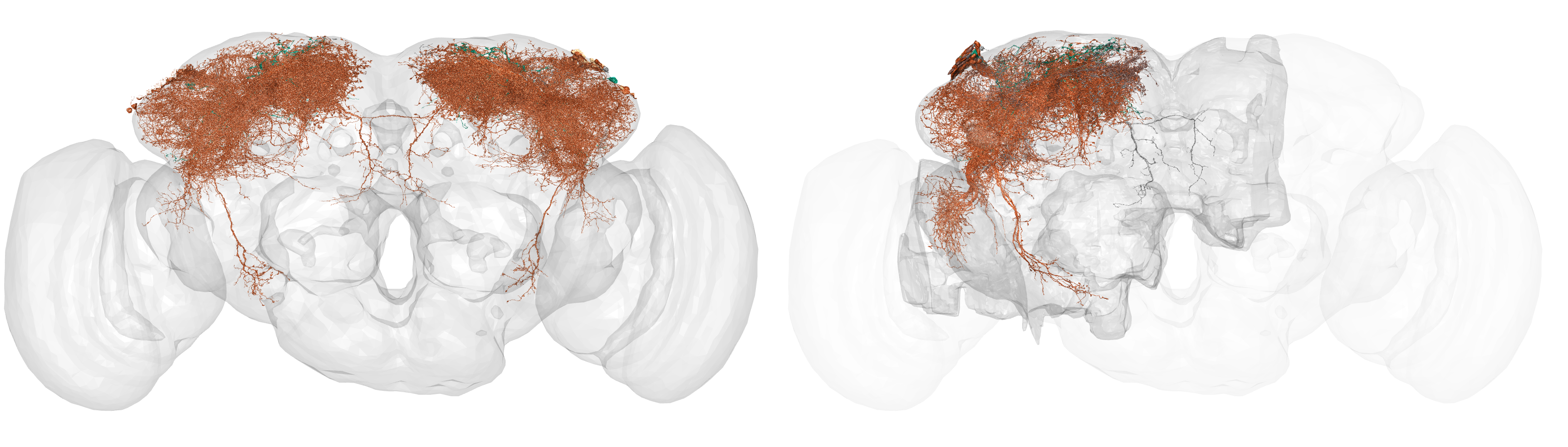

Supplement: Data S5. A .zip archive containing .png files depicting each of the 183 brain hemilineages we have used from the FAFB-FlyWire dataset, related to Figure 7 — Neurons in each hemilineage are colored by their neuron-level transmitter predictions, hemilineage names given in the file name. Hemilineage labels for the FAFB-FlyWire dataset are fully reported in Schlegel et al.S2 [file mmc6.zip › chosen_hemilineages/VLPd&p1_posterior__fafb_hemibrain.png]

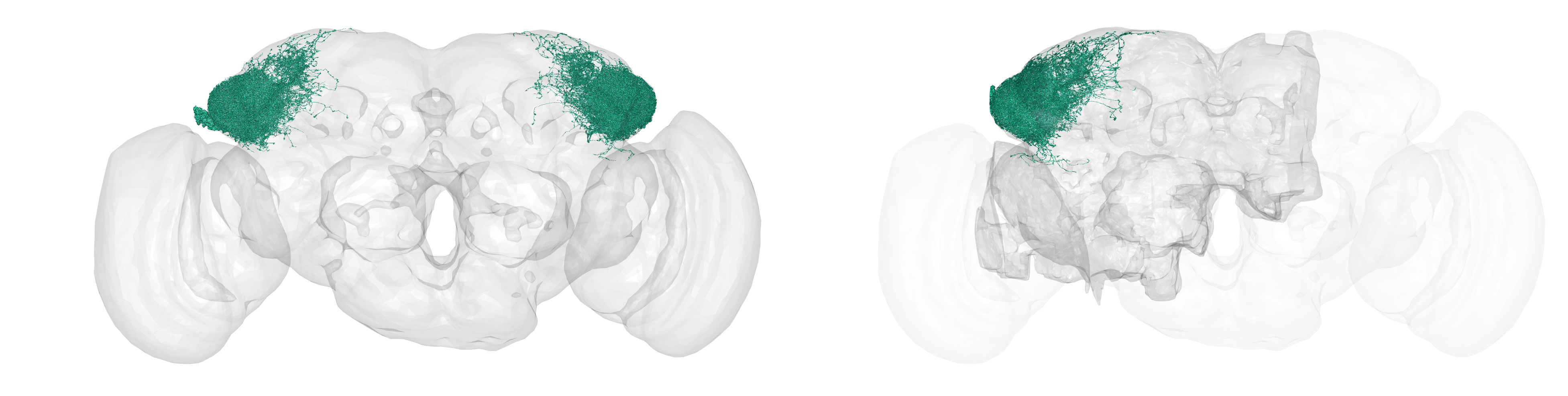

Supplement: Data S5. A .zip archive containing .png files depicting each of the 183 brain hemilineages we have used from the FAFB-FlyWire dataset, related to Figure 7 — Neurons in each hemilineage are colored by their neuron-level transmitter predictions, hemilineage names given in the file name. Hemilineage labels for the FAFB-FlyWire dataset are fully reported in Schlegel et al.S2 [file mmc6.zip › chosen_hemilineages/LHp1__fafb_hemibrain.png]

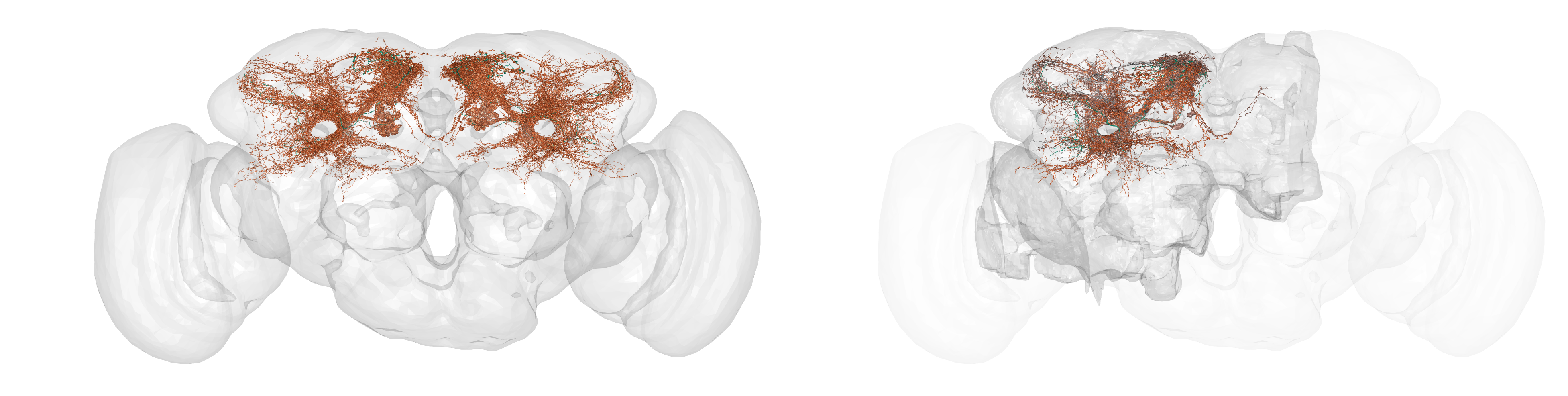

Supplement: Data S5. A .zip archive containing .png files depicting each of the 183 brain hemilineages we have used from the FAFB-FlyWire dataset, related to Figure 7 — Neurons in each hemilineage are colored by their neuron-level transmitter predictions, hemilineage names given in the file name. Hemilineage labels for the FAFB-FlyWire dataset are fully reported in Schlegel et al.S2 [file mmc6.zip › chosen_hemilineages/DM3_dorso_lateral__fafb_hemibrain.png]

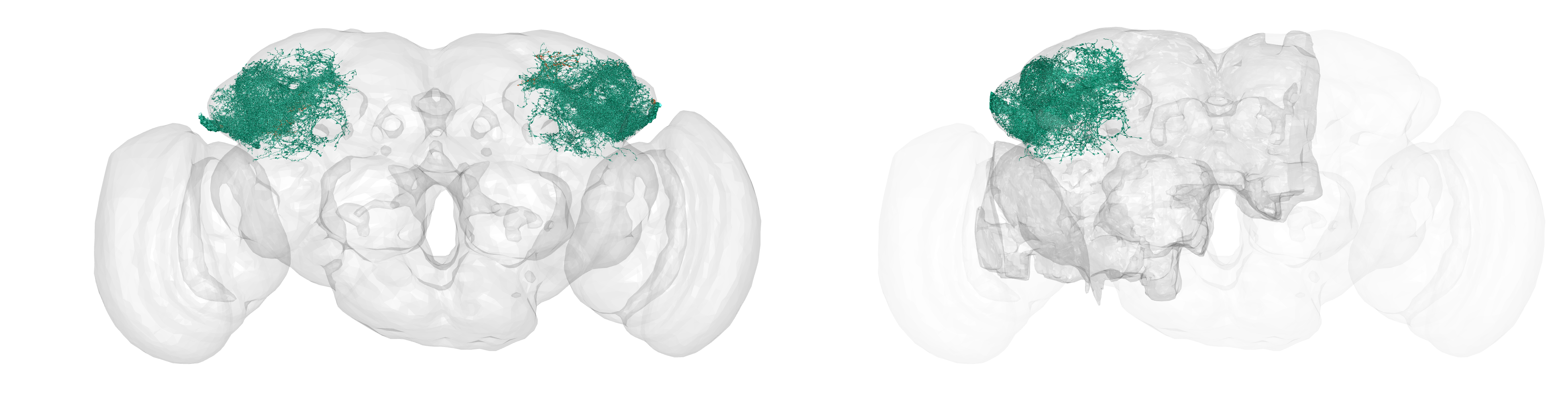

Supplement: Data S5. A .zip archive containing .png files depicting each of the 183 brain hemilineages we have used from the FAFB-FlyWire dataset, related to Figure 7 — Neurons in each hemilineage are colored by their neuron-level transmitter predictions, hemilineage names given in the file name. Hemilineage labels for the FAFB-FlyWire dataset are fully reported in Schlegel et al.S2 [file mmc6.zip › chosen_hemilineages/SLPp&v1_posterior__fafb_hemibrain.png]

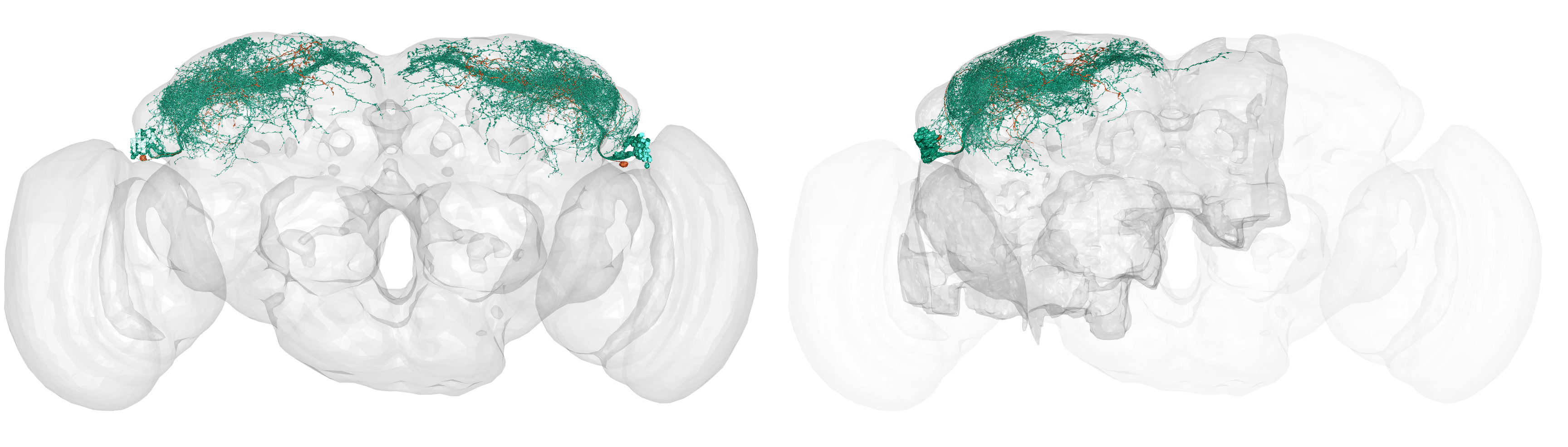

Supplement: Data S5. A .zip archive containing .png files depicting each of the 183 brain hemilineages we have used from the FAFB-FlyWire dataset, related to Figure 7 — Neurons in each hemilineage are colored by their neuron-level transmitter predictions, hemilineage names given in the file name. Hemilineage labels for the FAFB-FlyWire dataset are fully reported in Schlegel et al.S2 [file mmc6.zip › chosen_hemilineages/SLPal5__fafb_hemibrain.png]

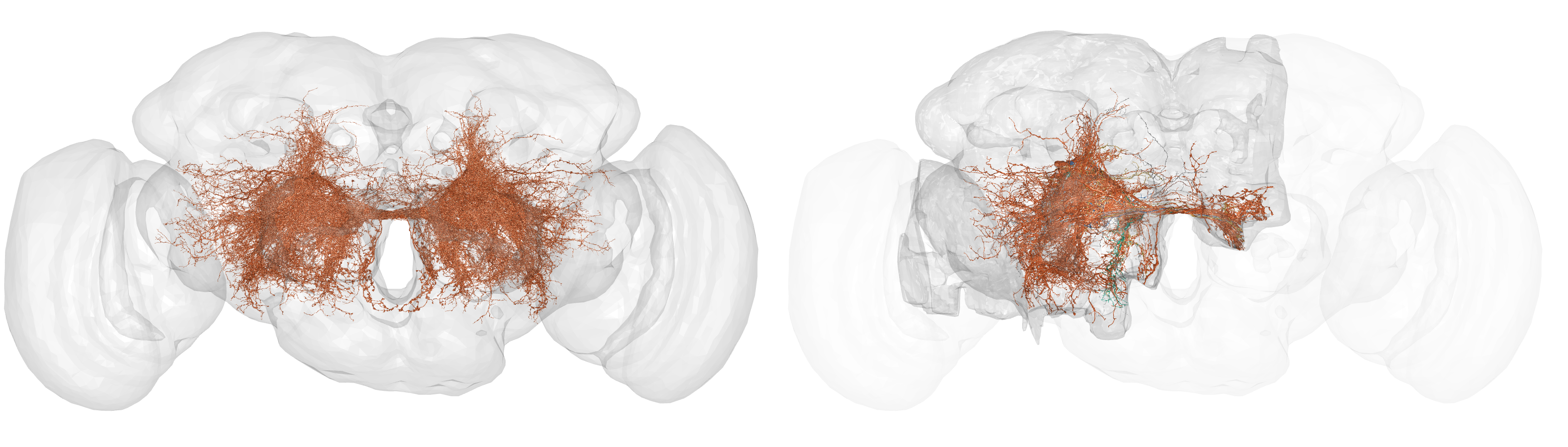

Supplement: Data S5. A .zip archive containing .png files depicting each of the 183 brain hemilineages we have used from the FAFB-FlyWire dataset, related to Figure 7 — Neurons in each hemilineage are colored by their neuron-level transmitter predictions, hemilineage names given in the file name. Hemilineage labels for the FAFB-FlyWire dataset are fully reported in Schlegel et al.S2 [file mmc6.zip › chosen_hemilineages/VLPp2__fafb_hemibrain.png]

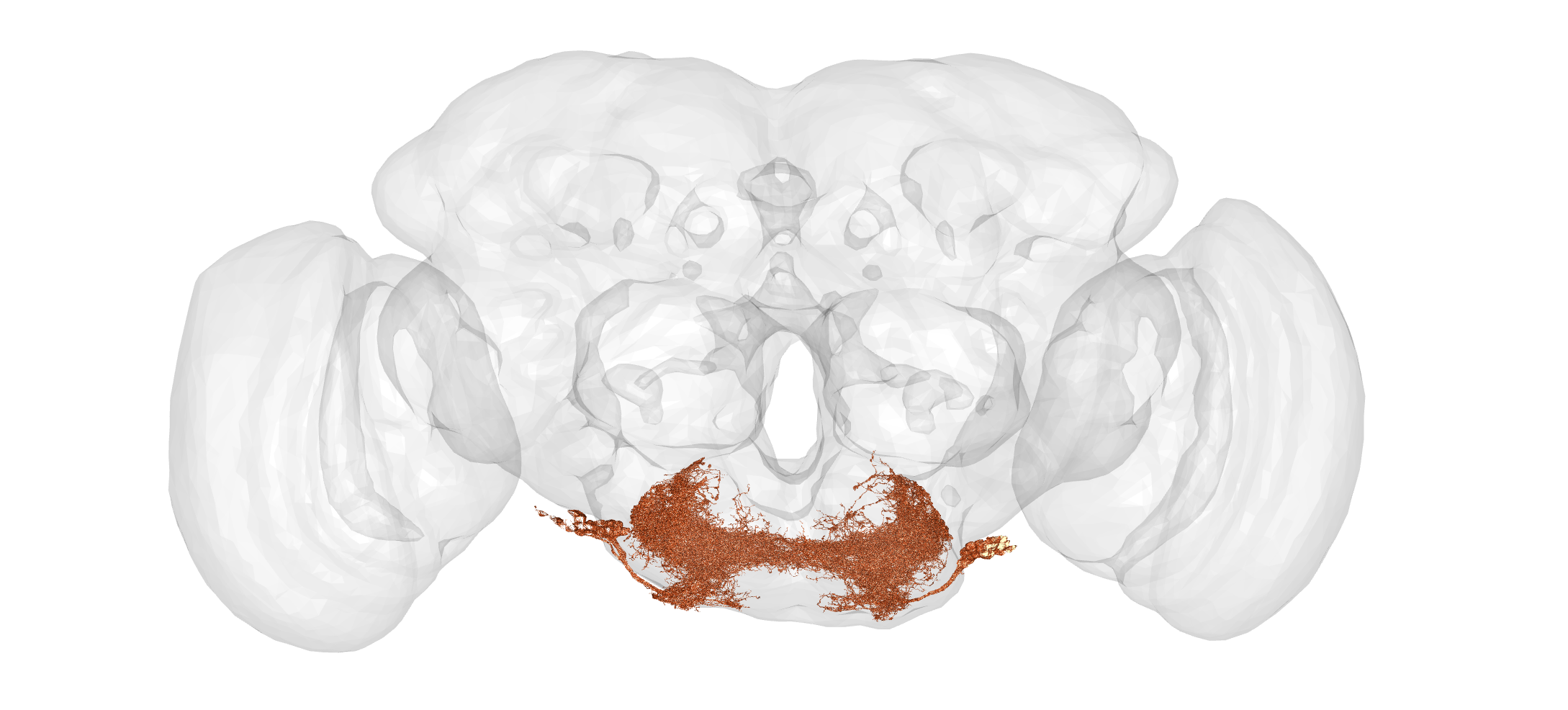

Supplement: Data S5. A .zip archive containing .png files depicting each of the 183 brain hemilineages we have used from the FAFB-FlyWire dataset, related to Figure 7 — Neurons in each hemilineage are colored by their neuron-level transmitter predictions, hemilineage names given in the file name. Hemilineage labels for the FAFB-FlyWire dataset are fully reported in Schlegel et al.S2 [file mmc6.zip › chosen_hemilineages/MD_SA1__fafb.png]

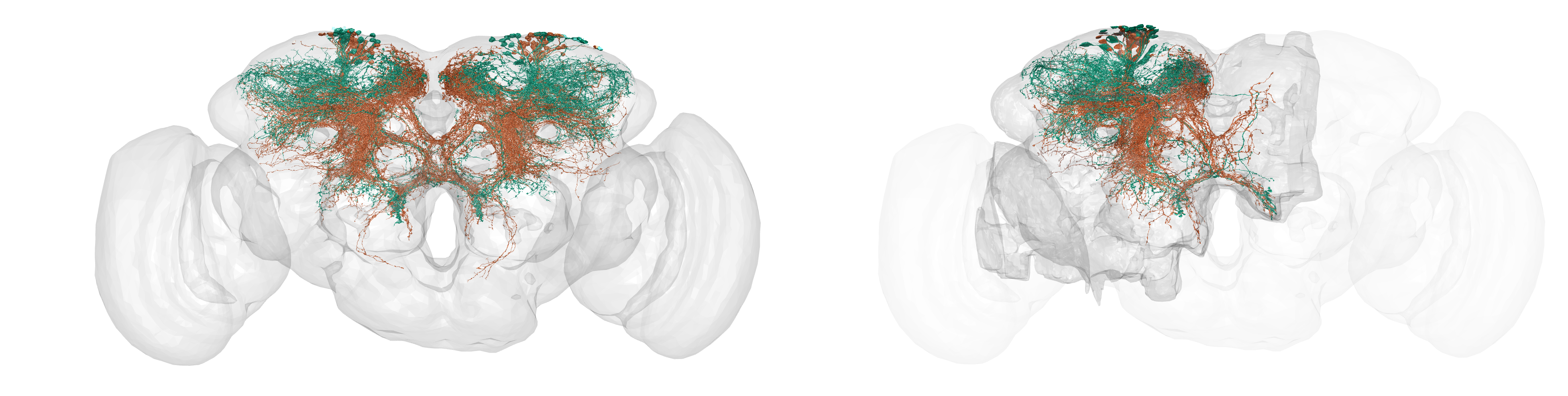

Supplement: Data S5. A .zip archive containing .png files depicting each of the 183 brain hemilineages we have used from the FAFB-FlyWire dataset, related to Figure 7 — Neurons in each hemilineage are colored by their neuron-level transmitter predictions, hemilineage names given in the file name. Hemilineage labels for the FAFB-FlyWire dataset are fully reported in Schlegel et al.S2 [file mmc6.zip › chosen_hemilineages/CLp1__fafb_hemibrain.png]

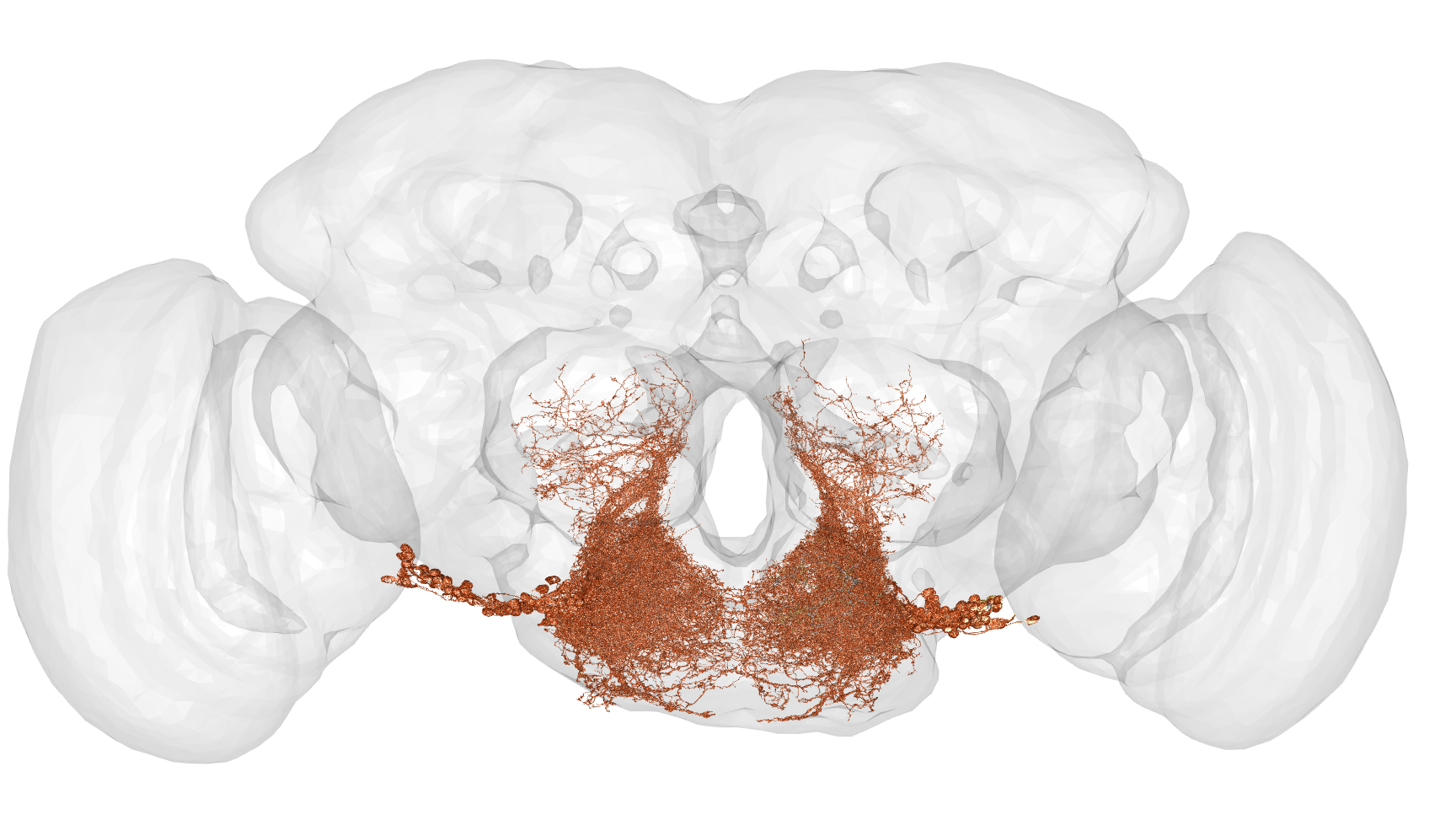

Supplement: Data S5. A .zip archive containing .png files depicting each of the 183 brain hemilineages we have used from the FAFB-FlyWire dataset, related to Figure 7 — Neurons in each hemilineage are colored by their neuron-level transmitter predictions, hemilineage names given in the file name. Hemilineage labels for the FAFB-FlyWire dataset are fully reported in Schlegel et al.S2 [file mmc6.zip › chosen_hemilineages/TRdl_b__fafb.png]

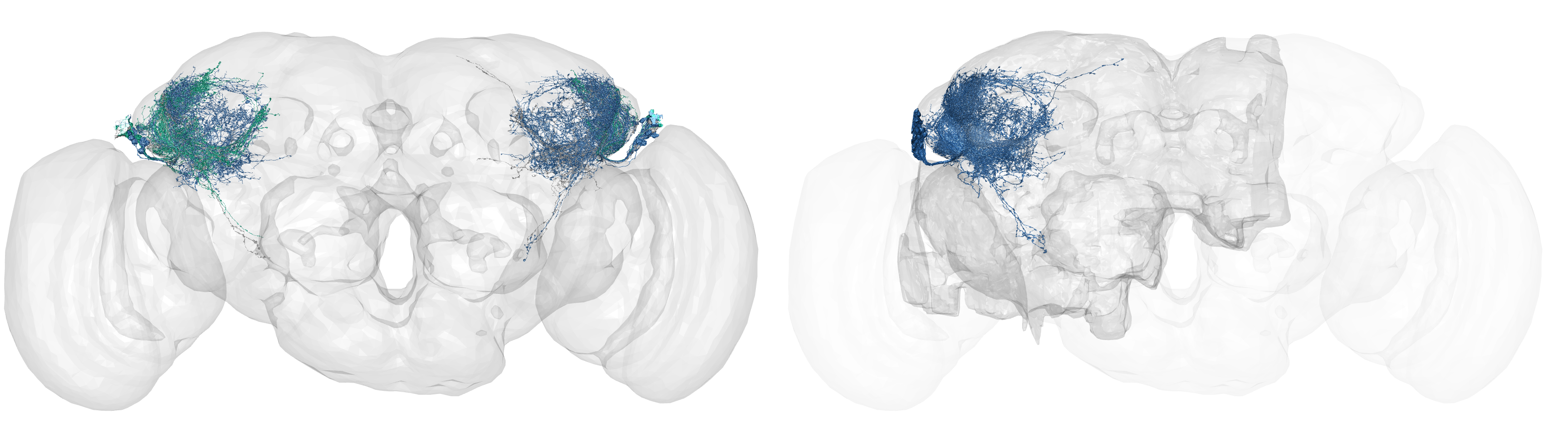

Supplement: Data S5. A .zip archive containing .png files depicting each of the 183 brain hemilineages we have used from the FAFB-FlyWire dataset, related to Figure 7 — Neurons in each hemilineage are colored by their neuron-level transmitter predictions, hemilineage names given in the file name. Hemilineage labels for the FAFB-FlyWire dataset are fully reported in Schlegel et al.S2 [file mmc6.zip › chosen_hemilineages/VLPl4_dorsal__fafb_hemibrain.png]

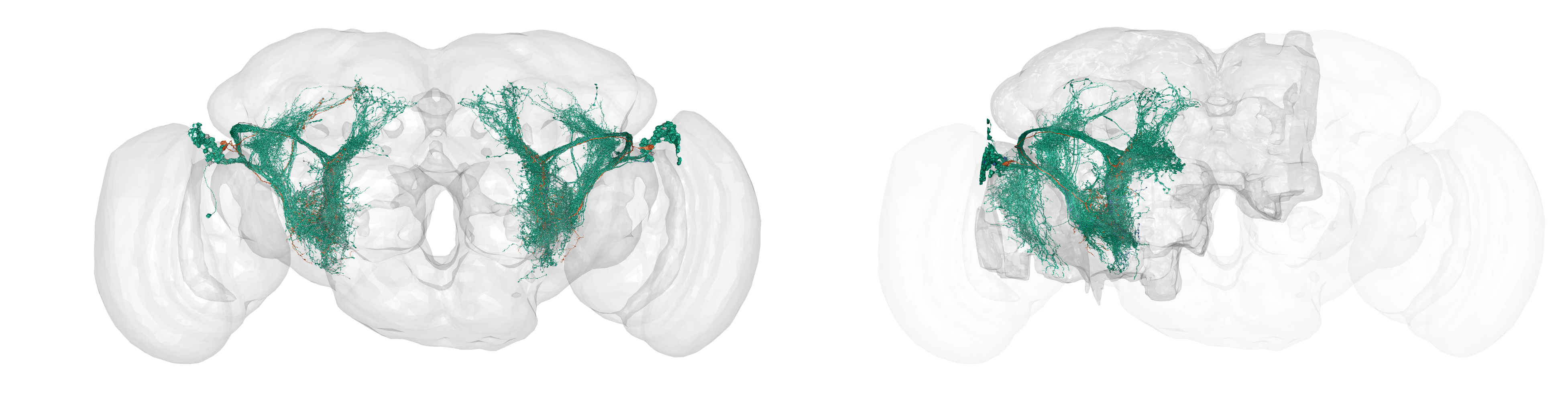

Supplement: Data S5. A .zip archive containing .png files depicting each of the 183 brain hemilineages we have used from the FAFB-FlyWire dataset, related to Figure 7 — Neurons in each hemilineage are colored by their neuron-level transmitter predictions, hemilineage names given in the file name. Hemilineage labels for the FAFB-FlyWire dataset are fully reported in Schlegel et al.S2 [file mmc6.zip › chosen_hemilineages/SLPa&l1_lateral__fafb_hemibrain.png]

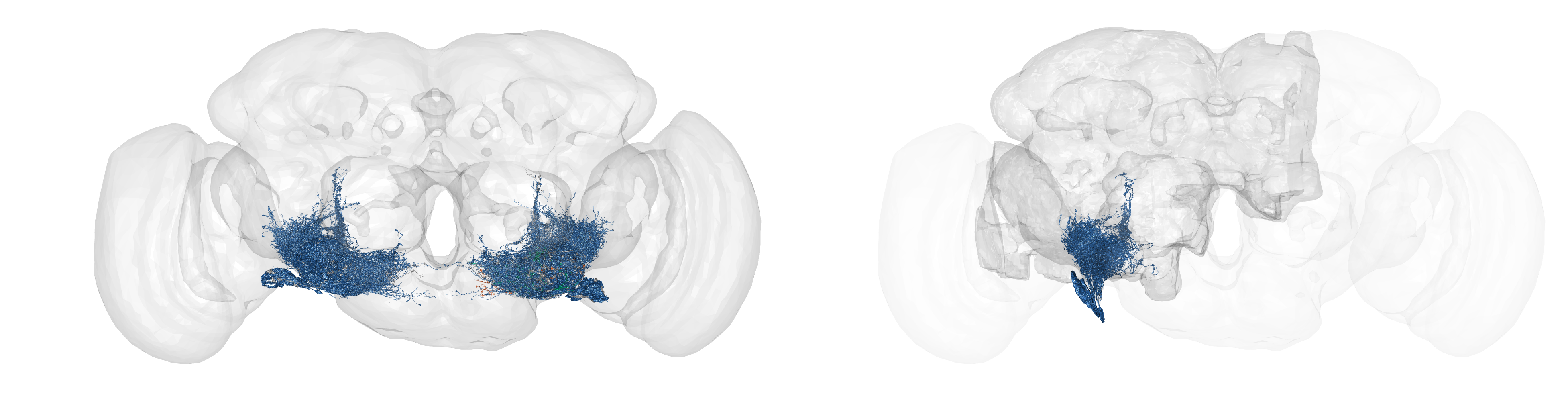

Supplement: Data S5. A .zip archive containing .png files depicting each of the 183 brain hemilineages we have used from the FAFB-FlyWire dataset, related to Figure 7 — Neurons in each hemilineage are colored by their neuron-level transmitter predictions, hemilineage names given in the file name. Hemilineage labels for the FAFB-FlyWire dataset are fully reported in Schlegel et al.S2 [file mmc6.zip › chosen_hemilineages/WEDa1__fafb_hemibrain.png]

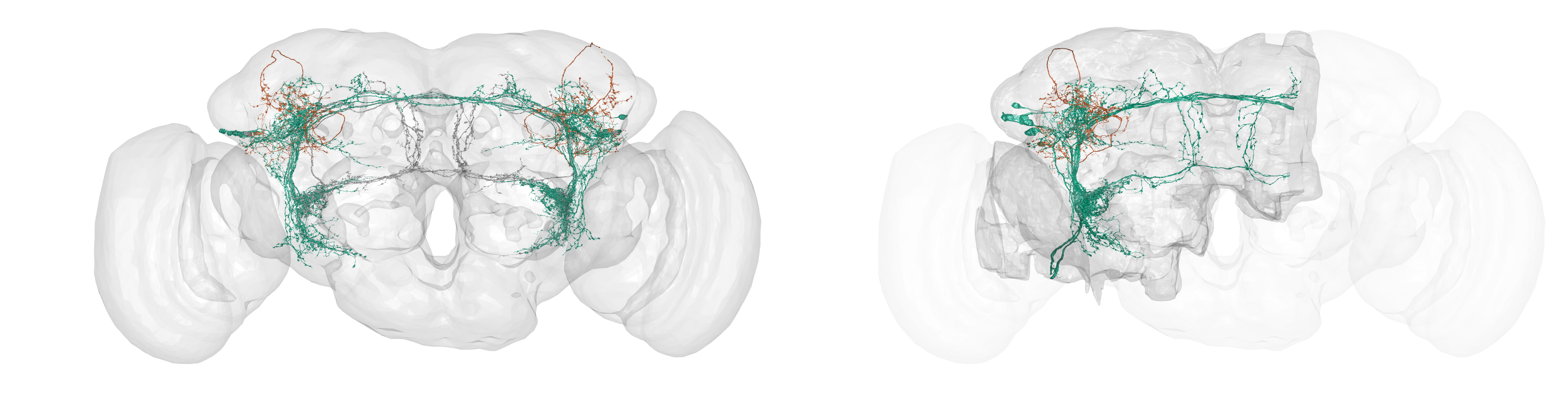

Supplement: Data S5. A .zip archive containing .png files depicting each of the 183 brain hemilineages we have used from the FAFB-FlyWire dataset, related to Figure 7 — Neurons in each hemilineage are colored by their neuron-level transmitter predictions, hemilineage names given in the file name. Hemilineage labels for the FAFB-FlyWire dataset are fully reported in Schlegel et al.S2 [file mmc6.zip › chosen_hemilineages/SLPp&v1_ventral__fafb_hemibrain.png]

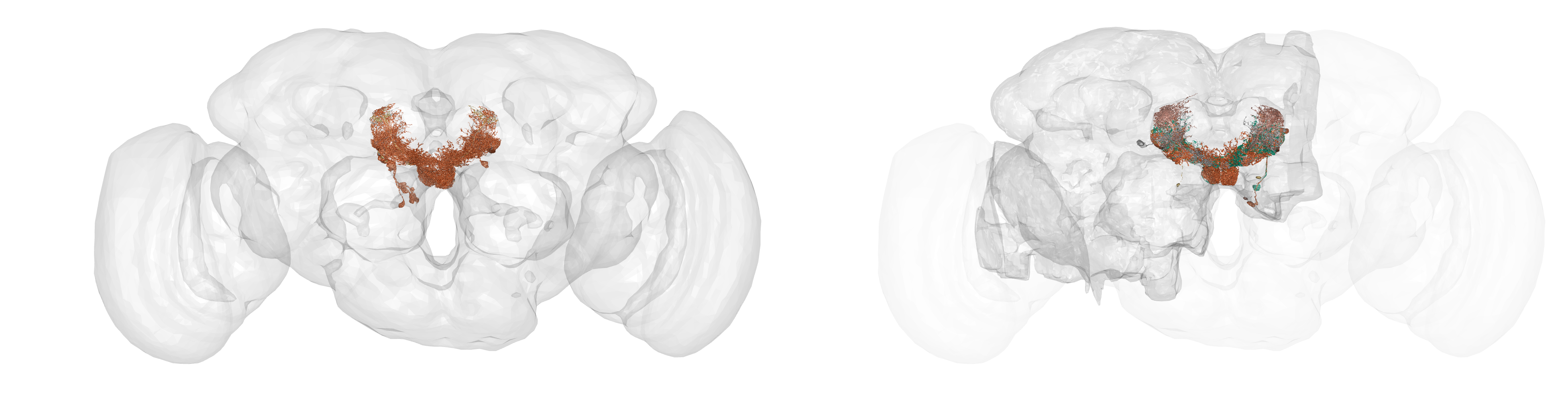

Supplement: Data S5. A .zip archive containing .png files depicting each of the 183 brain hemilineages we have used from the FAFB-FlyWire dataset, related to Figure 7 — Neurons in each hemilineage are colored by their neuron-level transmitter predictions, hemilineage names given in the file name. Hemilineage labels for the FAFB-FlyWire dataset are fully reported in Schlegel et al.S2 [file mmc6.zip › chosen_hemilineages/DM4_CX_v__fafb_hemibrain.png]

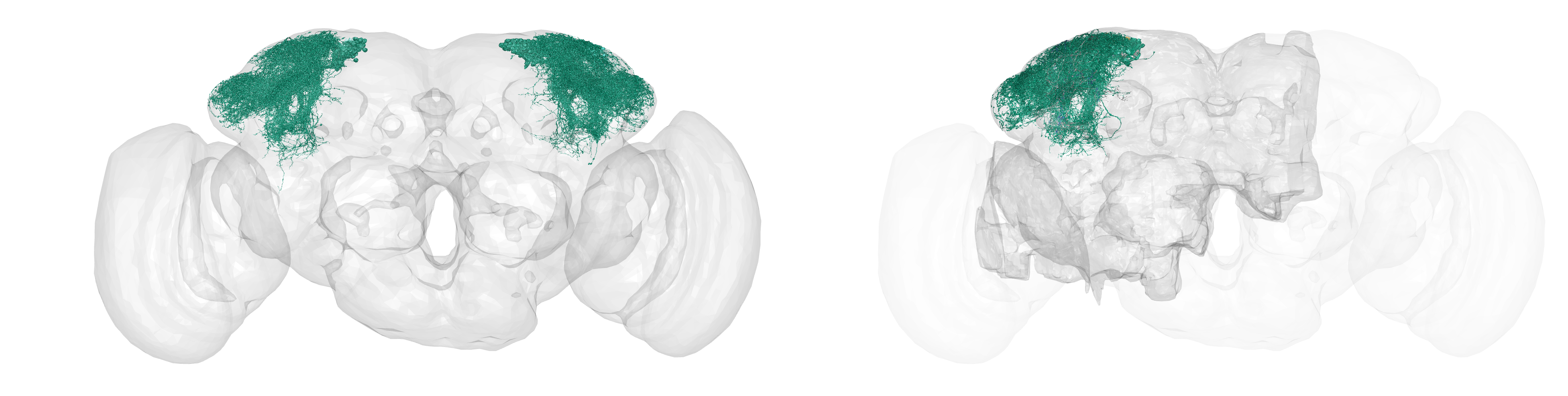

Supplement: Data S5. A .zip archive containing .png files depicting each of the 183 brain hemilineages we have used from the FAFB-FlyWire dataset, related to Figure 7 — Neurons in each hemilineage are colored by their neuron-level transmitter predictions, hemilineage names given in the file name. Hemilineage labels for the FAFB-FlyWire dataset are fully reported in Schlegel et al.S2 [file mmc6.zip › chosen_hemilineages/SLPpm1__fafb_hemibrain.png]

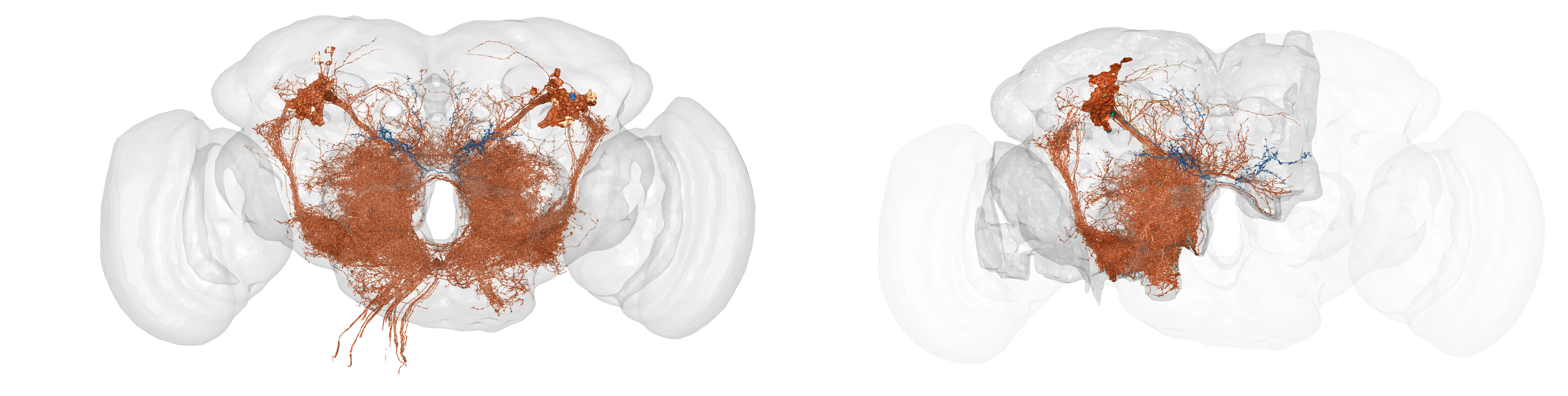

Supplement: Data S5. A .zip archive containing .png files depicting each of the 183 brain hemilineages we have used from the FAFB-FlyWire dataset, related to Figure 7 — Neurons in each hemilineage are colored by their neuron-level transmitter predictions, hemilineage names given in the file name. Hemilineage labels for the FAFB-FlyWire dataset are fully reported in Schlegel et al.S2 [file mmc6.zip › chosen_hemilineages/WEDd1__fafb_hemibrain.png]

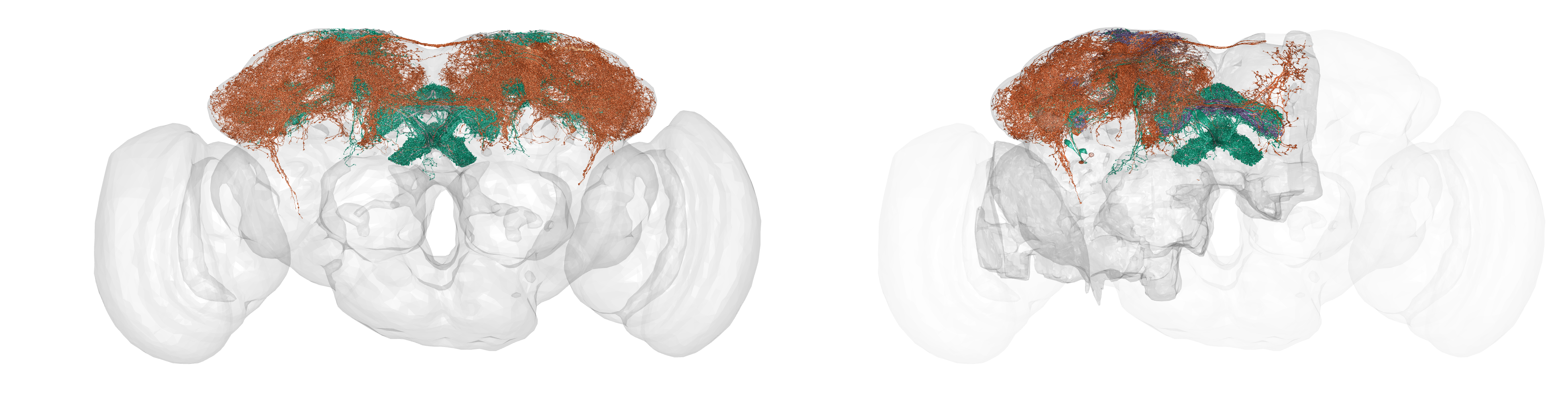

Supplement: Data S5. A .zip archive containing .png files depicting each of the 183 brain hemilineages we have used from the FAFB-FlyWire dataset, related to Figure 7 — Neurons in each hemilineage are colored by their neuron-level transmitter predictions, hemilineage names given in the file name. Hemilineage labels for the FAFB-FlyWire dataset are fully reported in Schlegel et al.S2 [file mmc6.zip › chosen_hemilineages/DL1_dorsal__fafb_hemibrain.png]

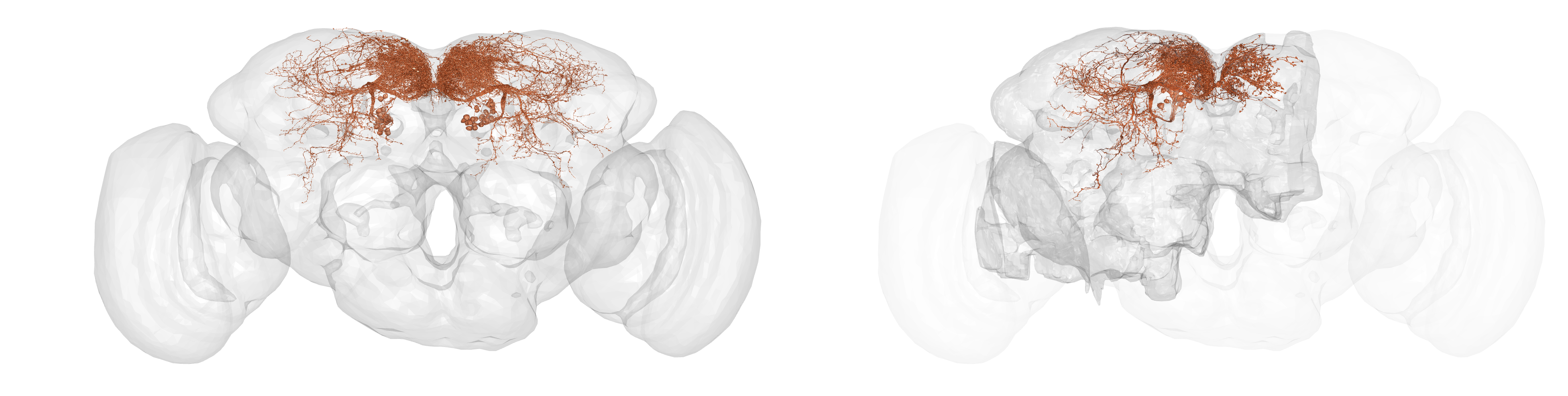

Supplement: Data S5. A .zip archive containing .png files depicting each of the 183 brain hemilineages we have used from the FAFB-FlyWire dataset, related to Figure 7 — Neurons in each hemilineage are colored by their neuron-level transmitter predictions, hemilineage names given in the file name. Hemilineage labels for the FAFB-FlyWire dataset are fully reported in Schlegel et al.S2 [file mmc6.zip › chosen_hemilineages/DM3_dorso_medial__fafb_hemibrain.png]

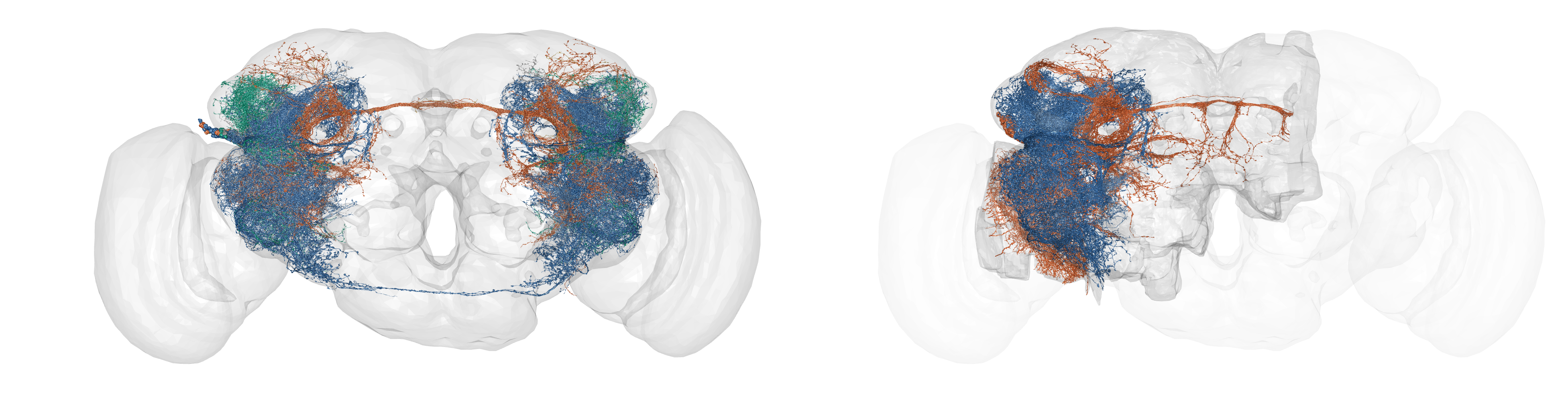

Supplement: Data S5. A .zip archive containing .png files depicting each of the 183 brain hemilineages we have used from the FAFB-FlyWire dataset, related to Figure 7 — Neurons in each hemilineage are colored by their neuron-level transmitter predictions, hemilineage names given in the file name. Hemilineage labels for the FAFB-FlyWire dataset are fully reported in Schlegel et al.S2 [file mmc6.zip › chosen_hemilineages/VPNp&v1_posterior__fafb_hemibrain.png]

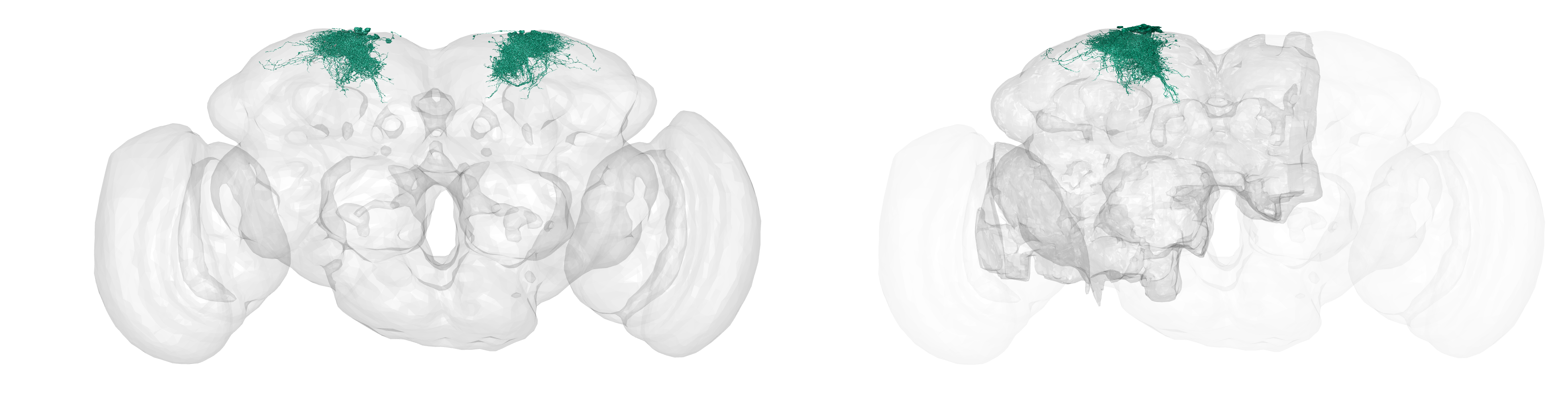

Supplement: Data S5. A .zip archive containing .png files depicting each of the 183 brain hemilineages we have used from the FAFB-FlyWire dataset, related to Figure 7 — Neurons in each hemilineage are colored by their neuron-level transmitter predictions, hemilineage names given in the file name. Hemilineage labels for the FAFB-FlyWire dataset are fully reported in Schlegel et al.S2 [file mmc6.zip › chosen_hemilineages/SLPpm2__fafb_hemibrain.png]

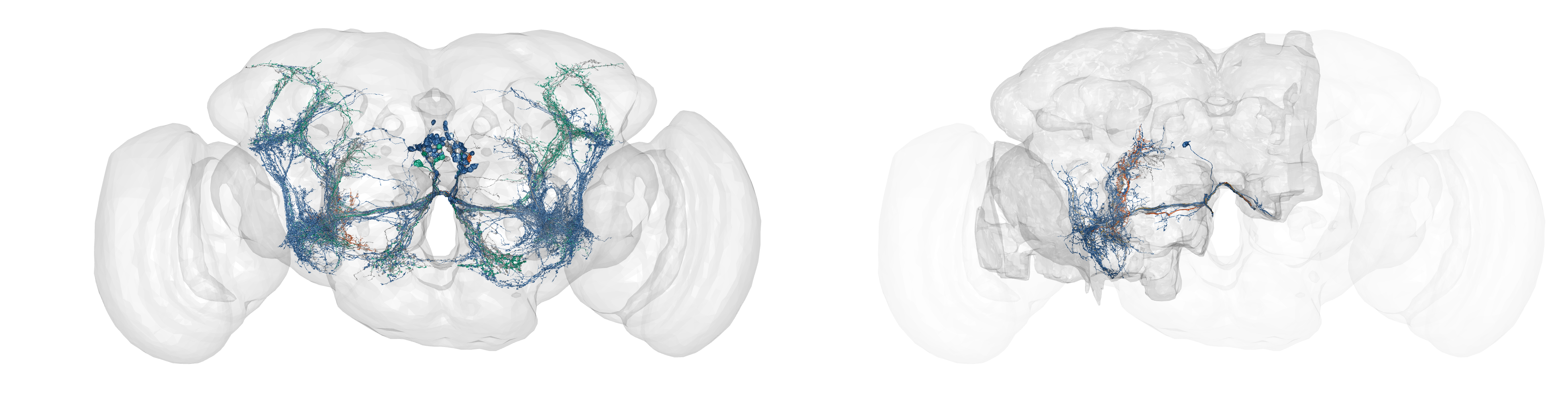

Supplement: Data S5. A .zip archive containing .png files depicting each of the 183 brain hemilineages we have used from the FAFB-FlyWire dataset, related to Figure 7 — Neurons in each hemilineage are colored by their neuron-level transmitter predictions, hemilineage names given in the file name. Hemilineage labels for the FAFB-FlyWire dataset are fully reported in Schlegel et al.S2 [file mmc6.zip › chosen_hemilineages/WEDd2__fafb_hemibrain.png]

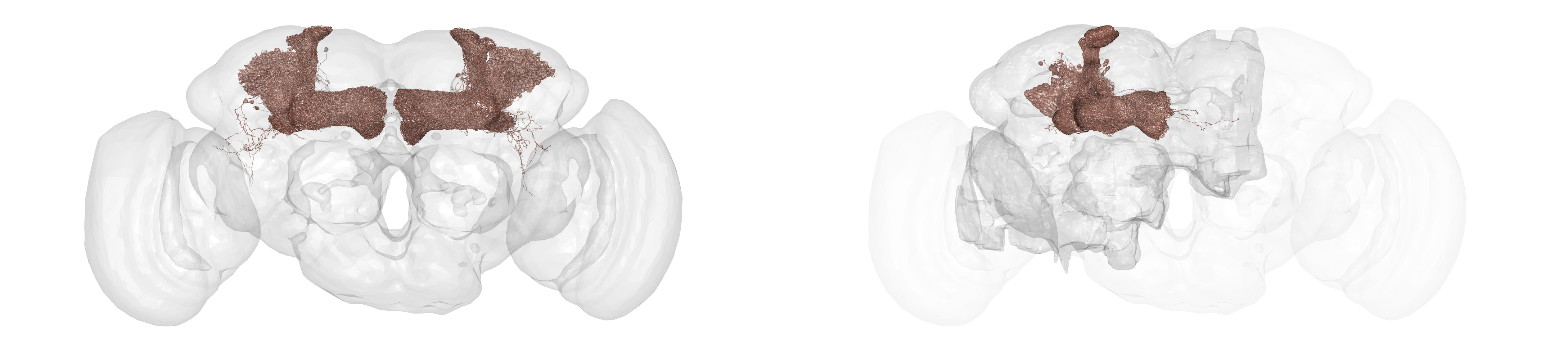

Supplement: Data S5. A .zip archive containing .png files depicting each of the 183 brain hemilineages we have used from the FAFB-FlyWire dataset, related to Figure 7 — Neurons in each hemilineage are colored by their neuron-level transmitter predictions, hemilineage names given in the file name. Hemilineage labels for the FAFB-FlyWire dataset are fully reported in Schlegel et al.S2 [file mmc6.zip › chosen_hemilineages/MBp4__fafb_hemibrain.png]

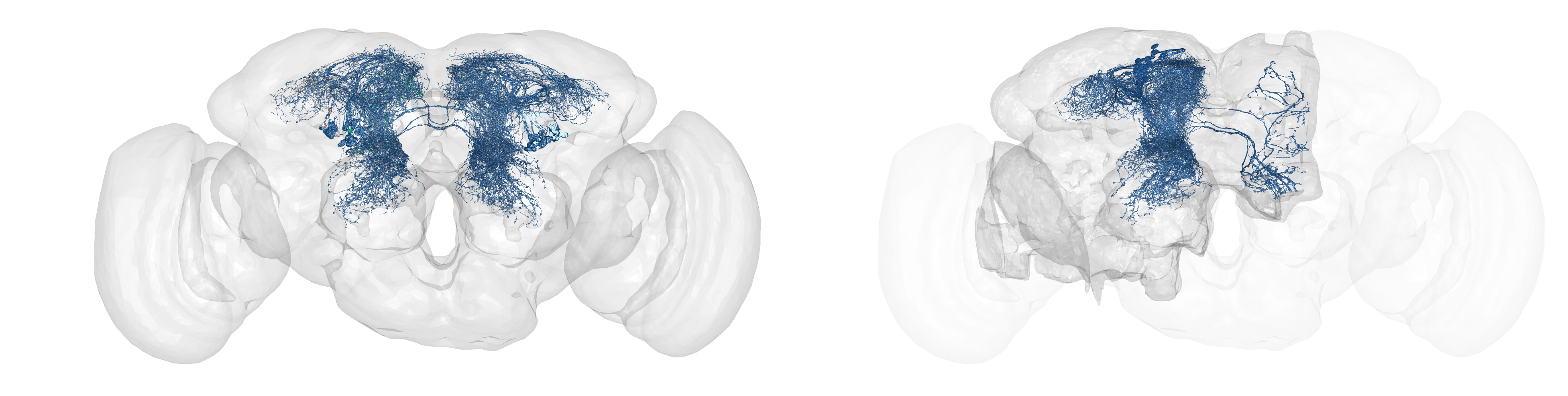

Supplement: Data S5. A .zip archive containing .png files depicting each of the 183 brain hemilineages we have used from the FAFB-FlyWire dataset, related to Figure 7 — Neurons in each hemilineage are colored by their neuron-level transmitter predictions, hemilineage names given in the file name. Hemilineage labels for the FAFB-FlyWire dataset are fully reported in Schlegel et al.S2 [file mmc6.zip › chosen_hemilineages/AOTUv1_medial__fafb_hemibrain.png]

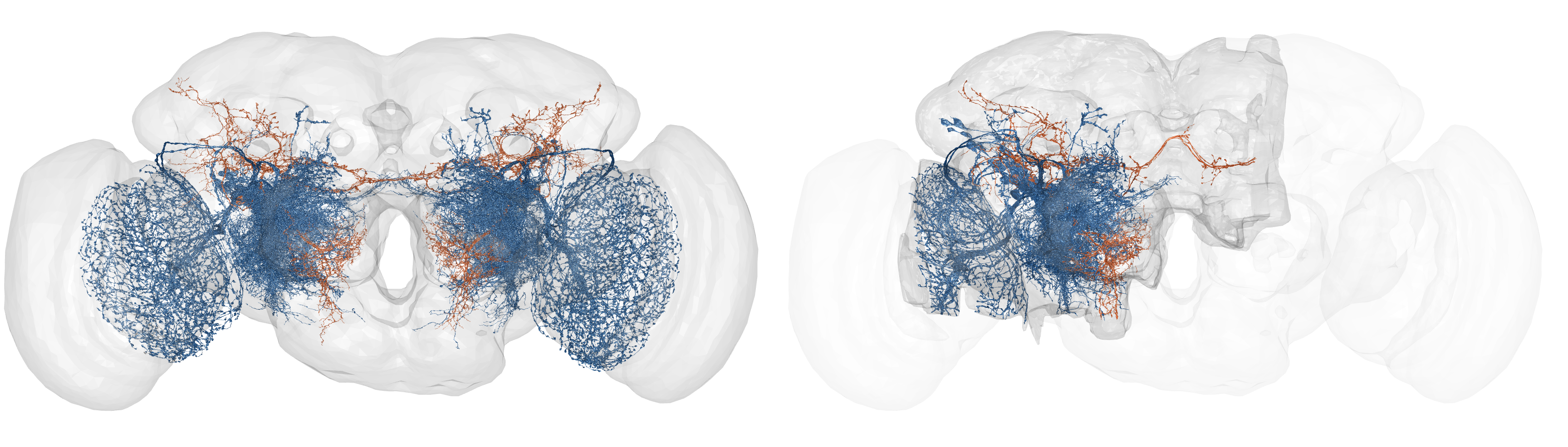

Supplement: Data S5. A .zip archive containing .png files depicting each of the 183 brain hemilineages we have used from the FAFB-FlyWire dataset, related to Figure 7 — Neurons in each hemilineage are colored by their neuron-level transmitter predictions, hemilineage names given in the file name. Hemilineage labels for the FAFB-FlyWire dataset are fully reported in Schlegel et al.S2 [file mmc6.zip › chosen_hemilineages/VLPp&l1_posterior__fafb_hemibrain.png]

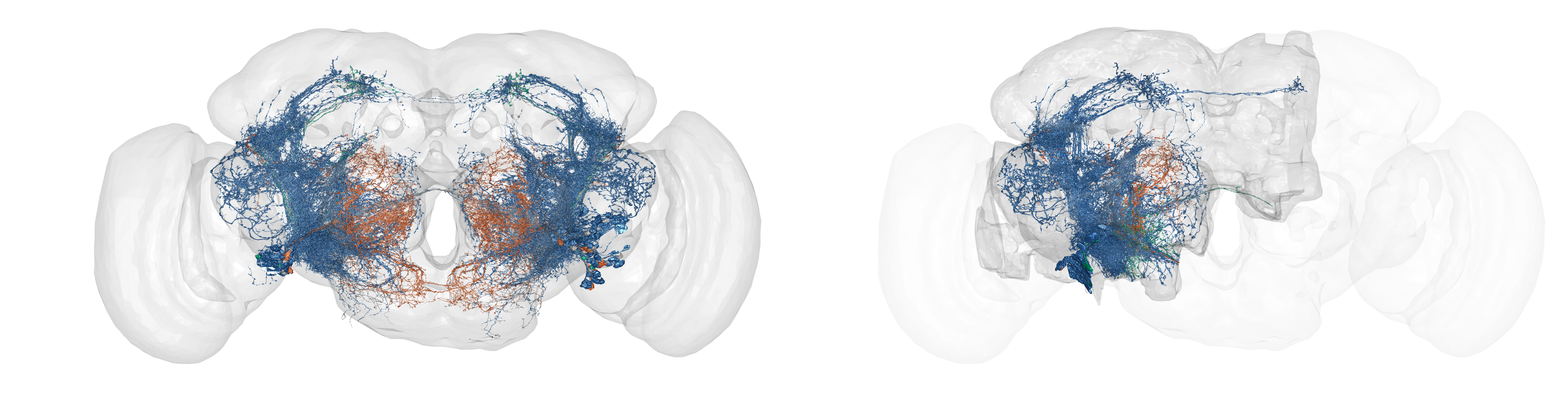

Supplement: Data S5. A .zip archive containing .png files depicting each of the 183 brain hemilineages we have used from the FAFB-FlyWire dataset, related to Figure 7 — Neurons in each hemilineage are colored by their neuron-level transmitter predictions, hemilineage names given in the file name. Hemilineage labels for the FAFB-FlyWire dataset are fully reported in Schlegel et al.S2 [file mmc6.zip › chosen_hemilineages/WEDa2__fafb_hemibrain.png]

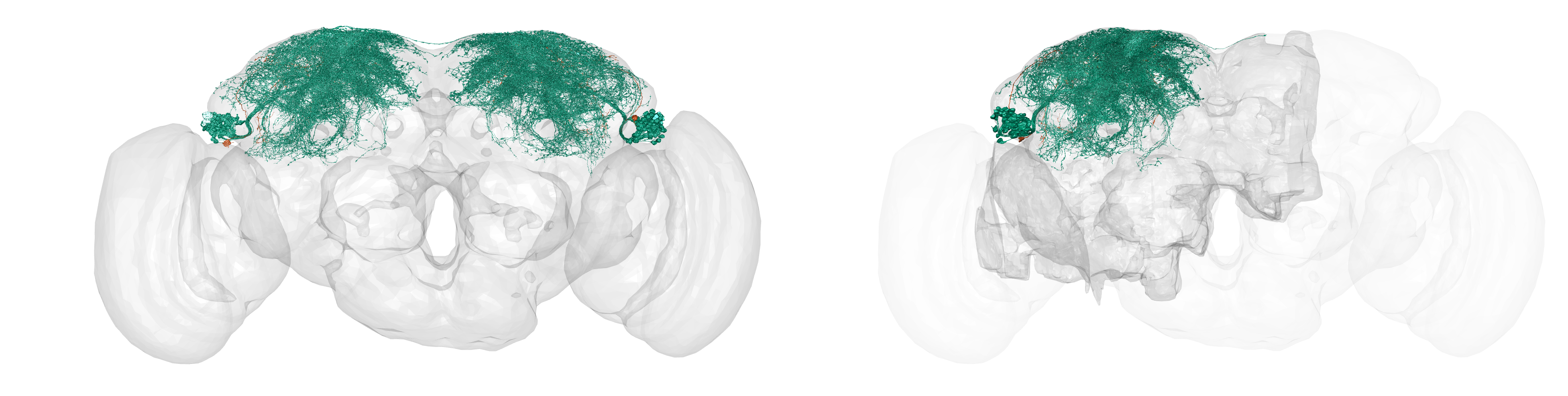

Supplement: Data S5. A .zip archive containing .png files depicting each of the 183 brain hemilineages we have used from the FAFB-FlyWire dataset, related to Figure 7 — Neurons in each hemilineage are colored by their neuron-level transmitter predictions, hemilineage names given in the file name. Hemilineage labels for the FAFB-FlyWire dataset are fully reported in Schlegel et al.S2 [file mmc6.zip › chosen_hemilineages/SIPa1_ventral__fafb_hemibrain.png]

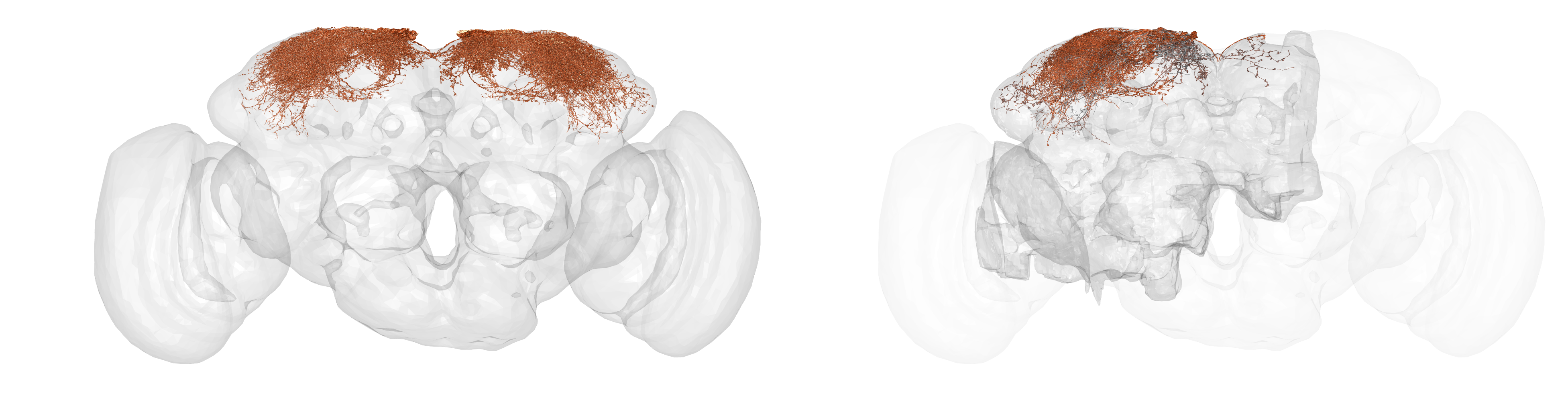

Supplement: Data S5. A .zip archive containing .png files depicting each of the 183 brain hemilineages we have used from the FAFB-FlyWire dataset, related to Figure 7 — Neurons in each hemilineage are colored by their neuron-level transmitter predictions, hemilineage names given in the file name. Hemilineage labels for the FAFB-FlyWire dataset are fully reported in Schlegel et al.S2 [file mmc6.zip › chosen_hemilineages/SLPpm3__fafb_hemibrain.png]

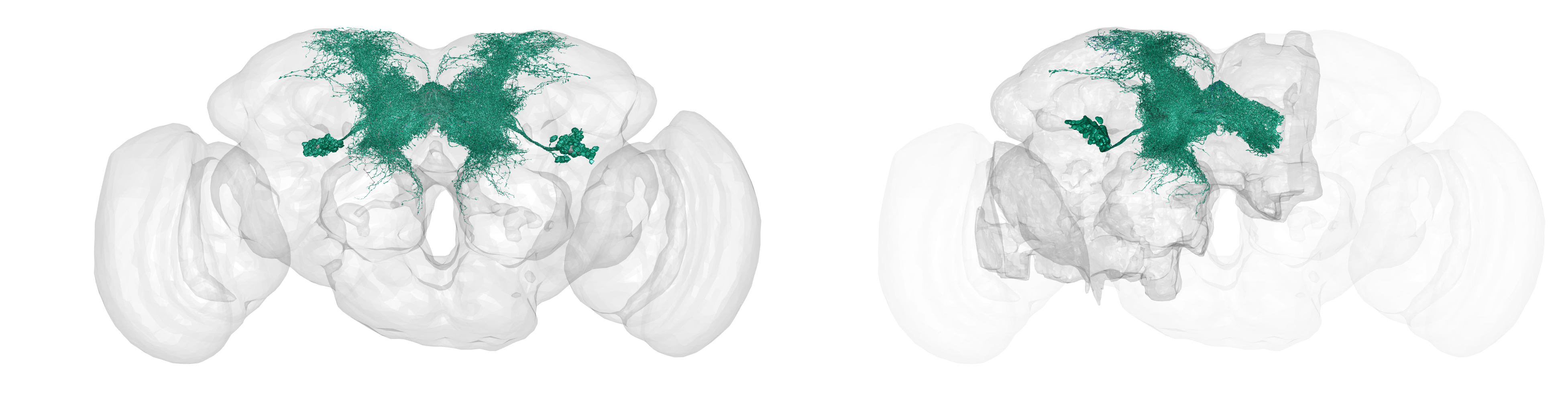

Supplement: Data S5. A .zip archive containing .png files depicting each of the 183 brain hemilineages we have used from the FAFB-FlyWire dataset, related to Figure 7 — Neurons in each hemilineage are colored by their neuron-level transmitter predictions, hemilineage names given in the file name. Hemilineage labels for the FAFB-FlyWire dataset are fully reported in Schlegel et al.S2 [file mmc6.zip › chosen_hemilineages/AOTUv4_ventral__fafb_hemibrain.png]

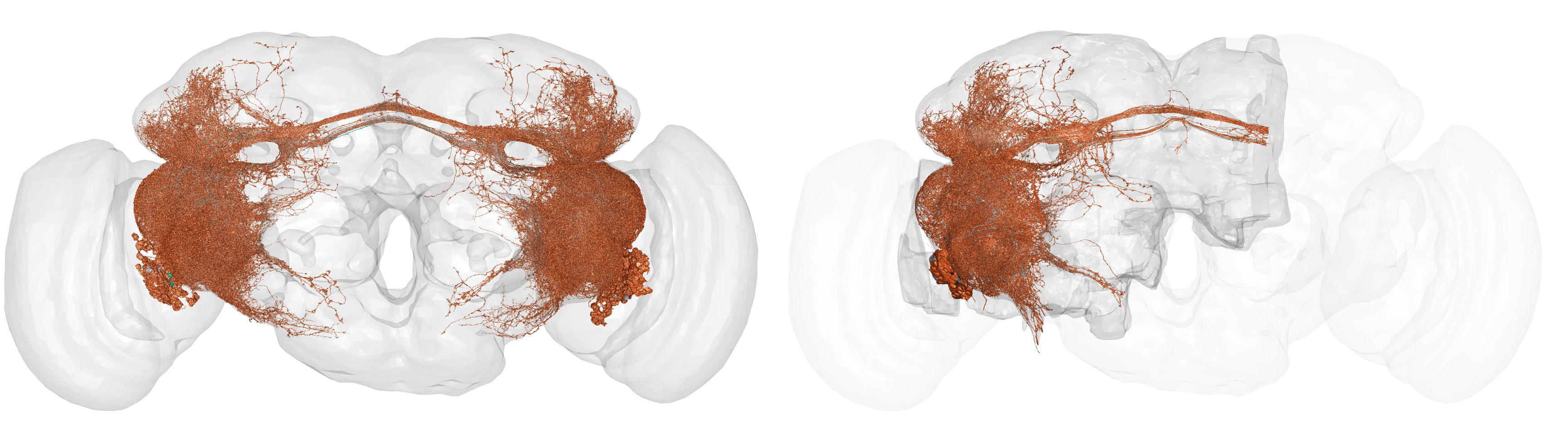

Supplement: Data S5. A .zip archive containing .png files depicting each of the 183 brain hemilineages we have used from the FAFB-FlyWire dataset, related to Figure 7 — Neurons in each hemilineage are colored by their neuron-level transmitter predictions, hemilineage names given in the file name. Hemilineage labels for the FAFB-FlyWire dataset are fully reported in Schlegel et al.S2 [file mmc6.zip › chosen_hemilineages/VLPl&p2_lateral__fafb_hemibrain.png]

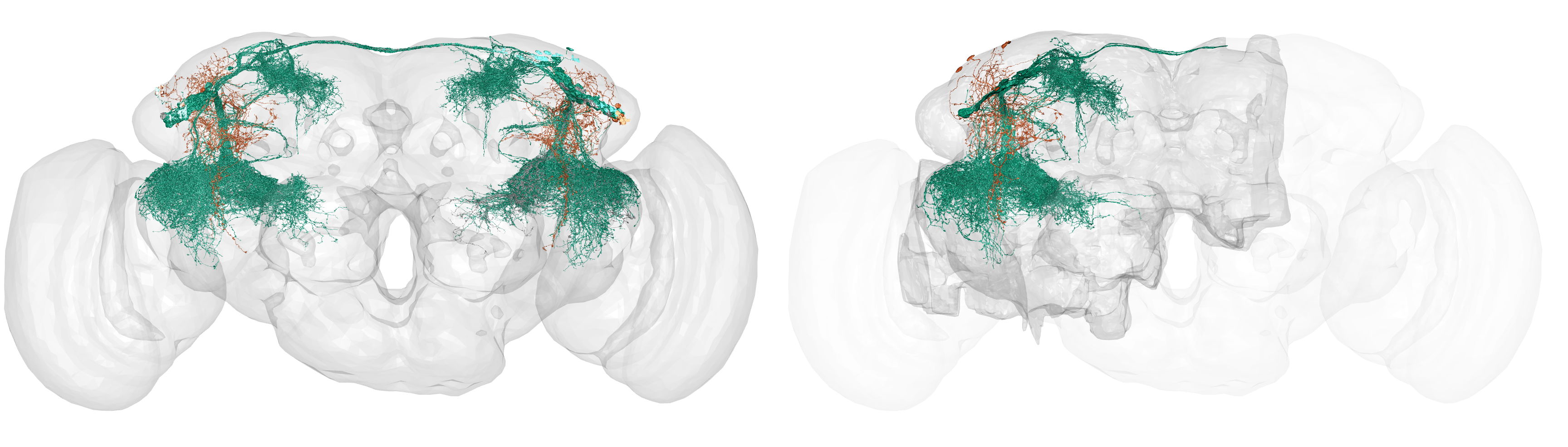

Supplement: Data S5. A .zip archive containing .png files depicting each of the 183 brain hemilineages we have used from the FAFB-FlyWire dataset, related to Figure 7 — Neurons in each hemilineage are colored by their neuron-level transmitter predictions, hemilineage names given in the file name. Hemilineage labels for the FAFB-FlyWire dataset are fully reported in Schlegel et al.S2 [file mmc6.zip › chosen_hemilineages/VLPd&p1_dorsal__fafb_hemibrain.png]

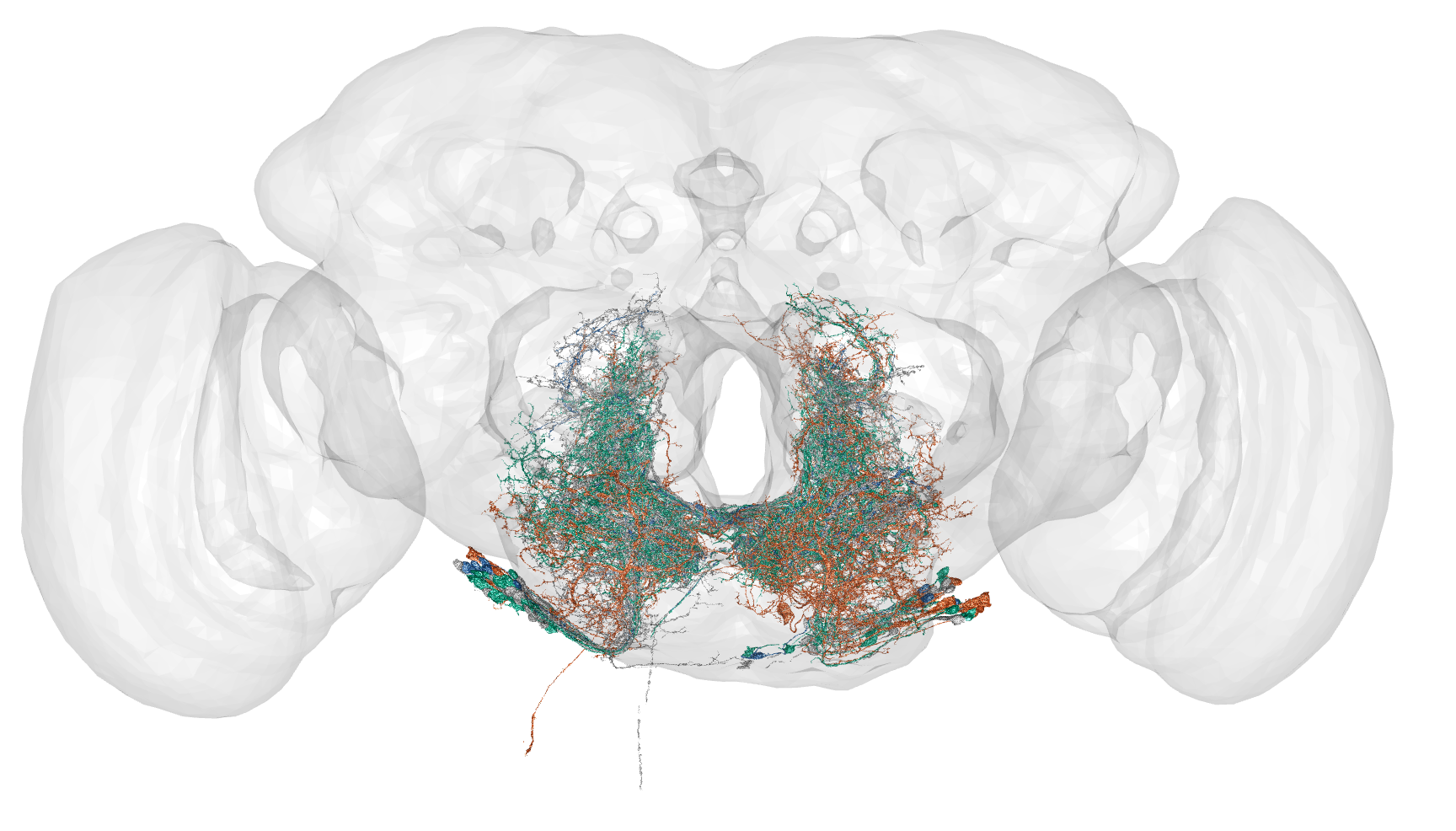

Supplement: Data S5. A .zip archive containing .png files depicting each of the 183 brain hemilineages we have used from the FAFB-FlyWire dataset, related to Figure 7 — Neurons in each hemilineage are colored by their neuron-level transmitter predictions, hemilineage names given in the file name. Hemilineage labels for the FAFB-FlyWire dataset are fully reported in Schlegel et al.S2 [file mmc6.zip › chosen_hemilineages/LB6__fafb.png]

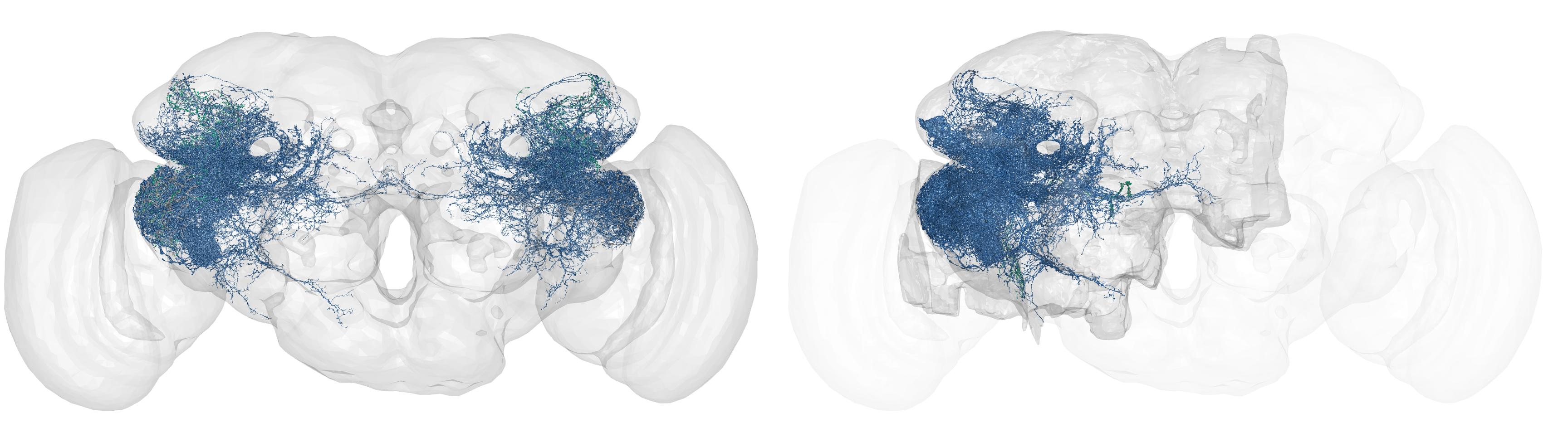

Supplement: Data S5. A .zip archive containing .png files depicting each of the 183 brain hemilineages we have used from the FAFB-FlyWire dataset, related to Figure 7 — Neurons in each hemilineage are colored by their neuron-level transmitter predictions, hemilineage names given in the file name. Hemilineage labels for the FAFB-FlyWire dataset are fully reported in Schlegel et al.S2 [file mmc6.zip › chosen_hemilineages/VLPp&l1_anterior__fafb_hemibrain.png]

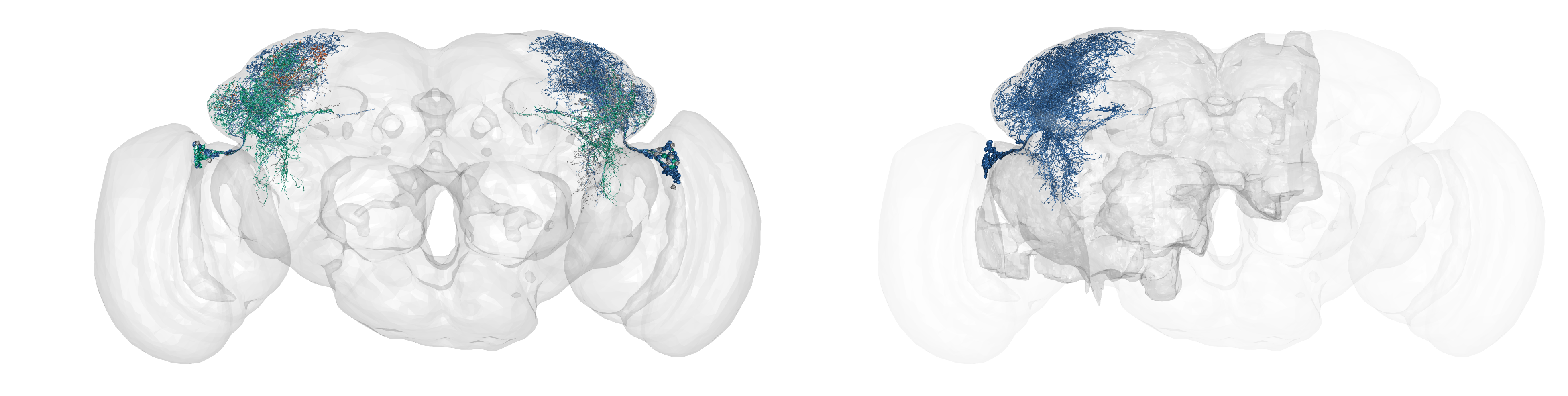

Supplement: Data S5. A .zip archive containing .png files depicting each of the 183 brain hemilineages we have used from the FAFB-FlyWire dataset, related to Figure 7 — Neurons in each hemilineage are colored by their neuron-level transmitter predictions, hemilineage names given in the file name. Hemilineage labels for the FAFB-FlyWire dataset are fully reported in Schlegel et al.S2 [file mmc6.zip › chosen_hemilineages/SLPav3__fafb_hemibrain.png]

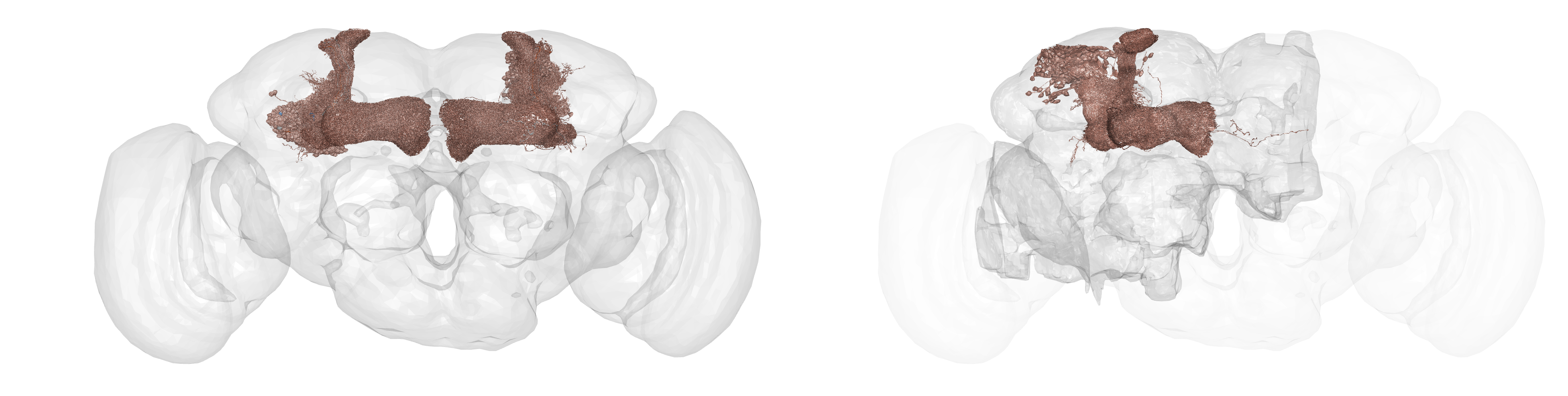

Supplement: Data S5. A .zip archive containing .png files depicting each of the 183 brain hemilineages we have used from the FAFB-FlyWire dataset, related to Figure 7 — Neurons in each hemilineage are colored by their neuron-level transmitter predictions, hemilineage names given in the file name. Hemilineage labels for the FAFB-FlyWire dataset are fully reported in Schlegel et al.S2 [file mmc6.zip › chosen_hemilineages/MBp3__fafb_hemibrain.png]

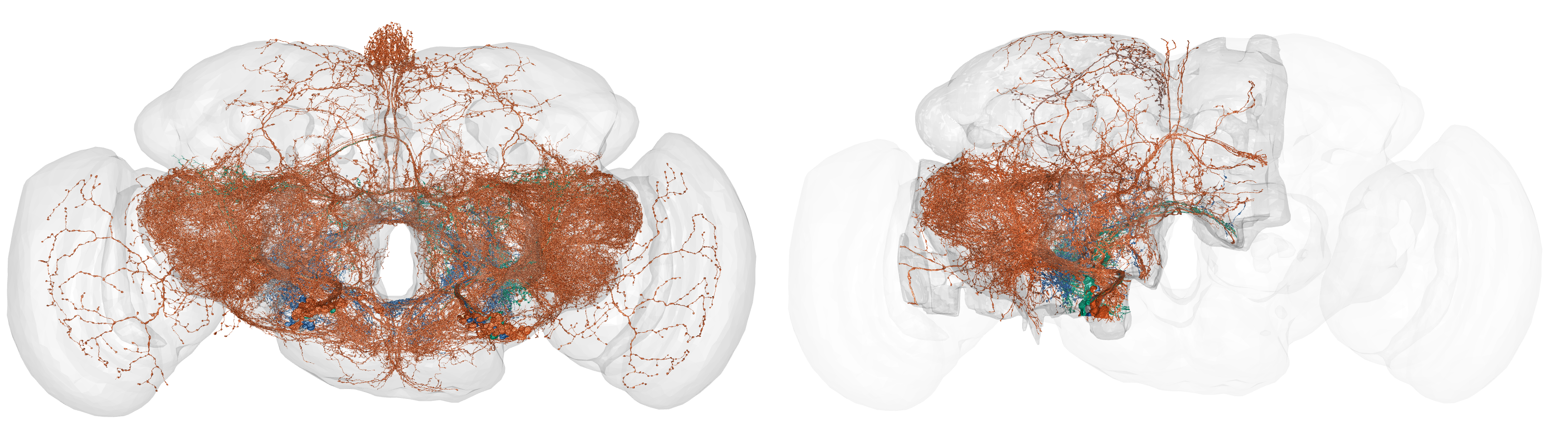

Supplement: Data S5. A .zip archive containing .png files depicting each of the 183 brain hemilineages we have used from the FAFB-FlyWire dataset, related to Figure 7 — Neurons in each hemilineage are colored by their neuron-level transmitter predictions, hemilineage names given in the file name. Hemilineage labels for the FAFB-FlyWire dataset are fully reported in Schlegel et al.S2 [file mmc6.zip › chosen_hemilineages/LALv1_ventral__fafb_hemibrain.png]

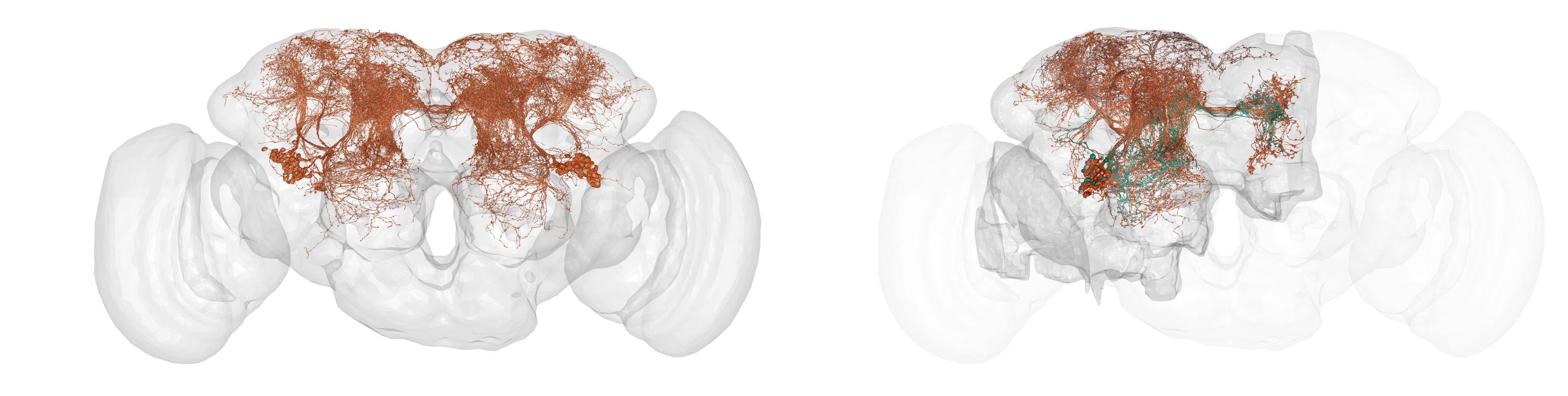

Supplement: Data S5. A .zip archive containing .png files depicting each of the 183 brain hemilineages we have used from the FAFB-FlyWire dataset, related to Figure 7 — Neurons in each hemilineage are colored by their neuron-level transmitter predictions, hemilineage names given in the file name. Hemilineage labels for the FAFB-FlyWire dataset are fully reported in Schlegel et al.S2 [file mmc6.zip › chosen_hemilineages/CREl1__fafb_hemibrain.png]

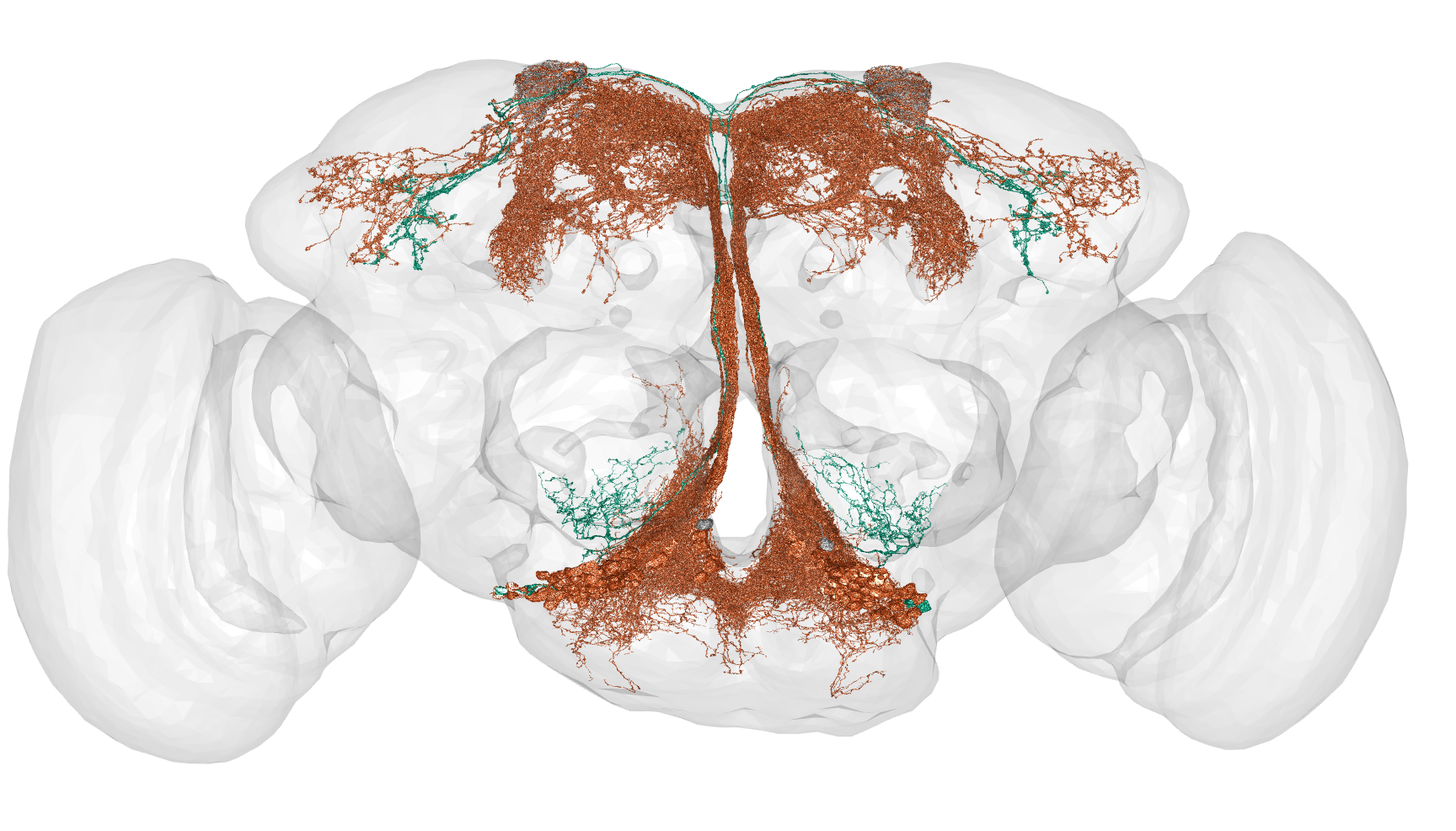

Supplement: Data S5. A .zip archive containing .png files depicting each of the 183 brain hemilineages we have used from the FAFB-FlyWire dataset, related to Figure 7 — Neurons in each hemilineage are colored by their neuron-level transmitter predictions, hemilineage names given in the file name. Hemilineage labels for the FAFB-FlyWire dataset are fully reported in Schlegel et al.S2 [file mmc6.zip › chosen_hemilineages/FLAa2__fafb.png]

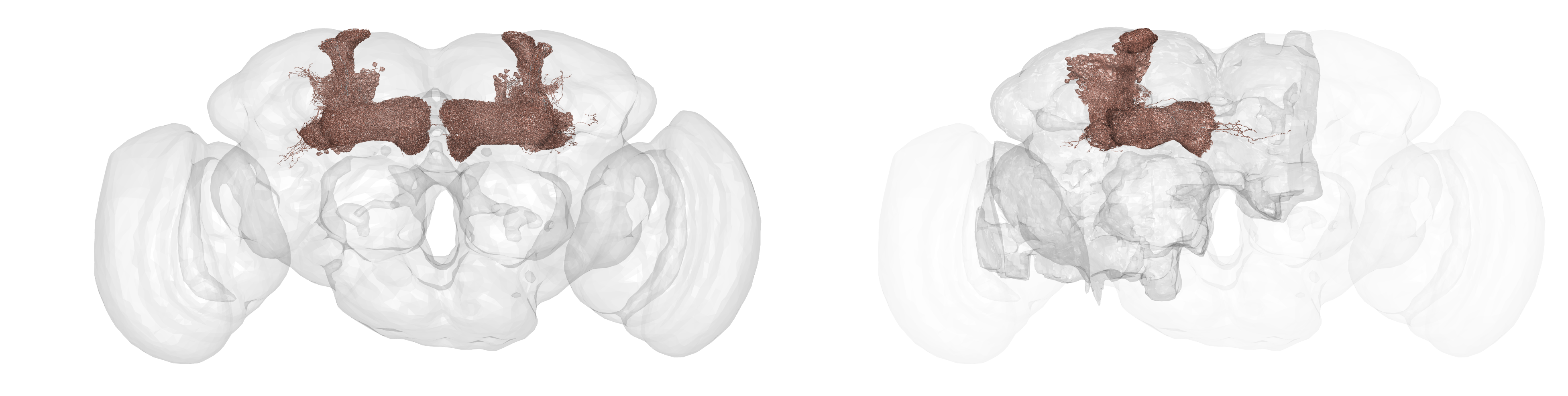

Supplement: Data S5. A .zip archive containing .png files depicting each of the 183 brain hemilineages we have used from the FAFB-FlyWire dataset, related to Figure 7 — Neurons in each hemilineage are colored by their neuron-level transmitter predictions, hemilineage names given in the file name. Hemilineage labels for the FAFB-FlyWire dataset are fully reported in Schlegel et al.S2 [file mmc6.zip › chosen_hemilineages/MBp2__fafb_hemibrain.png]

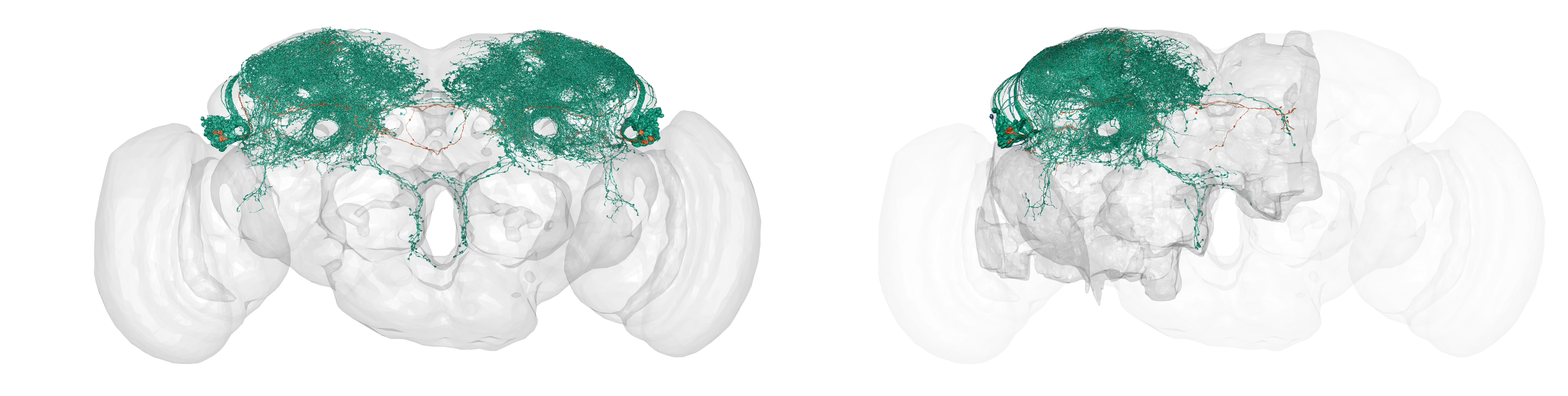

Supplement: Data S5. A .zip archive containing .png files depicting each of the 183 brain hemilineages we have used from the FAFB-FlyWire dataset, related to Figure 7 — Neurons in each hemilineage are colored by their neuron-level transmitter predictions, hemilineage names given in the file name. Hemilineage labels for the FAFB-FlyWire dataset are fully reported in Schlegel et al.S2 [file mmc6.zip › chosen_hemilineages/SLPav2__fafb_hemibrain.png]

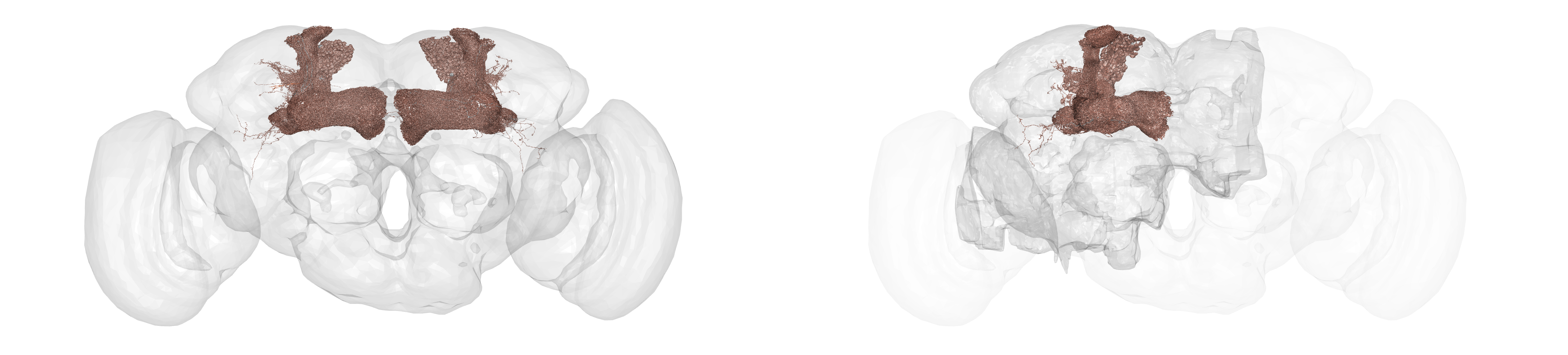

Supplement: Data S5. A .zip archive containing .png files depicting each of the 183 brain hemilineages we have used from the FAFB-FlyWire dataset, related to Figure 7 — Neurons in each hemilineage are colored by their neuron-level transmitter predictions, hemilineage names given in the file name. Hemilineage labels for the FAFB-FlyWire dataset are fully reported in Schlegel et al.S2 [file mmc6.zip › chosen_hemilineages/MBp1__fafb_hemibrain.png]

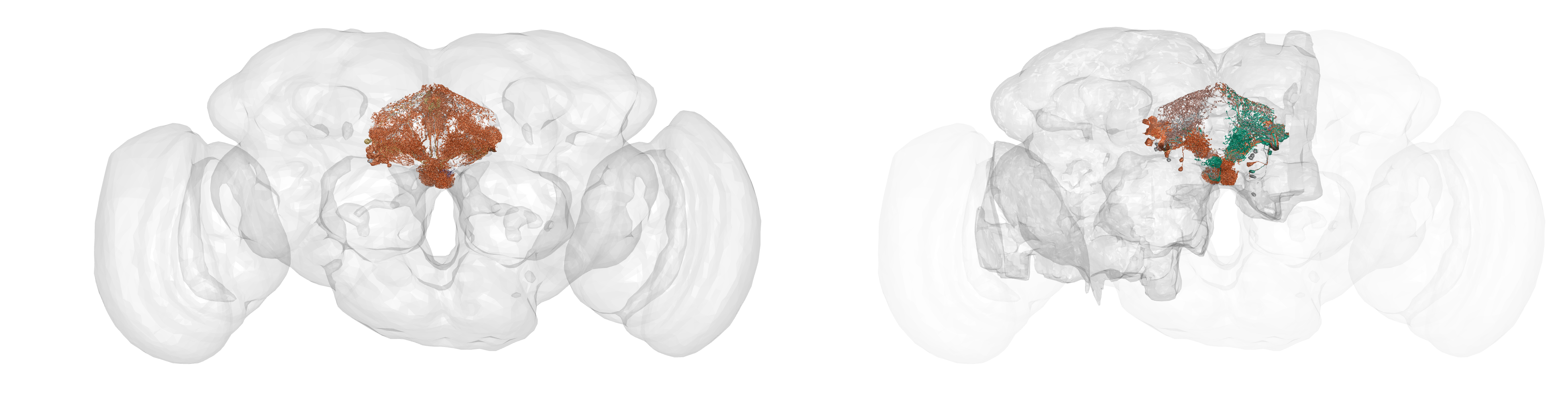

Supplement: Data S5. A .zip archive containing .png files depicting each of the 183 brain hemilineages we have used from the FAFB-FlyWire dataset, related to Figure 7 — Neurons in each hemilineage are colored by their neuron-level transmitter predictions, hemilineage names given in the file name. Hemilineage labels for the FAFB-FlyWire dataset are fully reported in Schlegel et al.S2 [file mmc6.zip › chosen_hemilineages/DM4_CX_p__fafb_hemibrain.png]

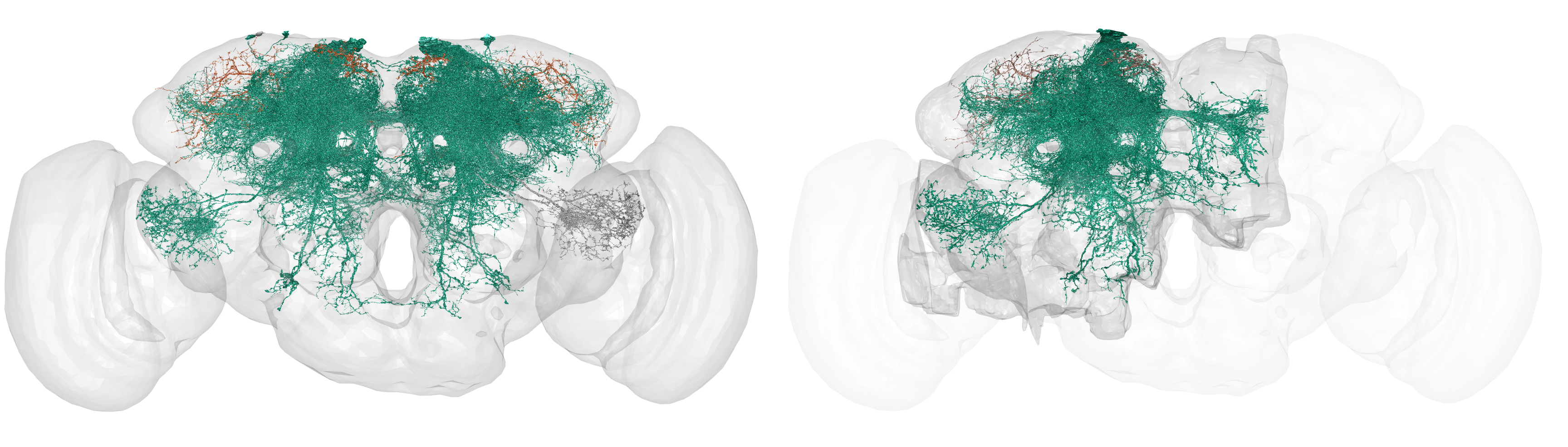

Supplement: Data S5. A .zip archive containing .png files depicting each of the 183 brain hemilineages we have used from the FAFB-FlyWire dataset, related to Figure 7 — Neurons in each hemilineage are colored by their neuron-level transmitter predictions, hemilineage names given in the file name. Hemilineage labels for the FAFB-FlyWire dataset are fully reported in Schlegel et al.S2 [file mmc6.zip › chosen_hemilineages/SMPp&v1_posterior__fafb_hemibrain.png]

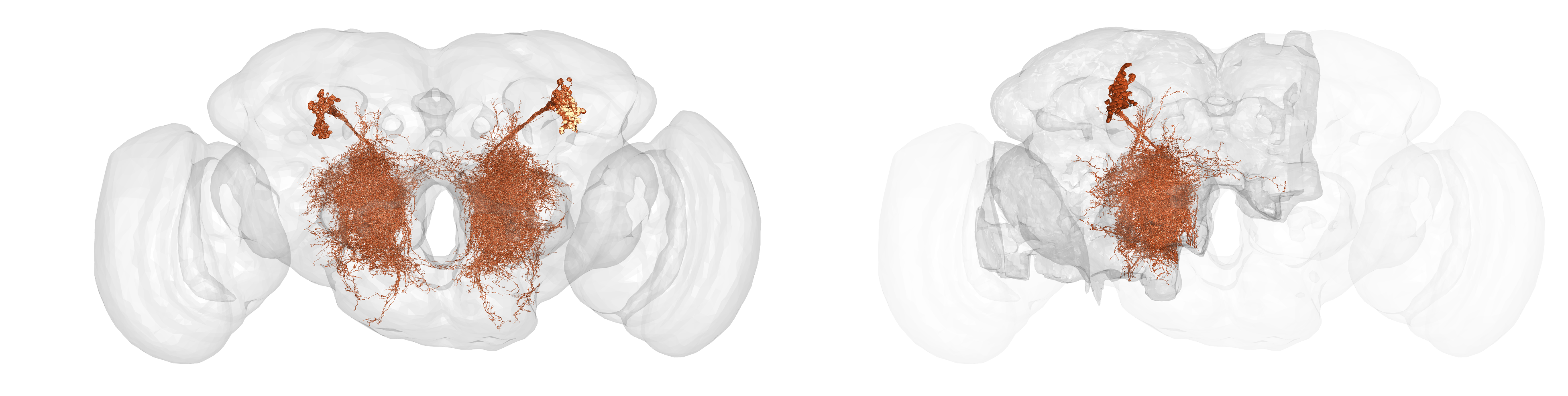

Supplement: Data S5. A .zip archive containing .png files depicting each of the 183 brain hemilineages we have used from the FAFB-FlyWire dataset, related to Figure 7 — Neurons in each hemilineage are colored by their neuron-level transmitter predictions, hemilineage names given in the file name. Hemilineage labels for the FAFB-FlyWire dataset are fully reported in Schlegel et al.S2 [file mmc6.zip › chosen_hemilineages/AOTUv1_ventral__fafb_hemibrain.png]

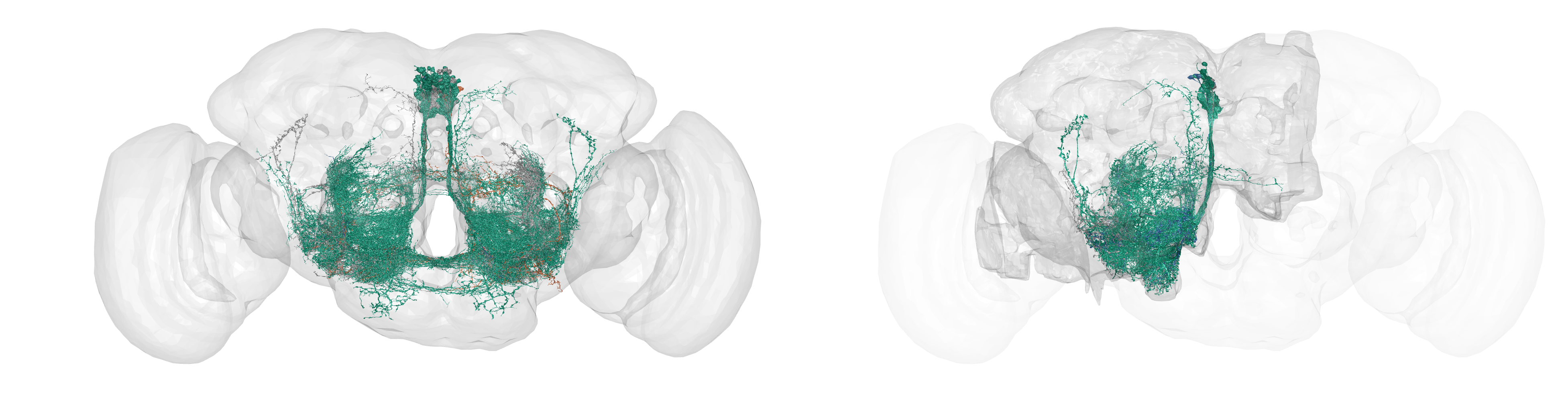

Supplement: Data S5. A .zip archive containing .png files depicting each of the 183 brain hemilineages we have used from the FAFB-FlyWire dataset, related to Figure 7 — Neurons in each hemilineage are colored by their neuron-level transmitter predictions, hemilineage names given in the file name. Hemilineage labels for the FAFB-FlyWire dataset are fully reported in Schlegel et al.S2 [file mmc6.zip › chosen_hemilineages/DM1_antero_ventral__fafb_hemibrain.png]

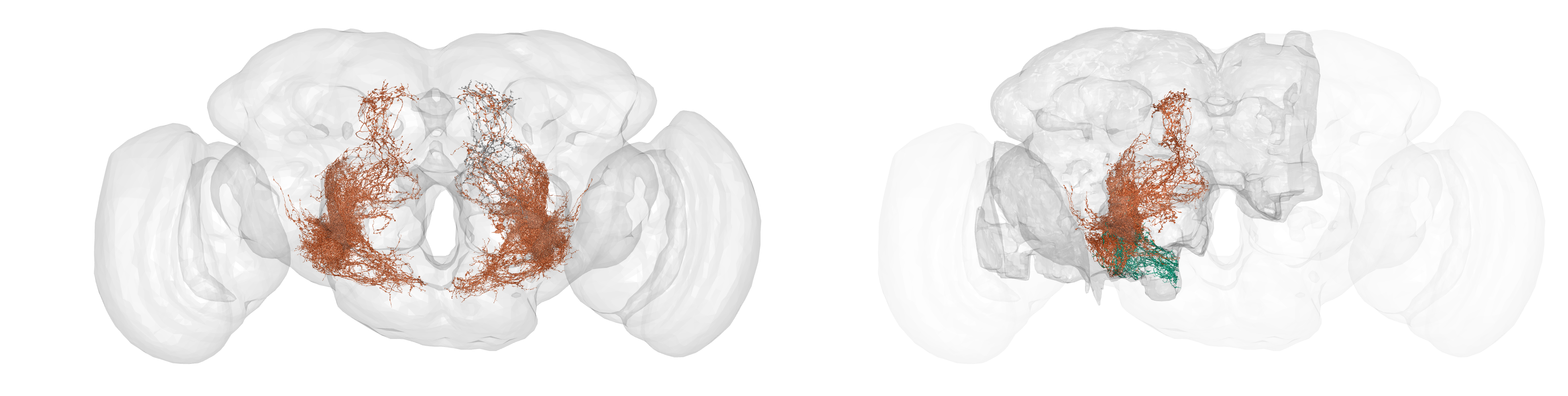

Supplement: Data S5. A .zip archive containing .png files depicting each of the 183 brain hemilineages we have used from the FAFB-FlyWire dataset, related to Figure 7 — Neurons in each hemilineage are colored by their neuron-level transmitter predictions, hemilineage names given in the file name. Hemilineage labels for the FAFB-FlyWire dataset are fully reported in Schlegel et al.S2 [file mmc6.zip › chosen_hemilineages/DM5_ventral__fafb_hemibrain.png]
