# Supplementary figures and images for: Neurotransmitter classification from electron microscopy images at synaptic sites in Drosophila melanogaster (part 1 of 2)
Source: Cell. 2024 May 9;187(10):2574–2594.e23. doi: 10.1016/j.cell.2024.03.016 (PMC11106717; doi:10.1016/j.cell.2024.03.016)

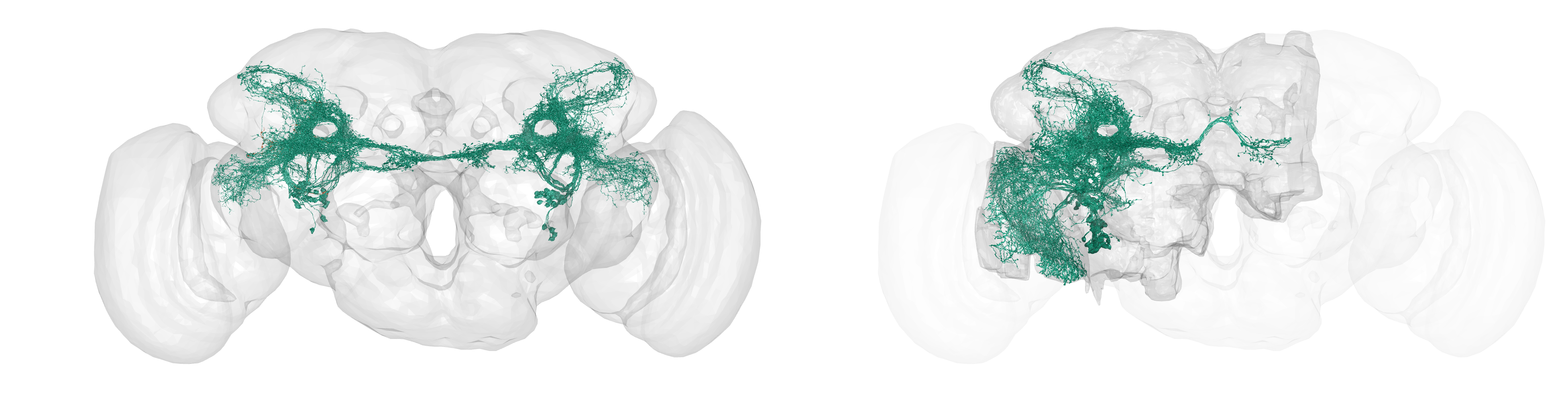

Supplement: Data S5. A .zip archive containing .png files depicting each of the 183 brain hemilineages we have used from the FAFB-FlyWire dataset, related to Figure 7 — Neurons in each hemilineage are colored by their neuron-level transmitter predictions, hemilineage names given in the file name. Hemilineage labels for the FAFB-FlyWire dataset are fully reported in Schlegel et al.S2 [file mmc6.zip › chosen_hemilineages/VPNp&v1_ventral__fafb_hemibrain.png]

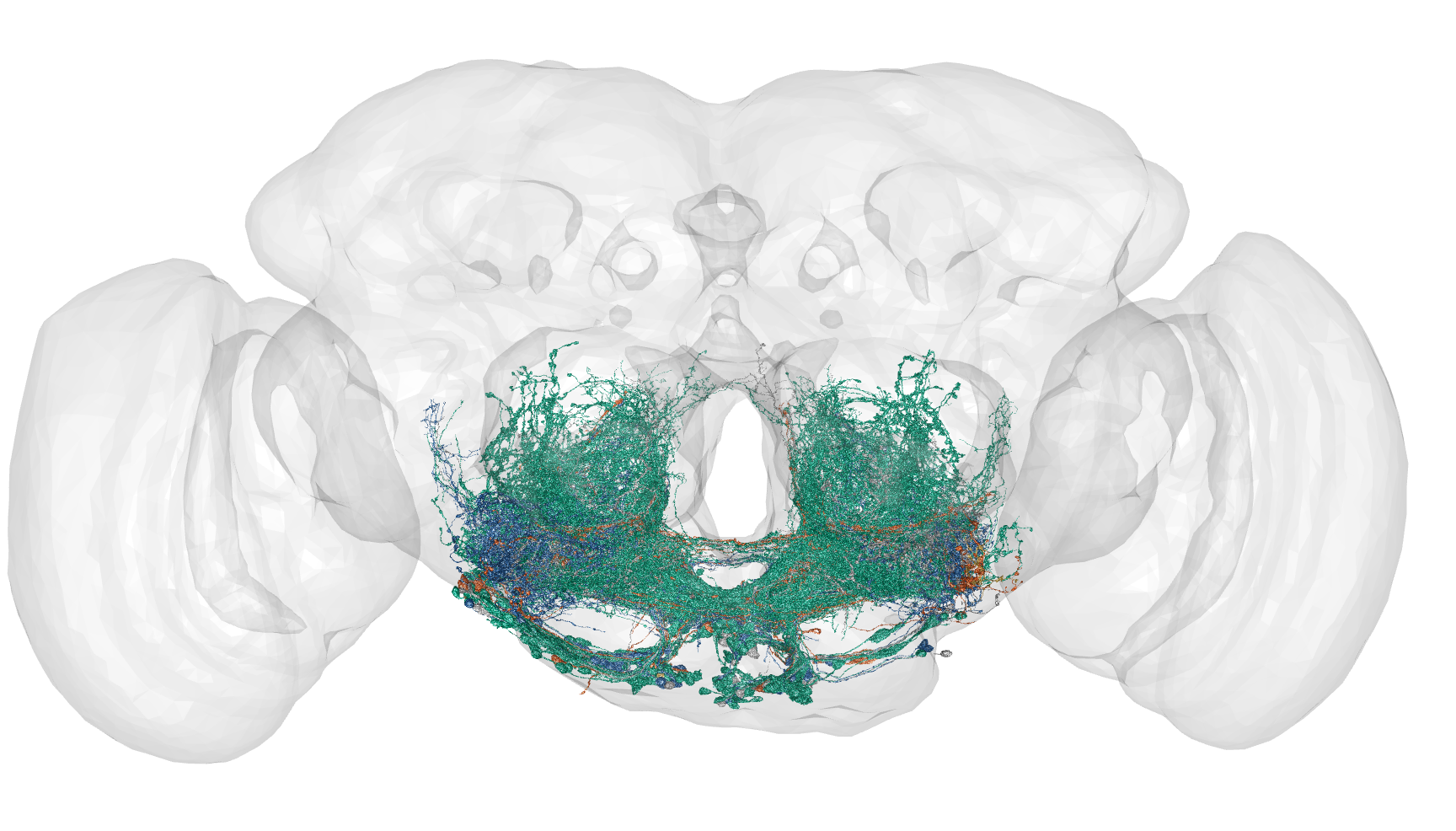

Supplement: Data S5. A .zip archive containing .png files depicting each of the 183 brain hemilineages we have used from the FAFB-FlyWire dataset, related to Figure 7 — Neurons in each hemilineage are colored by their neuron-level transmitter predictions, hemilineage names given in the file name. Hemilineage labels for the FAFB-FlyWire dataset are fully reported in Schlegel et al.S2 [file mmc6.zip › chosen_hemilineages/LB3__fafb.png]

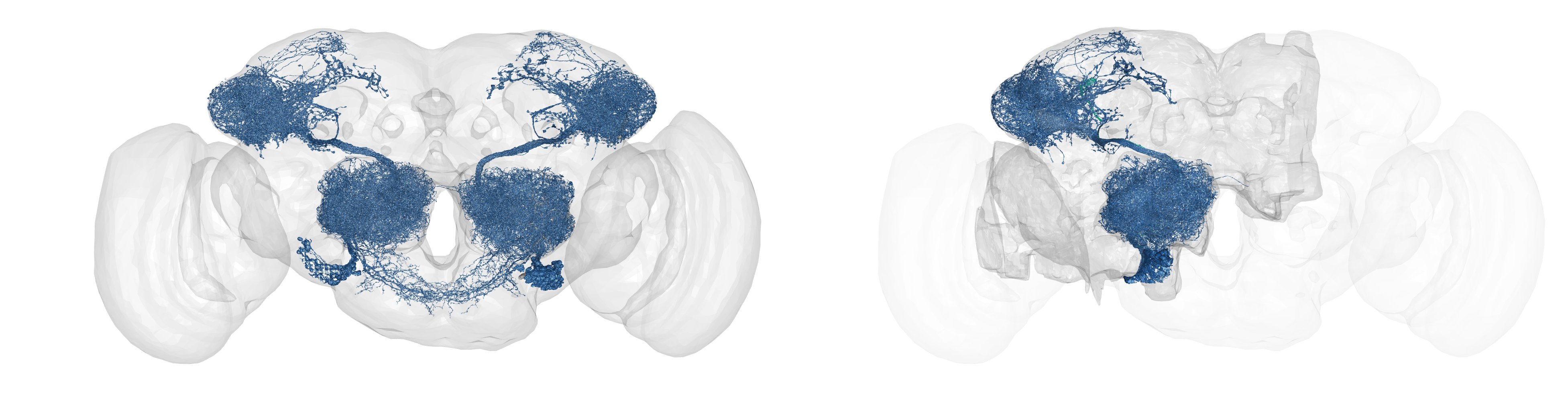

Supplement: Data S5. A .zip archive containing .png files depicting each of the 183 brain hemilineages we have used from the FAFB-FlyWire dataset, related to Figure 7 — Neurons in each hemilineage are colored by their neuron-level transmitter predictions, hemilineage names given in the file name. Hemilineage labels for the FAFB-FlyWire dataset are fully reported in Schlegel et al.S2 [file mmc6.zip › chosen_hemilineages/ALv1__fafb_hemibrain.png]

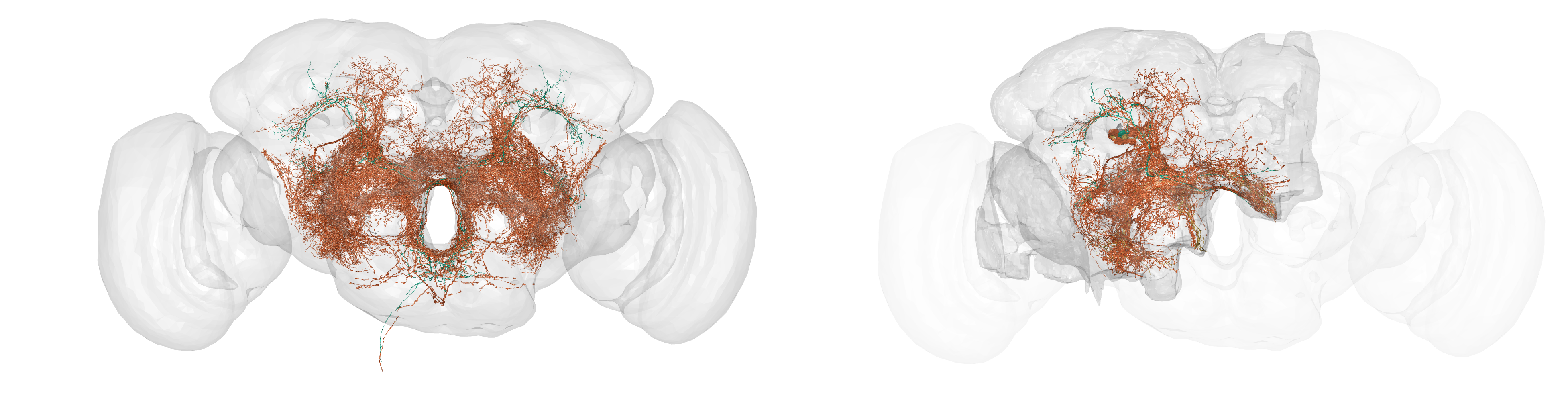

Supplement: Data S5. A .zip archive containing .png files depicting each of the 183 brain hemilineages we have used from the FAFB-FlyWire dataset, related to Figure 7 — Neurons in each hemilineage are colored by their neuron-level transmitter predictions, hemilineage names given in the file name. Hemilineage labels for the FAFB-FlyWire dataset are fully reported in Schlegel et al.S2 [file mmc6.zip › chosen_hemilineages/DM6_central1__fafb_hemibrain.png]

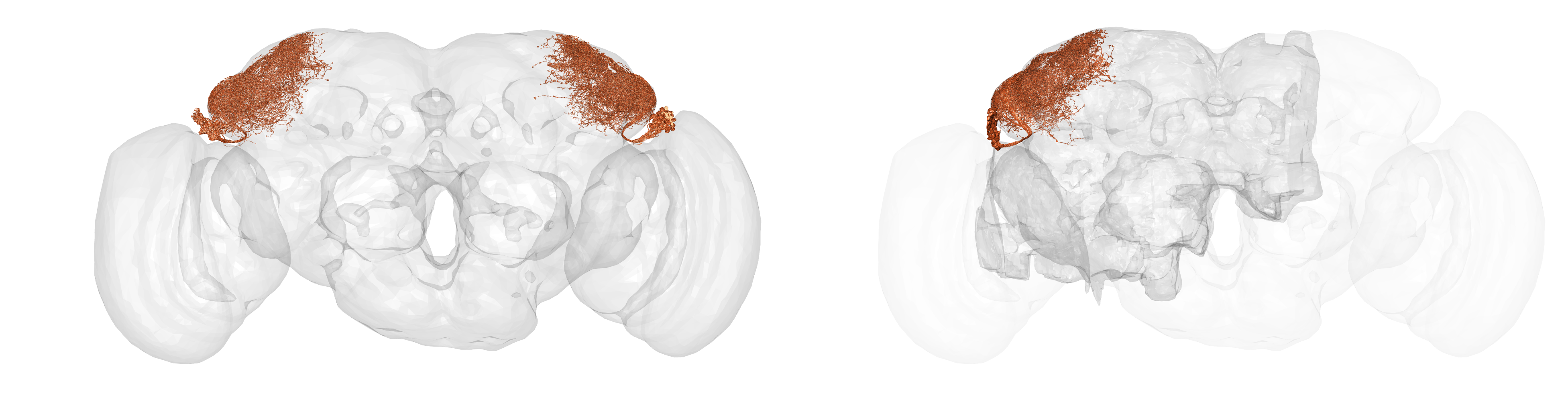

Supplement: Data S5. A .zip archive containing .png files depicting each of the 183 brain hemilineages we have used from the FAFB-FlyWire dataset, related to Figure 7 — Neurons in each hemilineage are colored by their neuron-level transmitter predictions, hemilineage names given in the file name. Hemilineage labels for the FAFB-FlyWire dataset are fully reported in Schlegel et al.S2 [file mmc6.zip › chosen_hemilineages/VPNl&d1_dorsal__fafb_hemibrain.png]

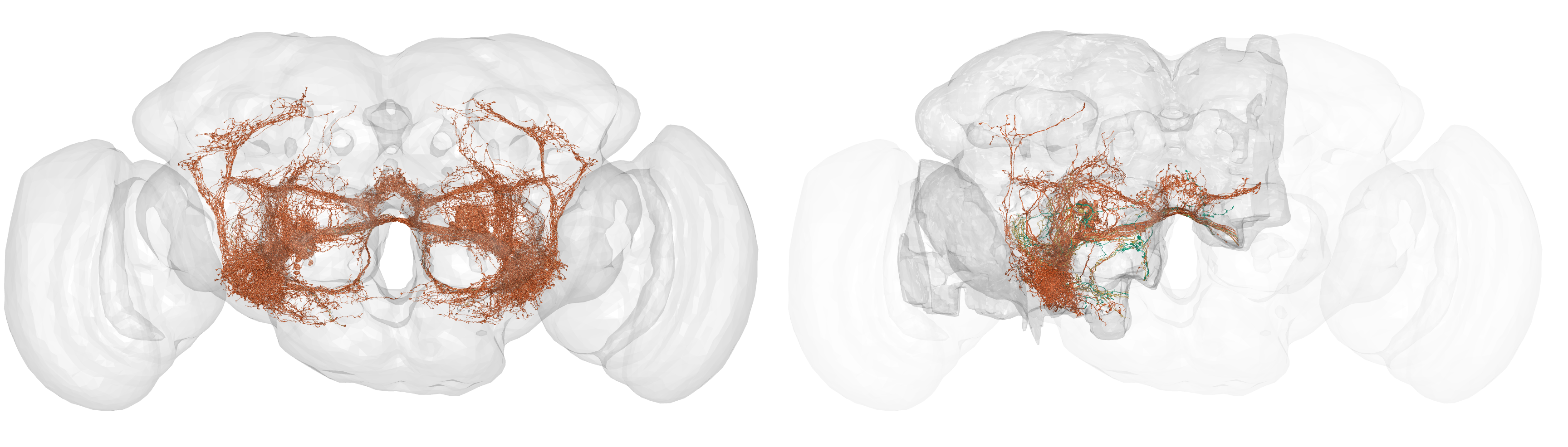

Supplement: Data S5. A .zip archive containing .png files depicting each of the 183 brain hemilineages we have used from the FAFB-FlyWire dataset, related to Figure 7 — Neurons in each hemilineage are colored by their neuron-level transmitter predictions, hemilineage names given in the file name. Hemilineage labels for the FAFB-FlyWire dataset are fully reported in Schlegel et al.S2 [file mmc6.zip › chosen_hemilineages/DM6_ventral__fafb_hemibrain.png]

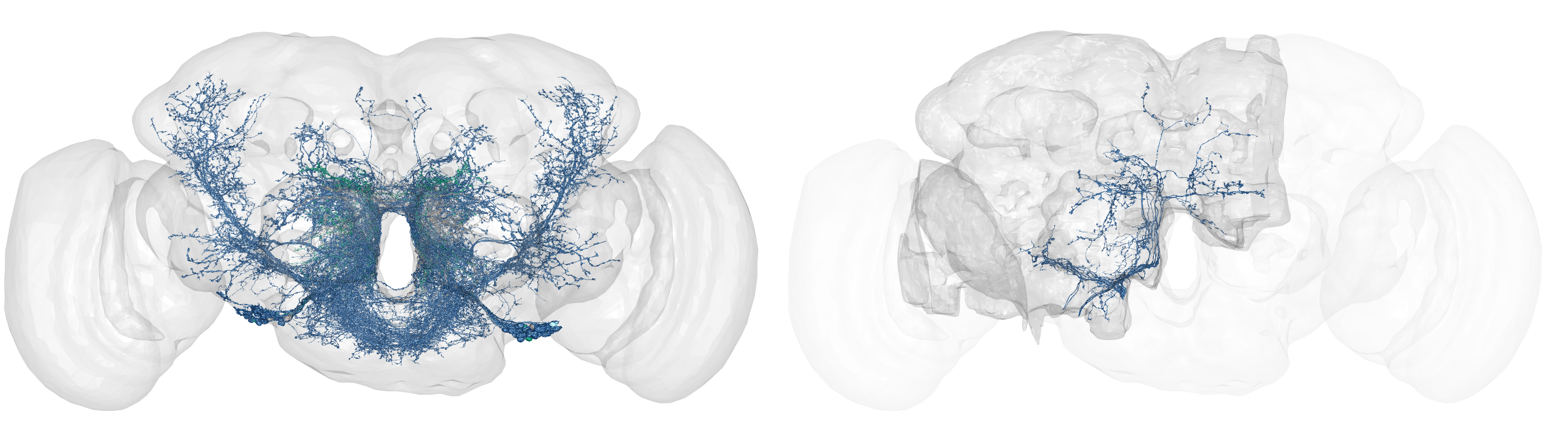

Supplement: Data S5. A .zip archive containing .png files depicting each of the 183 brain hemilineages we have used from the FAFB-FlyWire dataset, related to Figure 7 — Neurons in each hemilineage are colored by their neuron-level transmitter predictions, hemilineage names given in the file name. Hemilineage labels for the FAFB-FlyWire dataset are fully reported in Schlegel et al.S2 [file mmc6.zip › chosen_hemilineages/VESa1__fafb_hemibrain.png]

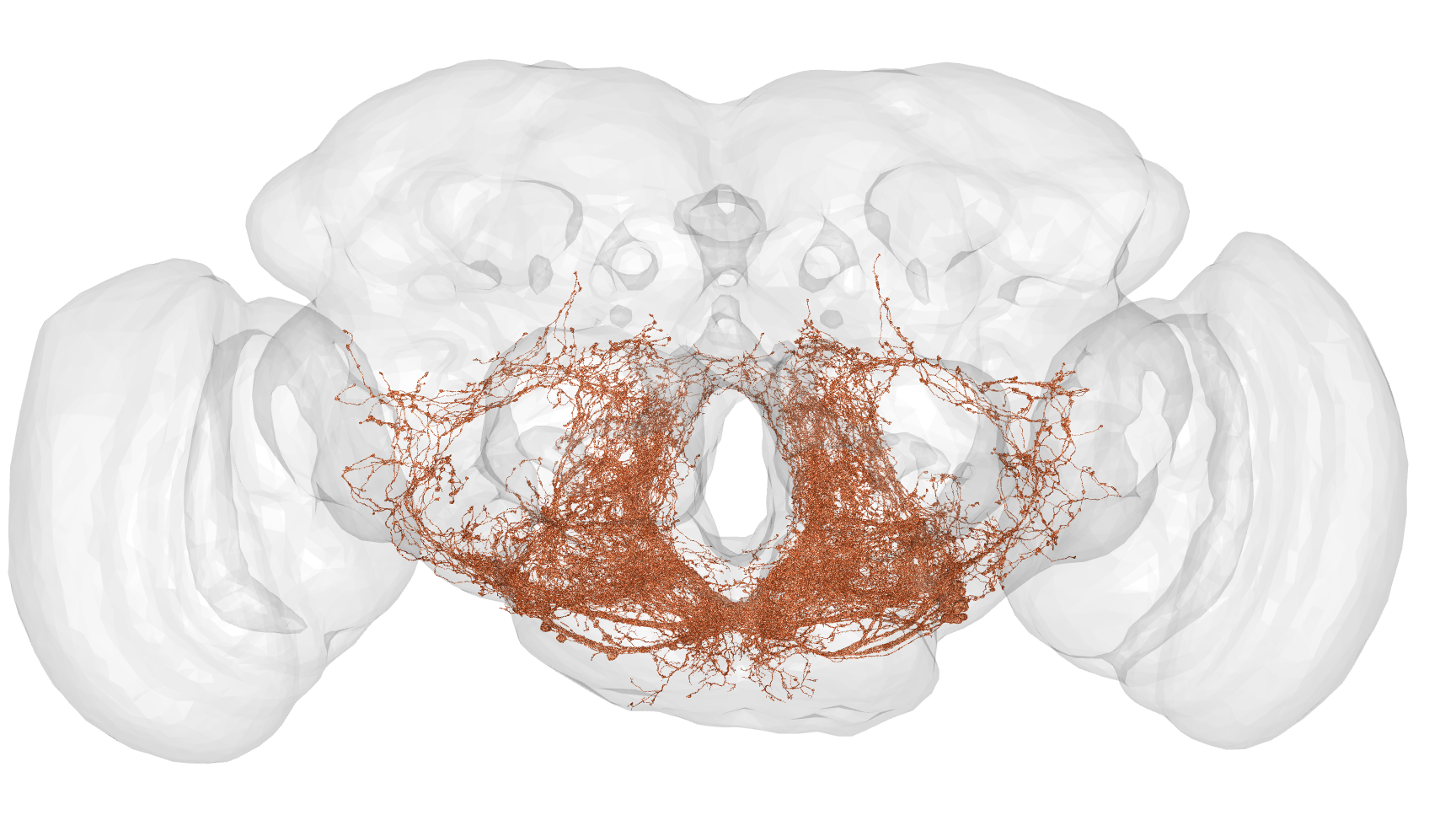

Supplement: Data S5. A .zip archive containing .png files depicting each of the 183 brain hemilineages we have used from the FAFB-FlyWire dataset, related to Figure 7 — Neurons in each hemilineage are colored by their neuron-level transmitter predictions, hemilineage names given in the file name. Hemilineage labels for the FAFB-FlyWire dataset are fully reported in Schlegel et al.S2 [file mmc6.zip › chosen_hemilineages/LB19__fafb.png]

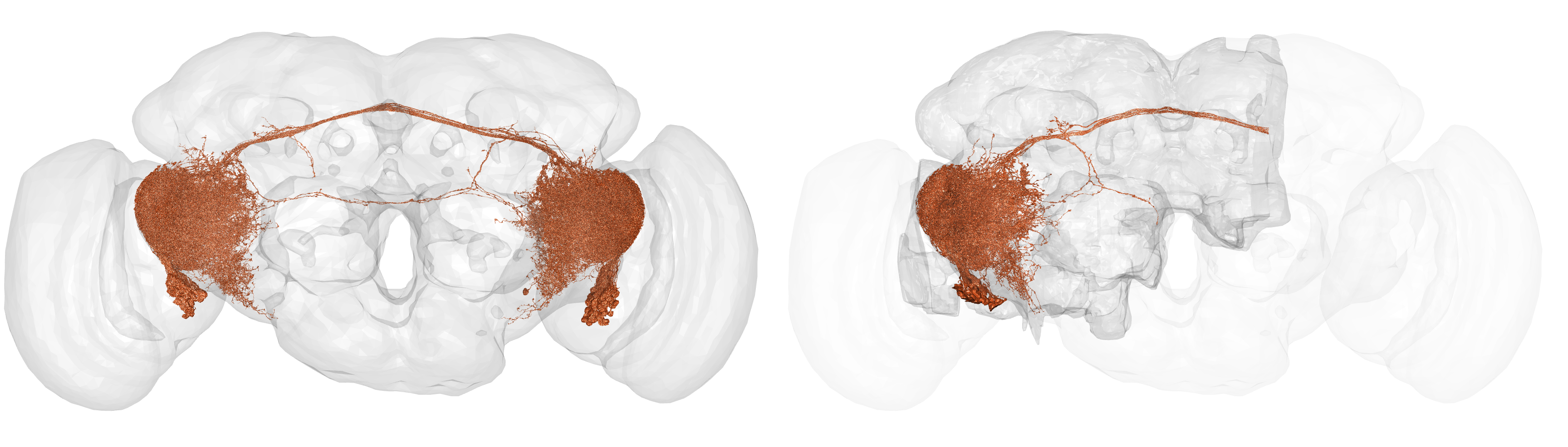

Supplement: Data S5. A .zip archive containing .png files depicting each of the 183 brain hemilineages we have used from the FAFB-FlyWire dataset, related to Figure 7 — Neurons in each hemilineage are colored by their neuron-level transmitter predictions, hemilineage names given in the file name. Hemilineage labels for the FAFB-FlyWire dataset are fully reported in Schlegel et al.S2 [file mmc6.zip › chosen_hemilineages/VLPl&p1_lateral__fafb_hemibrain.png]

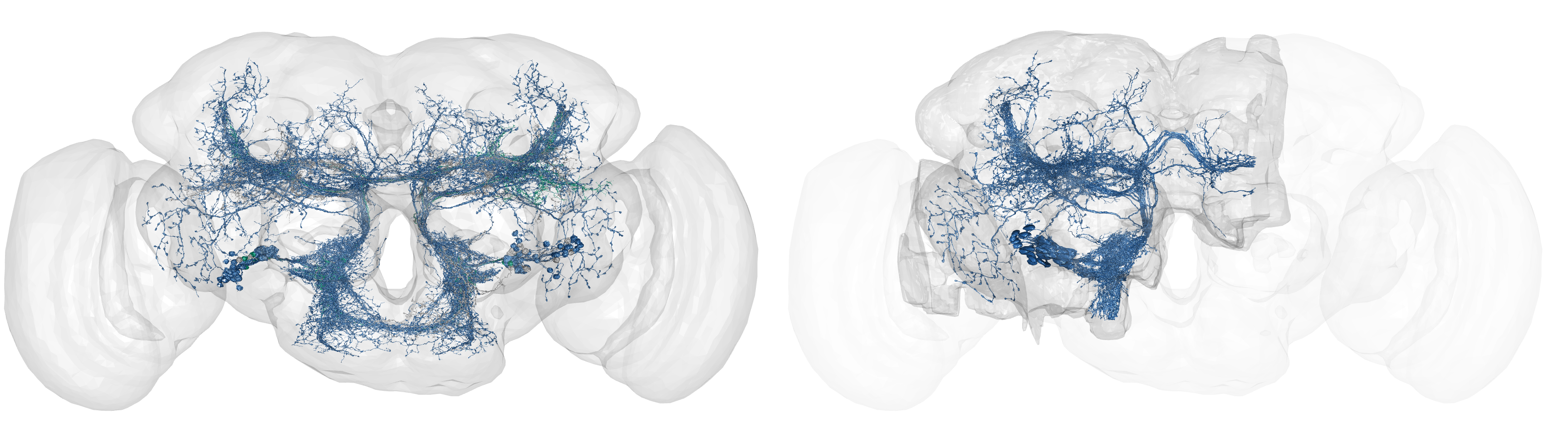

Supplement: Data S5. A .zip archive containing .png files depicting each of the 183 brain hemilineages we have used from the FAFB-FlyWire dataset, related to Figure 7 — Neurons in each hemilineage are colored by their neuron-level transmitter predictions, hemilineage names given in the file name. Hemilineage labels for the FAFB-FlyWire dataset are fully reported in Schlegel et al.S2 [file mmc6.zip › chosen_hemilineages/VESa2__fafb_hemibrain.png]

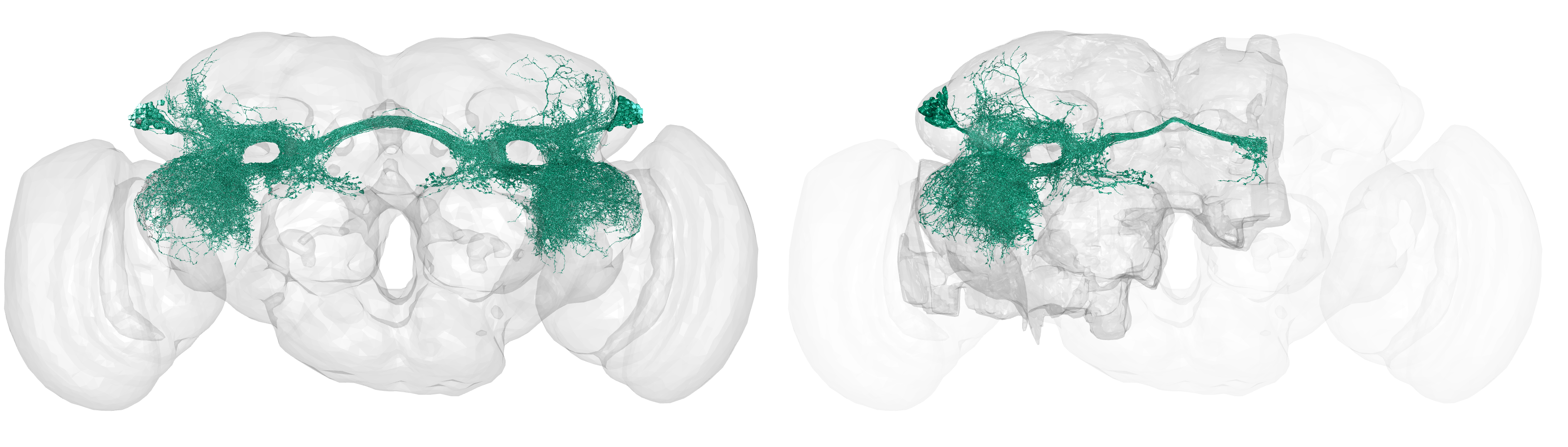

Supplement: Data S5. A .zip archive containing .png files depicting each of the 183 brain hemilineages we have used from the FAFB-FlyWire dataset, related to Figure 7 — Neurons in each hemilineage are colored by their neuron-level transmitter predictions, hemilineage names given in the file name. Hemilineage labels for the FAFB-FlyWire dataset are fully reported in Schlegel et al.S2 [file mmc6.zip › chosen_hemilineages/LHl2_ventral__fafb_hemibrain.png]

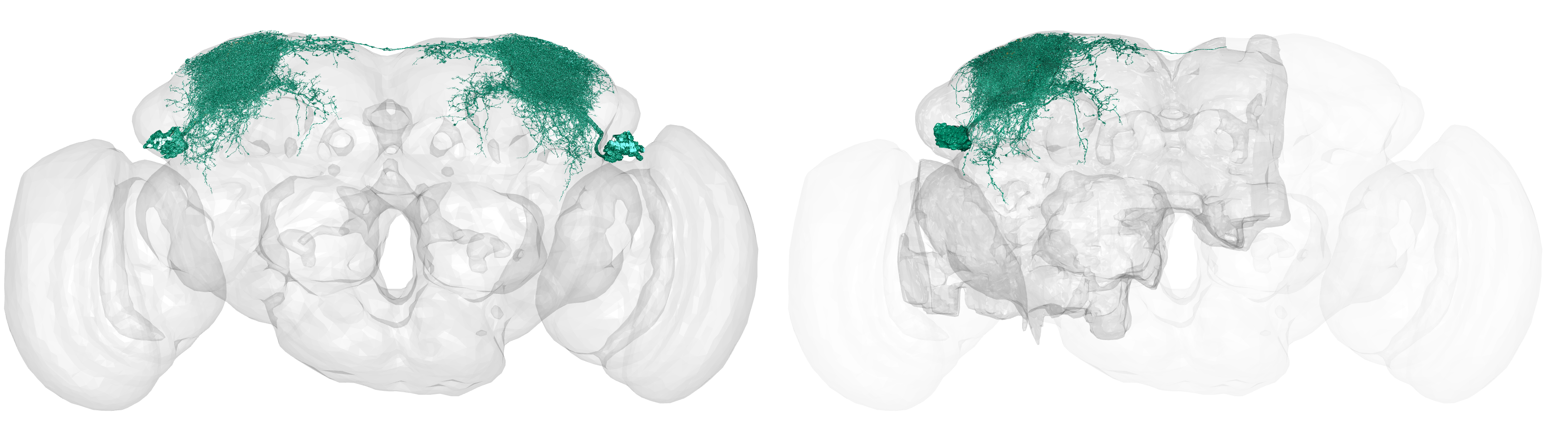

Supplement: Data S5. A .zip archive containing .png files depicting each of the 183 brain hemilineages we have used from the FAFB-FlyWire dataset, related to Figure 7 — Neurons in each hemilineage are colored by their neuron-level transmitter predictions, hemilineage names given in the file name. Hemilineage labels for the FAFB-FlyWire dataset are fully reported in Schlegel et al.S2 [file mmc6.zip › chosen_hemilineages/SLPal3_and_SLPal4_dorsal__fafb_hemibrain.png]

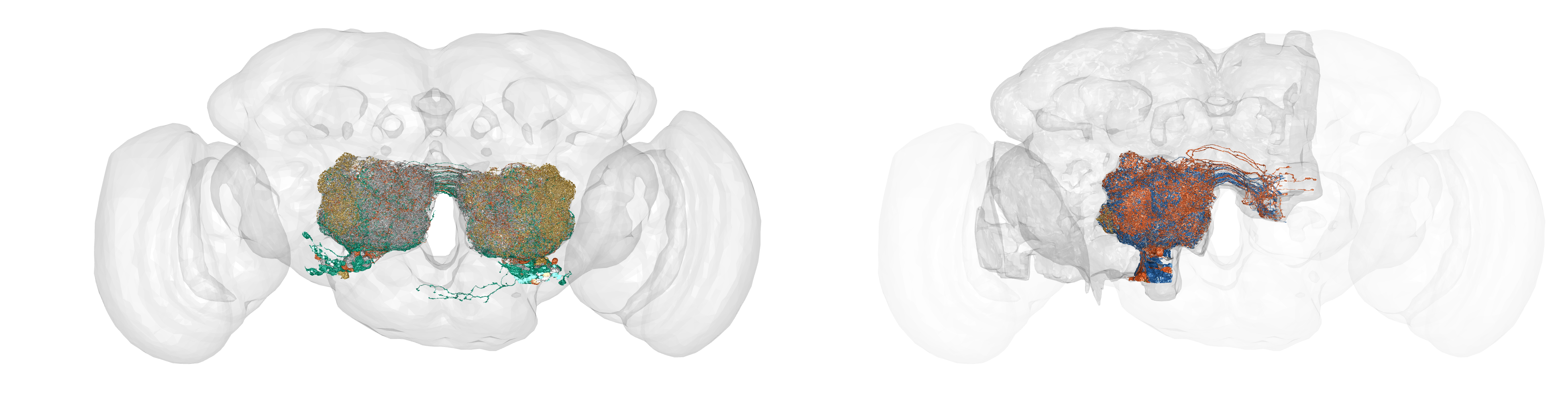

Supplement: Data S5. A .zip archive containing .png files depicting each of the 183 brain hemilineages we have used from the FAFB-FlyWire dataset, related to Figure 7 — Neurons in each hemilineage are colored by their neuron-level transmitter predictions, hemilineage names given in the file name. Hemilineage labels for the FAFB-FlyWire dataset are fully reported in Schlegel et al.S2 [file mmc6.zip › chosen_hemilineages/ALv2__fafb_hemibrain.png]

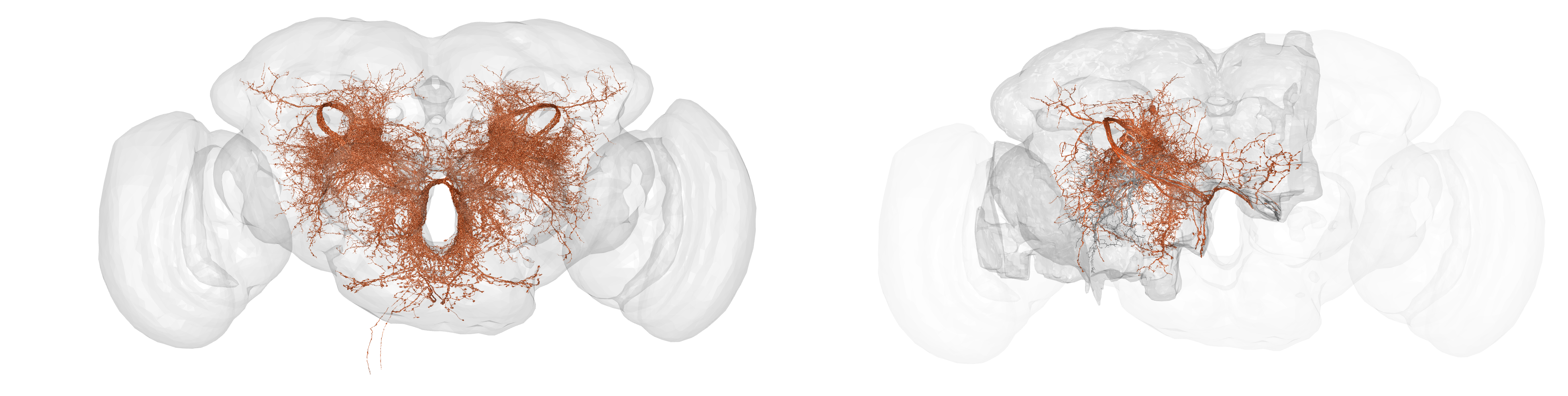

Supplement: Data S5. A .zip archive containing .png files depicting each of the 183 brain hemilineages we have used from the FAFB-FlyWire dataset, related to Figure 7 — Neurons in each hemilineage are colored by their neuron-level transmitter predictions, hemilineage names given in the file name. Hemilineage labels for the FAFB-FlyWire dataset are fully reported in Schlegel et al.S2 [file mmc6.zip › chosen_hemilineages/DM6_central2__fafb_hemibrain.png]

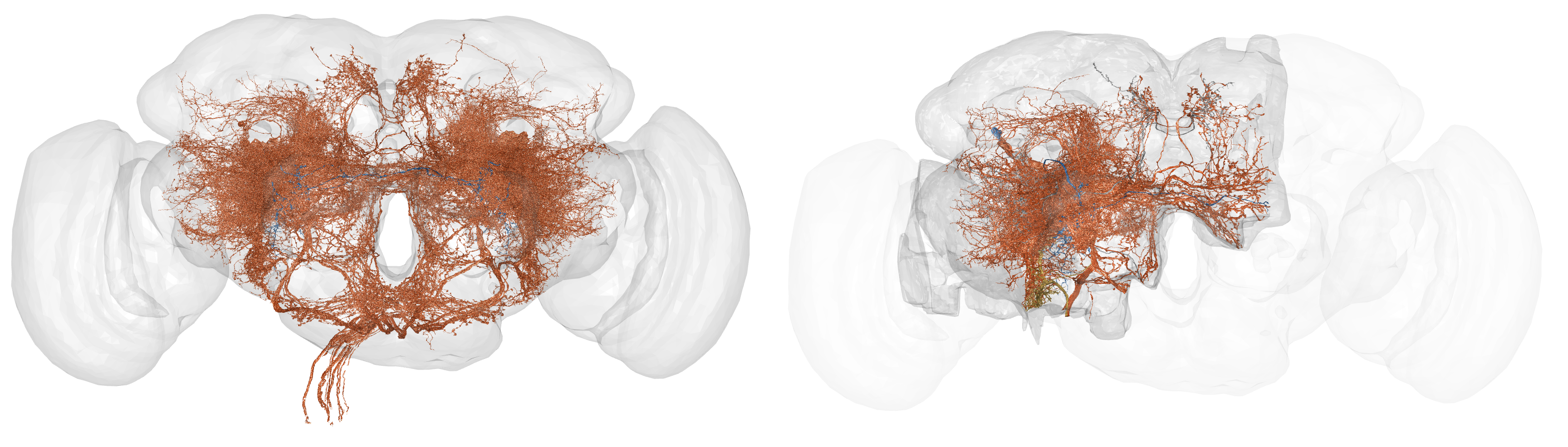

Supplement: Data S5. A .zip archive containing .png files depicting each of the 183 brain hemilineages we have used from the FAFB-FlyWire dataset, related to Figure 7 — Neurons in each hemilineage are colored by their neuron-level transmitter predictions, hemilineage names given in the file name. Hemilineage labels for the FAFB-FlyWire dataset are fully reported in Schlegel et al.S2 [file mmc6.zip › chosen_hemilineages/SMPpv2_ventral__fafb_hemibrain.png]

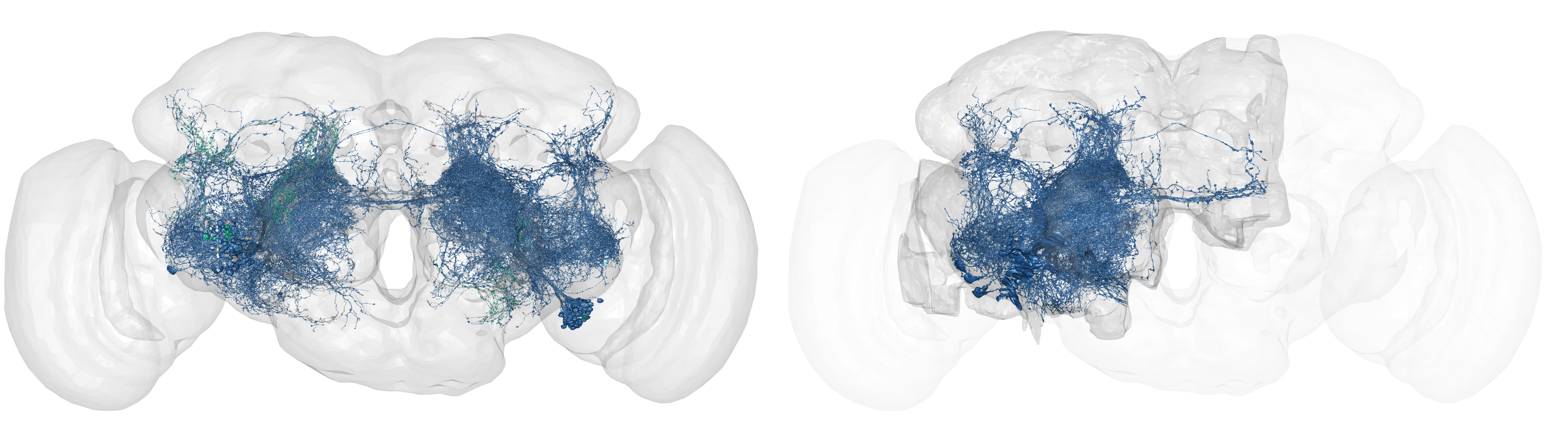

Supplement: Data S5. A .zip archive containing .png files depicting each of the 183 brain hemilineages we have used from the FAFB-FlyWire dataset, related to Figure 7 — Neurons in each hemilineage are colored by their neuron-level transmitter predictions, hemilineage names given in the file name. Hemilineage labels for the FAFB-FlyWire dataset are fully reported in Schlegel et al.S2 [file mmc6.zip › chosen_hemilineages/LALa1_anterior__fafb_hemibrain.png]

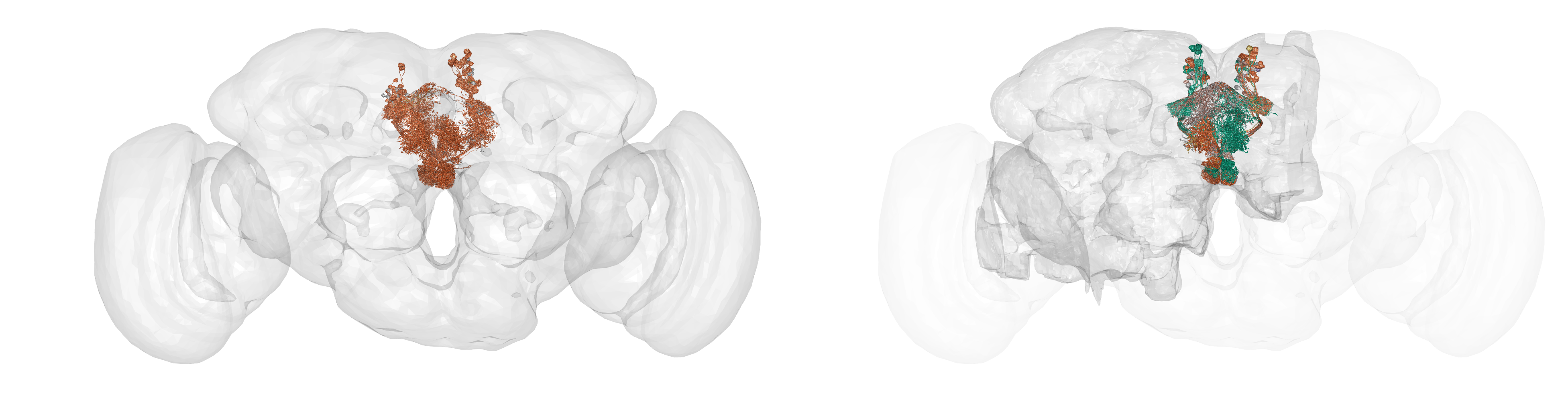

Supplement: Data S5. A .zip archive containing .png files depicting each of the 183 brain hemilineages we have used from the FAFB-FlyWire dataset, related to Figure 7 — Neurons in each hemilineage are colored by their neuron-level transmitter predictions, hemilineage names given in the file name. Hemilineage labels for the FAFB-FlyWire dataset are fully reported in Schlegel et al.S2 [file mmc6.zip › chosen_hemilineages/DM2_CX_v__fafb_hemibrain.png]

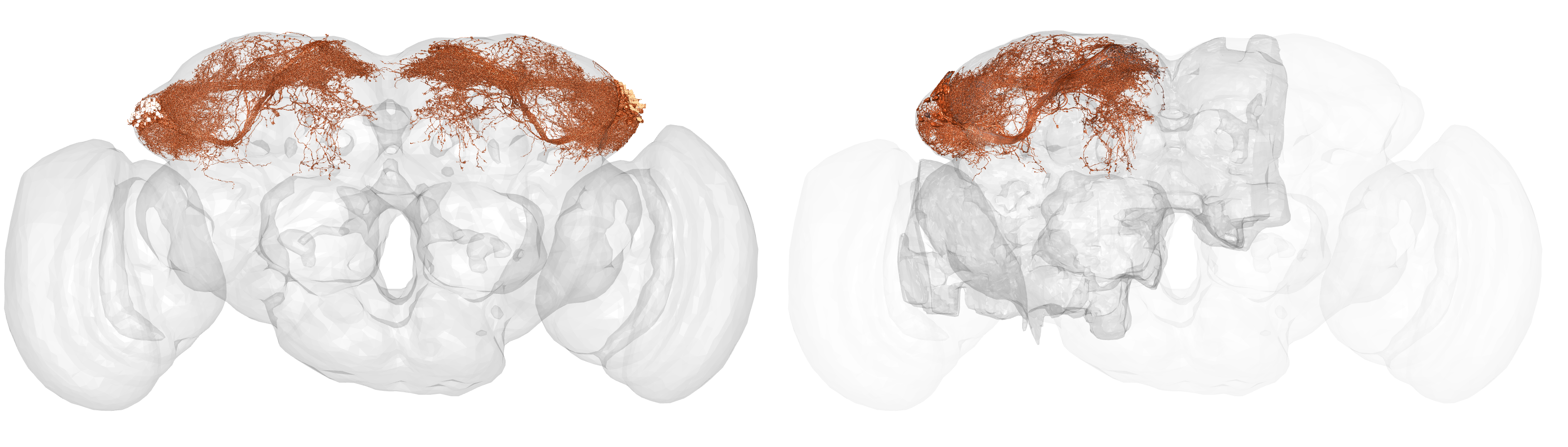

Supplement: Data S5. A .zip archive containing .png files depicting each of the 183 brain hemilineages we have used from the FAFB-FlyWire dataset, related to Figure 7 — Neurons in each hemilineage are colored by their neuron-level transmitter predictions, hemilineage names given in the file name. Hemilineage labels for the FAFB-FlyWire dataset are fully reported in Schlegel et al.S2 [file mmc6.zip › chosen_hemilineages/LHl2_dorsal__fafb_hemibrain.png]

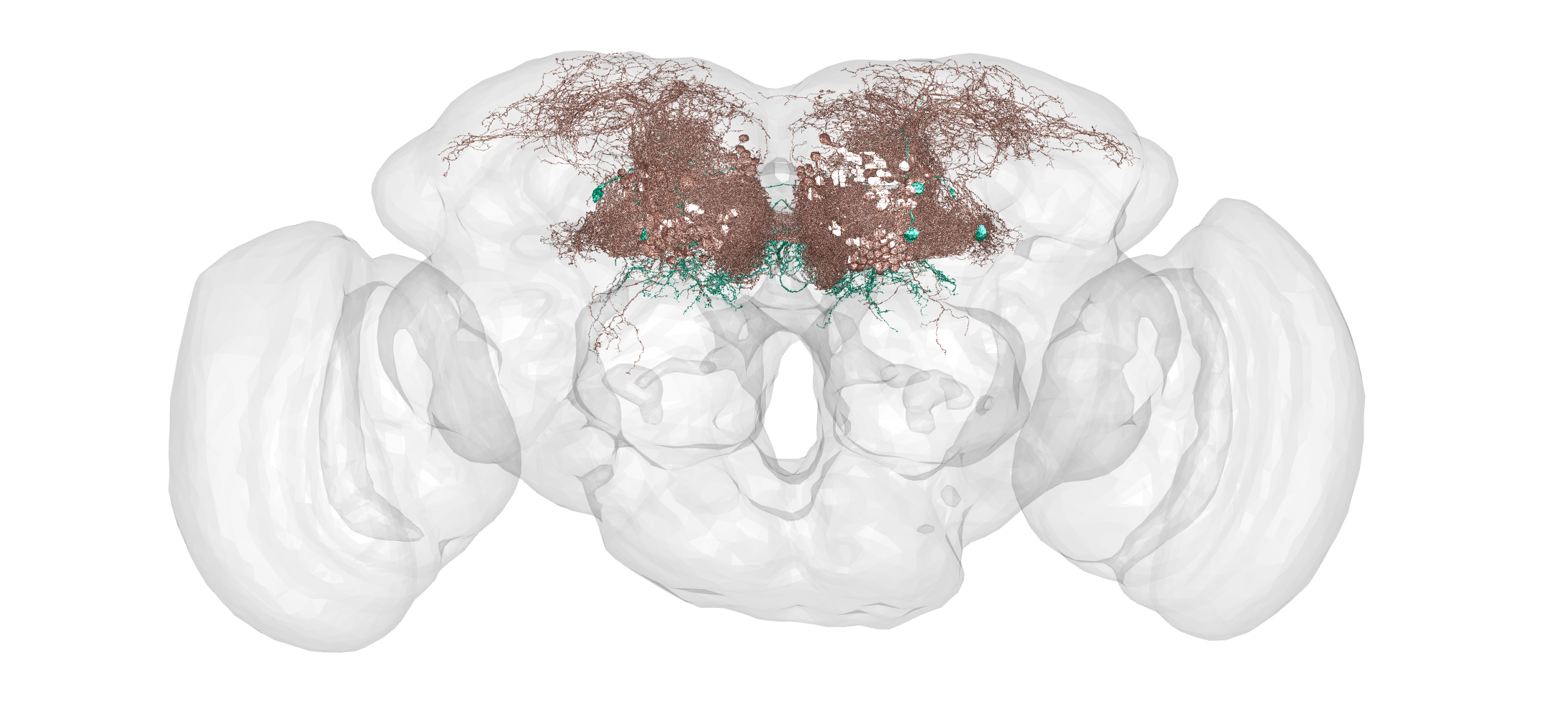

Supplement: Data S5. A .zip archive containing .png files depicting each of the 183 brain hemilineages we have used from the FAFB-FlyWire dataset, related to Figure 7 — Neurons in each hemilineage are colored by their neuron-level transmitter predictions, hemilineage names given in the file name. Hemilineage labels for the FAFB-FlyWire dataset are fully reported in Schlegel et al.S2 [file mmc6.zip › chosen_hemilineages/CREa1_dorsal__fafb.png]

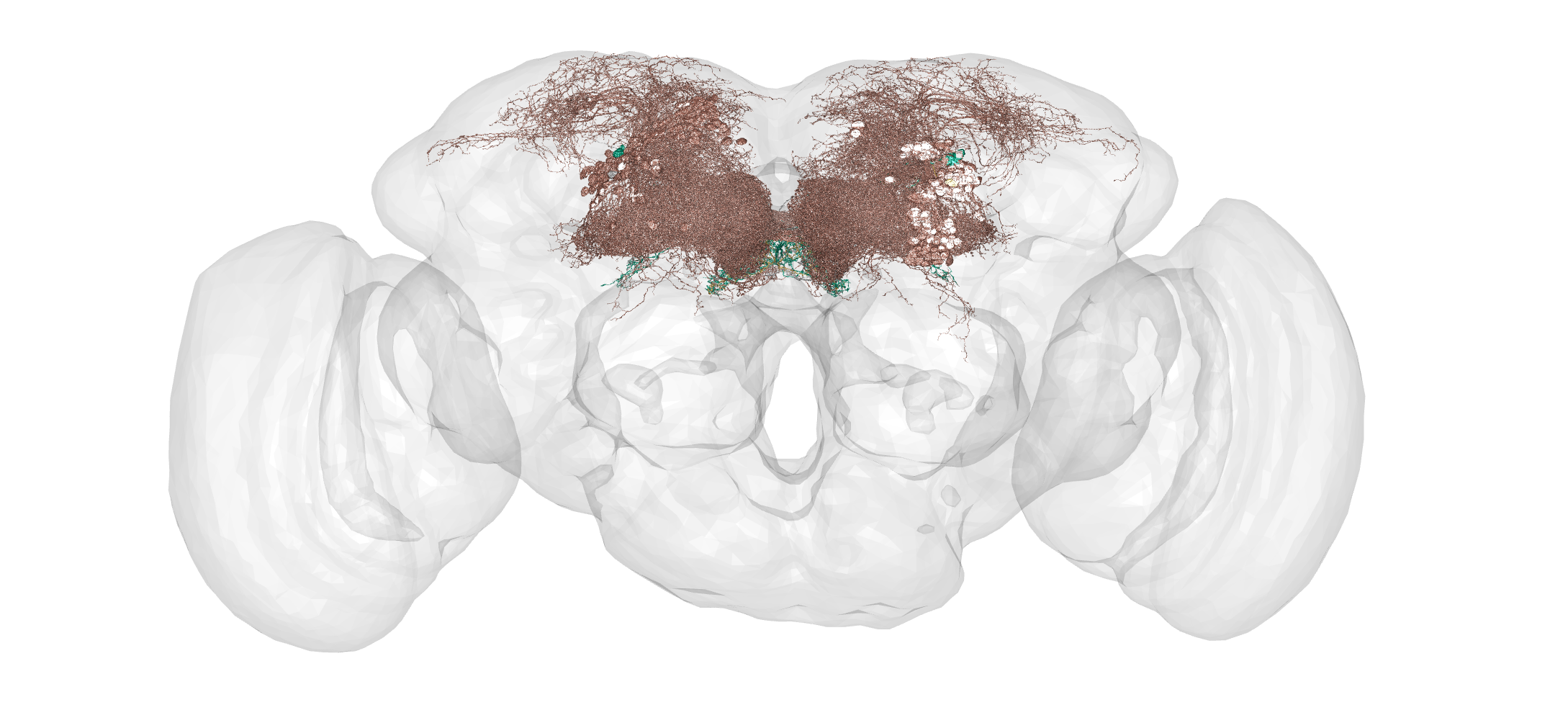

Supplement: Data S5. A .zip archive containing .png files depicting each of the 183 brain hemilineages we have used from the FAFB-FlyWire dataset, related to Figure 7 — Neurons in each hemilineage are colored by their neuron-level transmitter predictions, hemilineage names given in the file name. Hemilineage labels for the FAFB-FlyWire dataset are fully reported in Schlegel et al.S2 [file mmc6.zip › chosen_hemilineages/CREa2_medial__fafb.png]

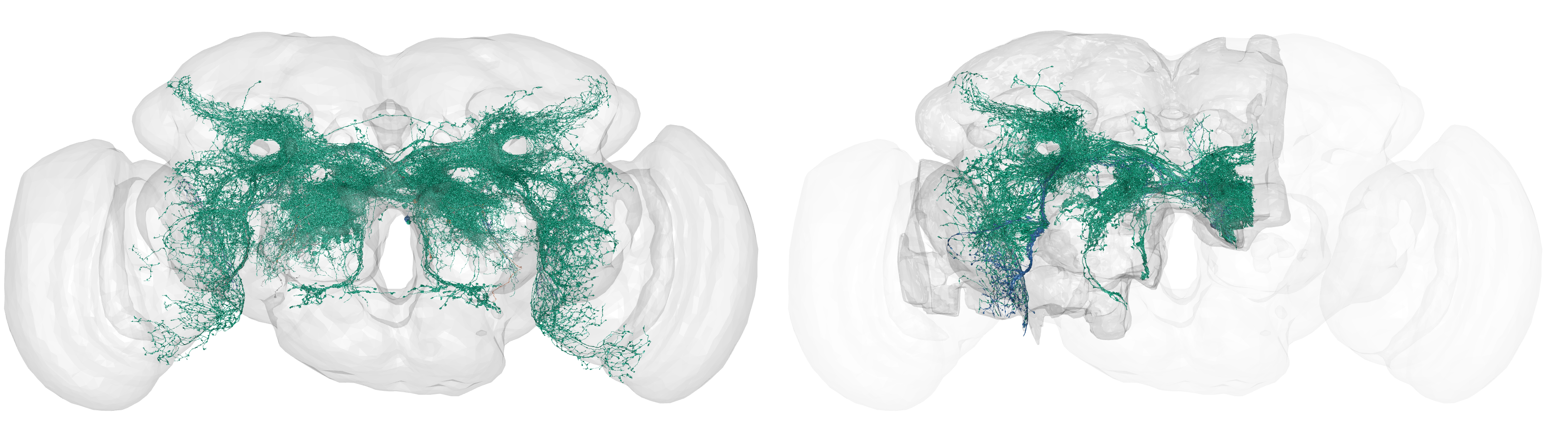

Supplement: Data S5. A .zip archive containing .png files depicting each of the 183 brain hemilineages we have used from the FAFB-FlyWire dataset, related to Figure 7 — Neurons in each hemilineage are colored by their neuron-level transmitter predictions, hemilineage names given in the file name. Hemilineage labels for the FAFB-FlyWire dataset are fully reported in Schlegel et al.S2 [file mmc6.zip › chosen_hemilineages/DM6_posterior__fafb_hemibrain.png]

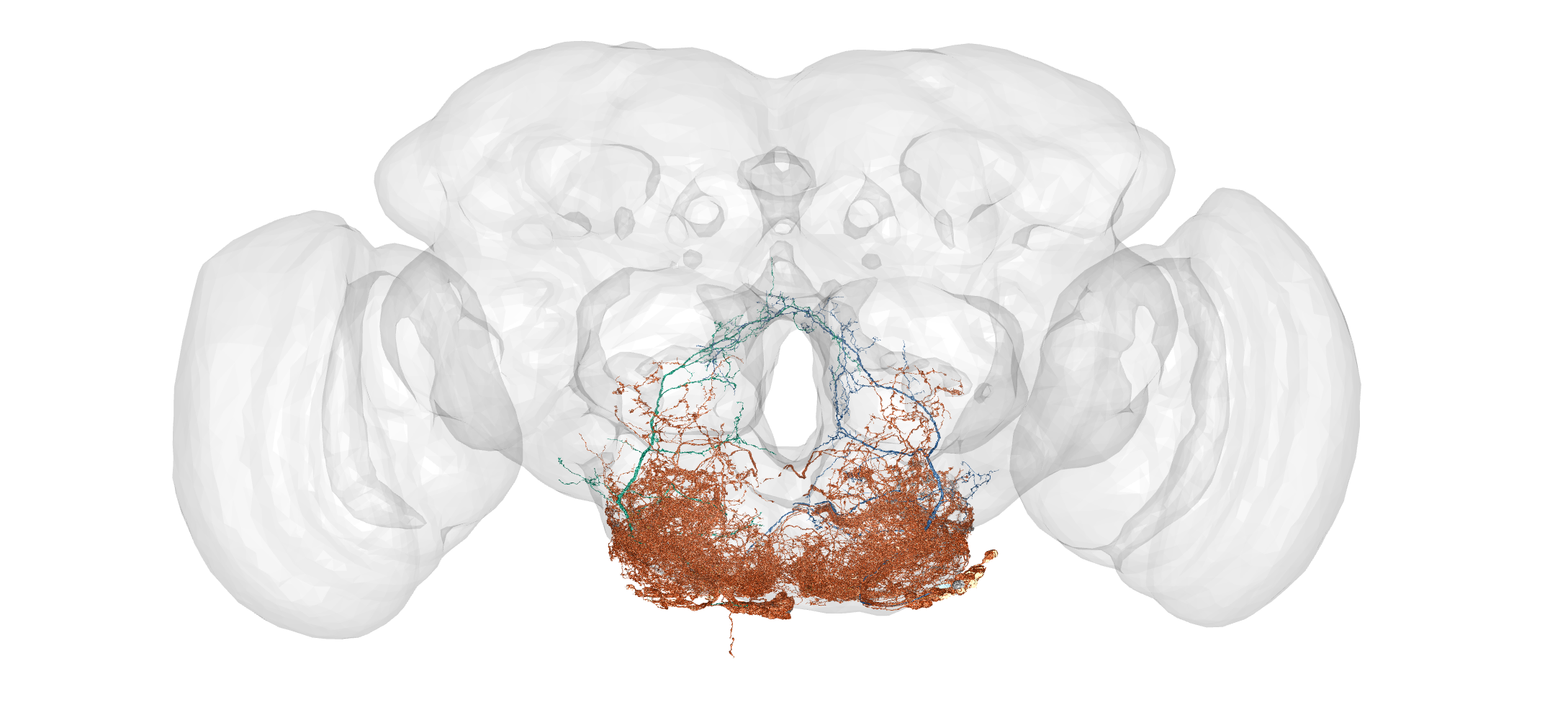

Supplement: Data S5. A .zip archive containing .png files depicting each of the 183 brain hemilineages we have used from the FAFB-FlyWire dataset, related to Figure 7 — Neurons in each hemilineage are colored by their neuron-level transmitter predictions, hemilineage names given in the file name. Hemilineage labels for the FAFB-FlyWire dataset are fully reported in Schlegel et al.S2 [file mmc6.zip › chosen_hemilineages/MX12__fafb.png]

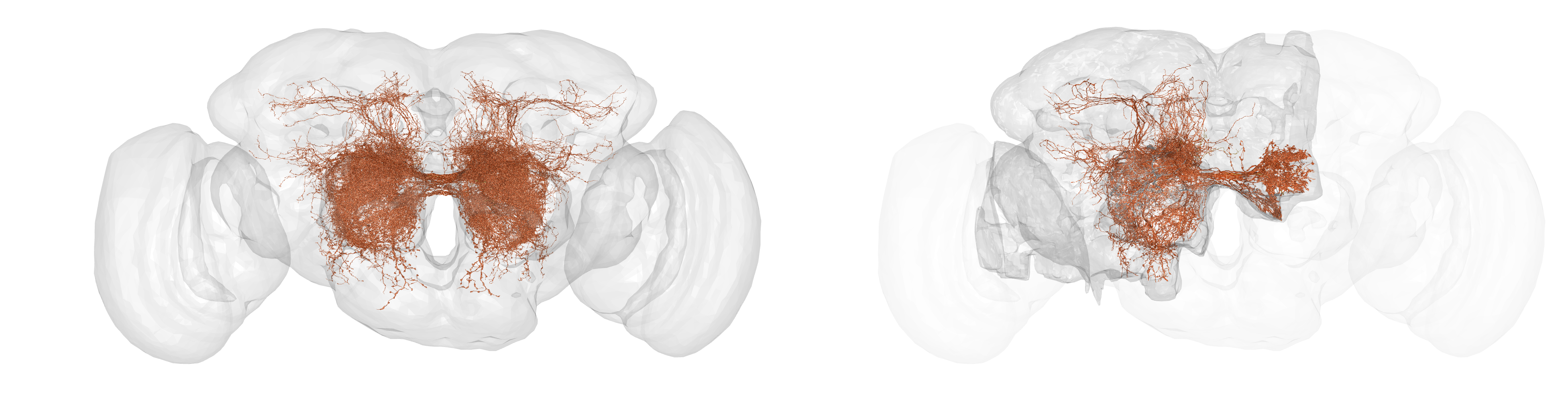

Supplement: Data S5. A .zip archive containing .png files depicting each of the 183 brain hemilineages we have used from the FAFB-FlyWire dataset, related to Figure 7 — Neurons in each hemilineage are colored by their neuron-level transmitter predictions, hemilineage names given in the file name. Hemilineage labels for the FAFB-FlyWire dataset are fully reported in Schlegel et al.S2 [file mmc6.zip › chosen_hemilineages/DM5_central__fafb_hemibrain.png]

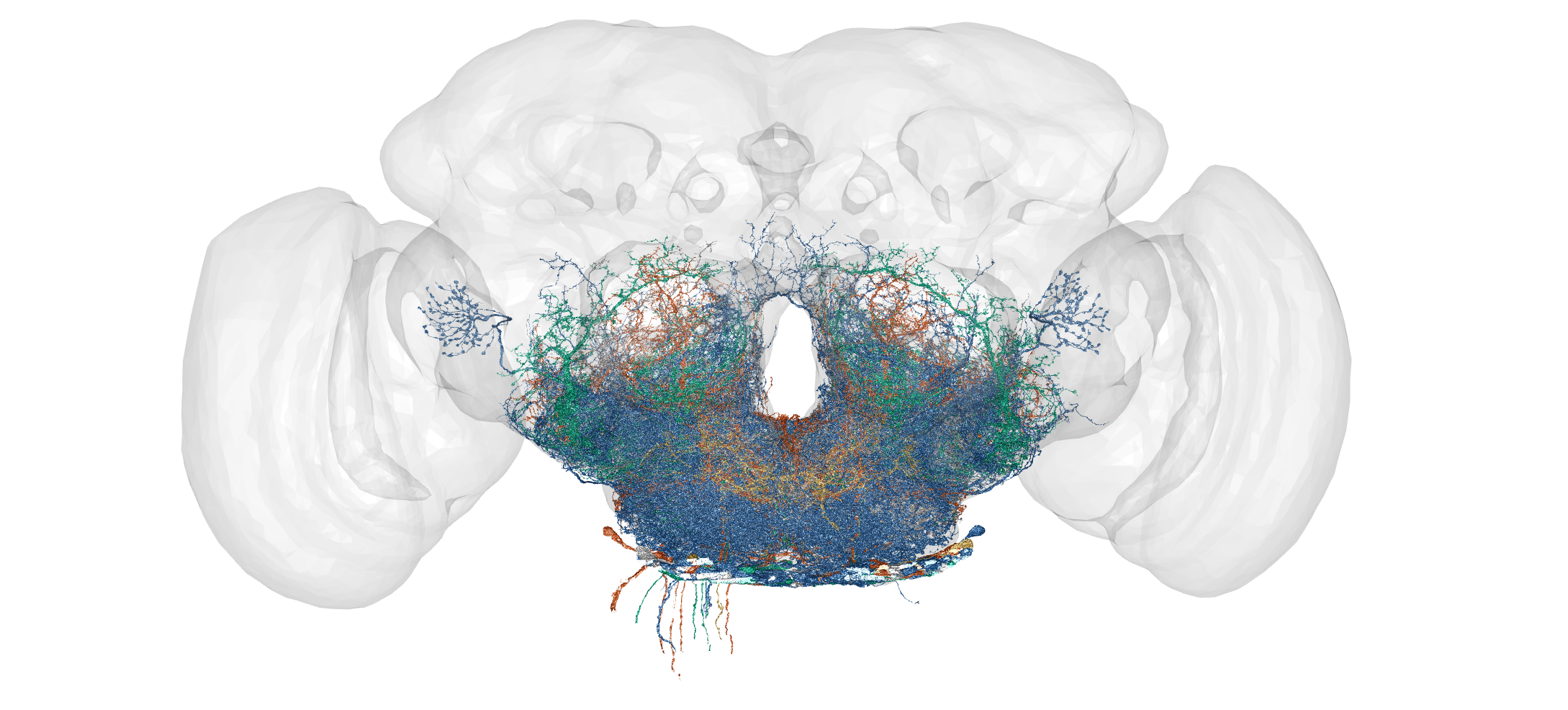

Supplement: Data S5. A .zip archive containing .png files depicting each of the 183 brain hemilineages we have used from the FAFB-FlyWire dataset, related to Figure 7 — Neurons in each hemilineage are colored by their neuron-level transmitter predictions, hemilineage names given in the file name. Hemilineage labels for the FAFB-FlyWire dataset are fully reported in Schlegel et al.S2 [file mmc6.zip › chosen_hemilineages/MX3__fafb.png]

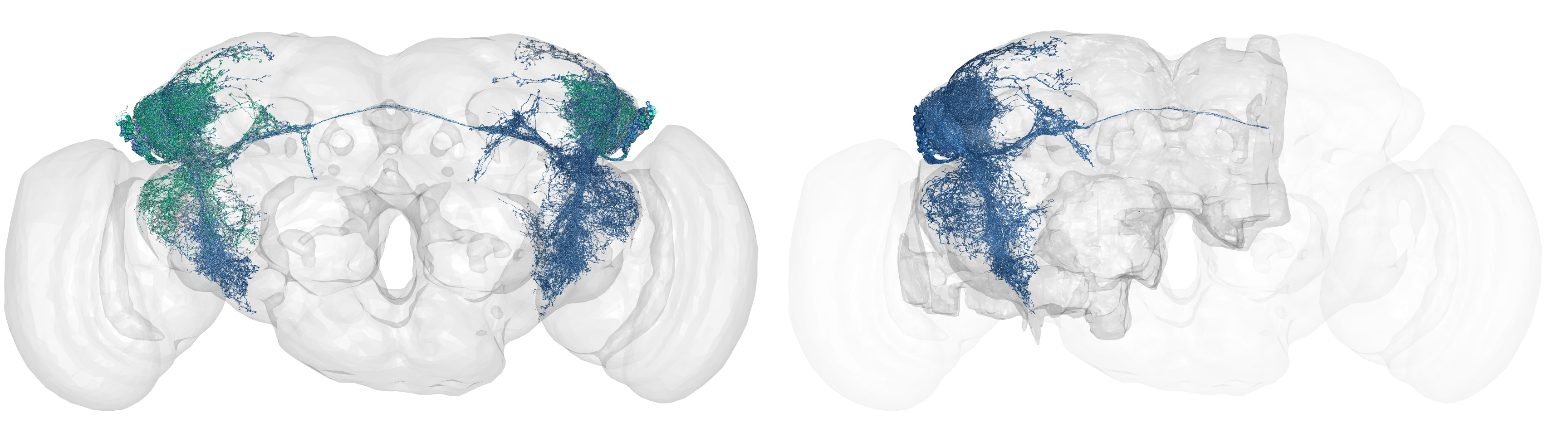

Supplement: Data S5. A .zip archive containing .png files depicting each of the 183 brain hemilineages we have used from the FAFB-FlyWire dataset, related to Figure 7 — Neurons in each hemilineage are colored by their neuron-level transmitter predictions, hemilineage names given in the file name. Hemilineage labels for the FAFB-FlyWire dataset are fully reported in Schlegel et al.S2 [file mmc6.zip › chosen_hemilineages/LHl1__fafb_hemibrain.png]

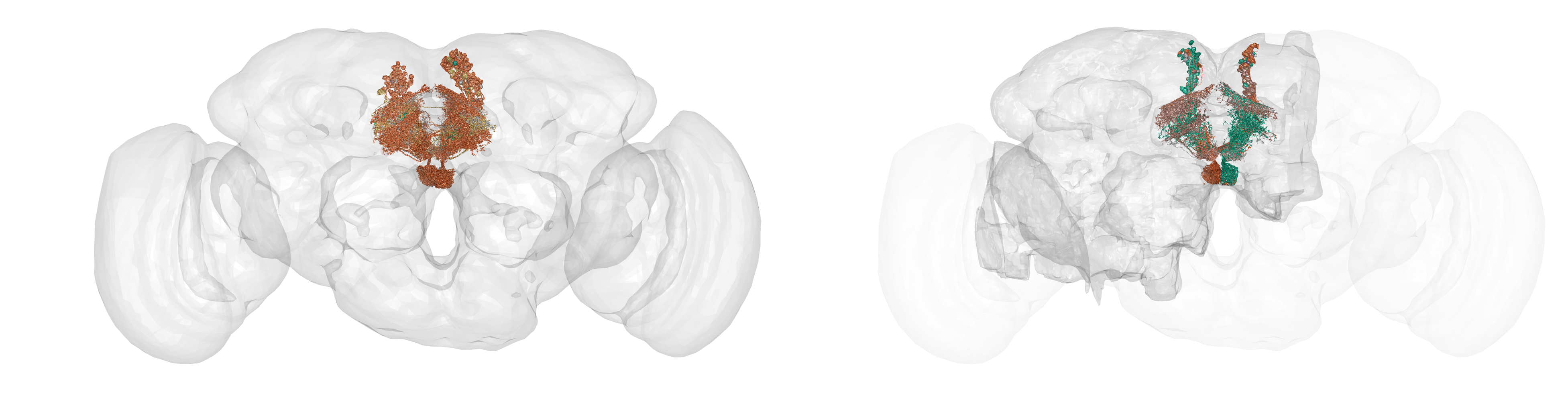

Supplement: Data S5. A .zip archive containing .png files depicting each of the 183 brain hemilineages we have used from the FAFB-FlyWire dataset, related to Figure 7 — Neurons in each hemilineage are colored by their neuron-level transmitter predictions, hemilineage names given in the file name. Hemilineage labels for the FAFB-FlyWire dataset are fully reported in Schlegel et al.S2 [file mmc6.zip › chosen_hemilineages/DM2_CX_p__fafb_hemibrain.png]

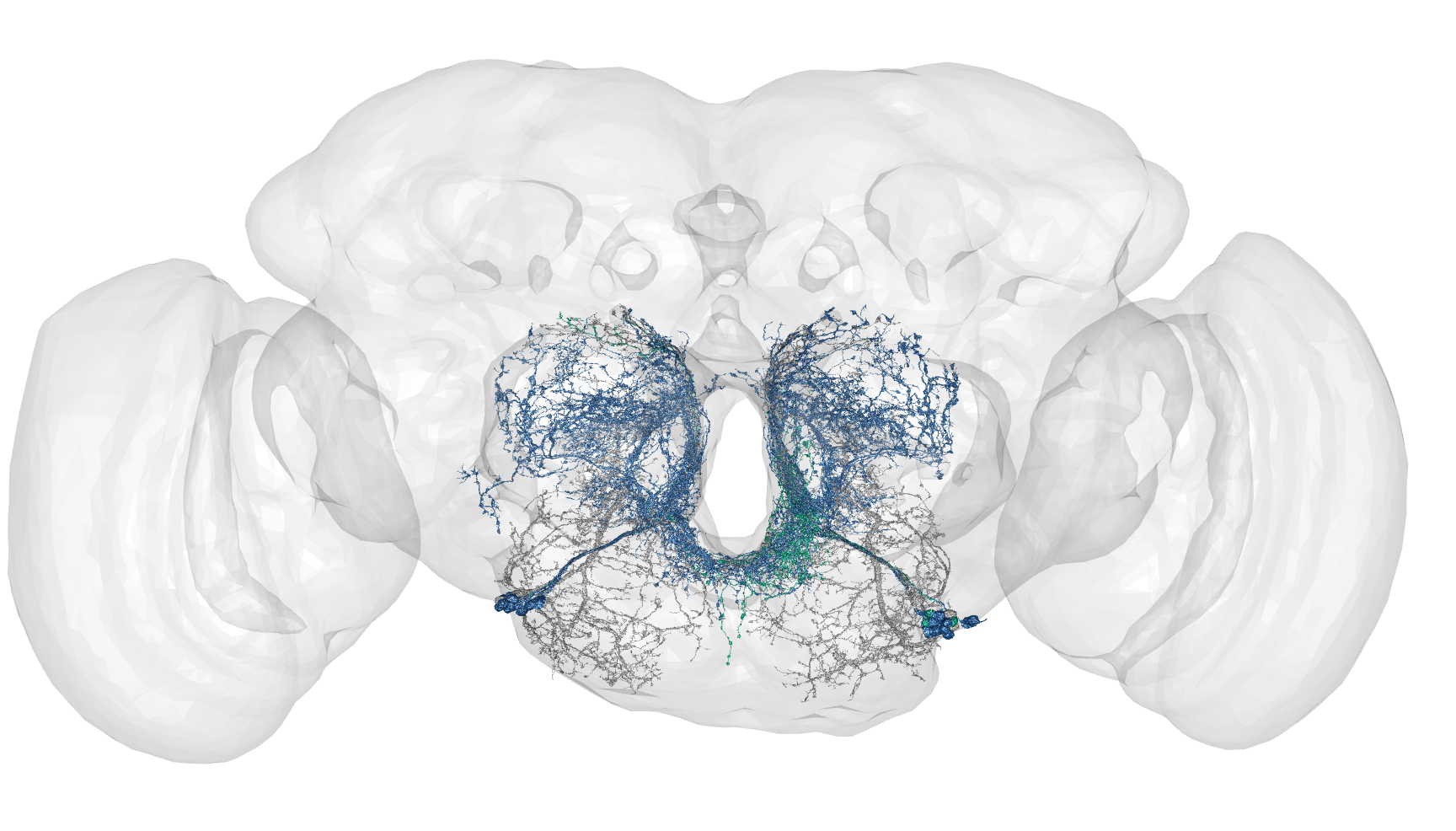

Supplement: Data S5. A .zip archive containing .png files depicting each of the 183 brain hemilineages we have used from the FAFB-FlyWire dataset, related to Figure 7 — Neurons in each hemilineage are colored by their neuron-level transmitter predictions, hemilineage names given in the file name. Hemilineage labels for the FAFB-FlyWire dataset are fully reported in Schlegel et al.S2 [file mmc6.zip › chosen_hemilineages/FLAa1__fafb.png]

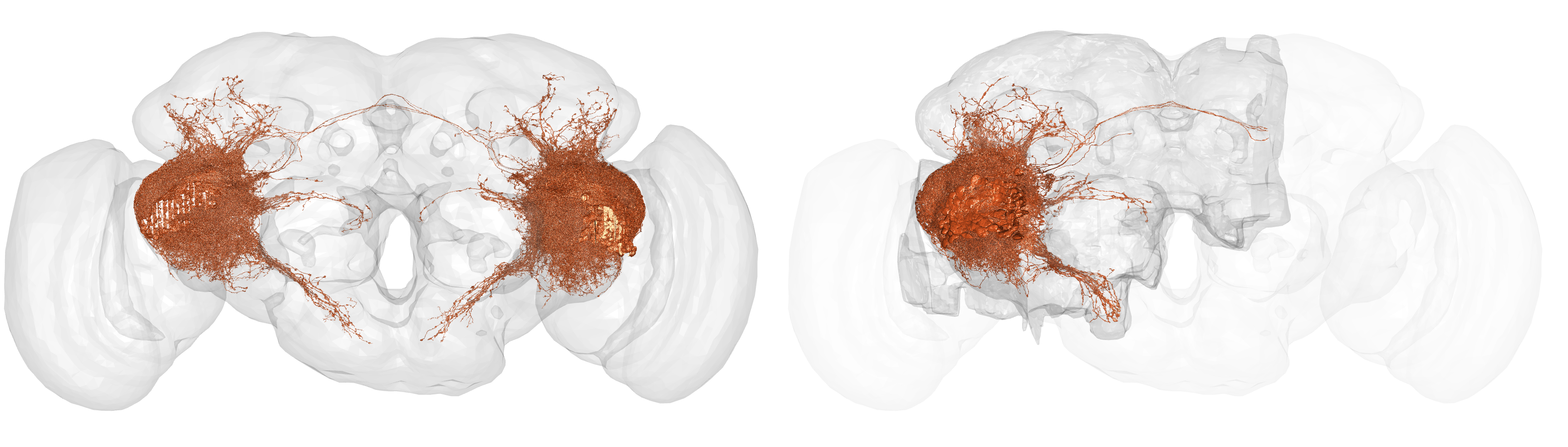

Supplement: Data S5. A .zip archive containing .png files depicting each of the 183 brain hemilineages we have used from the FAFB-FlyWire dataset, related to Figure 7 — Neurons in each hemilineage are colored by their neuron-level transmitter predictions, hemilineage names given in the file name. Hemilineage labels for the FAFB-FlyWire dataset are fully reported in Schlegel et al.S2 [file mmc6.zip › chosen_hemilineages/VLPa1_lateral__fafb_hemibrain.png]

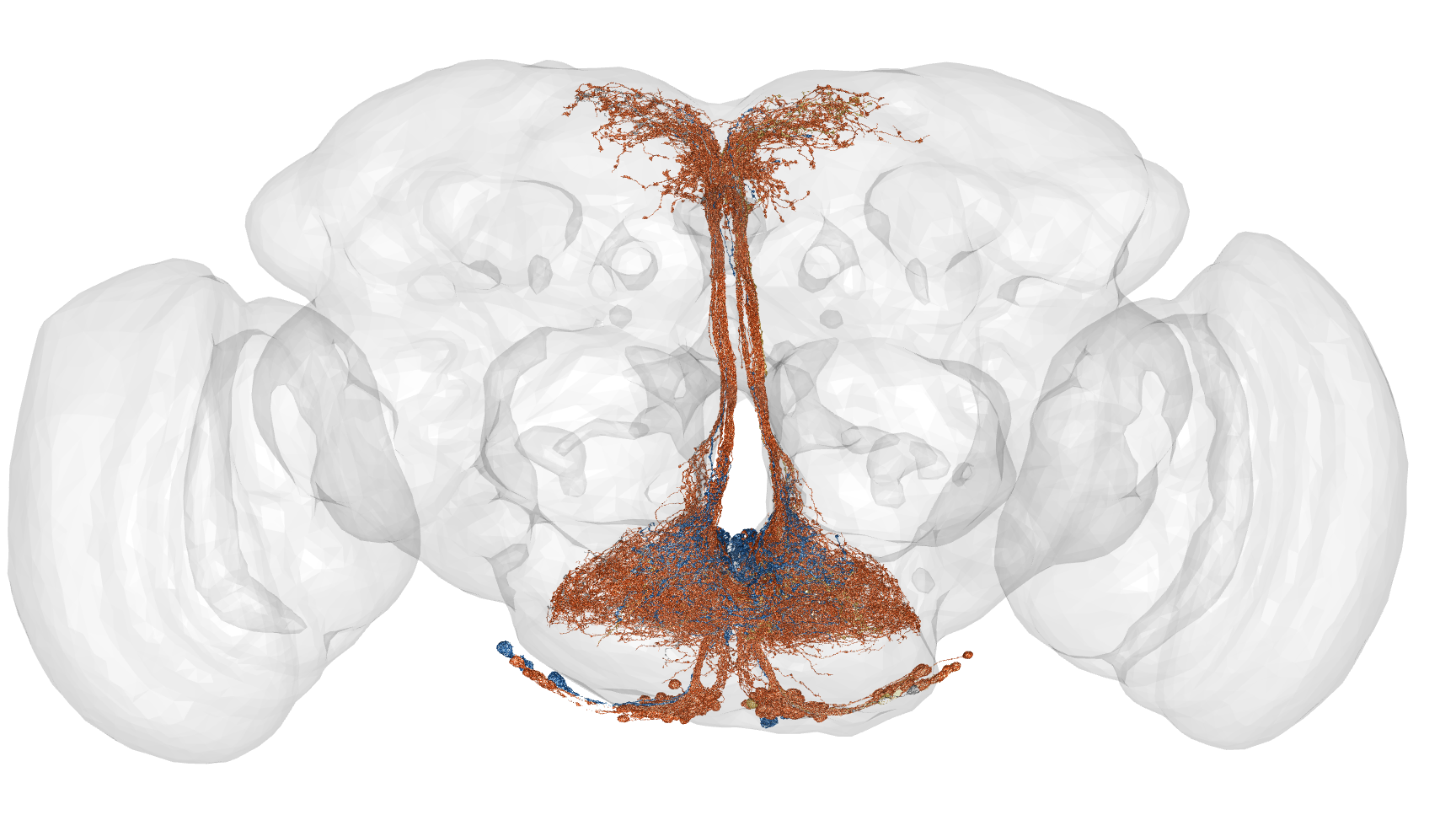

Supplement: Data S5. A .zip archive containing .png files depicting each of the 183 brain hemilineages we have used from the FAFB-FlyWire dataset, related to Figure 7 — Neurons in each hemilineage are colored by their neuron-level transmitter predictions, hemilineage names given in the file name. Hemilineage labels for the FAFB-FlyWire dataset are fully reported in Schlegel et al.S2 [file mmc6.zip › chosen_hemilineages/LB5__fafb.png]

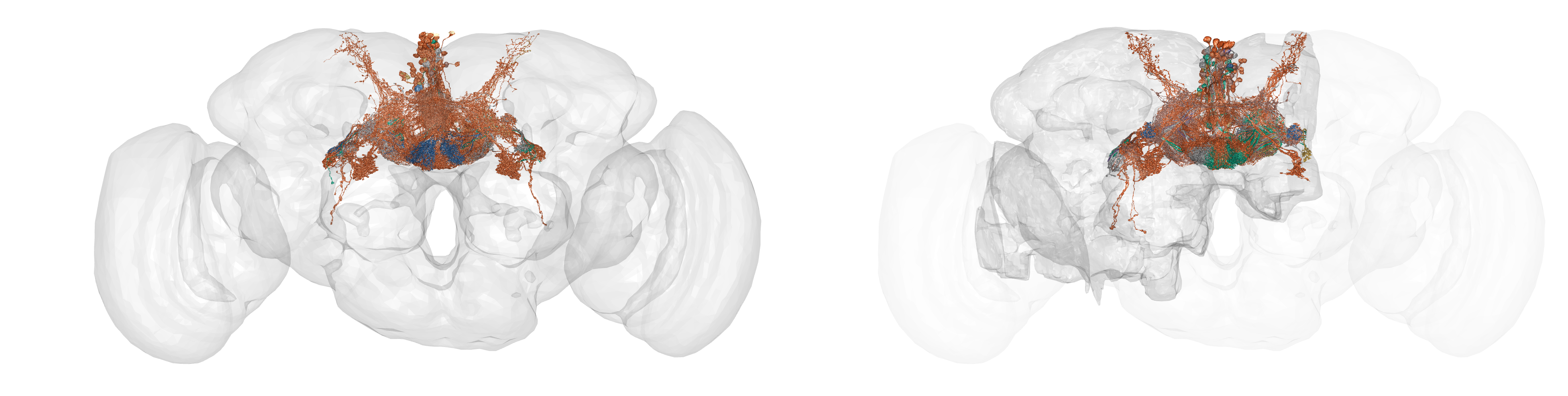

Supplement: Data S5. A .zip archive containing .png files depicting each of the 183 brain hemilineages we have used from the FAFB-FlyWire dataset, related to Figure 7 — Neurons in each hemilineage are colored by their neuron-level transmitter predictions, hemilineage names given in the file name. Hemilineage labels for the FAFB-FlyWire dataset are fully reported in Schlegel et al.S2 [file mmc6.zip › chosen_hemilineages/DM1_CX_d2__fafb_hemibrain.png]

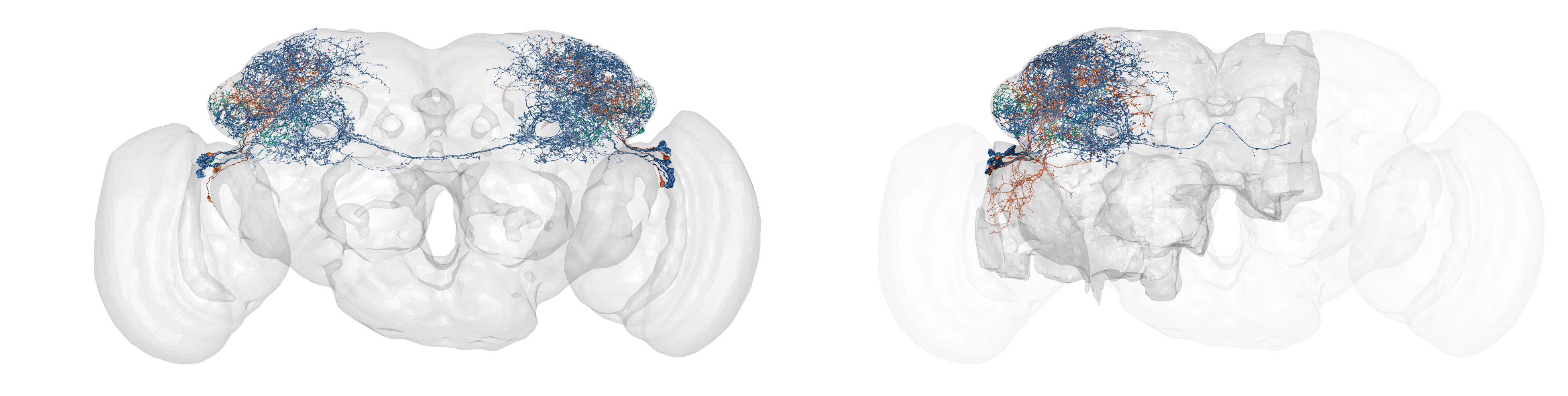

Supplement: Data S5. A .zip archive containing .png files depicting each of the 183 brain hemilineages we have used from the FAFB-FlyWire dataset, related to Figure 7 — Neurons in each hemilineage are colored by their neuron-level transmitter predictions, hemilineage names given in the file name. Hemilineage labels for the FAFB-FlyWire dataset are fully reported in Schlegel et al.S2 [file mmc6.zip › chosen_hemilineages/LHl3__fafb_hemibrain.png]

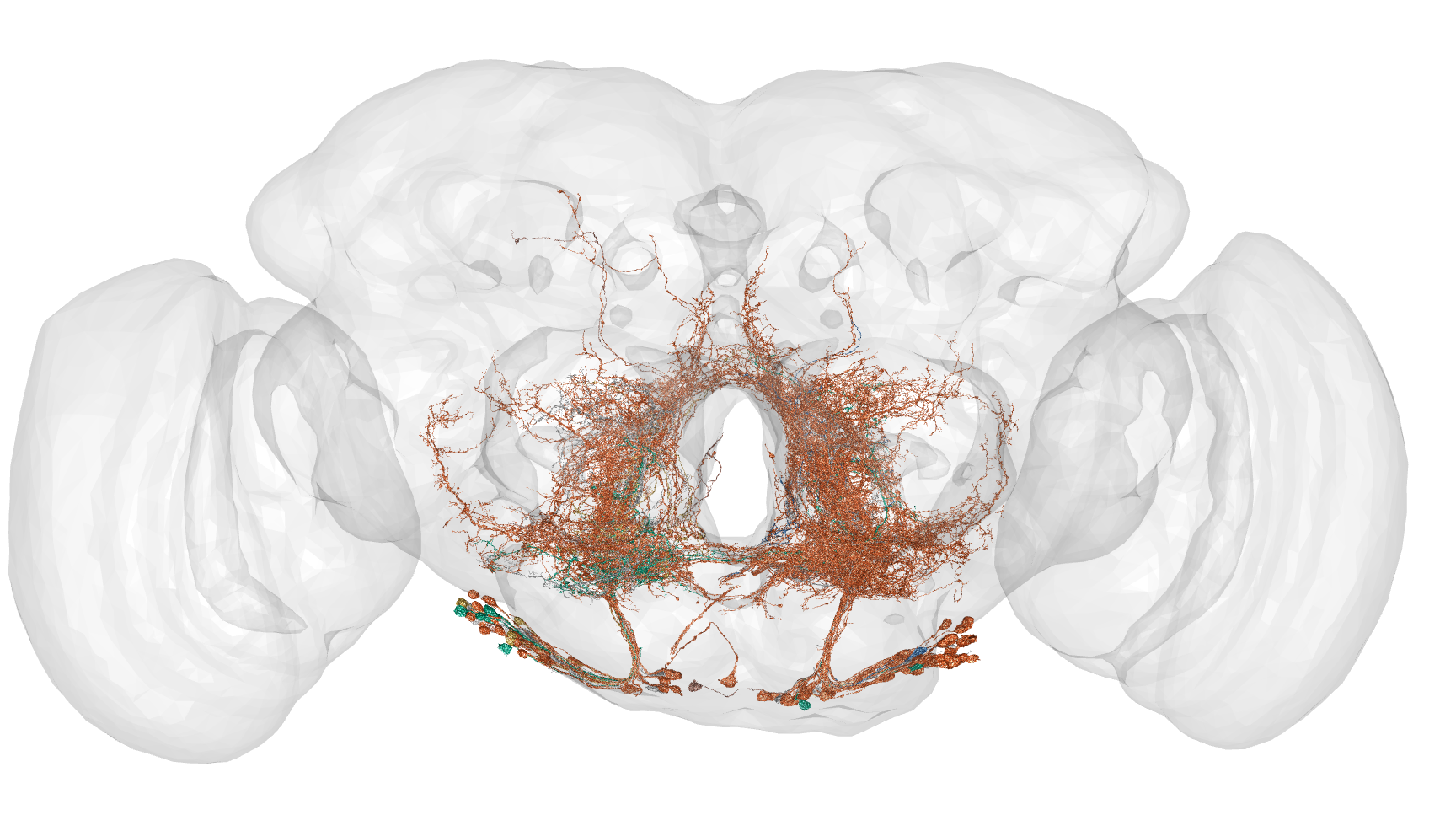

Supplement: Data S5. A .zip archive containing .png files depicting each of the 183 brain hemilineages we have used from the FAFB-FlyWire dataset, related to Figure 7 — Neurons in each hemilineage are colored by their neuron-level transmitter predictions, hemilineage names given in the file name. Hemilineage labels for the FAFB-FlyWire dataset are fully reported in Schlegel et al.S2 [file mmc6.zip › chosen_hemilineages/LB12__fafb.png]

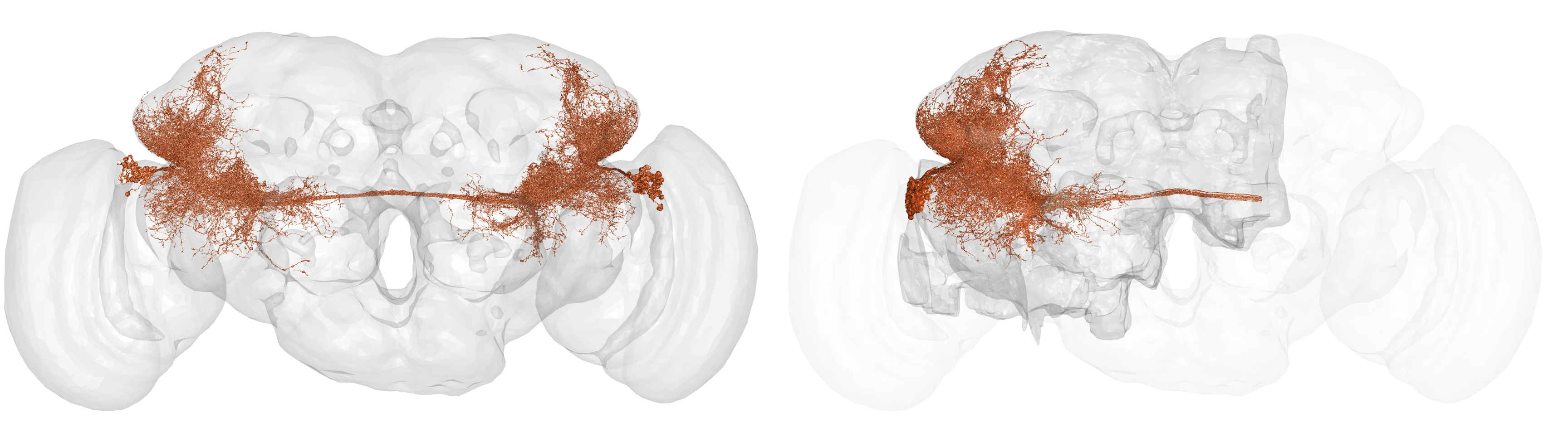

Supplement: Data S5. A .zip archive containing .png files depicting each of the 183 brain hemilineages we have used from the FAFB-FlyWire dataset, related to Figure 7 — Neurons in each hemilineage are colored by their neuron-level transmitter predictions, hemilineage names given in the file name. Hemilineage labels for the FAFB-FlyWire dataset are fully reported in Schlegel et al.S2 [file mmc6.zip › chosen_hemilineages/VLPl2_posterior__fafb_hemibrain.png]

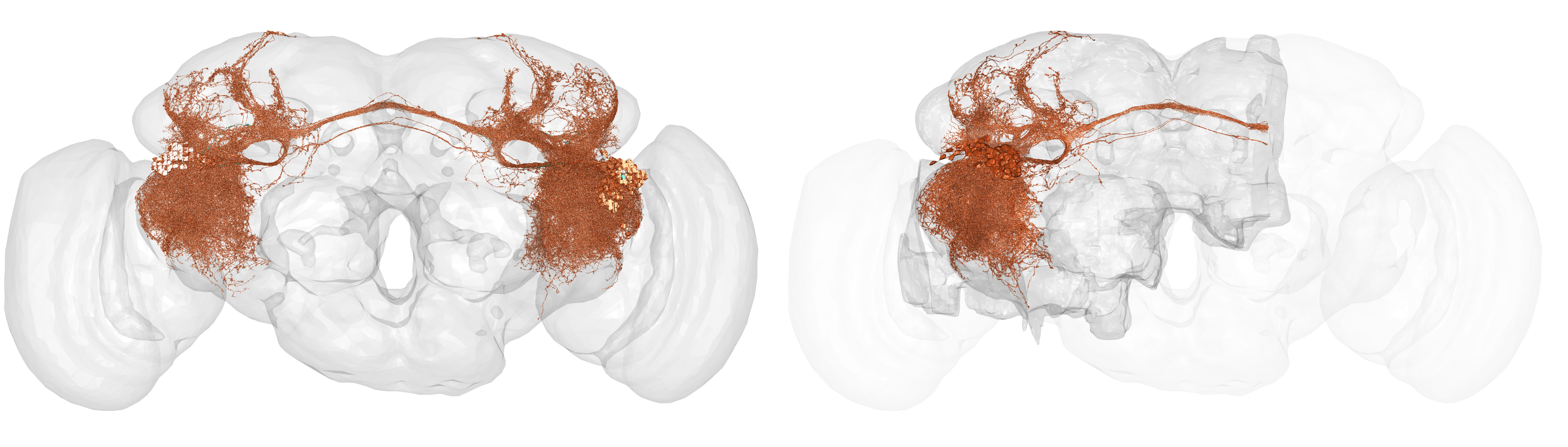

Supplement: Data S5. A .zip archive containing .png files depicting each of the 183 brain hemilineages we have used from the FAFB-FlyWire dataset, related to Figure 7 — Neurons in each hemilineage are colored by their neuron-level transmitter predictions, hemilineage names given in the file name. Hemilineage labels for the FAFB-FlyWire dataset are fully reported in Schlegel et al.S2 [file mmc6.zip › chosen_hemilineages/VLPl&d1_dorsal__fafb_hemibrain.png]

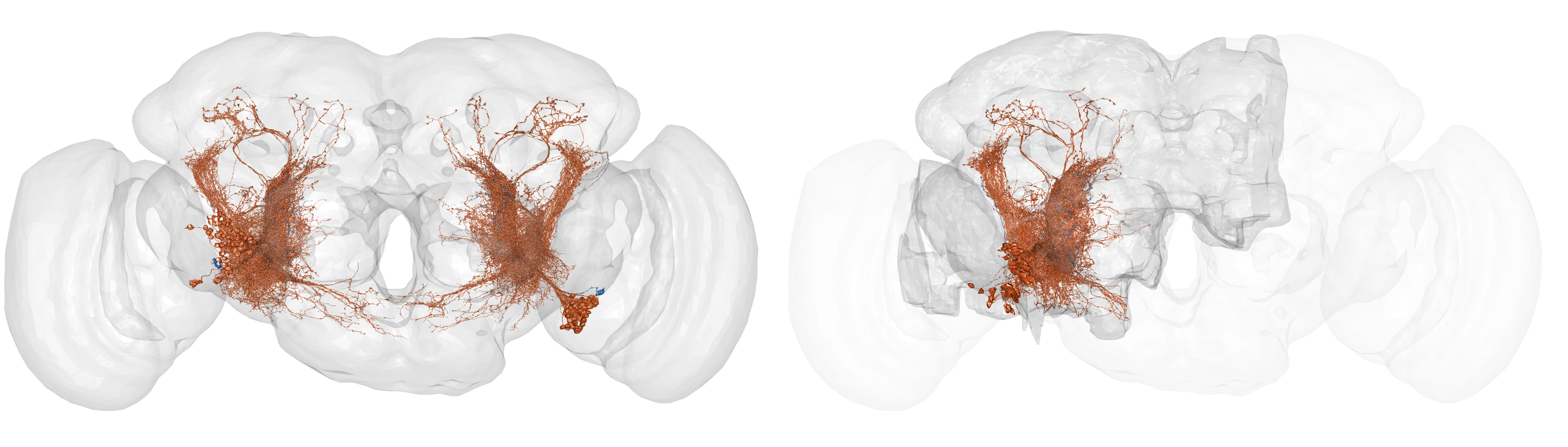

Supplement: Data S5. A .zip archive containing .png files depicting each of the 183 brain hemilineages we have used from the FAFB-FlyWire dataset, related to Figure 7 — Neurons in each hemilineage are colored by their neuron-level transmitter predictions, hemilineage names given in the file name. Hemilineage labels for the FAFB-FlyWire dataset are fully reported in Schlegel et al.S2 [file mmc6.zip › chosen_hemilineages/LALa1_posterior__fafb_hemibrain.png]

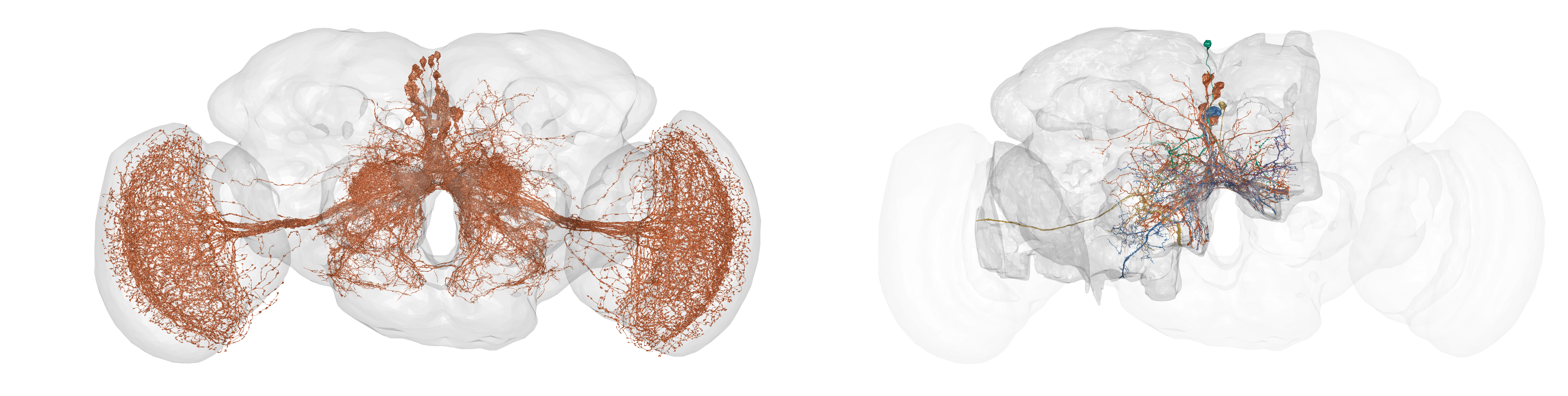

Supplement: Data S5. A .zip archive containing .png files depicting each of the 183 brain hemilineages we have used from the FAFB-FlyWire dataset, related to Figure 7 — Neurons in each hemilineage are colored by their neuron-level transmitter predictions, hemilineage names given in the file name. Hemilineage labels for the FAFB-FlyWire dataset are fully reported in Schlegel et al.S2 [file mmc6.zip › chosen_hemilineages/DM1_posterior__fafb_hemibrain.png]

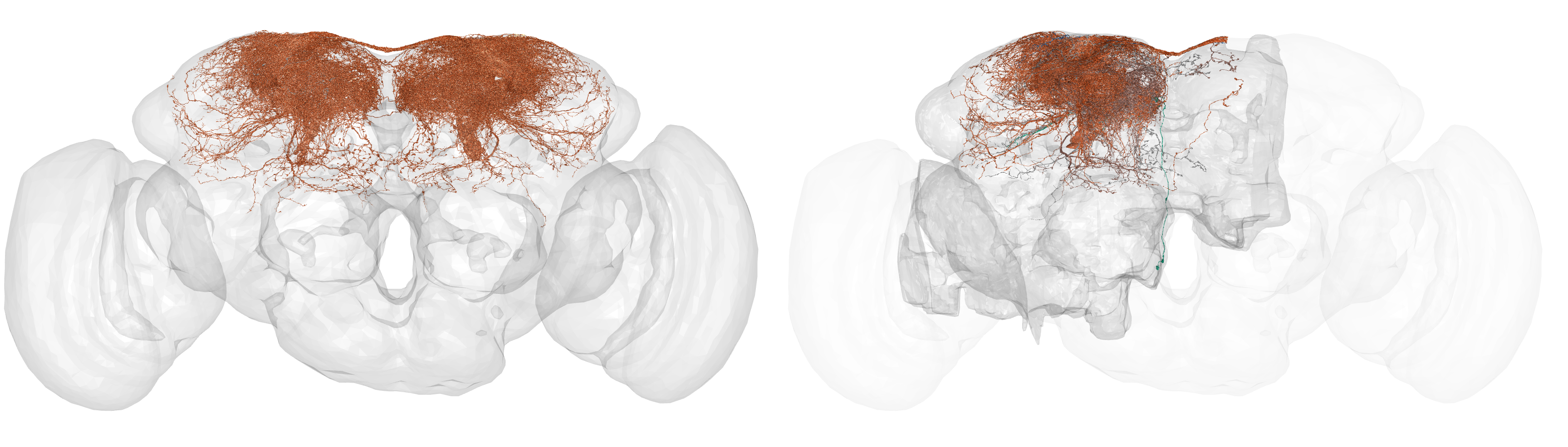

Supplement: Data S5. A .zip archive containing .png files depicting each of the 183 brain hemilineages we have used from the FAFB-FlyWire dataset, related to Figure 7 — Neurons in each hemilineage are colored by their neuron-level transmitter predictions, hemilineage names given in the file name. Hemilineage labels for the FAFB-FlyWire dataset are fully reported in Schlegel et al.S2 [file mmc6.zip › chosen_hemilineages/DM6_dorso_lateral__fafb_hemibrain.png]

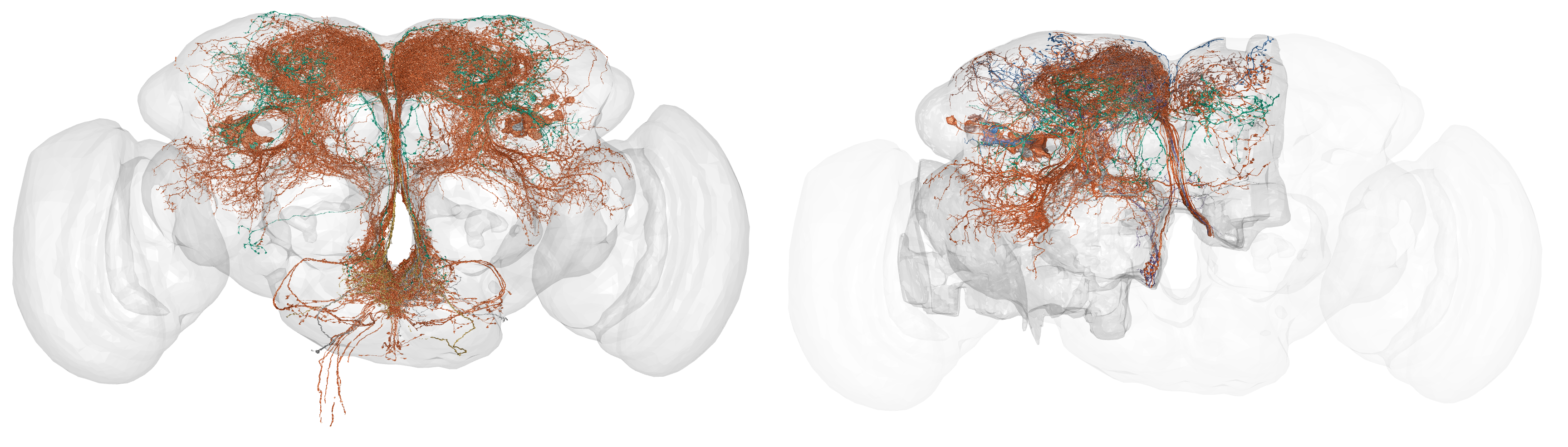

Supplement: Data S5. A .zip archive containing .png files depicting each of the 183 brain hemilineages we have used from the FAFB-FlyWire dataset, related to Figure 7 — Neurons in each hemilineage are colored by their neuron-level transmitter predictions, hemilineage names given in the file name. Hemilineage labels for the FAFB-FlyWire dataset are fully reported in Schlegel et al.S2 [file mmc6.zip › chosen_hemilineages/SMPpv2_dorsal__fafb_hemibrain.png]

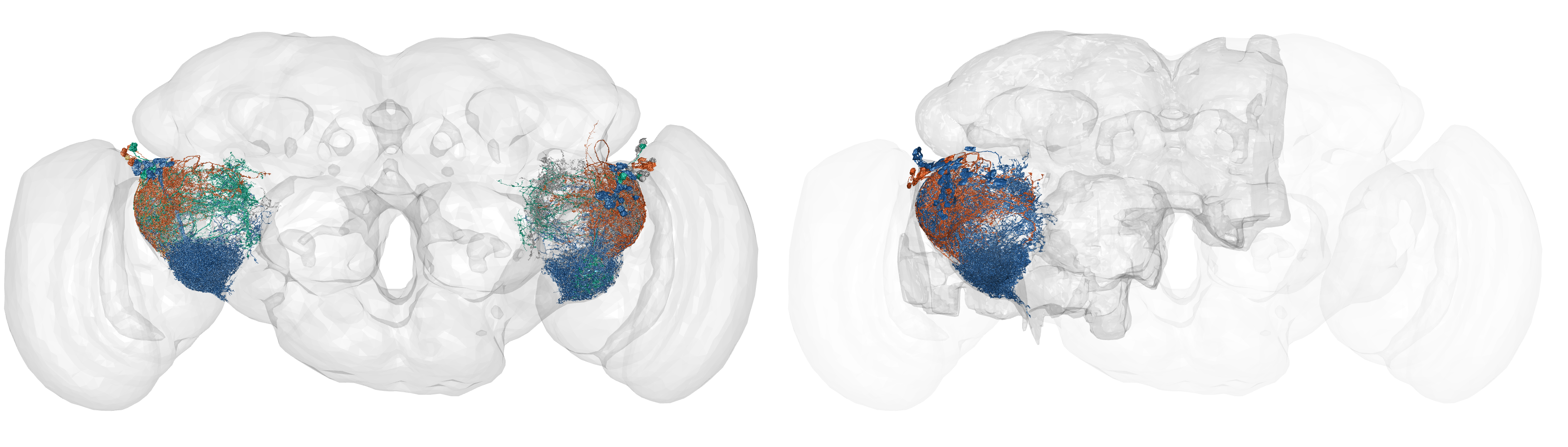

Supplement: Data S5. A .zip archive containing .png files depicting each of the 183 brain hemilineages we have used from the FAFB-FlyWire dataset, related to Figure 7 — Neurons in each hemilineage are colored by their neuron-level transmitter predictions, hemilineage names given in the file name. Hemilineage labels for the FAFB-FlyWire dataset are fully reported in Schlegel et al.S2 [file mmc6.zip › chosen_hemilineages/VLPl4_anterior__fafb_hemibrain.png]

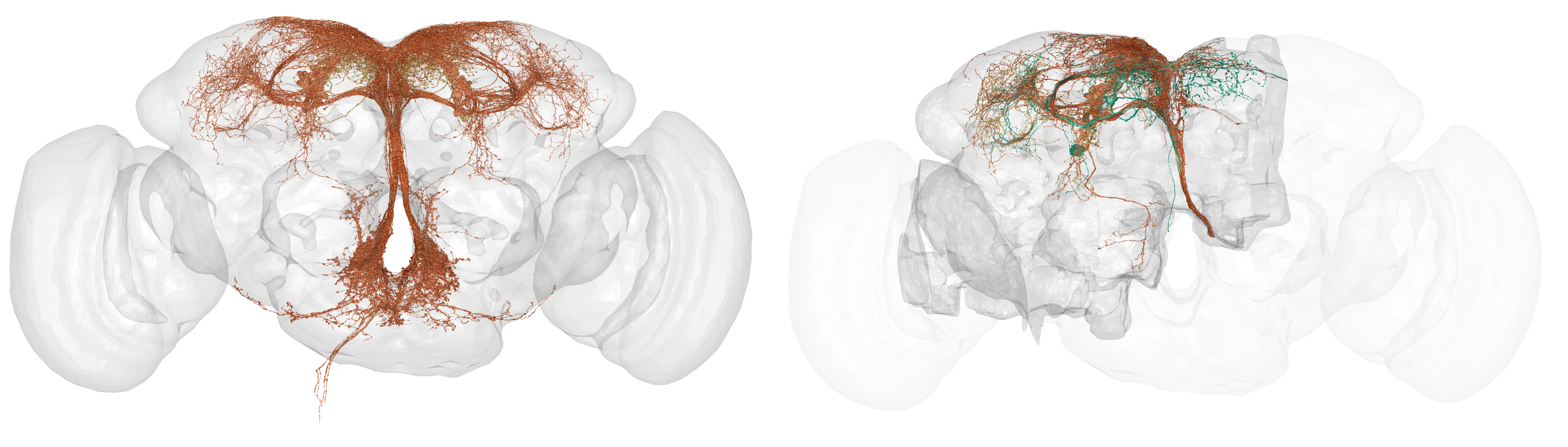

Supplement: Data S5. A .zip archive containing .png files depicting each of the 183 brain hemilineages we have used from the FAFB-FlyWire dataset, related to Figure 7 — Neurons in each hemilineage are colored by their neuron-level transmitter predictions, hemilineage names given in the file name. Hemilineage labels for the FAFB-FlyWire dataset are fully reported in Schlegel et al.S2 [file mmc6.zip › chosen_hemilineages/SMPpv1__fafb_hemibrain.png]

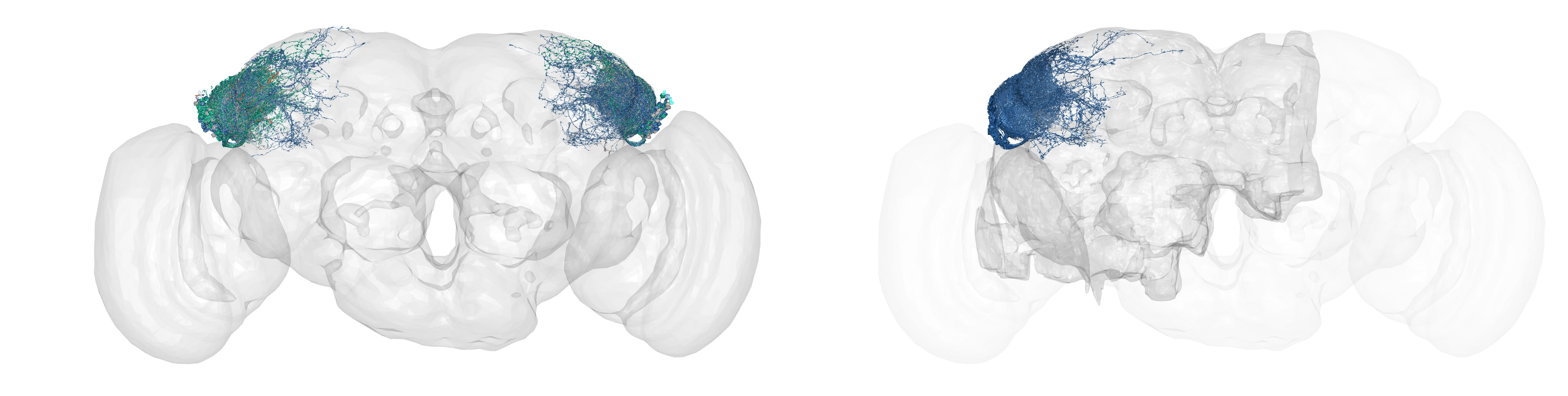

Supplement: Data S5. A .zip archive containing .png files depicting each of the 183 brain hemilineages we have used from the FAFB-FlyWire dataset, related to Figure 7 — Neurons in each hemilineage are colored by their neuron-level transmitter predictions, hemilineage names given in the file name. Hemilineage labels for the FAFB-FlyWire dataset are fully reported in Schlegel et al.S2 [file mmc6.zip › chosen_hemilineages/LHl4_dorsal__fafb_hemibrain.png]

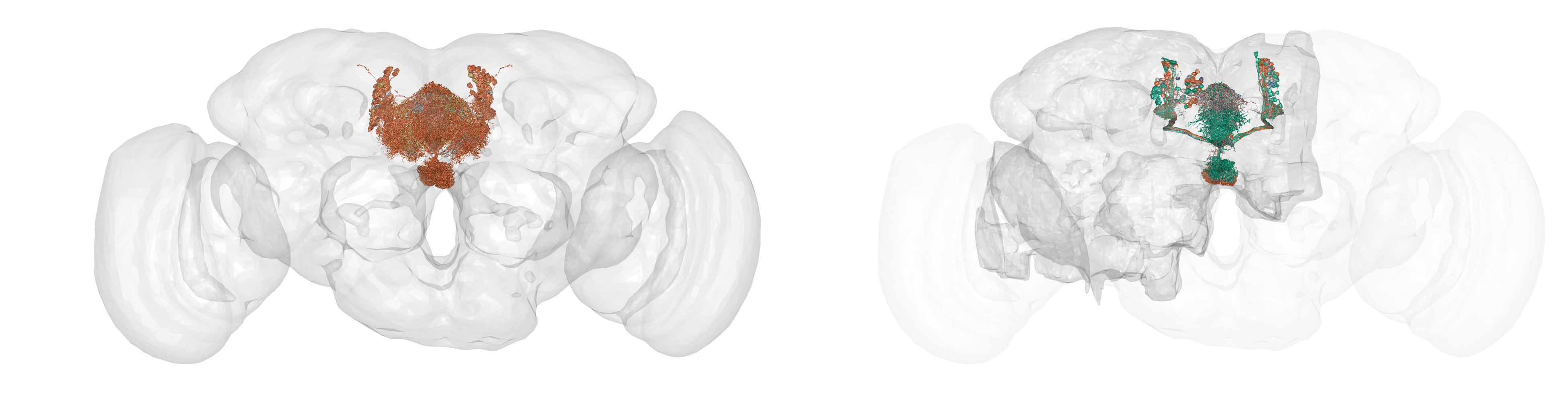

Supplement: Data S5. A .zip archive containing .png files depicting each of the 183 brain hemilineages we have used from the FAFB-FlyWire dataset, related to Figure 7 — Neurons in each hemilineage are colored by their neuron-level transmitter predictions, hemilineage names given in the file name. Hemilineage labels for the FAFB-FlyWire dataset are fully reported in Schlegel et al.S2 [file mmc6.zip › chosen_hemilineages/DM3_CX_p__fafb_hemibrain.png]

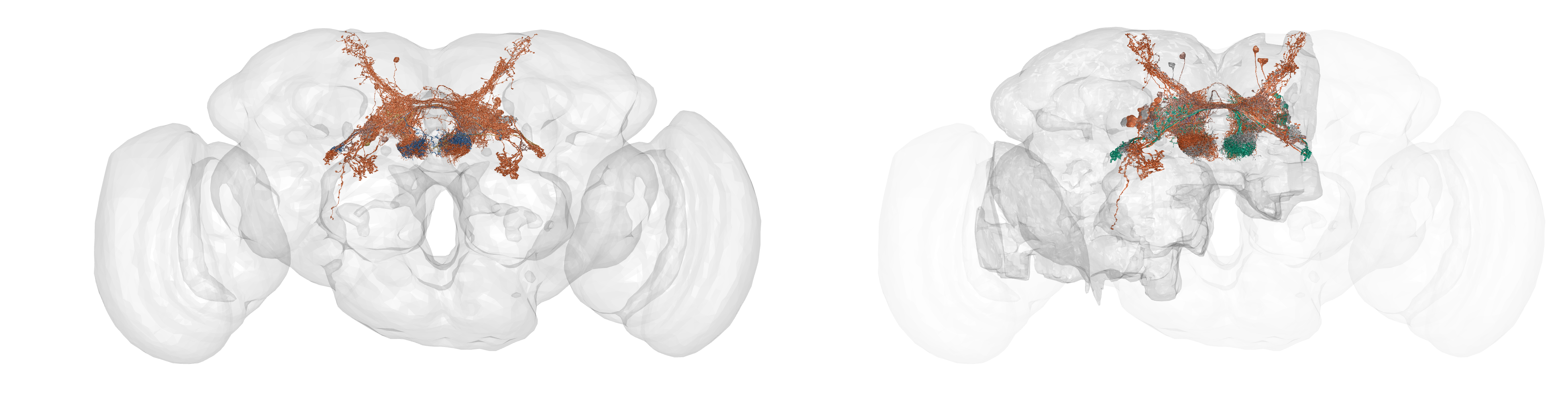

Supplement: Data S5. A .zip archive containing .png files depicting each of the 183 brain hemilineages we have used from the FAFB-FlyWire dataset, related to Figure 7 — Neurons in each hemilineage are colored by their neuron-level transmitter predictions, hemilineage names given in the file name. Hemilineage labels for the FAFB-FlyWire dataset are fully reported in Schlegel et al.S2 [file mmc6.zip › chosen_hemilineages/DM4_CX_d1__fafb_hemibrain.png]

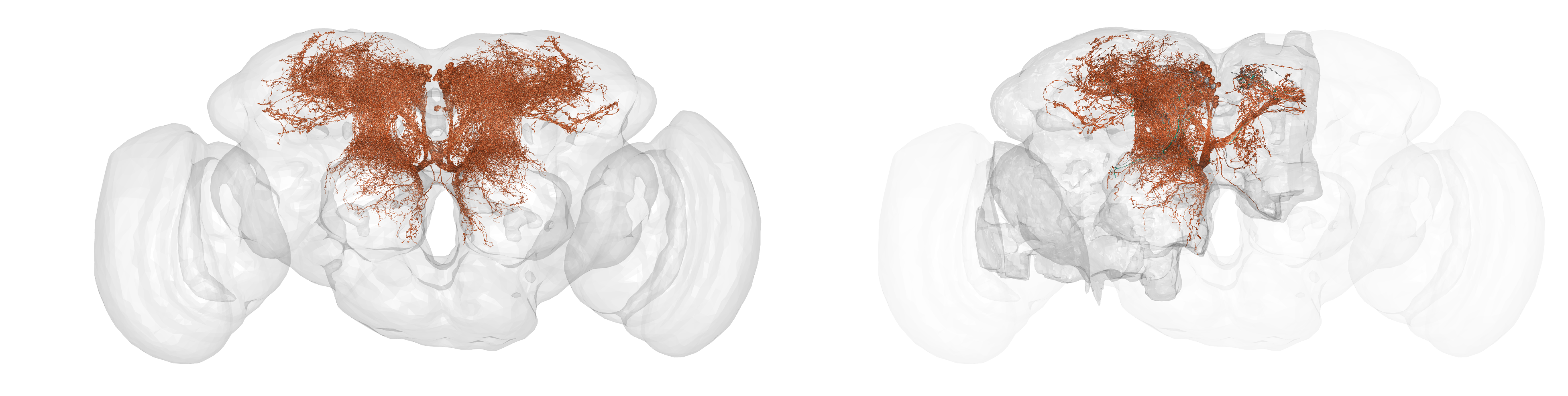

Supplement: Data S5. A .zip archive containing .png files depicting each of the 183 brain hemilineages we have used from the FAFB-FlyWire dataset, related to Figure 7 — Neurons in each hemilineage are colored by their neuron-level transmitter predictions, hemilineage names given in the file name. Hemilineage labels for the FAFB-FlyWire dataset are fully reported in Schlegel et al.S2 [file mmc6.zip › chosen_hemilineages/DM1_antero_dorsal__fafb_hemibrain.png]

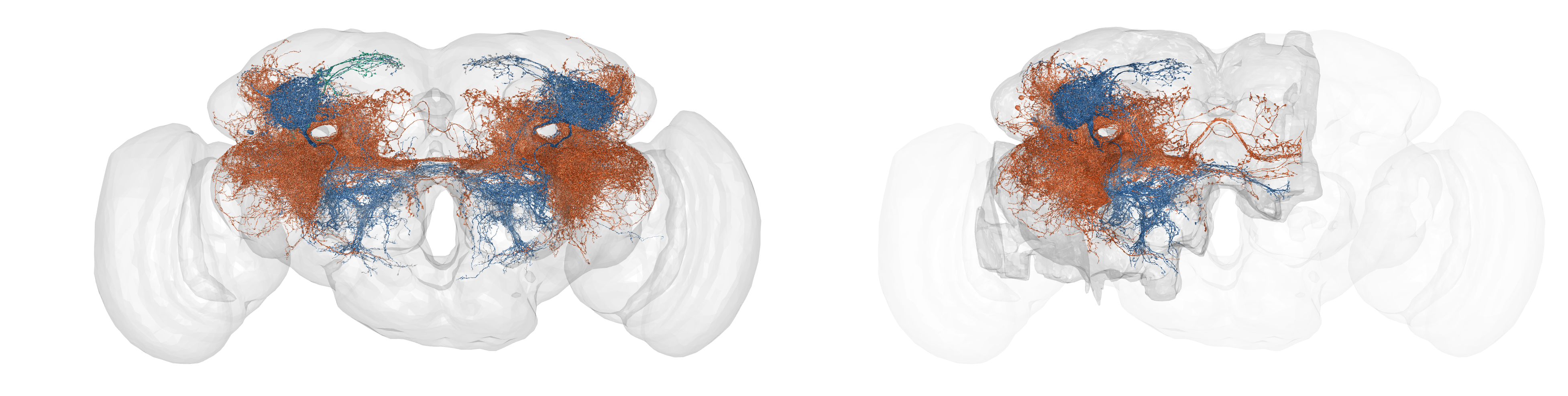

Supplement: Data S5. A .zip archive containing .png files depicting each of the 183 brain hemilineages we have used from the FAFB-FlyWire dataset, related to Figure 7 — Neurons in each hemilineage are colored by their neuron-level transmitter predictions, hemilineage names given in the file name. Hemilineage labels for the FAFB-FlyWire dataset are fully reported in Schlegel et al.S2 [file mmc6.zip › chosen_hemilineages/DL1_ventral__fafb_hemibrain.png]

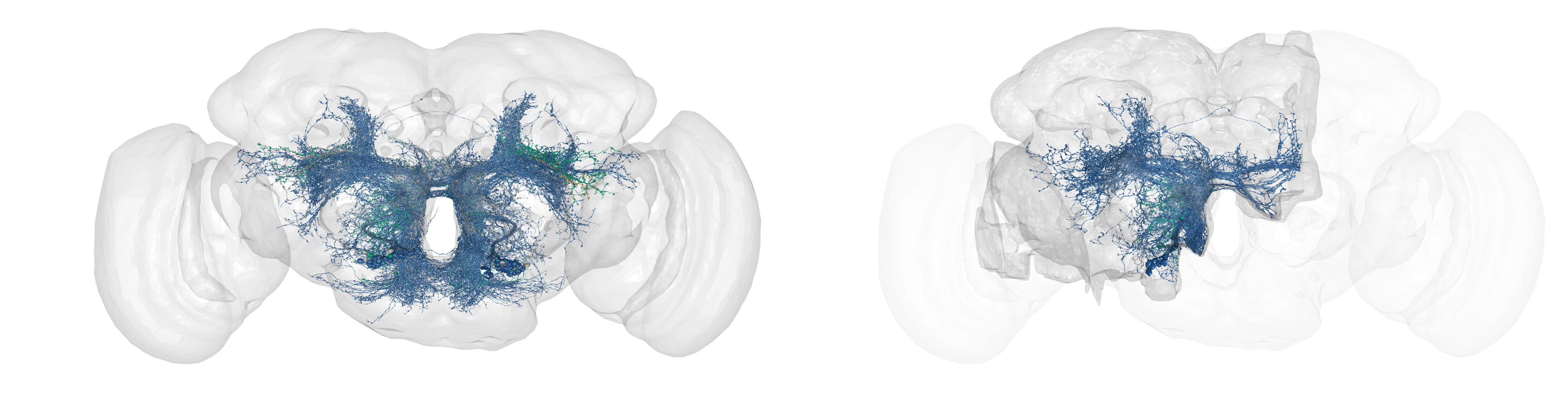

Supplement: Data S5. A .zip archive containing .png files depicting each of the 183 brain hemilineages we have used from the FAFB-FlyWire dataset, related to Figure 7 — Neurons in each hemilineage are colored by their neuron-level transmitter predictions, hemilineage names given in the file name. Hemilineage labels for the FAFB-FlyWire dataset are fully reported in Schlegel et al.S2 [file mmc6.zip › chosen_hemilineages/PSa1__fafb_hemibrain.png]

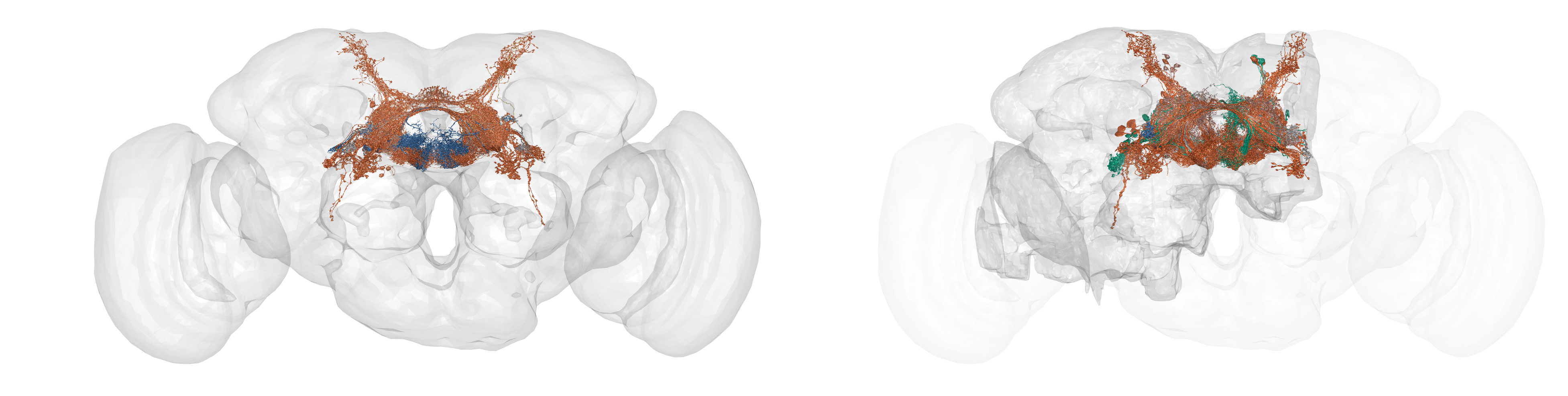

Supplement: Data S5. A .zip archive containing .png files depicting each of the 183 brain hemilineages we have used from the FAFB-FlyWire dataset, related to Figure 7 — Neurons in each hemilineage are colored by their neuron-level transmitter predictions, hemilineage names given in the file name. Hemilineage labels for the FAFB-FlyWire dataset are fully reported in Schlegel et al.S2 [file mmc6.zip › chosen_hemilineages/DM4_CX_d2__fafb_hemibrain.png]

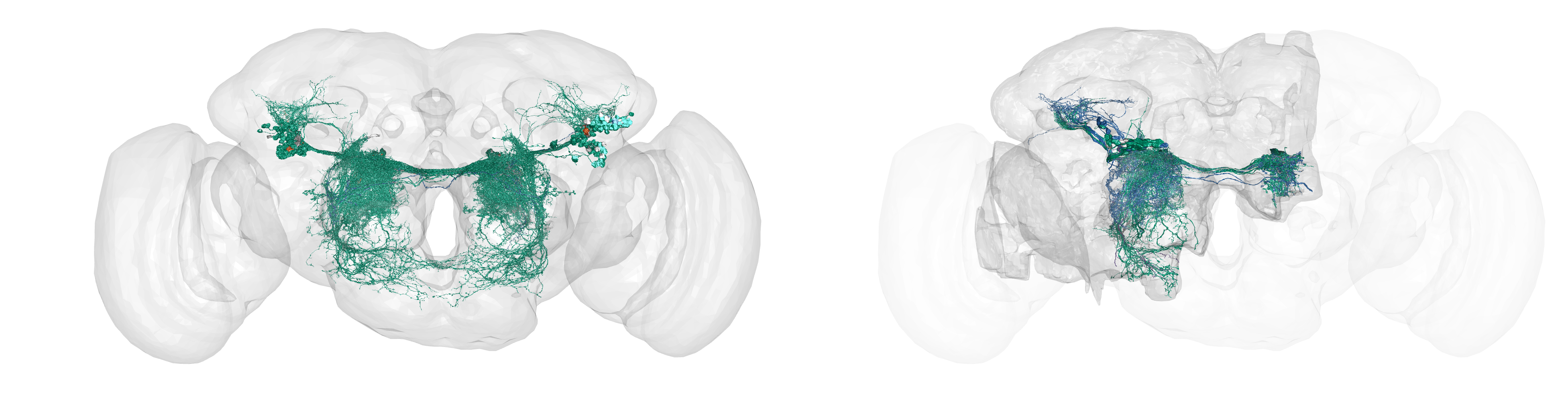

Supplement: Data S5. A .zip archive containing .png files depicting each of the 183 brain hemilineages we have used from the FAFB-FlyWire dataset, related to Figure 7 — Neurons in each hemilineage are colored by their neuron-level transmitter predictions, hemilineage names given in the file name. Hemilineage labels for the FAFB-FlyWire dataset are fully reported in Schlegel et al.S2 [file mmc6.zip › chosen_hemilineages/AOTUv3_ventral__fafb_hemibrain.png]

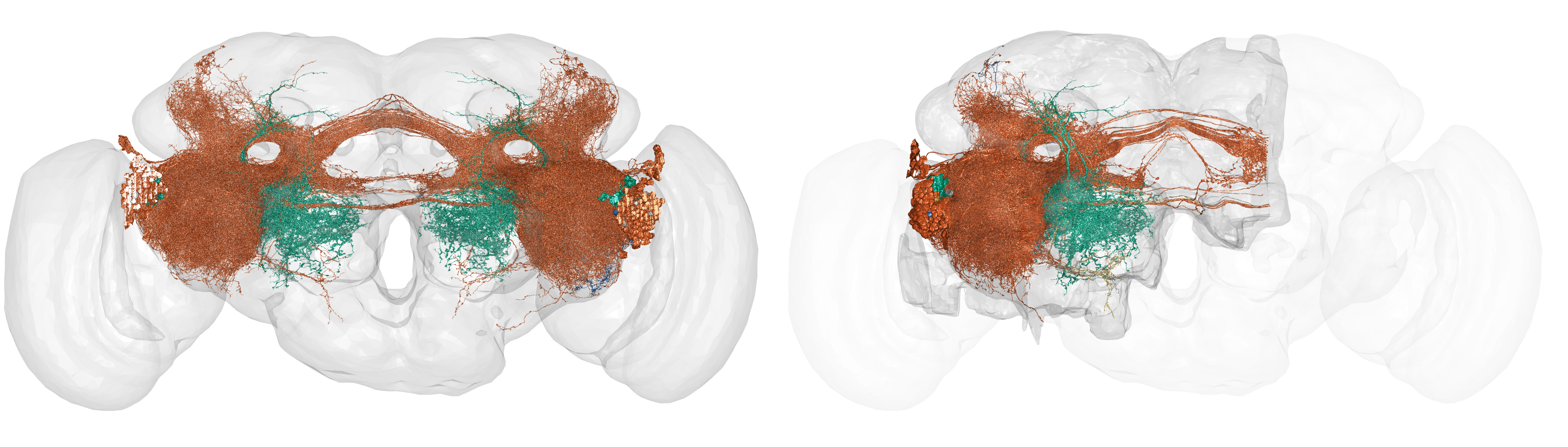

Supplement: Data S5. A .zip archive containing .png files depicting each of the 183 brain hemilineages we have used from the FAFB-FlyWire dataset, related to Figure 7 — Neurons in each hemilineage are colored by their neuron-level transmitter predictions, hemilineage names given in the file name. Hemilineage labels for the FAFB-FlyWire dataset are fully reported in Schlegel et al.S2 [file mmc6.zip › chosen_hemilineages/VLPl2_medial__fafb_hemibrain.png]

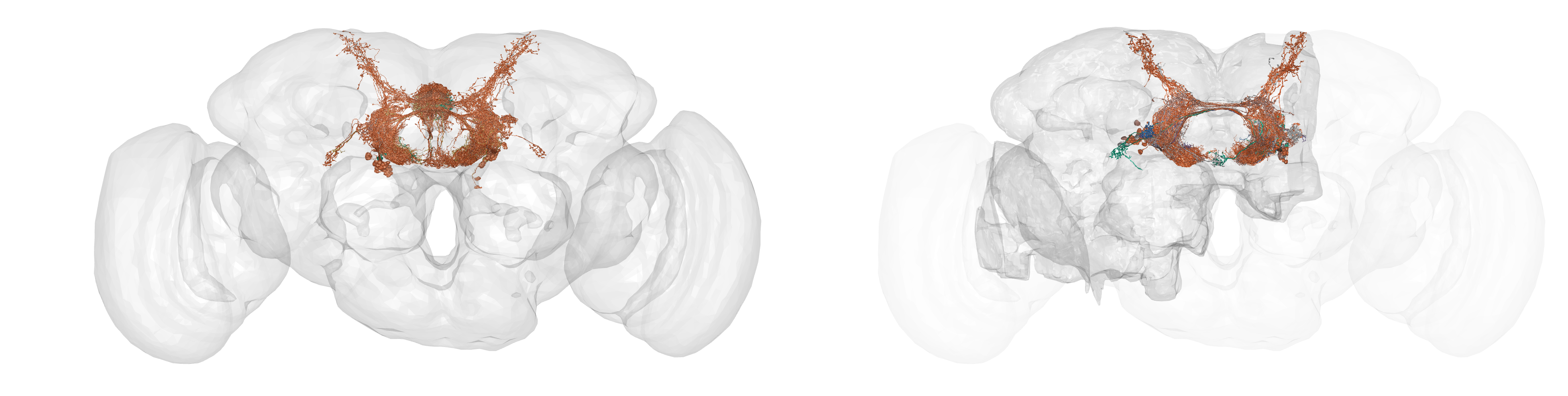

Supplement: Data S5. A .zip archive containing .png files depicting each of the 183 brain hemilineages we have used from the FAFB-FlyWire dataset, related to Figure 7 — Neurons in each hemilineage are colored by their neuron-level transmitter predictions, hemilineage names given in the file name. Hemilineage labels for the FAFB-FlyWire dataset are fully reported in Schlegel et al.S2 [file mmc6.zip › chosen_hemilineages/DM4_CX_d3__fafb_hemibrain.png]

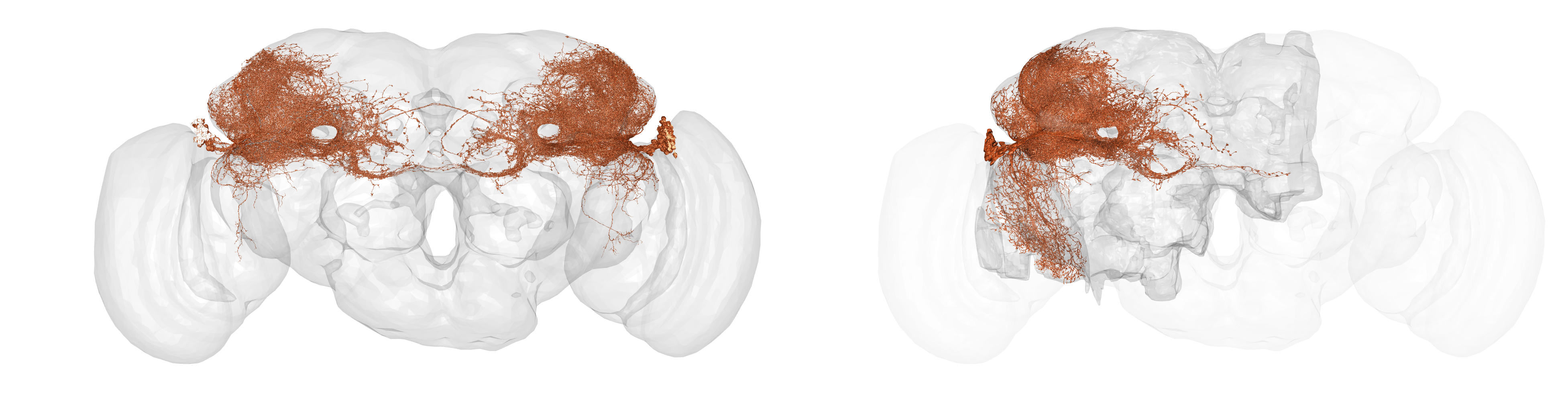

Supplement: Data S5. A .zip archive containing .png files depicting each of the 183 brain hemilineages we have used from the FAFB-FlyWire dataset, related to Figure 7 — Neurons in each hemilineage are colored by their neuron-level transmitter predictions, hemilineage names given in the file name. Hemilineage labels for the FAFB-FlyWire dataset are fully reported in Schlegel et al.S2 [file mmc6.zip › chosen_hemilineages/SLPav1_medial__fafb_hemibrain.png]

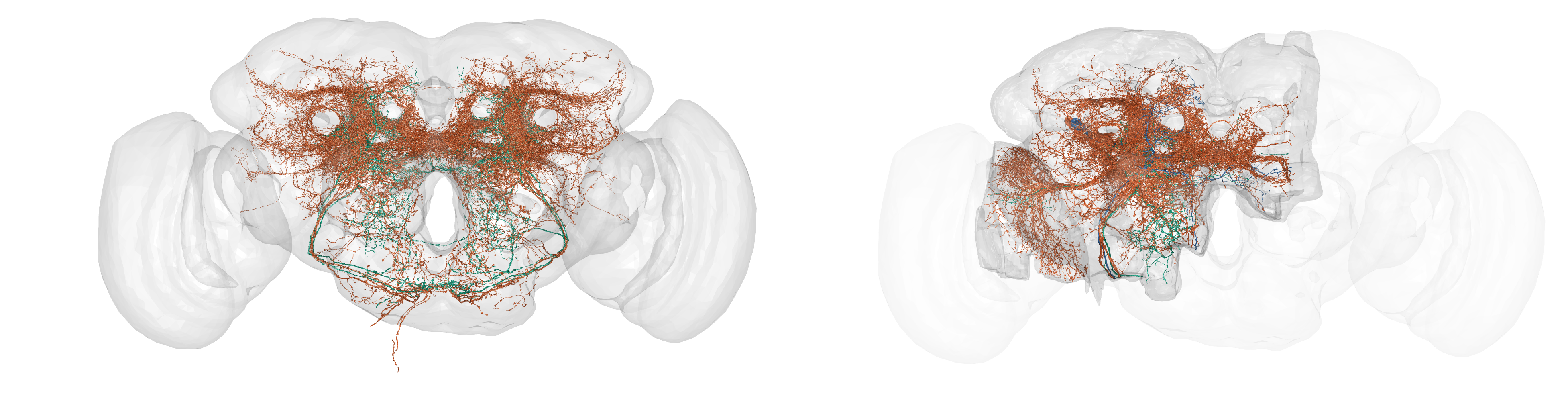

Supplement: Data S5. A .zip archive containing .png files depicting each of the 183 brain hemilineages we have used from the FAFB-FlyWire dataset, related to Figure 7 — Neurons in each hemilineage are colored by their neuron-level transmitter predictions, hemilineage names given in the file name. Hemilineage labels for the FAFB-FlyWire dataset are fully reported in Schlegel et al.S2 [file mmc6.zip › chosen_hemilineages/PSp3__fafb_hemibrain.png]

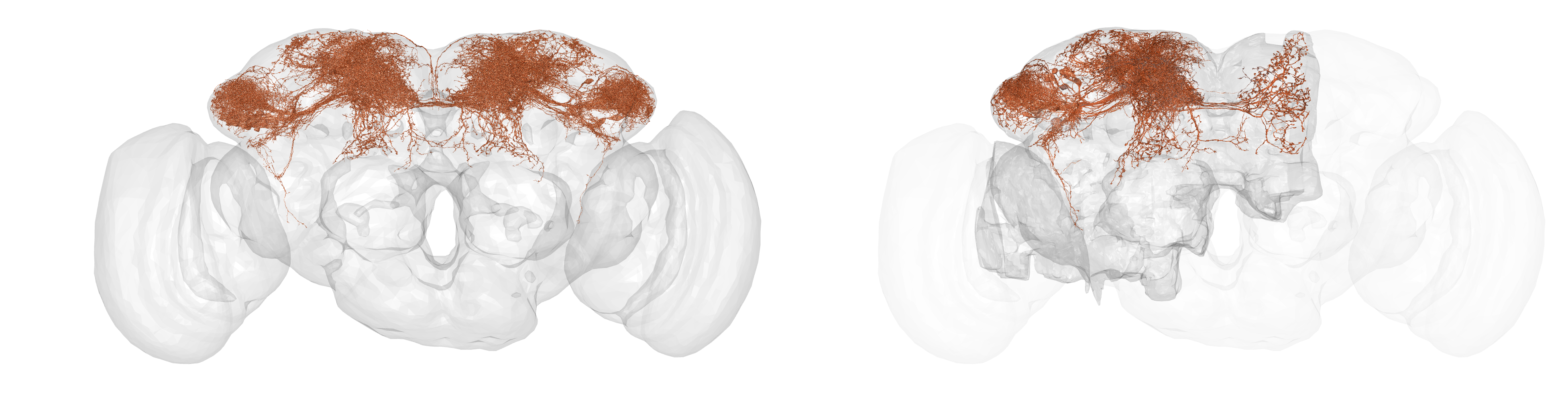

Supplement: Data S5. A .zip archive containing .png files depicting each of the 183 brain hemilineages we have used from the FAFB-FlyWire dataset, related to Figure 7 — Neurons in each hemilineage are colored by their neuron-level transmitter predictions, hemilineage names given in the file name. Hemilineage labels for the FAFB-FlyWire dataset are fully reported in Schlegel et al.S2 [file mmc6.zip › chosen_hemilineages/DL2_dorsal__fafb_hemibrain.png]

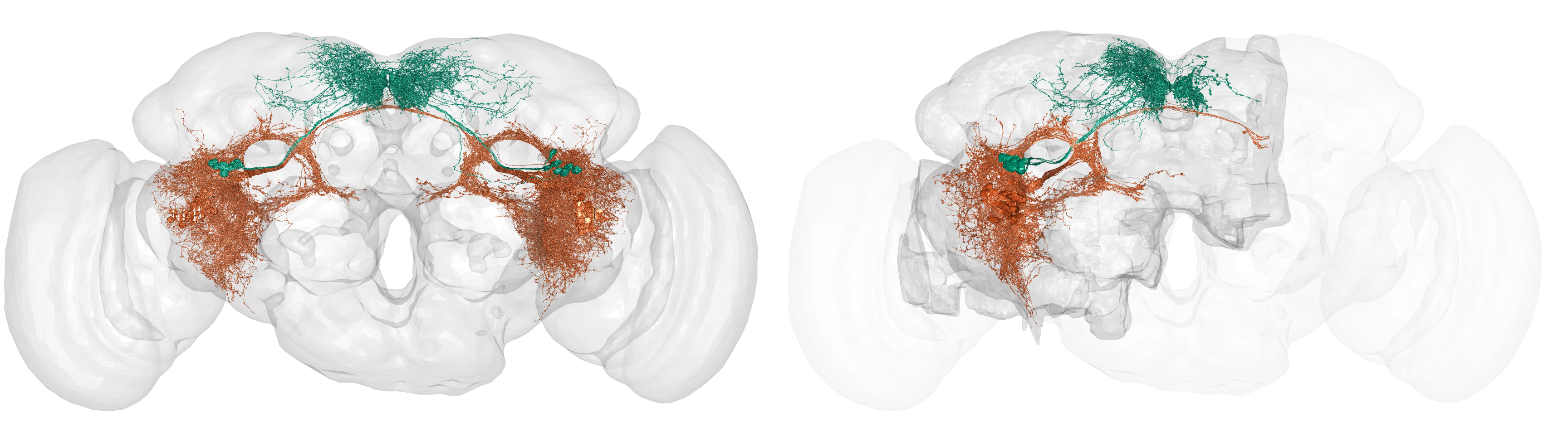

Supplement: Data S5. A .zip archive containing .png files depicting each of the 183 brain hemilineages we have used from the FAFB-FlyWire dataset, related to Figure 7 — Neurons in each hemilineage are colored by their neuron-level transmitter predictions, hemilineage names given in the file name. Hemilineage labels for the FAFB-FlyWire dataset are fully reported in Schlegel et al.S2 [file mmc6.zip › chosen_hemilineages/VLPa1_medial__fafb_hemibrain.png]

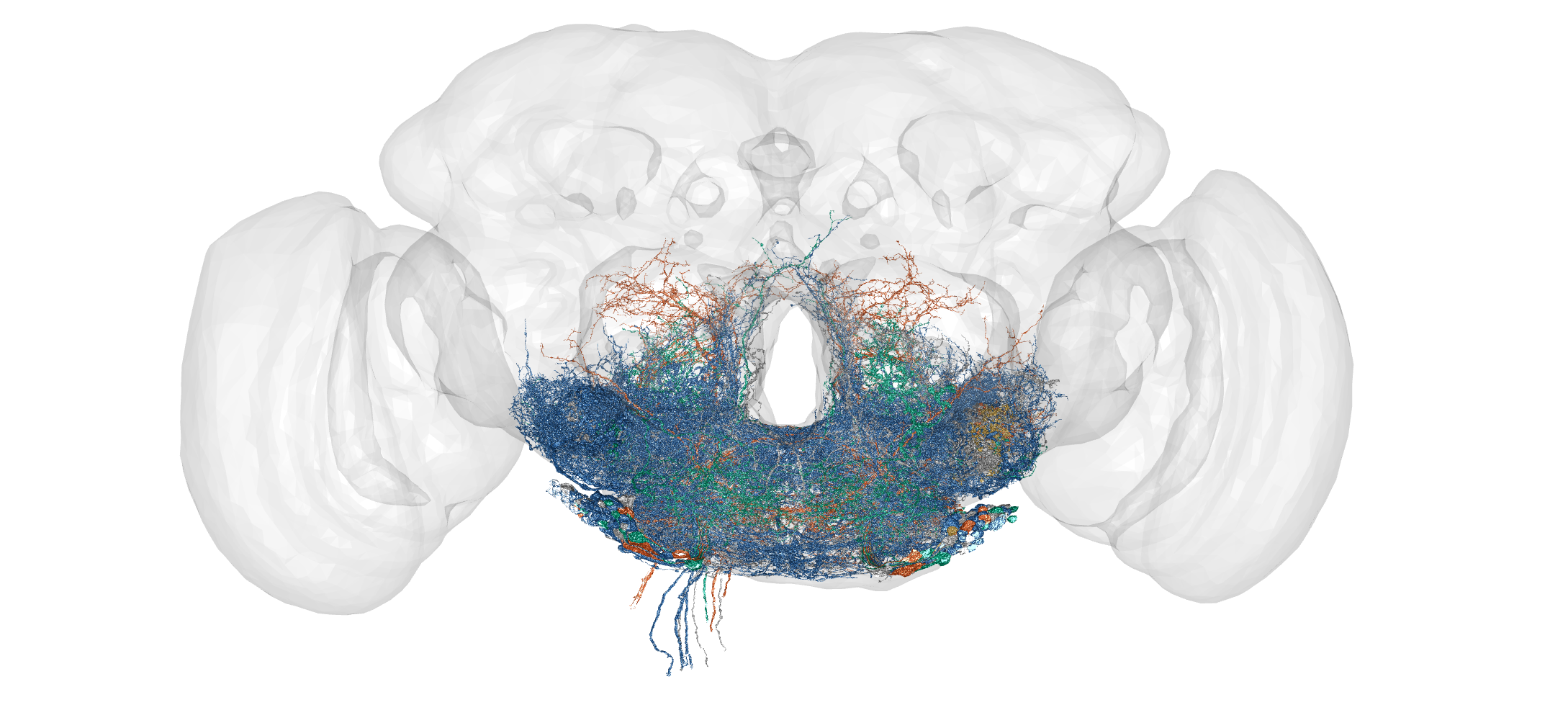

Supplement: Data S5. A .zip archive containing .png files depicting each of the 183 brain hemilineages we have used from the FAFB-FlyWire dataset, related to Figure 7 — Neurons in each hemilineage are colored by their neuron-level transmitter predictions, hemilineage names given in the file name. Hemilineage labels for the FAFB-FlyWire dataset are fully reported in Schlegel et al.S2 [file mmc6.zip › chosen_hemilineages/MD3__fafb.png]

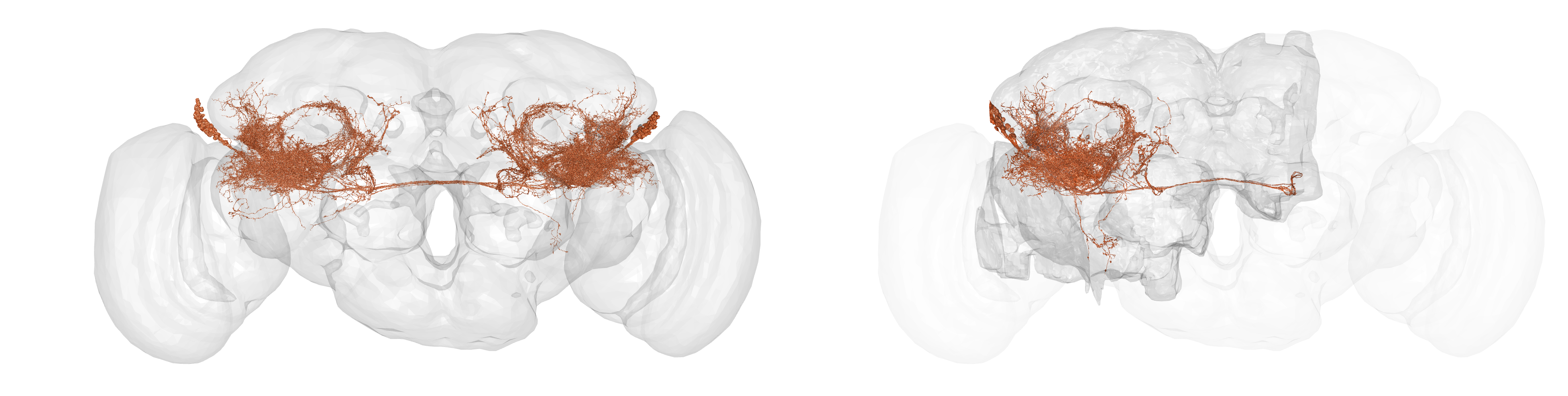

Supplement: Data S5. A .zip archive containing .png files depicting each of the 183 brain hemilineages we have used from the FAFB-FlyWire dataset, related to Figure 7 — Neurons in each hemilineage are colored by their neuron-level transmitter predictions, hemilineage names given in the file name. Hemilineage labels for the FAFB-FlyWire dataset are fully reported in Schlegel et al.S2 [file mmc6.zip › chosen_hemilineages/VPNp1_medial__fafb_hemibrain.png]

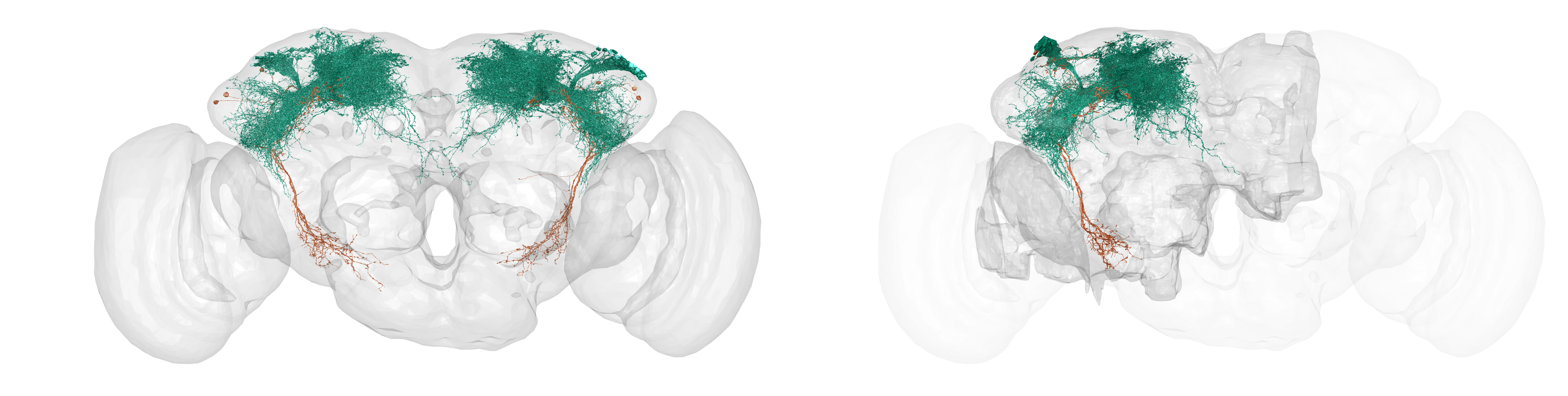

Supplement: Data S5. A .zip archive containing .png files depicting each of the 183 brain hemilineages we have used from the FAFB-FlyWire dataset, related to Figure 7 — Neurons in each hemilineage are colored by their neuron-level transmitter predictions, hemilineage names given in the file name. Hemilineage labels for the FAFB-FlyWire dataset are fully reported in Schlegel et al.S2 [file mmc6.zip › chosen_hemilineages/SLPad1_posterior__fafb_hemibrain.png]

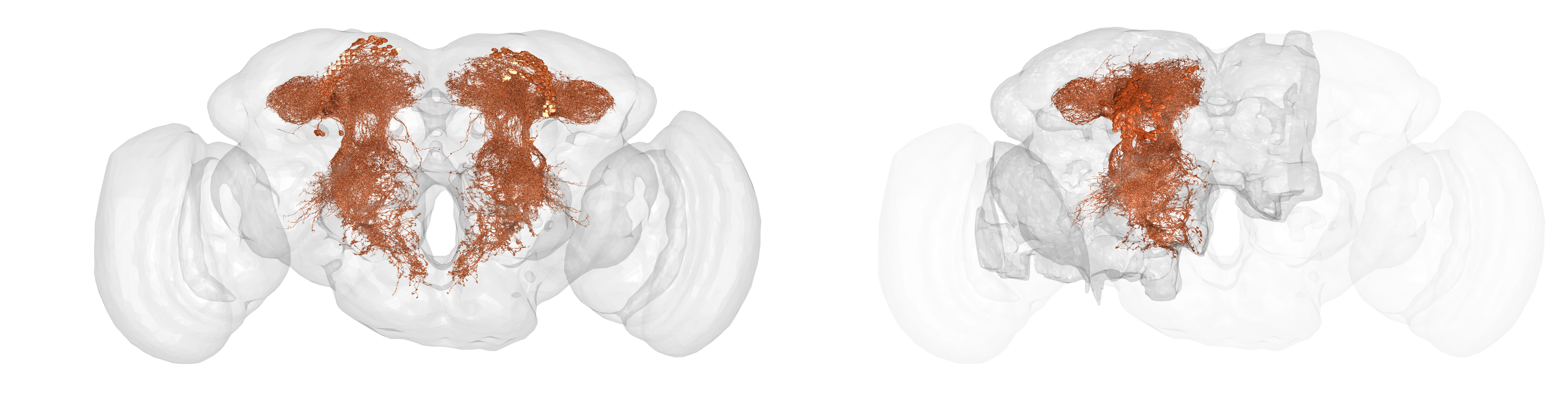

Supplement: Data S5. A .zip archive containing .png files depicting each of the 183 brain hemilineages we have used from the FAFB-FlyWire dataset, related to Figure 7 — Neurons in each hemilineage are colored by their neuron-level transmitter predictions, hemilineage names given in the file name. Hemilineage labels for the FAFB-FlyWire dataset are fully reported in Schlegel et al.S2 [file mmc6.zip › chosen_hemilineages/CREa2_ventral__fafb_hemibrain.png]

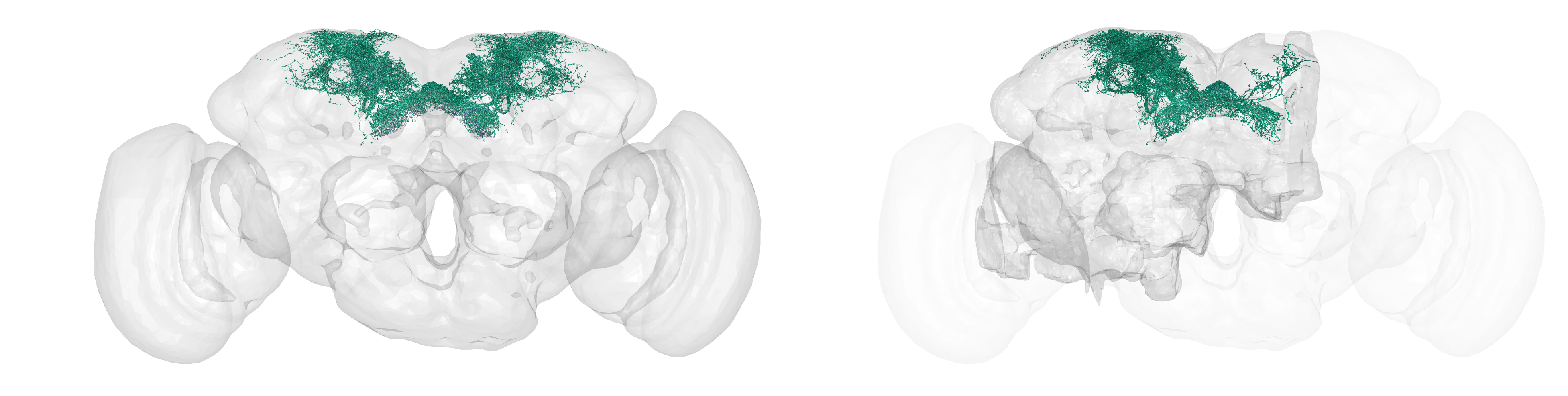

Supplement: Data S5. A .zip archive containing .png files depicting each of the 183 brain hemilineages we have used from the FAFB-FlyWire dataset, related to Figure 7 — Neurons in each hemilineage are colored by their neuron-level transmitter predictions, hemilineage names given in the file name. Hemilineage labels for the FAFB-FlyWire dataset are fully reported in Schlegel et al.S2 [file mmc6.zip › chosen_hemilineages/SIPp1__fafb_hemibrain.png]

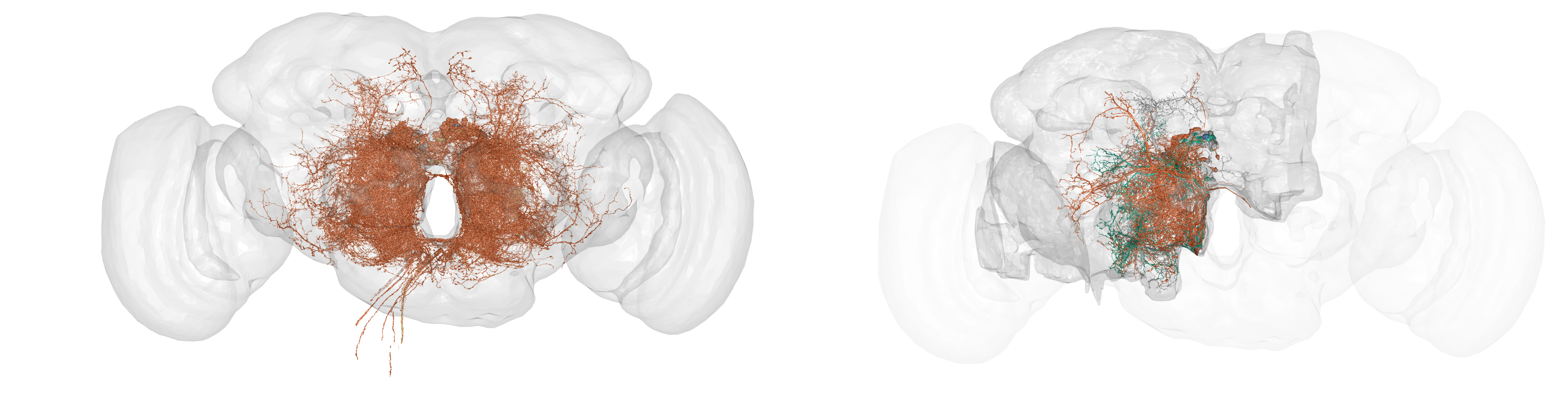

Supplement: Data S5. A .zip archive containing .png files depicting each of the 183 brain hemilineages we have used from the FAFB-FlyWire dataset, related to Figure 7 — Neurons in each hemilineage are colored by their neuron-level transmitter predictions, hemilineage names given in the file name. Hemilineage labels for the FAFB-FlyWire dataset are fully reported in Schlegel et al.S2 [file mmc6.zip › chosen_hemilineages/PSp2__fafb_hemibrain.png]

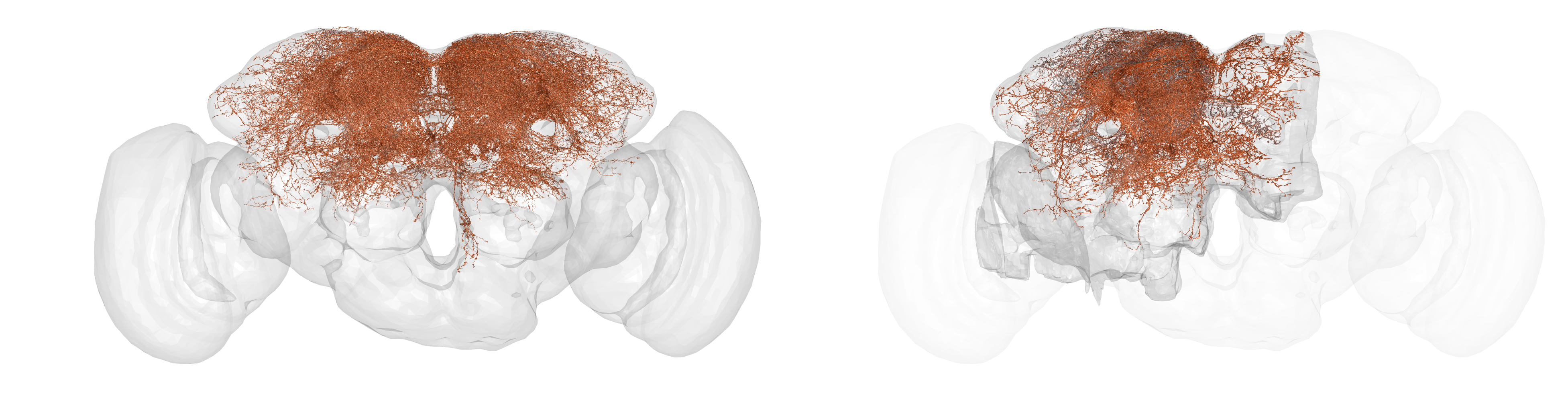

Supplement: Data S5. A .zip archive containing .png files depicting each of the 183 brain hemilineages we have used from the FAFB-FlyWire dataset, related to Figure 7 — Neurons in each hemilineage are colored by their neuron-level transmitter predictions, hemilineage names given in the file name. Hemilineage labels for the FAFB-FlyWire dataset are fully reported in Schlegel et al.S2 [file mmc6.zip › chosen_hemilineages/DM4_dorsal__fafb_hemibrain.png]

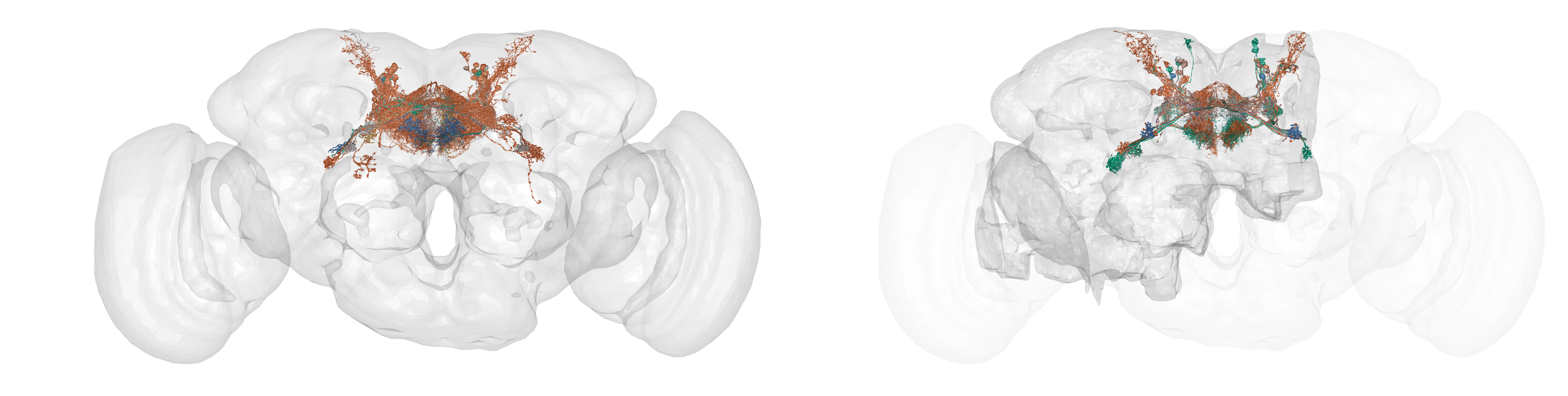

Supplement: Data S5. A .zip archive containing .png files depicting each of the 183 brain hemilineages we have used from the FAFB-FlyWire dataset, related to Figure 7 — Neurons in each hemilineage are colored by their neuron-level transmitter predictions, hemilineage names given in the file name. Hemilineage labels for the FAFB-FlyWire dataset are fully reported in Schlegel et al.S2 [file mmc6.zip › chosen_hemilineages/DM3_CX_d2__fafb_hemibrain.png]

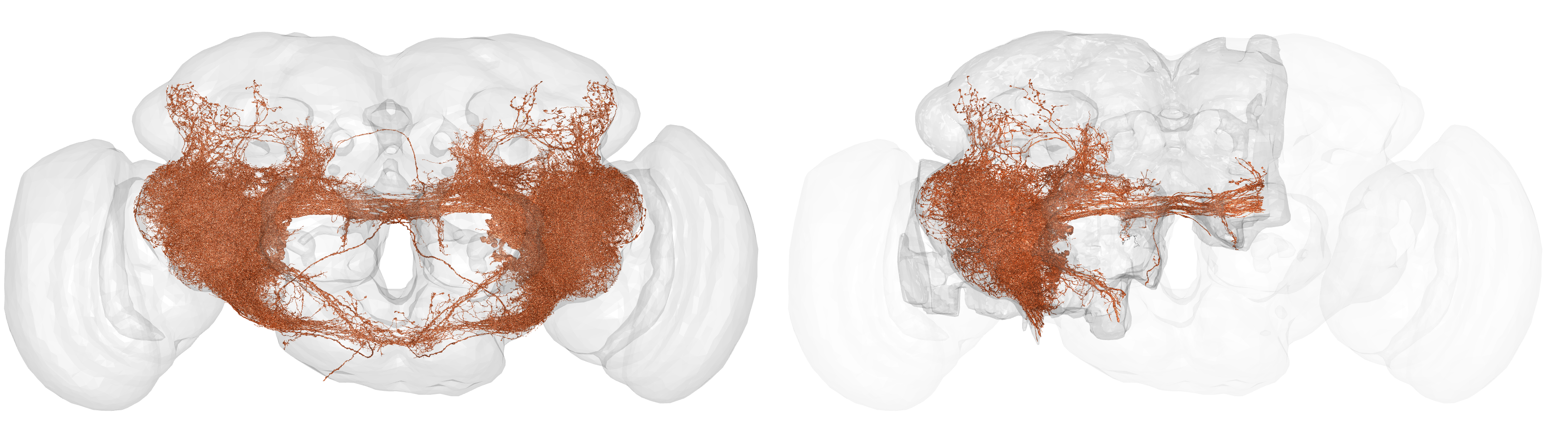

Supplement: Data S5. A .zip archive containing .png files depicting each of the 183 brain hemilineages we have used from the FAFB-FlyWire dataset, related to Figure 7 — Neurons in each hemilineage are colored by their neuron-level transmitter predictions, hemilineage names given in the file name. Hemilineage labels for the FAFB-FlyWire dataset are fully reported in Schlegel et al.S2 [file mmc6.zip › chosen_hemilineages/VLPl&p2_posterior__fafb_hemibrain.png]

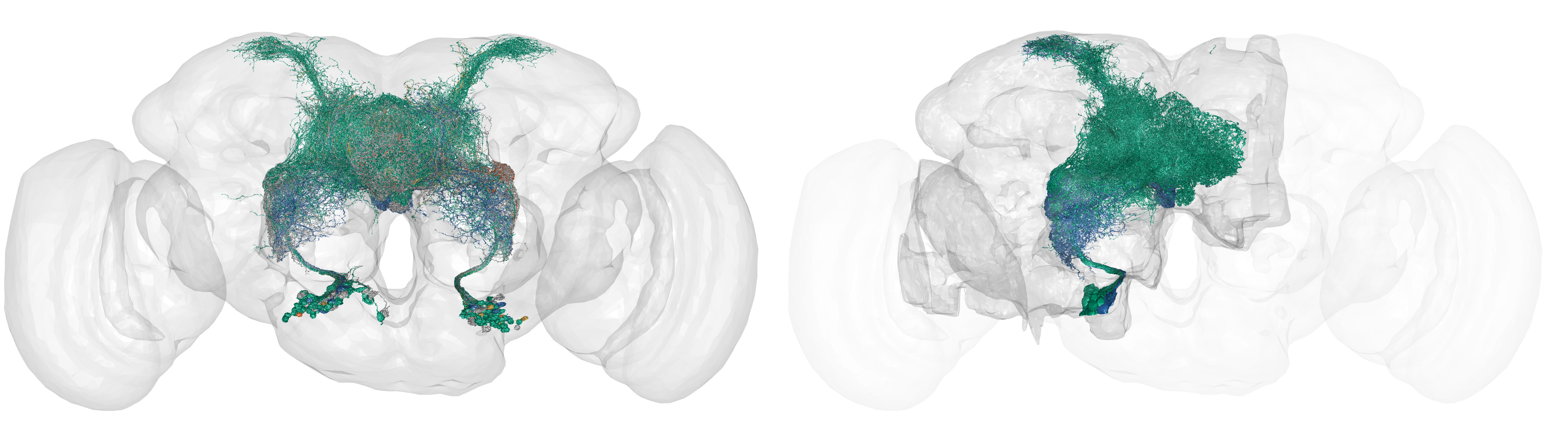

Supplement: Data S5. A .zip archive containing .png files depicting each of the 183 brain hemilineages we have used from the FAFB-FlyWire dataset, related to Figure 7 — Neurons in each hemilineage are colored by their neuron-level transmitter predictions, hemilineage names given in the file name. Hemilineage labels for the FAFB-FlyWire dataset are fully reported in Schlegel et al.S2 [file mmc6.zip › chosen_hemilineages/LALv1_dorsal__fafb_hemibrain.png]

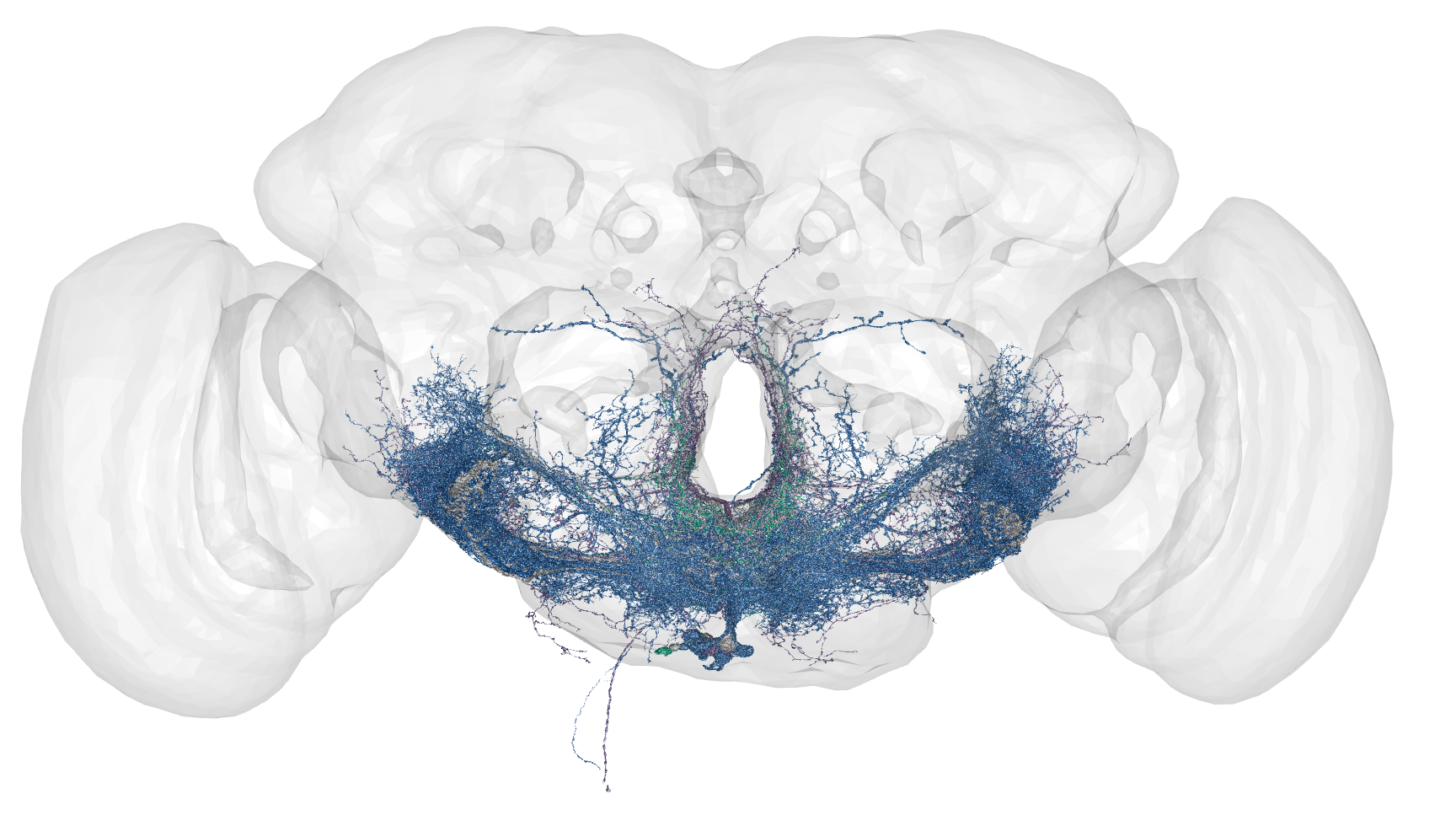

Supplement: Data S5. A .zip archive containing .png files depicting each of the 183 brain hemilineages we have used from the FAFB-FlyWire dataset, related to Figure 7 — Neurons in each hemilineage are colored by their neuron-level transmitter predictions, hemilineage names given in the file name. Hemilineage labels for the FAFB-FlyWire dataset are fully reported in Schlegel et al.S2 [file mmc6.zip › chosen_hemilineages/LB0_anterior__fafb.png]

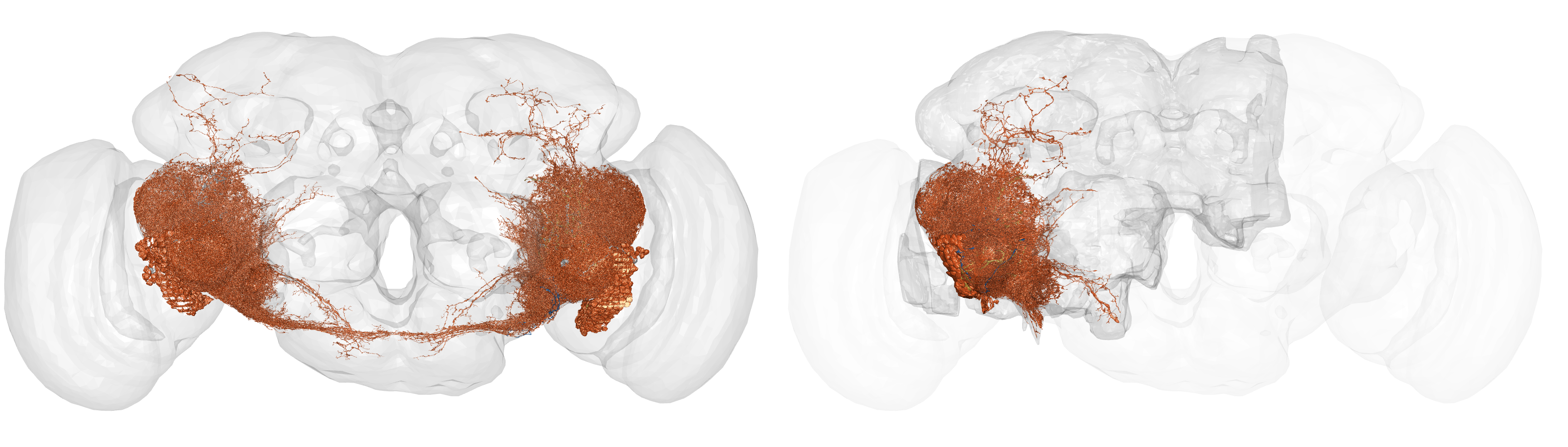

Supplement: Data S5. A .zip archive containing .png files depicting each of the 183 brain hemilineages we have used from the FAFB-FlyWire dataset, related to Figure 7 — Neurons in each hemilineage are colored by their neuron-level transmitter predictions, hemilineage names given in the file name. Hemilineage labels for the FAFB-FlyWire dataset are fully reported in Schlegel et al.S2 [file mmc6.zip › chosen_hemilineages/VLPl1_or_VLPl5__fafb_hemibrain.png]

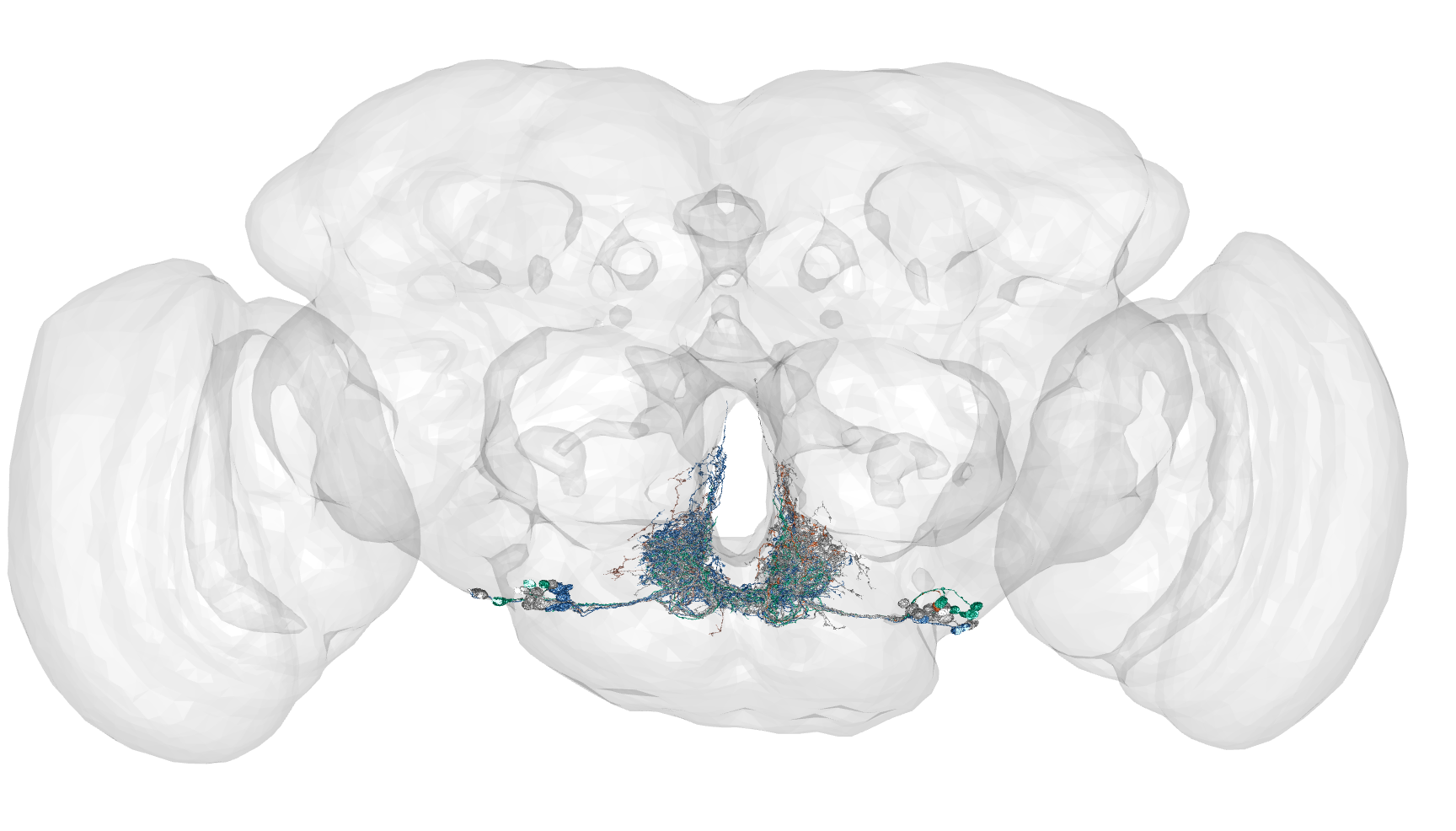

Supplement: Data S5. A .zip archive containing .png files depicting each of the 183 brain hemilineages we have used from the FAFB-FlyWire dataset, related to Figure 7 — Neurons in each hemilineage are colored by their neuron-level transmitter predictions, hemilineage names given in the file name. Hemilineage labels for the FAFB-FlyWire dataset are fully reported in Schlegel et al.S2 [file mmc6.zip › chosen_hemilineages/TRdl_a__fafb.png]

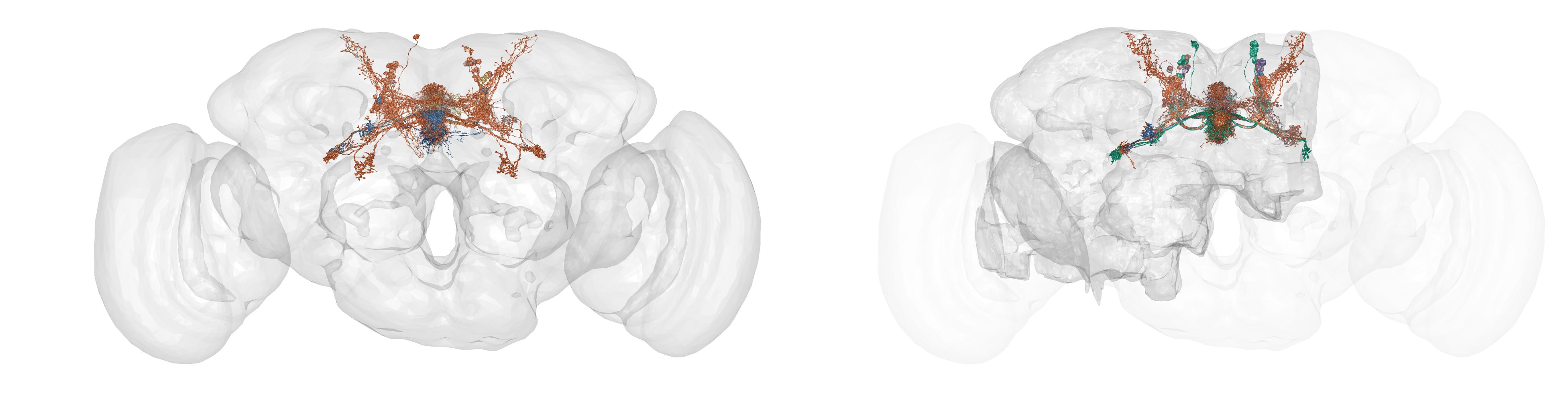

Supplement: Data S5. A .zip archive containing .png files depicting each of the 183 brain hemilineages we have used from the FAFB-FlyWire dataset, related to Figure 7 — Neurons in each hemilineage are colored by their neuron-level transmitter predictions, hemilineage names given in the file name. Hemilineage labels for the FAFB-FlyWire dataset are fully reported in Schlegel et al.S2 [file mmc6.zip › chosen_hemilineages/DM3_CX_d1__fafb_hemibrain.png]

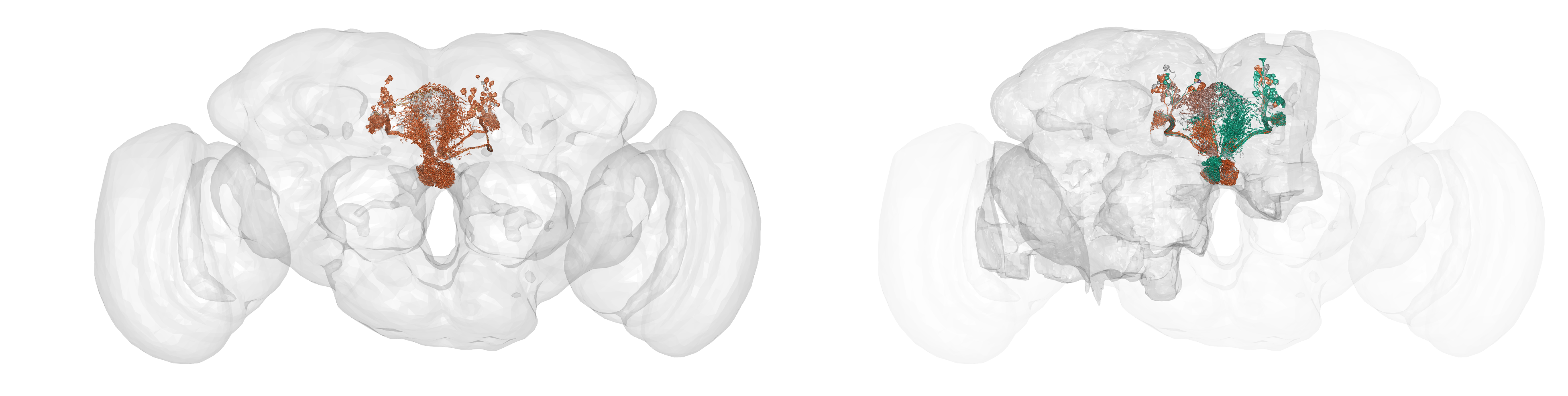

Supplement: Data S5. A .zip archive containing .png files depicting each of the 183 brain hemilineages we have used from the FAFB-FlyWire dataset, related to Figure 7 — Neurons in each hemilineage are colored by their neuron-level transmitter predictions, hemilineage names given in the file name. Hemilineage labels for the FAFB-FlyWire dataset are fully reported in Schlegel et al.S2 [file mmc6.zip › chosen_hemilineages/DM3_CX_v__fafb_hemibrain.png]

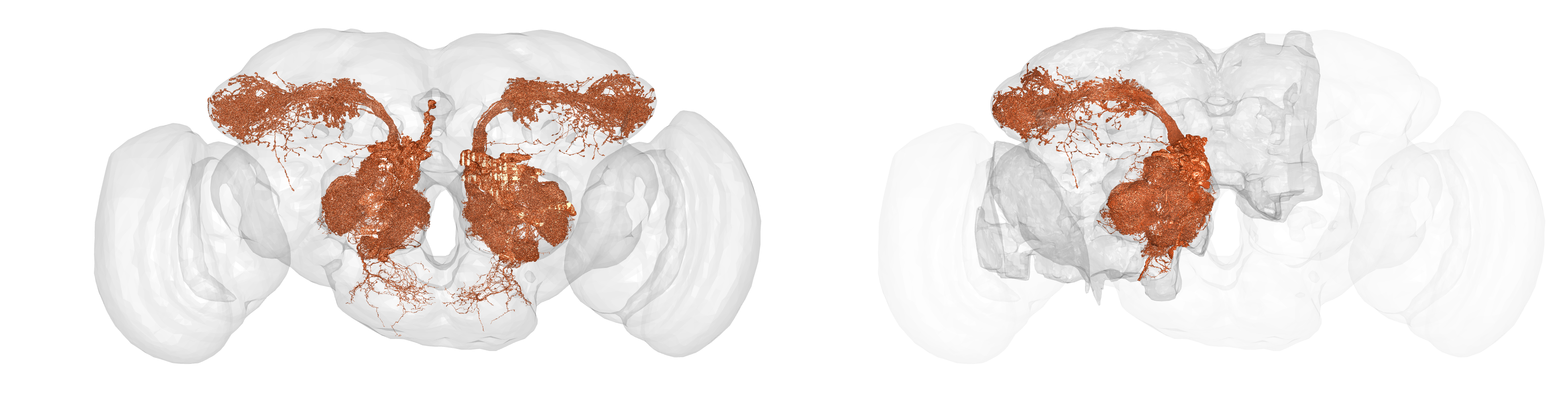

Supplement: Data S5. A .zip archive containing .png files depicting each of the 183 brain hemilineages we have used from the FAFB-FlyWire dataset, related to Figure 7 — Neurons in each hemilineage are colored by their neuron-level transmitter predictions, hemilineage names given in the file name. Hemilineage labels for the FAFB-FlyWire dataset are fully reported in Schlegel et al.S2 [file mmc6.zip › chosen_hemilineages/ALad1__fafb_hemibrain.png]

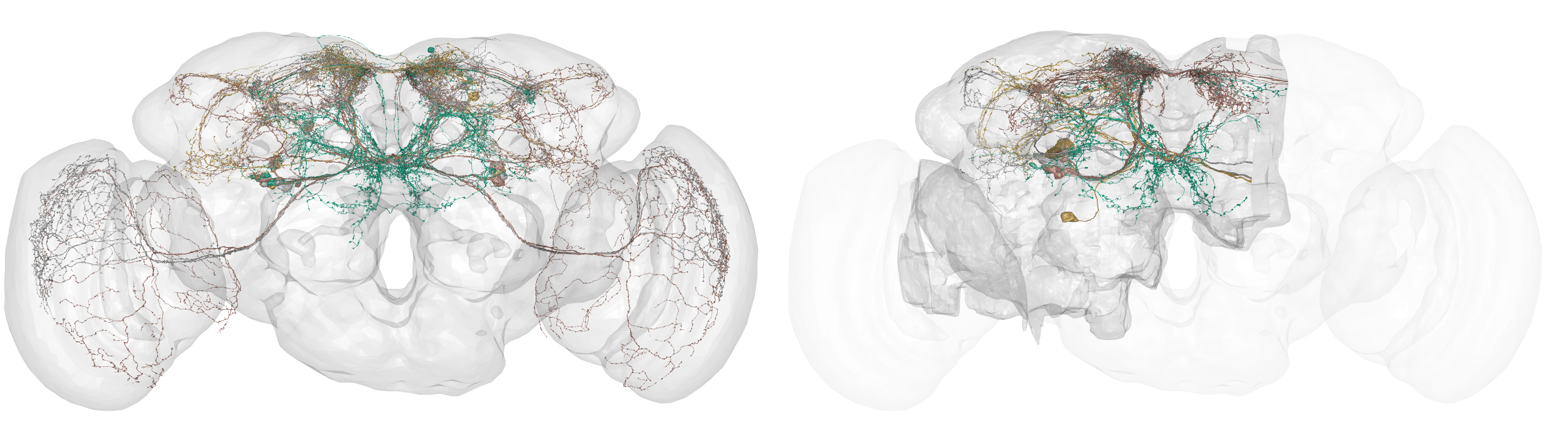

Supplement: Data S5. A .zip archive containing .png files depicting each of the 183 brain hemilineages we have used from the FAFB-FlyWire dataset, related to Figure 7 — Neurons in each hemilineage are colored by their neuron-level transmitter predictions, hemilineage names given in the file name. Hemilineage labels for the FAFB-FlyWire dataset are fully reported in Schlegel et al.S2 [file mmc6.zip › chosen_hemilineages/SMPp&v1_ventral__fafb_hemibrain.png]

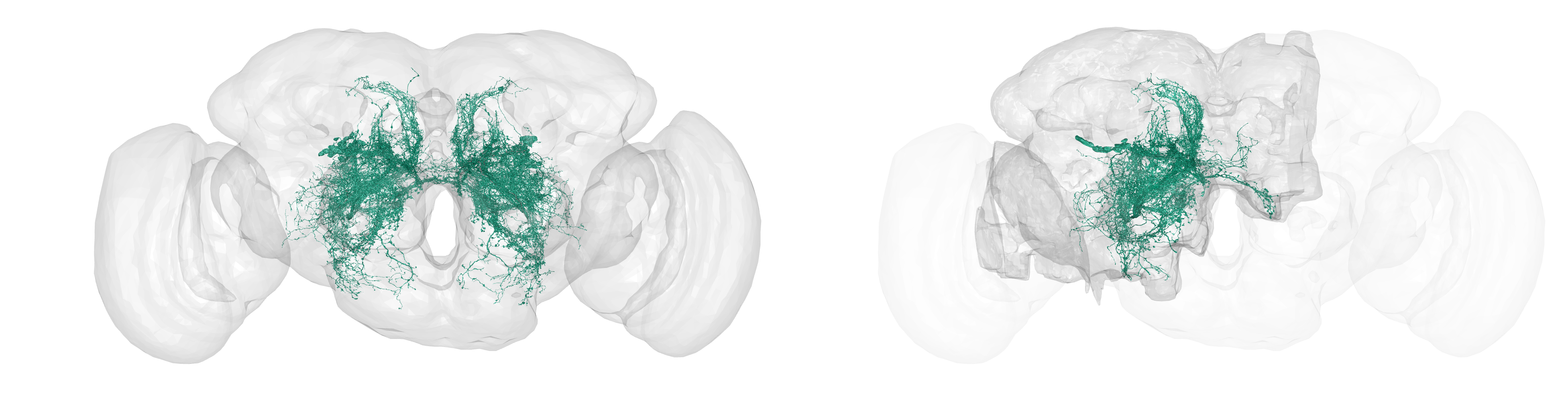

Supplement: Data S5. A .zip archive containing .png files depicting each of the 183 brain hemilineages we have used from the FAFB-FlyWire dataset, related to Figure 7 — Neurons in each hemilineage are colored by their neuron-level transmitter predictions, hemilineage names given in the file name. Hemilineage labels for the FAFB-FlyWire dataset are fully reported in Schlegel et al.S2 [file mmc6.zip › chosen_hemilineages/PSp1__fafb_hemibrain.png]

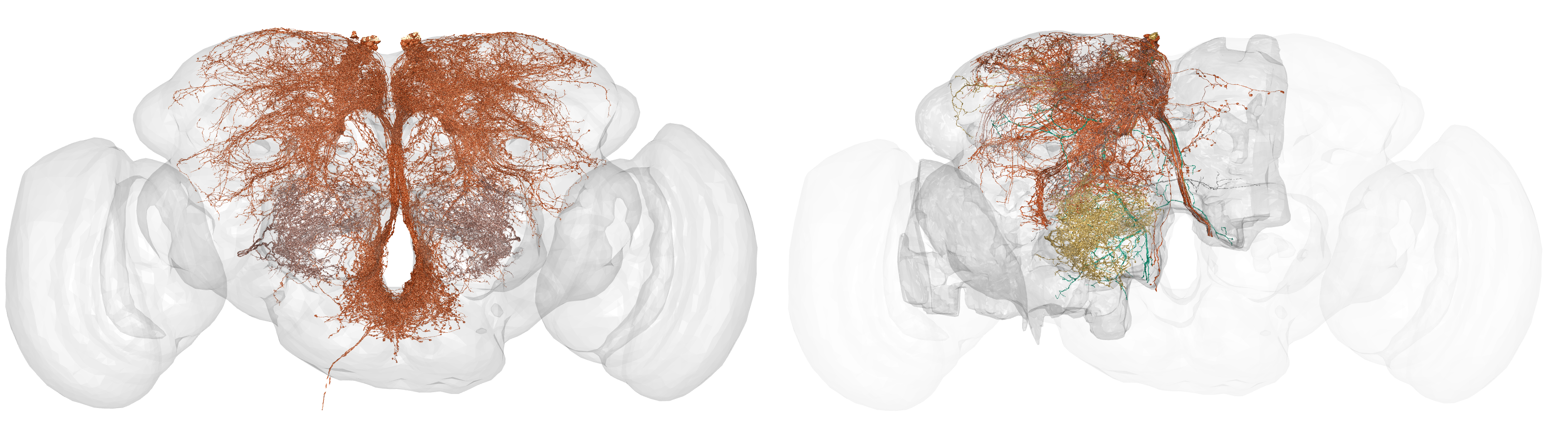

Supplement: Data S5. A .zip archive containing .png files depicting each of the 183 brain hemilineages we have used from the FAFB-FlyWire dataset, related to Figure 7 — Neurons in each hemilineage are colored by their neuron-level transmitter predictions, hemilineage names given in the file name. Hemilineage labels for the FAFB-FlyWire dataset are fully reported in Schlegel et al.S2 [file mmc6.zip › chosen_hemilineages/SMPpm1__fafb_hemibrain.png]

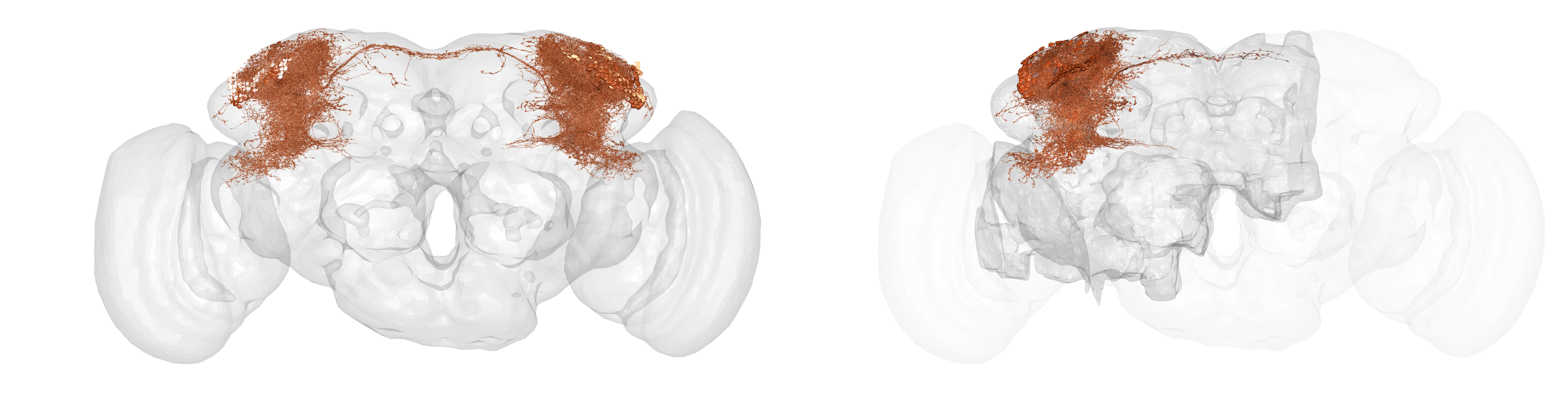

Supplement: Data S5. A .zip archive containing .png files depicting each of the 183 brain hemilineages we have used from the FAFB-FlyWire dataset, related to Figure 7 — Neurons in each hemilineage are colored by their neuron-level transmitter predictions, hemilineage names given in the file name. Hemilineage labels for the FAFB-FlyWire dataset are fully reported in Schlegel et al.S2 [file mmc6.zip › chosen_hemilineages/SLPad1_anterior__fafb_hemibrain.png]

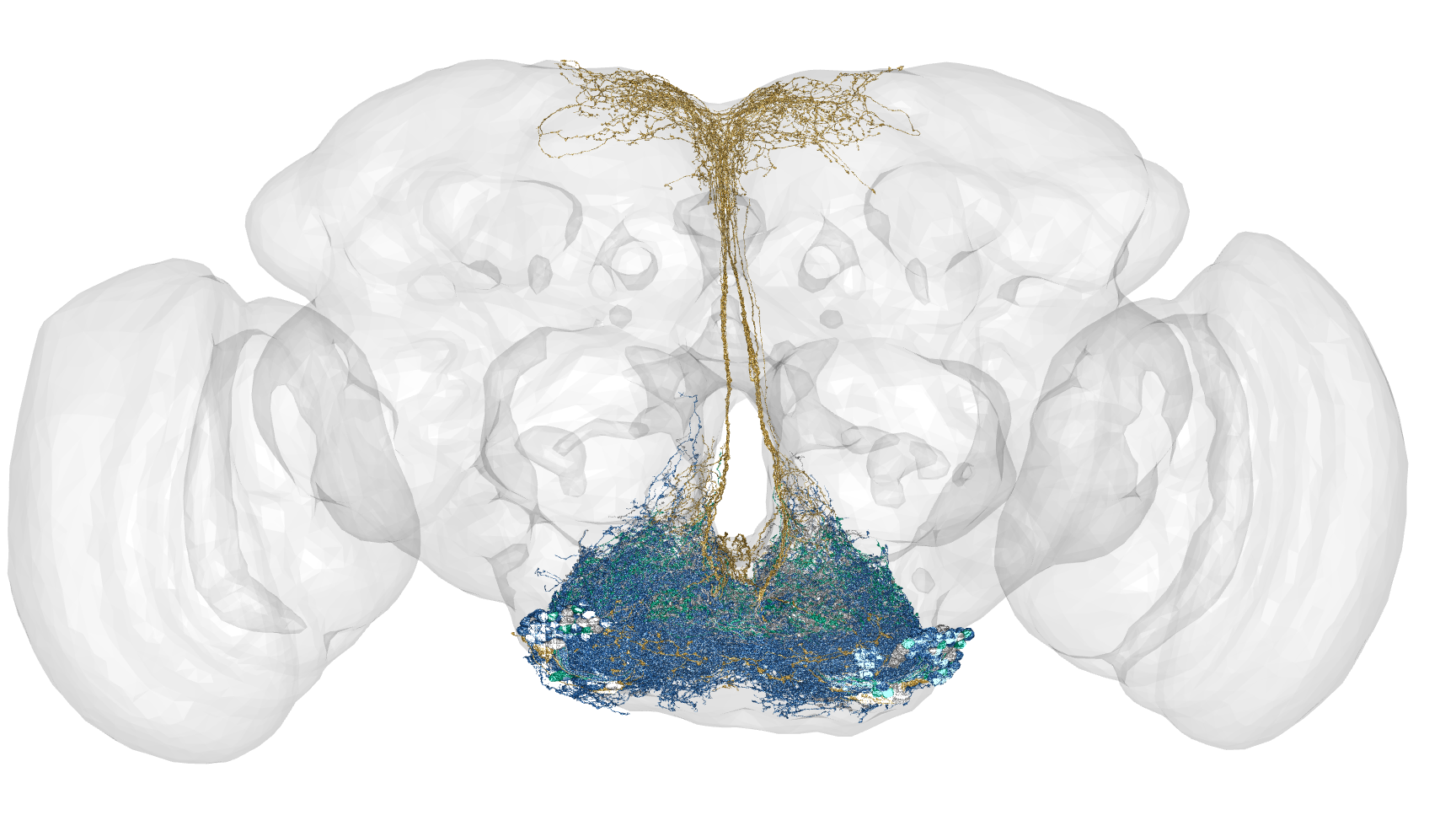

Supplement: Data S5. A .zip archive containing .png files depicting each of the 183 brain hemilineages we have used from the FAFB-FlyWire dataset, related to Figure 7 — Neurons in each hemilineage are colored by their neuron-level transmitter predictions, hemilineage names given in the file name. Hemilineage labels for the FAFB-FlyWire dataset are fully reported in Schlegel et al.S2 [file mmc6.zip › chosen_hemilineages/TRdm__fafb.png]

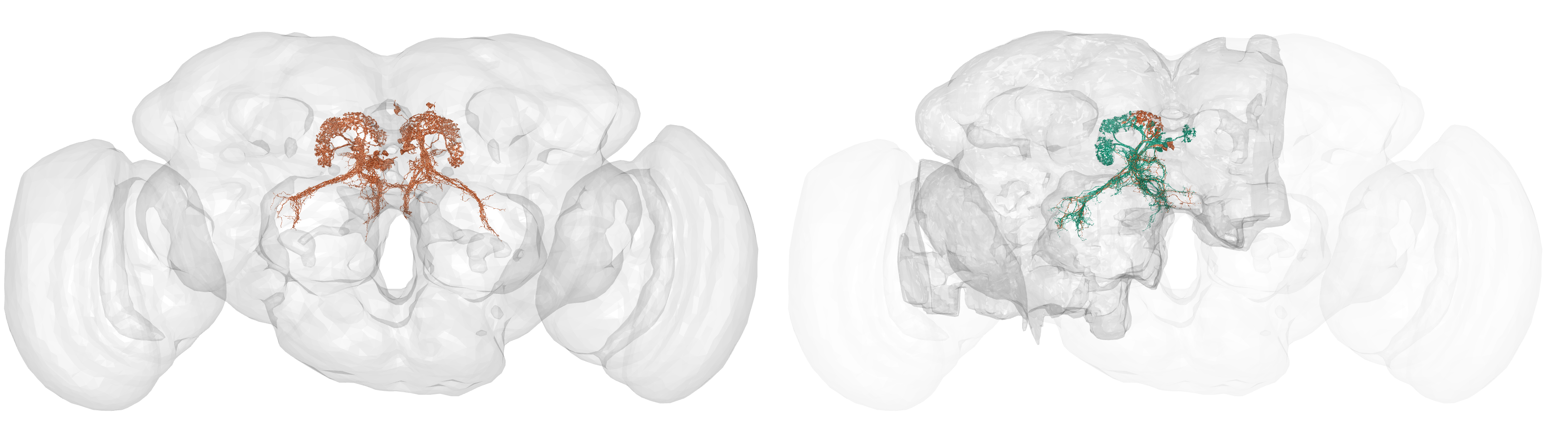

Supplement: Data S5. A .zip archive containing .png files depicting each of the 183 brain hemilineages we have used from the FAFB-FlyWire dataset, related to Figure 7 — Neurons in each hemilineage are colored by their neuron-level transmitter predictions, hemilineage names given in the file name. Hemilineage labels for the FAFB-FlyWire dataset are fully reported in Schlegel et al.S2 [file mmc6.zip › chosen_hemilineages/DM6_IbSpsP__fafb_hemibrain.png]

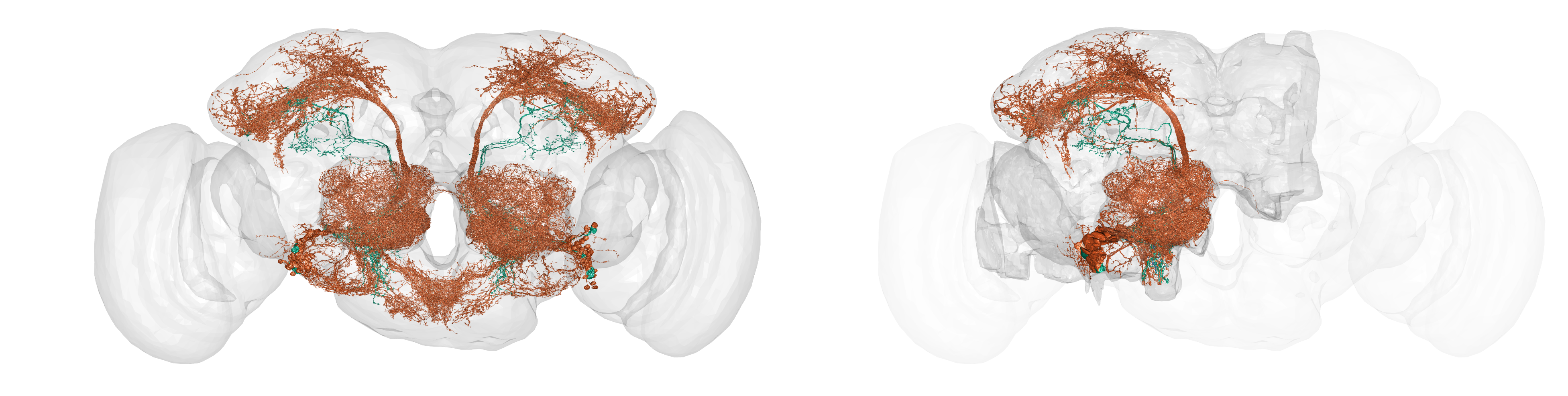

Supplement: Data S5. A .zip archive containing .png files depicting each of the 183 brain hemilineages we have used from the FAFB-FlyWire dataset, related to Figure 7 — Neurons in each hemilineage are colored by their neuron-level transmitter predictions, hemilineage names given in the file name. Hemilineage labels for the FAFB-FlyWire dataset are fully reported in Schlegel et al.S2 [file mmc6.zip › chosen_hemilineages/ALlv1__fafb_hemibrain.png]

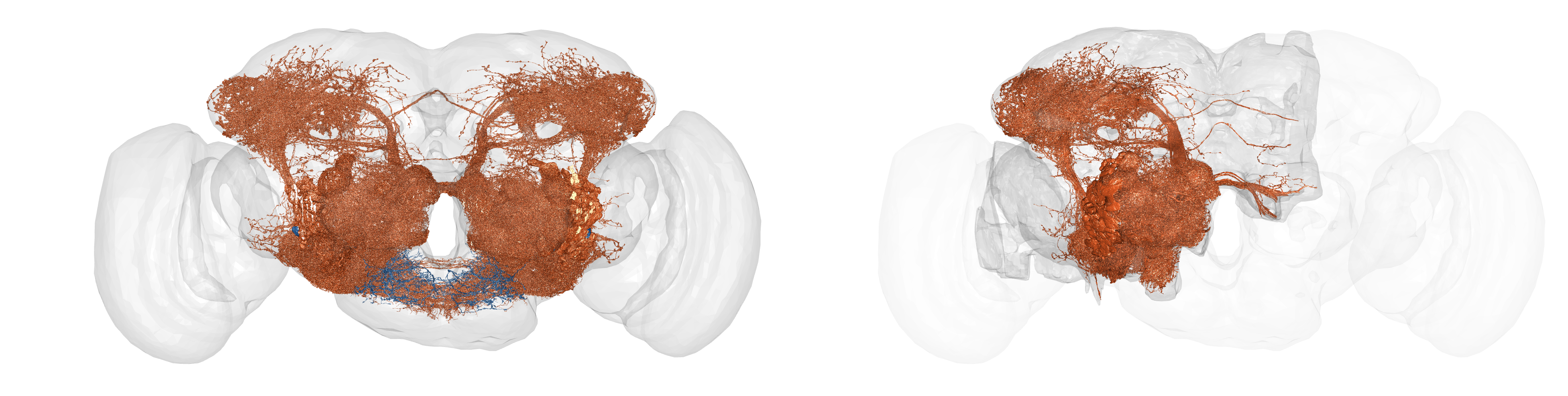

Supplement: Data S5. A .zip archive containing .png files depicting each of the 183 brain hemilineages we have used from the FAFB-FlyWire dataset, related to Figure 7 — Neurons in each hemilineage are colored by their neuron-level transmitter predictions, hemilineage names given in the file name. Hemilineage labels for the FAFB-FlyWire dataset are fully reported in Schlegel et al.S2 [file mmc6.zip › chosen_hemilineages/ALl1_ventral__fafb_hemibrain.png]

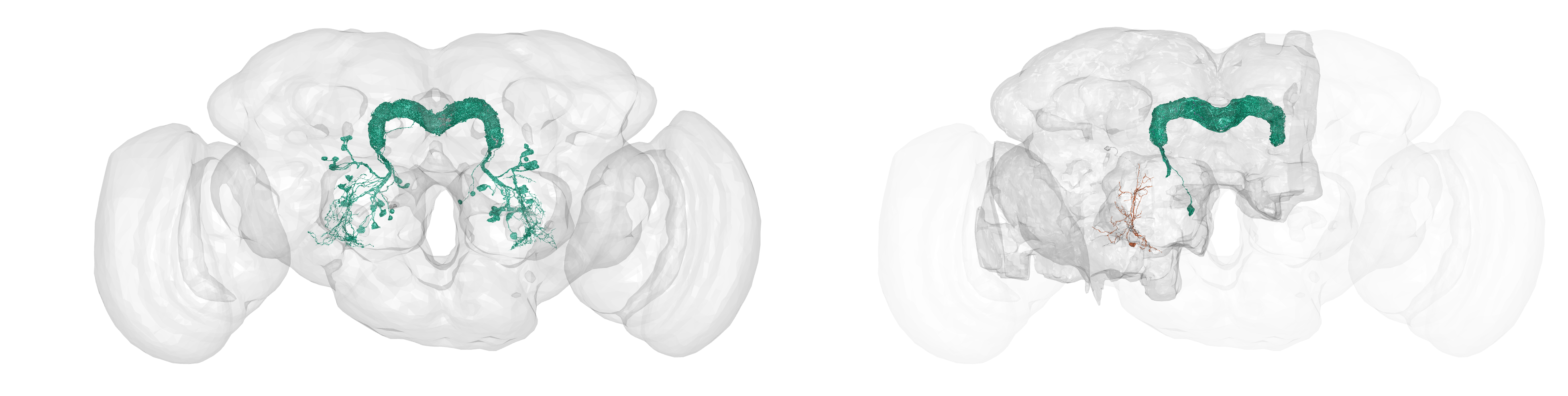

Supplement: Data S5. A .zip archive containing .png files depicting each of the 183 brain hemilineages we have used from the FAFB-FlyWire dataset, related to Figure 7 — Neurons in each hemilineage are colored by their neuron-level transmitter predictions, hemilineage names given in the file name. Hemilineage labels for the FAFB-FlyWire dataset are fully reported in Schlegel et al.S2 [file mmc6.zip › chosen_hemilineages/PBp1__fafb_hemibrain.png]

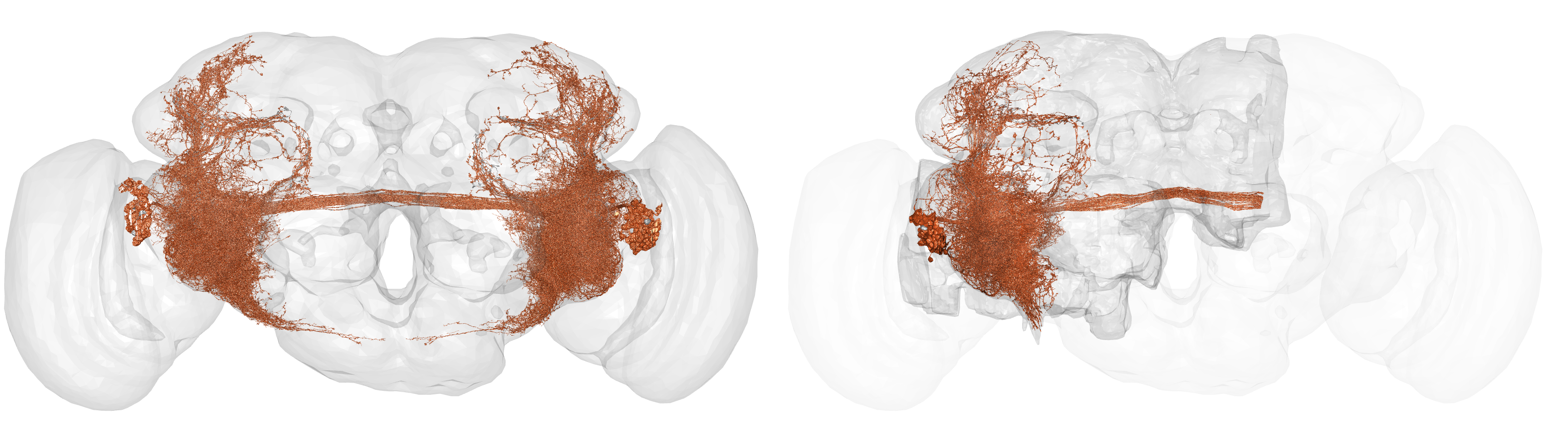

Supplement: Data S5. A .zip archive containing .png files depicting each of the 183 brain hemilineages we have used from the FAFB-FlyWire dataset, related to Figure 7 — Neurons in each hemilineage are colored by their neuron-level transmitter predictions, hemilineage names given in the file name. Hemilineage labels for the FAFB-FlyWire dataset are fully reported in Schlegel et al.S2 [file mmc6.zip › chosen_hemilineages/VLPl&d1_lateral__fafb_hemibrain.png]

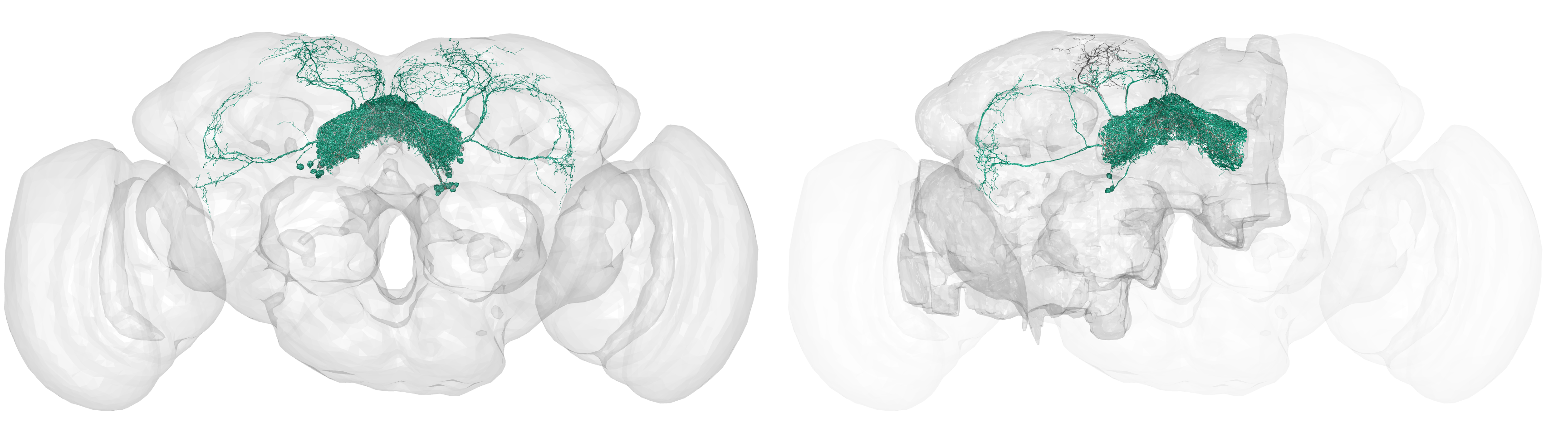

Supplement: Data S5. A .zip archive containing .png files depicting each of the 183 brain hemilineages we have used from the FAFB-FlyWire dataset, related to Figure 7 — Neurons in each hemilineage are colored by their neuron-level transmitter predictions, hemilineage names given in the file name. Hemilineage labels for the FAFB-FlyWire dataset are fully reported in Schlegel et al.S2 [file mmc6.zip › chosen_hemilineages/DM6_dorso_medial__fafb_hemibrain.png]

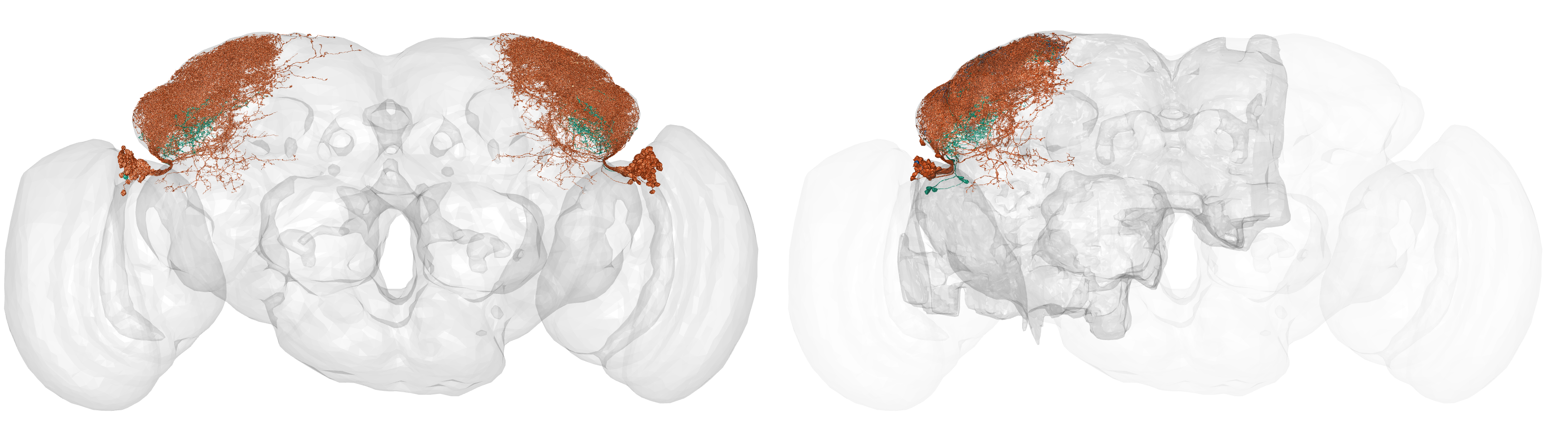

Supplement: Data S5. A .zip archive containing .png files depicting each of the 183 brain hemilineages we have used from the FAFB-FlyWire dataset, related to Figure 7 — Neurons in each hemilineage are colored by their neuron-level transmitter predictions, hemilineage names given in the file name. Hemilineage labels for the FAFB-FlyWire dataset are fully reported in Schlegel et al.S2 [file mmc6.zip › chosen_hemilineages/LHa3__fafb_hemibrain.png]

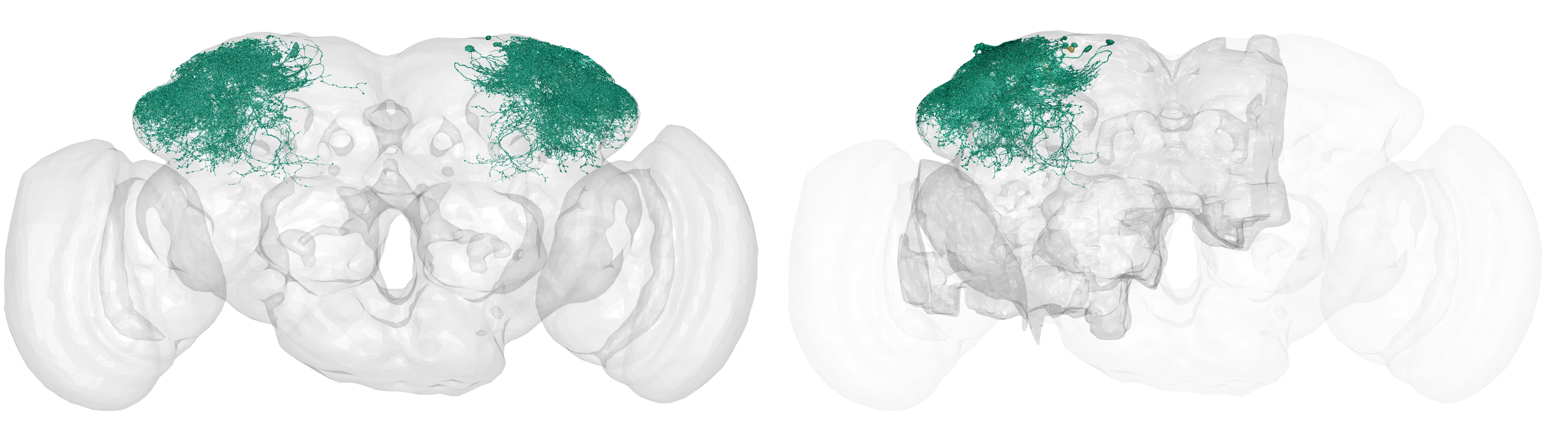

Supplement: Data S5. A .zip archive containing .png files depicting each of the 183 brain hemilineages we have used from the FAFB-FlyWire dataset, related to Figure 7 — Neurons in each hemilineage are colored by their neuron-level transmitter predictions, hemilineage names given in the file name. Hemilineage labels for the FAFB-FlyWire dataset are fully reported in Schlegel et al.S2 [file mmc6.zip › chosen_hemilineages/LHd2__fafb_hemibrain.png]

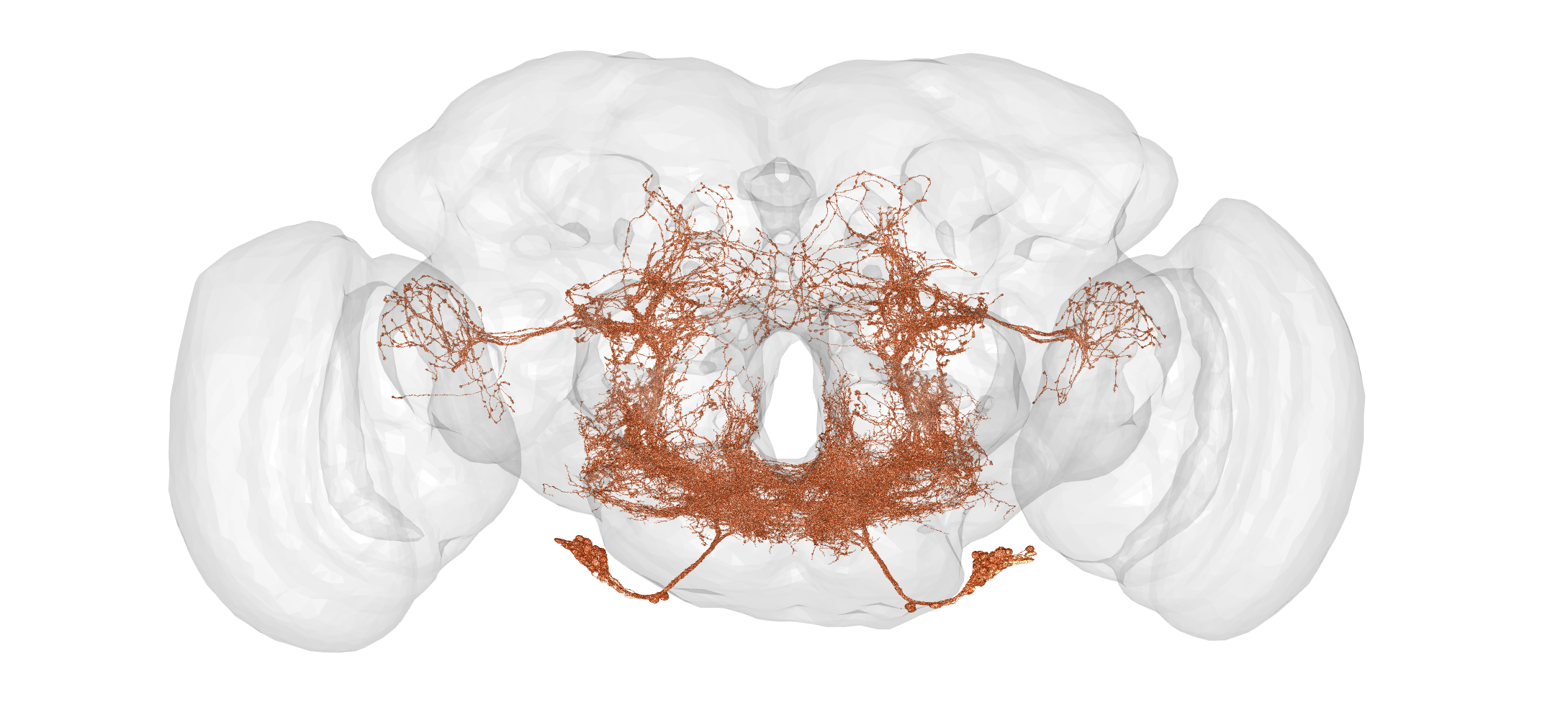

Supplement: Data S5. A .zip archive containing .png files depicting each of the 183 brain hemilineages we have used from the FAFB-FlyWire dataset, related to Figure 7 — Neurons in each hemilineage are colored by their neuron-level transmitter predictions, hemilineage names given in the file name. Hemilineage labels for the FAFB-FlyWire dataset are fully reported in Schlegel et al.S2 [file mmc6.zip › chosen_hemilineages/MX7__fafb.png]

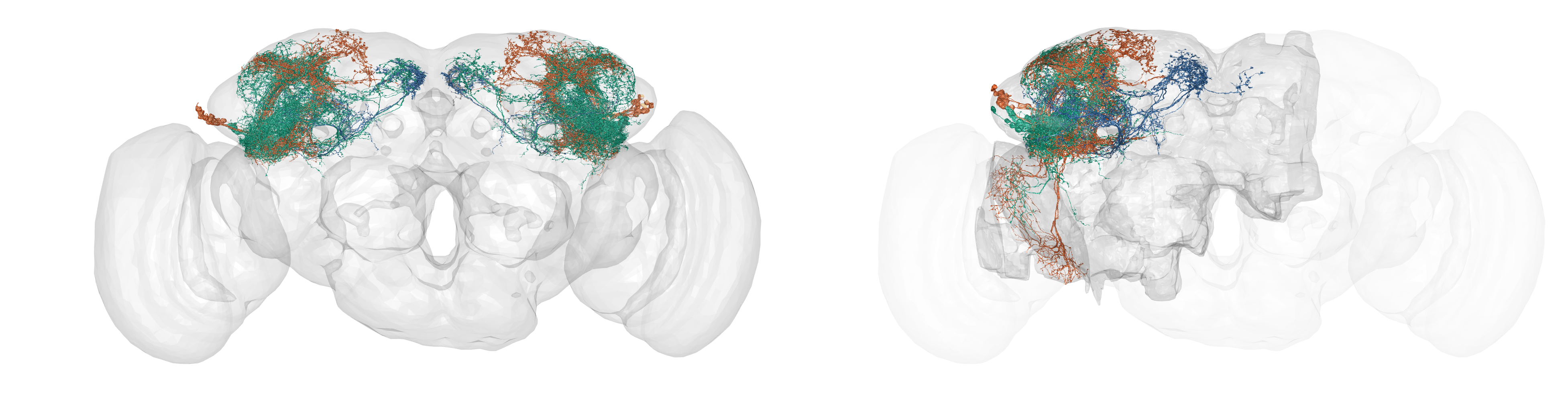

Supplement: Data S5. A .zip archive containing .png files depicting each of the 183 brain hemilineages we have used from the FAFB-FlyWire dataset, related to Figure 7 — Neurons in each hemilineage are colored by their neuron-level transmitter predictions, hemilineage names given in the file name. Hemilineage labels for the FAFB-FlyWire dataset are fully reported in Schlegel et al.S2 [file mmc6.zip › chosen_hemilineages/SLPpl2__fafb_hemibrain.png]

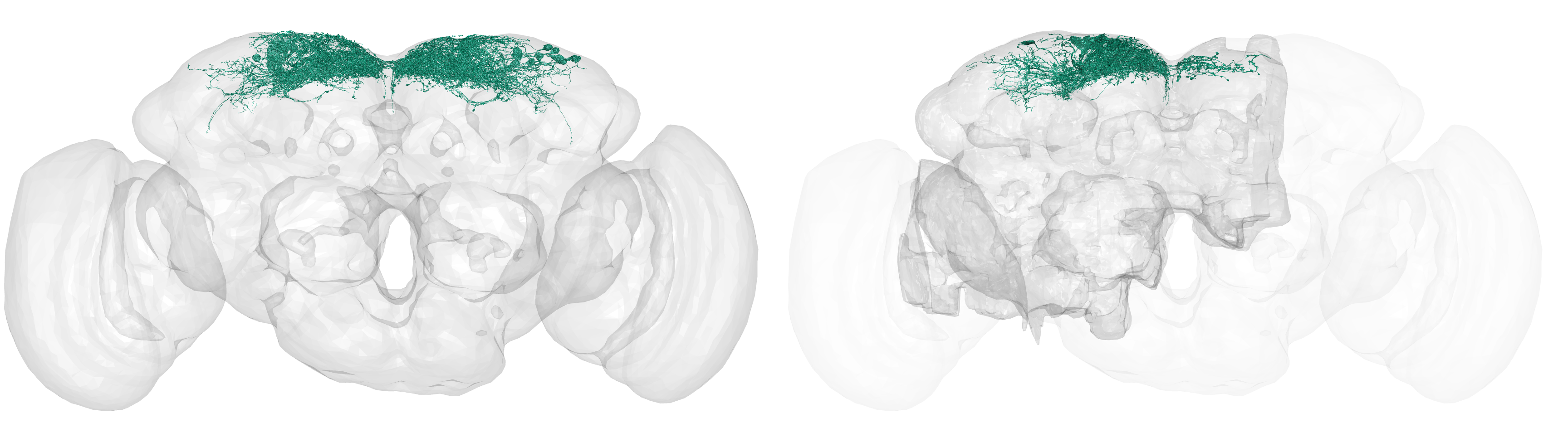

Supplement: Data S5. A .zip archive containing .png files depicting each of the 183 brain hemilineages we have used from the FAFB-FlyWire dataset, related to Figure 7 — Neurons in each hemilineage are colored by their neuron-level transmitter predictions, hemilineage names given in the file name. Hemilineage labels for the FAFB-FlyWire dataset are fully reported in Schlegel et al.S2 [file mmc6.zip › chosen_hemilineages/SMPpd2__fafb_hemibrain.png]

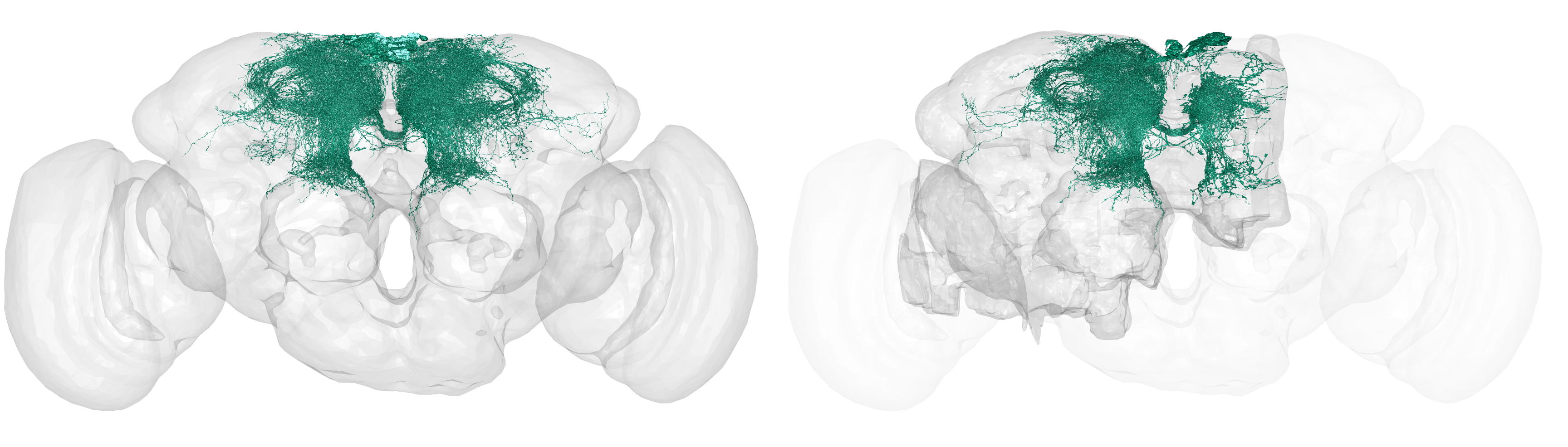

Supplement: Data S5. A .zip archive containing .png files depicting each of the 183 brain hemilineages we have used from the FAFB-FlyWire dataset, related to Figure 7 — Neurons in each hemilineage are colored by their neuron-level transmitter predictions, hemilineage names given in the file name. Hemilineage labels for the FAFB-FlyWire dataset are fully reported in Schlegel et al.S2 [file mmc6.zip › chosen_hemilineages/SMPad3__fafb_hemibrain.png]

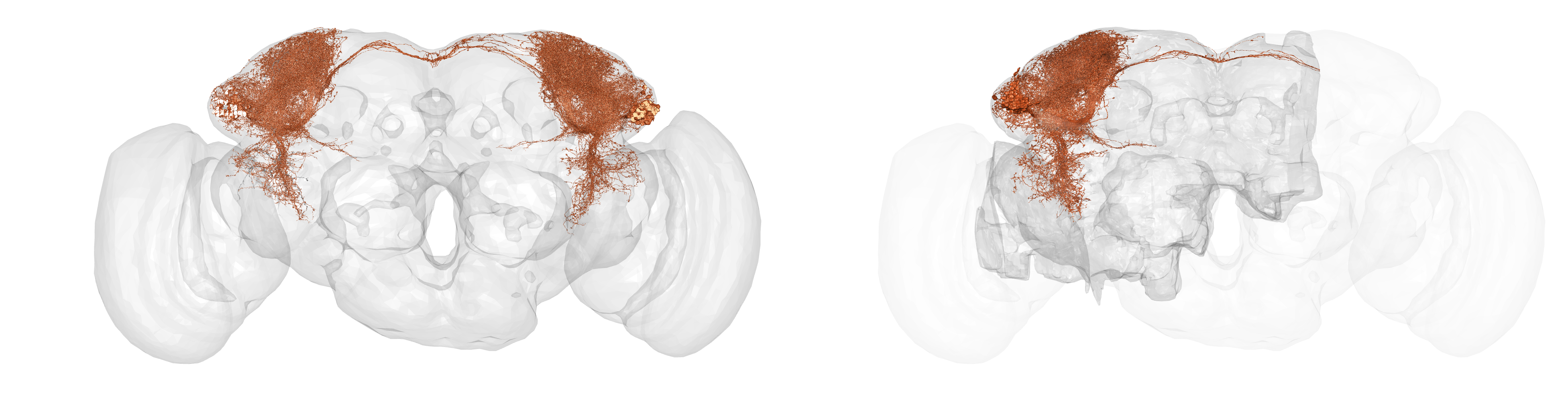

Supplement: Data S5. A .zip archive containing .png files depicting each of the 183 brain hemilineages we have used from the FAFB-FlyWire dataset, related to Figure 7 — Neurons in each hemilineage are colored by their neuron-level transmitter predictions, hemilineage names given in the file name. Hemilineage labels for the FAFB-FlyWire dataset are fully reported in Schlegel et al.S2 [file mmc6.zip › chosen_hemilineages/SLPal2__fafb_hemibrain.png]

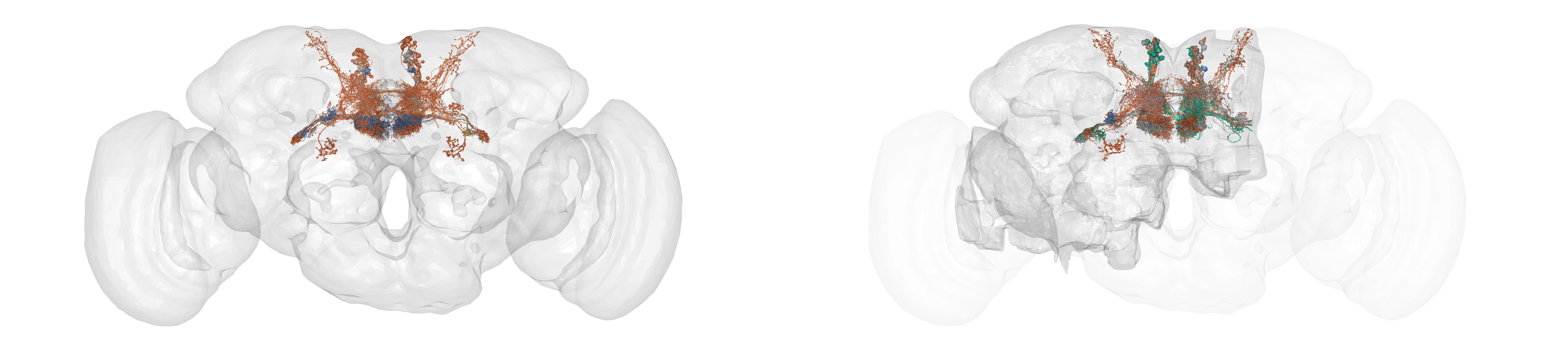

Supplement: Data S5. A .zip archive containing .png files depicting each of the 183 brain hemilineages we have used from the FAFB-FlyWire dataset, related to Figure 7 — Neurons in each hemilineage are colored by their neuron-level transmitter predictions, hemilineage names given in the file name. Hemilineage labels for the FAFB-FlyWire dataset are fully reported in Schlegel et al.S2 [file mmc6.zip › chosen_hemilineages/DM2_CX_d1__fafb_hemibrain.png]

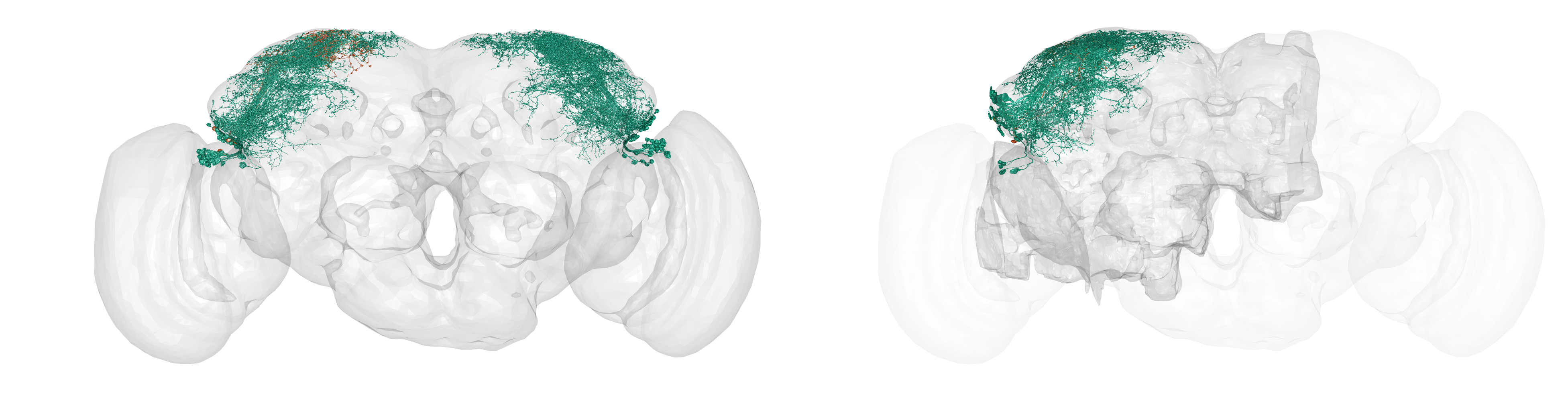

Supplement: Data S5. A .zip archive containing .png files depicting each of the 183 brain hemilineages we have used from the FAFB-FlyWire dataset, related to Figure 7 — Neurons in each hemilineage are colored by their neuron-level transmitter predictions, hemilineage names given in the file name. Hemilineage labels for the FAFB-FlyWire dataset are fully reported in Schlegel et al.S2 [file mmc6.zip › chosen_hemilineages/SLPpl3__fafb_hemibrain.png]

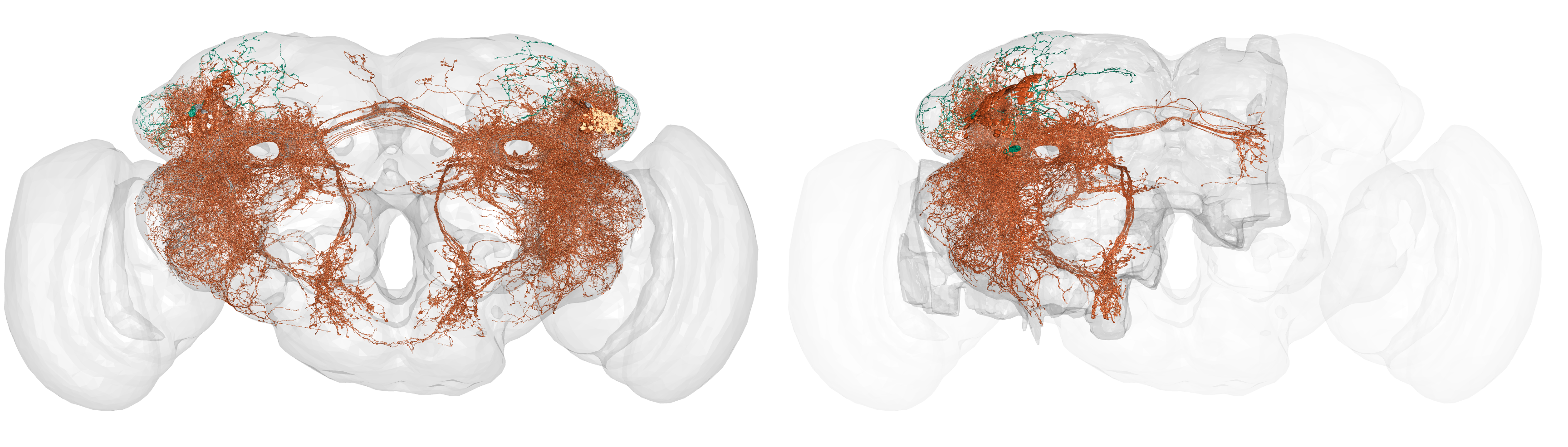

Supplement: Data S5. A .zip archive containing .png files depicting each of the 183 brain hemilineages we have used from the FAFB-FlyWire dataset, related to Figure 7 — Neurons in each hemilineage are colored by their neuron-level transmitter predictions, hemilineage names given in the file name. Hemilineage labels for the FAFB-FlyWire dataset are fully reported in Schlegel et al.S2 [file mmc6.zip › chosen_hemilineages/VLPd1__fafb_hemibrain.png]

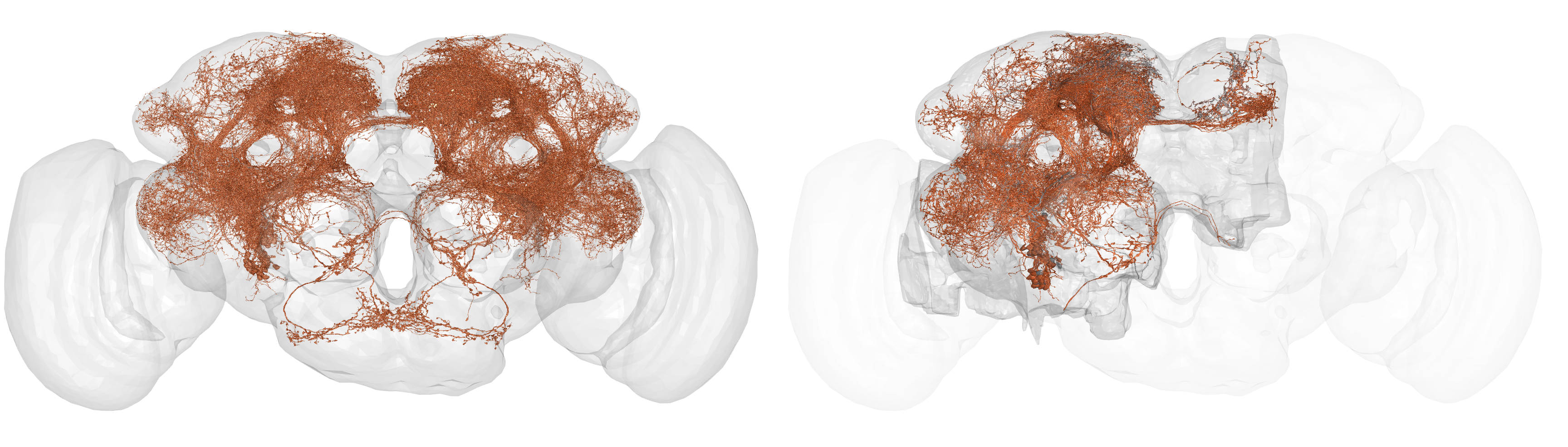

Supplement: Data S5. A .zip archive containing .png files depicting each of the 183 brain hemilineages we have used from the FAFB-FlyWire dataset, related to Figure 7 — Neurons in each hemilineage are colored by their neuron-level transmitter predictions, hemilineage names given in the file name. Hemilineage labels for the FAFB-FlyWire dataset are fully reported in Schlegel et al.S2 [file mmc6.zip › chosen_hemilineages/VLPl&p1_posterior__fafb_hemibrain.png]

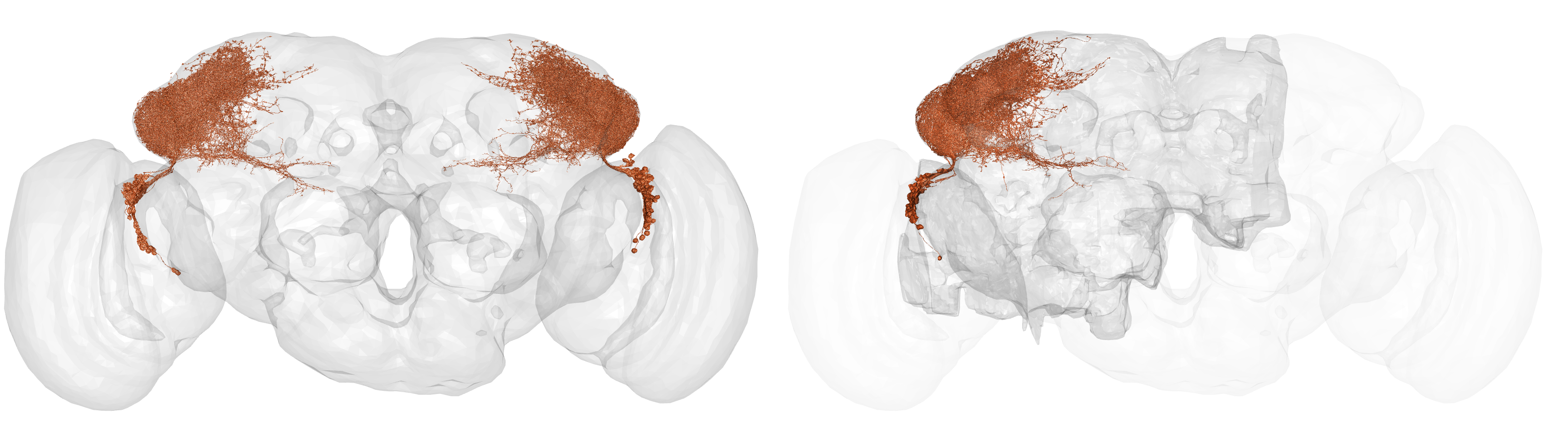

Supplement: Data S5. A .zip archive containing .png files depicting each of the 183 brain hemilineages we have used from the FAFB-FlyWire dataset, related to Figure 7 — Neurons in each hemilineage are colored by their neuron-level transmitter predictions, hemilineage names given in the file name. Hemilineage labels for the FAFB-FlyWire dataset are fully reported in Schlegel et al.S2 [file mmc6.zip › chosen_hemilineages/LHa2__fafb_hemibrain.png]

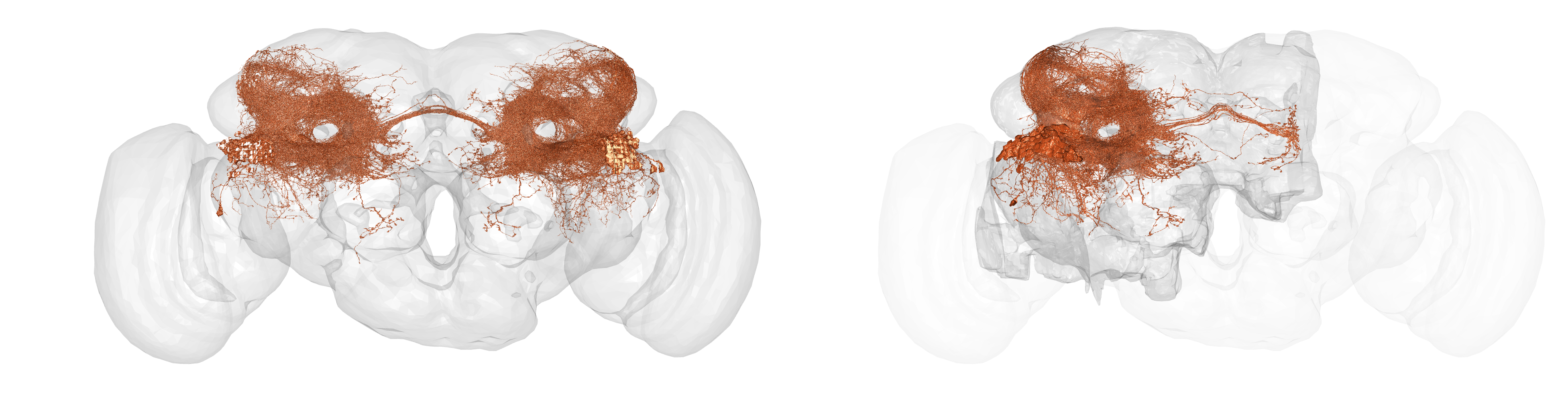

Supplement: Data S5. A .zip archive containing .png files depicting each of the 183 brain hemilineages we have used from the FAFB-FlyWire dataset, related to Figure 7 — Neurons in each hemilineage are colored by their neuron-level transmitter predictions, hemilineage names given in the file name. Hemilineage labels for the FAFB-FlyWire dataset are fully reported in Schlegel et al.S2 [file mmc6.zip › chosen_hemilineages/SLPa&l1_anterior__fafb_hemibrain.png]

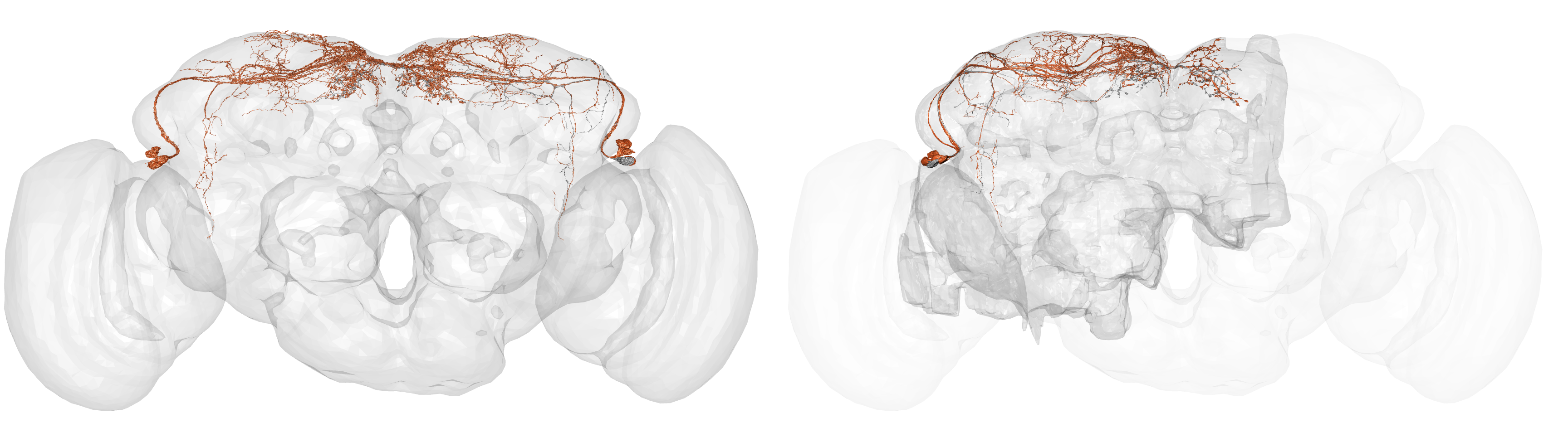

Supplement: Data S5. A .zip archive containing .png files depicting each of the 183 brain hemilineages we have used from the FAFB-FlyWire dataset, related to Figure 7 — Neurons in each hemilineage are colored by their neuron-level transmitter predictions, hemilineage names given in the file name. Hemilineage labels for the FAFB-FlyWire dataset are fully reported in Schlegel et al.S2 [file mmc6.zip › chosen_hemilineages/SLPav1_lateral__fafb_hemibrain.png]

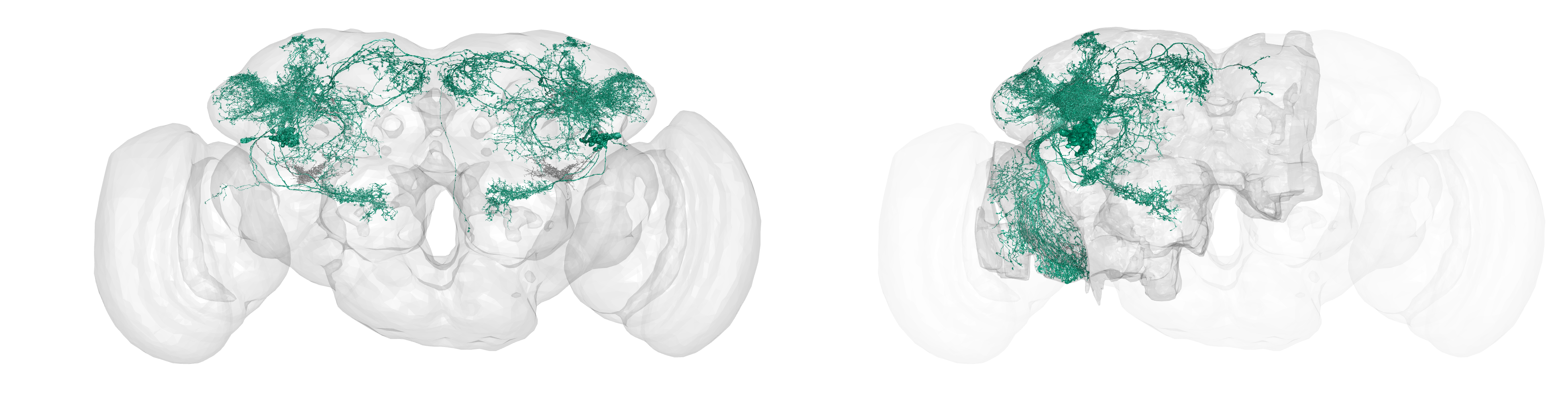

Supplement: Data S5. A .zip archive containing .png files depicting each of the 183 brain hemilineages we have used from the FAFB-FlyWire dataset, related to Figure 7 — Neurons in each hemilineage are colored by their neuron-level transmitter predictions, hemilineage names given in the file name. Hemilineage labels for the FAFB-FlyWire dataset are fully reported in Schlegel et al.S2 [file mmc6.zip › chosen_hemilineages/AOTUv2__fafb_hemibrain.png]

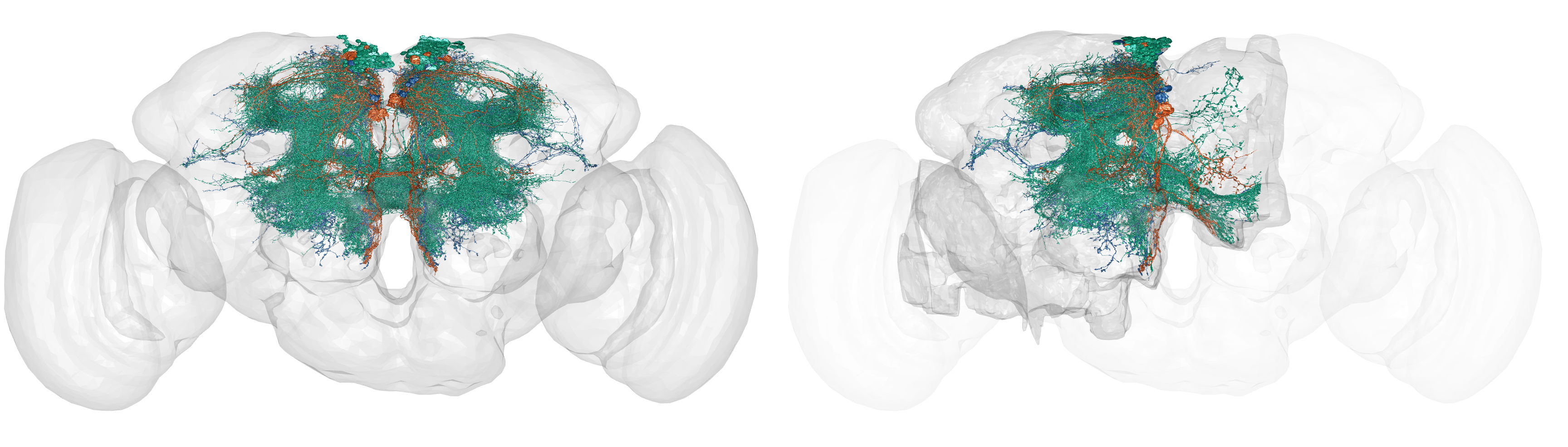

Supplement: Data S5. A .zip archive containing .png files depicting each of the 183 brain hemilineages we have used from the FAFB-FlyWire dataset, related to Figure 7 — Neurons in each hemilineage are colored by their neuron-level transmitter predictions, hemilineage names given in the file name. Hemilineage labels for the FAFB-FlyWire dataset are fully reported in Schlegel et al.S2 [file mmc6.zip › chosen_hemilineages/SMPad2__fafb_hemibrain.png]

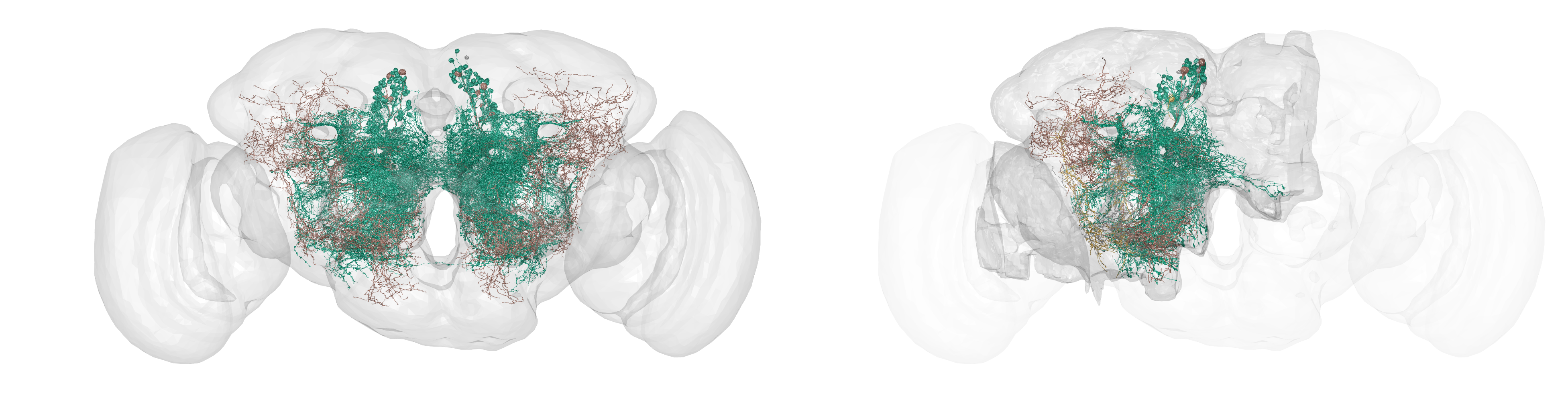

Supplement: Data S5. A .zip archive containing .png files depicting each of the 183 brain hemilineages we have used from the FAFB-FlyWire dataset, related to Figure 7 — Neurons in each hemilineage are colored by their neuron-level transmitter predictions, hemilineage names given in the file name. Hemilineage labels for the FAFB-FlyWire dataset are fully reported in Schlegel et al.S2 [file mmc6.zip › chosen_hemilineages/DM2_or_DM3_posterior__fafb_hemibrain.png]

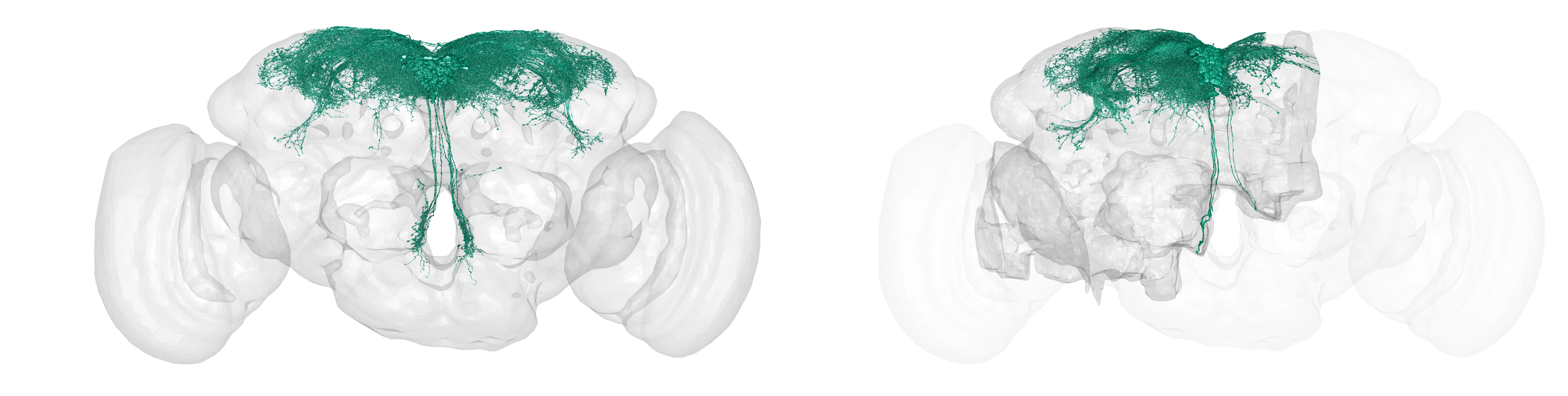

Supplement: Data S5. A .zip archive containing .png files depicting each of the 183 brain hemilineages we have used from the FAFB-FlyWire dataset, related to Figure 7 — Neurons in each hemilineage are colored by their neuron-level transmitter predictions, hemilineage names given in the file name. Hemilineage labels for the FAFB-FlyWire dataset are fully reported in Schlegel et al.S2 [file mmc6.zip › chosen_hemilineages/SMPad1__fafb_hemibrain.png]

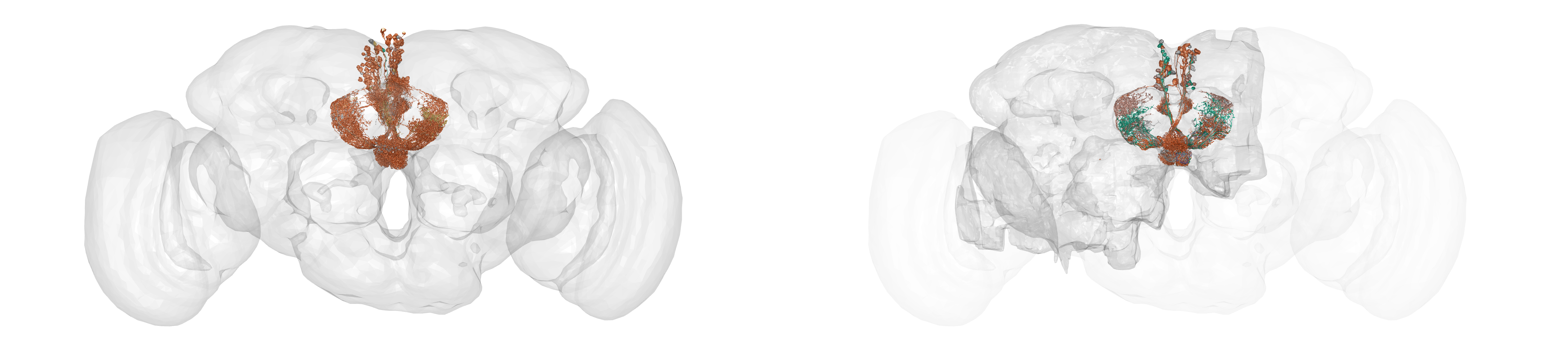

Supplement: Data S5. A .zip archive containing .png files depicting each of the 183 brain hemilineages we have used from the FAFB-FlyWire dataset, related to Figure 7 — Neurons in each hemilineage are colored by their neuron-level transmitter predictions, hemilineage names given in the file name. Hemilineage labels for the FAFB-FlyWire dataset are fully reported in Schlegel et al.S2 [file mmc6.zip › chosen_hemilineages/DM1_CX_p__fafb_hemibrain.png]
